# Divergent Access to α,β-Unsaturated Thiolated and Selenolated Lactams via a Unified Palladium-Catalyzed Carbonylative Transformation

**DOI:** 10.1021/acs.orglett.5c05383

**Published:** 2026-01-21

**Authors:** Zhiping Yin, Shuhui Sun, Xiaowen Qin, Heng Li, Fengxiang Zhu, Xiao-Feng Wu

**Affiliations:** † School of Pharmacy, 12676 Jiangsu University, Zhenjiang 212013, China; § School of Chemistry and Chemical Engineering, 12441 Shanxi University, Wucheng Road 92, Taiyuan 030006, China; ‡ Dalian National Laboratory for Clean Energy, Dalian Institute of Chemical Physics, Chinese Academy of Sciences, 116023 Dalian, Liaoning, China

## Abstract

Herein, a unified and divergent palladium-catalyzed carbonylative procedure for the direct synthesis of α,β-unsaturated thiolated and selenolated lactams is reported. This strategy enables the simultaneous construction of the lactam core and installation of valuable sulfur or selenium functionalities via a single catalytic operation. By employing simple propargylamine substrates together with diphenyl disulfide or diselenide under a carbon monoxide atmosphere, the corresponding lactams were formed in moderate to good yields with promising functional group tolerance.

## Graphical abstract

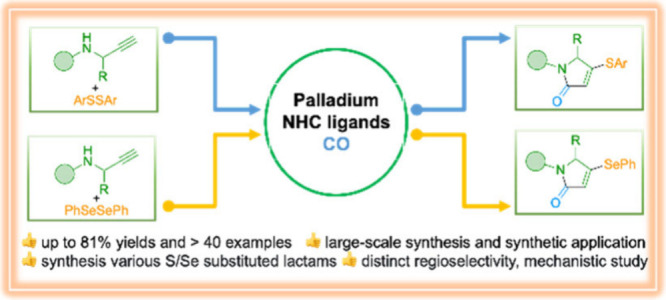

α,β-Unsaturated amides, particularly in the form of lactams, constitute a privileged structural motif in medicinal chemistry and chemical biology.^1^ Their electrophilic α,β-unsaturated carbonyl system serves as a key handle for irreversible engagement with biological nucleophiles, such as cysteine residues, via conjugate addition, making them invaluable in the design of covalent inhibitors and bioactive probes.^2^ Consequently, the development of efficient and modular synthetic methods to access diversely functionalized unsaturated lactams remains a high-priority objective in synthetic organic chemistry.

Transition-metal-catalyzed carbonylative cyclizations have emerged as a powerful and direct strategy for constructing heterocyclic cores from unsaturated precursors and CO gas (Scheme 1a).^3^ Among these transformations, the hydroformylative cyclization of alkyne-tethered amines represents a well-established route to lactams.^4^ However, this approach is inherently constrained by its reliance on the incorporation of a hydrogen atom across the alkyne, thereby failing to introduce versatile functional handles that would enable further molecular diversification. In parallel, transition-metal-catalyzed intramolecular oxidative carbonylative difunctionalization of alkynes offers a complementary route to various heterocycles. A representative example is the work by Gabriele and co-workers, who achieved the synthesis of (*Z*)-α-(methoxycarbonyl)­methylene-β-lactams from propargylamines using a catalytic system comprising PdI_2_ and KI (Scheme 1b).^5^ While such oxidative carbonylative methodologies expand structural diversity, they predominantly follow a β-selective cyclization pathway and typically depend on external oxidants. Consequently, the direct and selective installation of valuable heteroatomic functionalities—such as sulfur or selenium, each of which is known to modulate pharmacokinetic profiles and serve as synthetic handles for further elaboration—specifically at the α-position of the alkyne during lactam formation, remains a significant and unmet challenge.^6^

**Scheme 1:**
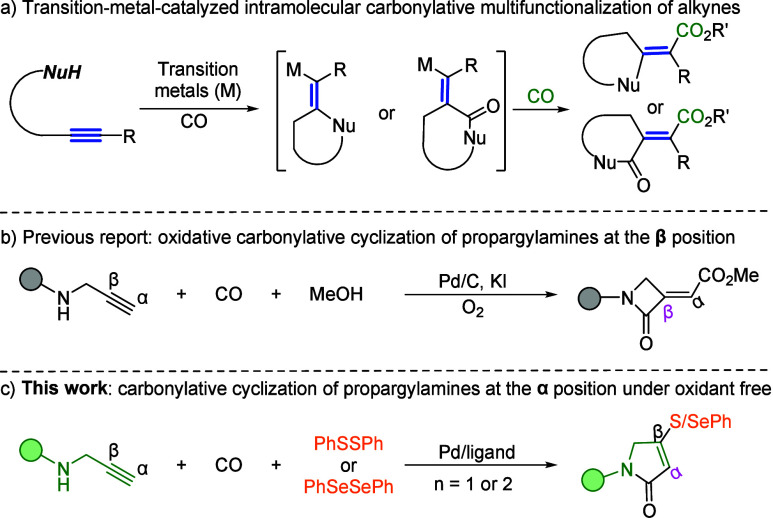
Investigation of Carbonylative Multifunctionalization of Alkynes

Although notable progress has been made in intermolecular seleno- and thiocarbonylation of alkynes, demonstrating the utility of (PhSe)_2_ or (PhS)_2_ for assembling acyclic β-functionalized acrylates,^7^ the translation of this difunctionalization logic into the more demanding context of intramolecular carbonylative cyclization has not been realized, representing a conspicuous gap in synthetic methodology. Notably, in 1997, Sonoda, Ogawa, and co-workers developed a palladium-catalyzed carbonylative cyclization of propargyl alcohols with diaryl diselenides and diaryl disulfide.^6h^ Various lactones were formed in moderate to good yields with Pd­(PPh_3_)_4_ as the catalyst.

Inspired by these precedents and driven by our group’s long-standing focus on carbonylative heterocycle synthesis,^8^ we now report a distinct catalytic paradigm that directly addresses this formidable challenge (Scheme 1c). This method enables an oxidant-free, α-selective carbonylative cyclization of propargylamines, directly constructing the α,β-unsaturated lactam scaffold. The key innovation lies in the synergistic interception of the pivotal vinyl-palladium intermediate by dichalcogenides (PhSSPh or PhSeSePh). This seamless merger of cyclization with C–S or C–Se bond formation achieves a dual breakthrough. It fundamentally shifts the selectivity from the traditional β-position to the novel α-position of alkynes and concurrently installs valuable sulfur or selenium functionalities at the β-position of alkynes. This mild and modular protocol not only provides a unified route to previously elusive, densely functionalized heterocyclic building blocks but also exhibits remarkable divergence, enabling the programmable synthesis of both five- and six-membered lactams from a common set of starting materials simply by varying the tether length.

Building upon the established importance of selenolated lactams, we initiated our study by exploring the carbonylative cyclization of *N*-benzylprop-2-yn-1-amine (**1a**) with diphenyl diselenide (**2a′**) as a model substrate. While our initial attempt using molybdenum hexacarbonyl as a solid CO surrogate proved to be unsuccessful, switching to carbon monoxide gas enabled the formation of the desired 1-benzyl-4-(phenylselanyl)-1,5-dihydro-2*H*-pyrrol-2-one (**4a**). This preliminary conversion was achieved using Pd­(PPh_3_)_4_ with additional PPh_3_ as a ligand in toluene at 90 °C (see the Supporting Information). Systematic screening of phosphine ligands following this initial result, however, did not lead to positive improvements in yield. A pivotal advancement came with the observation that N-heterocyclic carbene (NHC) ligands consistently outperformed their phosphine counterparts, which might be due to NHC coordinating more strongly with Pd.^9,10^ Guided by this finding, we evaluated several catalysts and additives (Table 1, entries 2–4, and Supporting Information). Although common additives showed limited effects, the introduction of molecular sieves (MS) notably enhanced the reaction efficiency. The possible reason might be the presence of moisture in the system, which led noncarbonylation or hydrosulfuration as the main side reaction. Capitalizing on the superior performance of NHC ligands, we subsequently screened a series of such ligands (Table 1) and identified ligand **7** as being optimal. Further optimization of the reaction medium led to the adoption of a toluene/DMSO mixed solvent system, ultimately delivering model product **4a** in 74% yield. Here, we believe that the addition of DMSO can increase the solubility of the selenium reagent. Notably, due to the high coordinating and toxicity properties of sulfur to palladium compared with Se, more steric ligand is needed to achieve better results. With the optimized protocol for selenium incorporation established, we next examined the generality of our catalytic system by extending it to the analogous sulfurative cyclization using diphenyl disulfide (**2a**). Employing substrate **1a** under comparable conditions, optimization of the ligand and solvent identified DME as the preferred solvent, with Pd­(PPh_3_)_4_ and ligand **1** serving as cocatalysts under 5 bar of CO at 90 °C as the optimal setup for the thiolated lactam synthesis. The reaction efficiency for both transformations decreased dramatically in the absence of a catalyst, a ligand, or molecular sieves (Table 1, entries 8–10).

**Table 1:**
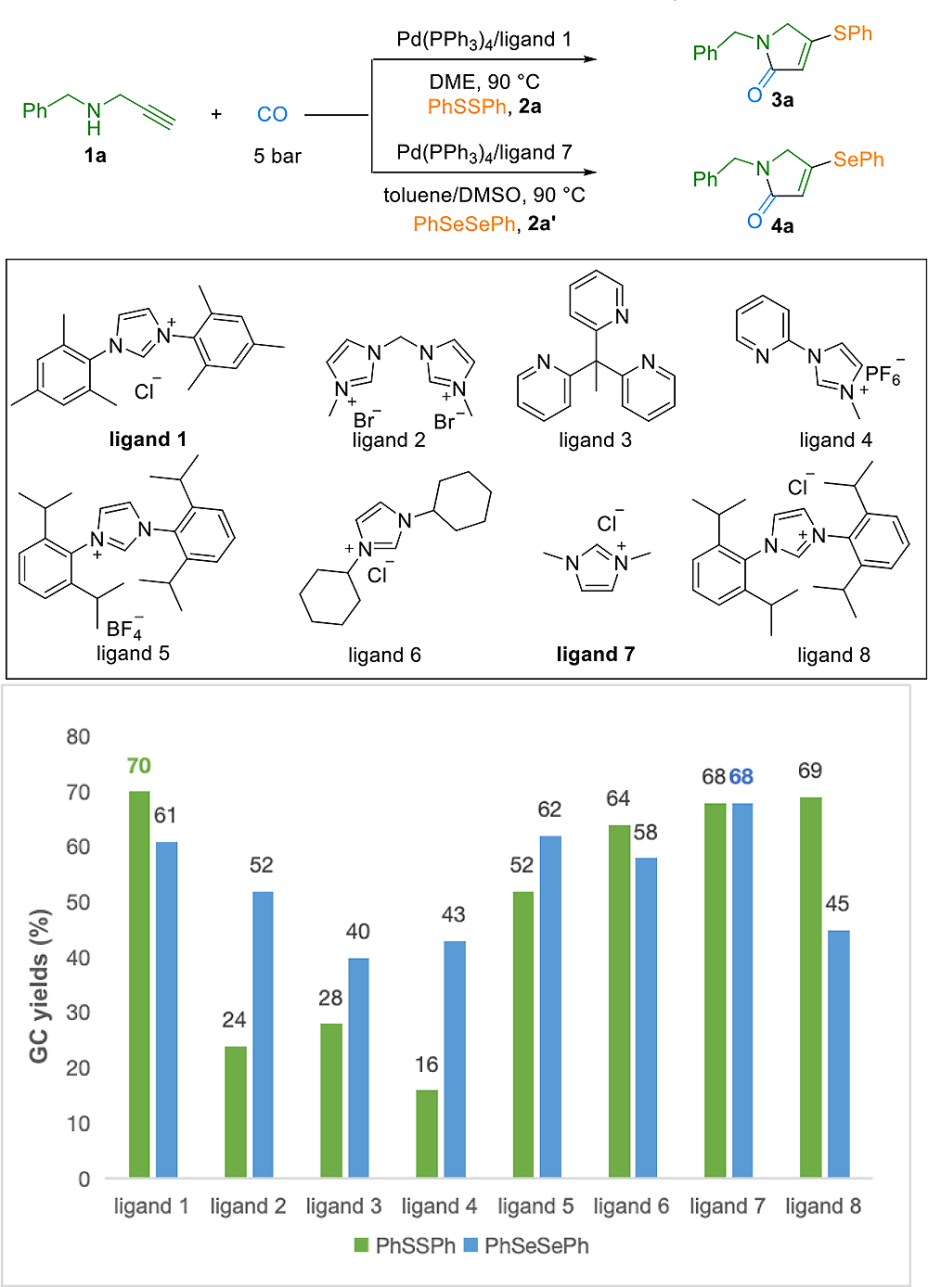
Optimization of the Reaction Conditions^a^,^b^

entry	conditions	yield^c^ of **3a** (%)	yield^c^ of **4a** (%)
1	none	78 (72^d^)	74 (70^d^)
2^e^	Pd_2_dba_3_	55	49
3^e^	PdCl_2_(PPh_3_)_2_	50	40
4^e^	Pd(PPh_3_)_4_	70	61
5^f^	toluene	64	52
6^f^	DMSO	60	50
7^f^	DME	70	51
8	without Pd(PPh_3_)_4_	NR^a^	NR^a^
9	without a ligand	26	12
10	without MS	40	39

a Reaction conditions: **2a** (18.5 mg, 0.085 mmol, 0.85 equiv), **1a** (14 μL, 0.1 mmol, 1.0 equiv), Pd­(PPh_3_)_4_ (2 mol %), ligand **1** (10 mol %), molecular sieves (100 mg), DME (1 mL), 5 bar of CO, 90 °C, 24 h.

b Reaction conditions: **2a′** (26.5 mg, 0.085 mmol, 0.85 equiv), **1a** (14 μL, 0.1 mmol, 1.0 equiv), Pd­(PPh_3_)_4_ (5 mol %), ligand **7** (20 mol %), molecular sieves (100 mg), toluene (0.8 mL), DMSO (0.2 mL), 5 bar of CO, 90 °C, 24 h.

c GC yields.

d Isolated yields.

e **2a**/**2a′** (0.1 mmol, 1.0 equiv), **1a** (14 μL, 0.1 mmol, 1.0 equiv), Pd­(PPh_3_)_4_ (2 or 5 mol %), ligand **1** (10 mol %), molecular sieves (50 mg), solvent (1 mL), 90 °C, 24 h.

f **2a**/**2a′** (0.1 mmol, 1.0 equiv), **1a** (14 μL, 0.1 mmol, 1.0 equiv), Pd­(PPh_3_)_4_ (2 or 5 mol %), ligand **1** (10 mol %), molecular sieves (50 mg), solvent (1 mL), 5 bar of CO, 90 °C, 24 h.

g No reaction.

Under the optimized reaction conditions, we systematically evaluated the substrate scope of this palladium-catalyzed carbonylative cascade process. As summarized in Table 2, the transformation demonstrated excellent functional group compatibility, delivering a diverse array of α,β-unsaturated thiolated lactams in moderate to excellent yields.

**Table 2:**
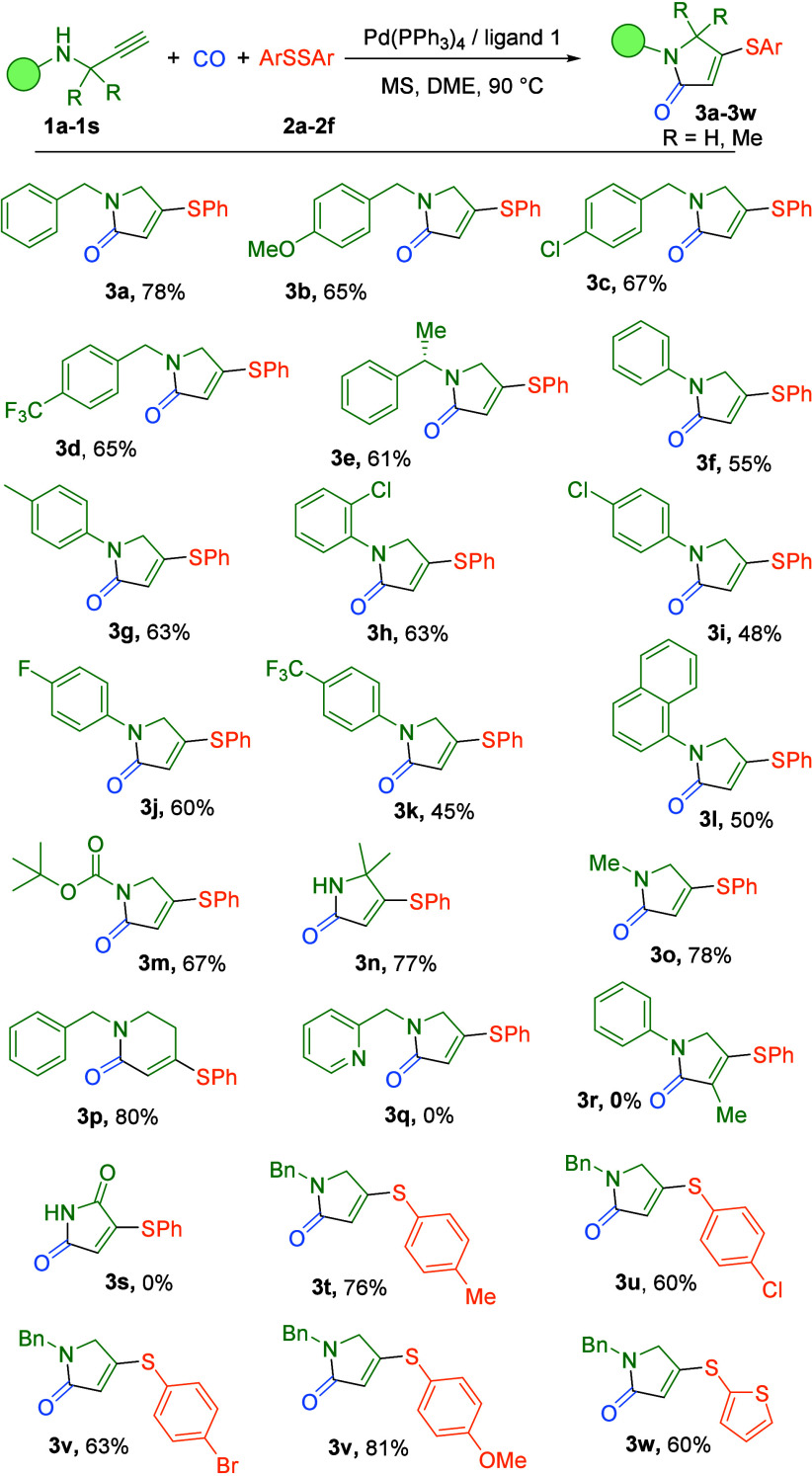
Carbonylative Synthesis of α,β-Unsaturated Thiolated Lactams^a^

a Reaction conditions: **2a**–**2f** (0.17 mmol, 0.85 equiv), **1a**–**1s** (0.2 mmol, 1.0 equiv), Pd­(PPh_3_)_4_ (2 mol %), ligand **1** (10 mol %), MS (200 mg), DME (2 mL), 5 bar of CO, 90 °C, 24 h. Isolated yields were determined as the average of two independent runs.

Systematic evaluation of electronic effects revealed that benzylic alkynes bearing either electron-donating (MeO) or electron-withdrawing (Cl and CF_3_) substituents participated effectively in the transformation, affording the corresponding products in 65–67% yields (Table 2, **3b–3d**). Notably, the reaction proved to be compatible with a chiral substrate derived from (*S*)-1-phenylethan-1-amine, affording the desired lactam in 61% yield (Table 2, **3e**). The protocol was further extended to aromatic amine-derived alkynes bearing various substituents, with both electron-donating (Me) and electron-withdrawing groups (Cl, F, and CF_3_) being well tolerated (45–63% yields) (Table 2, **3f–3l**). Investigation of amine protecting groups demonstrated that diverse nitrogen functionalities, including Boc-protected, *N*-methyl, and free amine variants, all underwent smooth conversion to the target lactams (67–78% yields) (Table 2, **3m–3o**). Particularly significant was the successful formation of a six-membered lactam ring from a homoallylic amine substrate in 80% yield, highlighting the method’s versatility in ring-size control (Table 2, **3p**). For pyridine-substituted alkyne substrates, internal alkynes, and amide-linked alkynes, the carbonylative transformation proved to be ineffective, yielding no target product (Table 2, **3q–3** **s**). To further establish the reaction generality, we examined the scope of disulfide coupling partners using alkyne **1a** as the standard substrate. The method accommodated various *para*-substituted diphenyl disulfides (Me, Cl, Br, and OMe) with comparable efficiency (60–81% yields) (Table 2, **3t**–**3v**). Importantly, heteroaromatic 1,2-di­(thiophen-2-yl)­disulfane also participated effectively, delivering the thiolated product in 60% yield (Table 2, **3w**).

Subsequently, recognizing the significant biological profiles of selenolated lactams, we extended our catalytic system to the direct construction of this valuable scaffold. As summarized in Table 3, this selenium variant exhibited excellent compatibility with the full range of alkyne substrates previously investigated. Benzyl alkynes bearing electronically diverse substituents (MeO, Cl, and CF_3_) delivered the corresponding selenolated products in 62–74% yields, showing the same efficiency compared to their sulfur analogues (Table 3, **4a**–**4d**). The chiral (*S*)-1-phenylethan-1-amine-derived alkyne also provides the selenolactam in 62% yield (Table 3, **4e**). Aromatic amine-tethered alkynes with both electron-donating and -withdrawing groups afforded products in 47–66% yields, demonstrating consistent electronic tolerance (Table 3, **4f**–**4l**). Various nitrogen protecting groups (Boc, free NH_2_) all proved to be viable under the selenium conditions, affording products in 57–66% yields (Table 3, **4m** and **4n**). Notably, the six-membered ring formation proceeded with acceptable efficiency, achieving 58% yield from the homoallylic amine substrate (Table 3, **4o**). This systematic evaluation establishes the robust generality of our catalytic system for selenium incorporation.

**Table 3:**
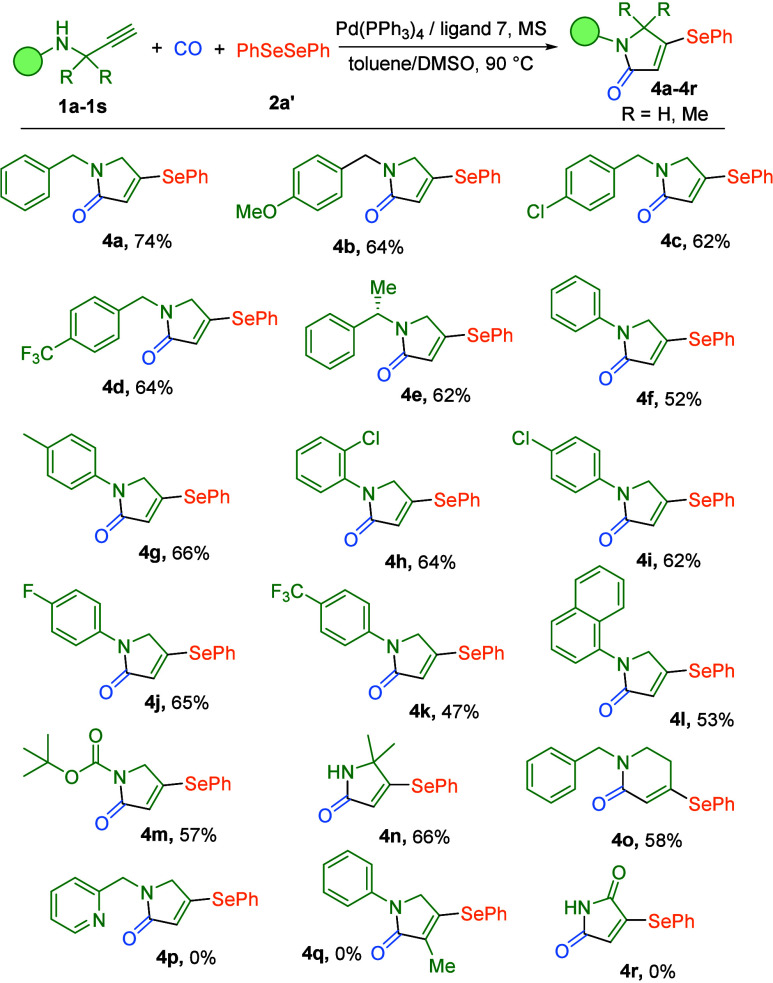
Carbonylative Synthesis of α,β-Unsaturated Selenolated Lactams^a^

a Reaction conditions: **2a′** (0.17 mmol, 0.85 equiv), **1a**–**1s** (0.2 mmol, 1.0 equiv), Pd­(PPh_3_)_4_ (5 mol %), ligand 7 (20 mol %), MS (200 mg), toluene (0.8 mL), DMSO (0.2 mL), 5 bar of CO, 90 °C, 24 h. Isolated yields were determined as the average of two independent runs.

To demonstrate the practical utility of this methodology, a gram-scale reaction was performed at a 4 mmol scale, affording thiolated lactam **3a** in 67% isolated yield with efficiency comparable to that of the small-scale standard reaction (Scheme 2, I). This result confirms the robustness and potential scalability of the protocol for industrial applications. Furthermore, the synthetic versatility of the selenolated lactam products was explored through a palladium-catalyzed Suzuki–Miyaura cross-coupling with phenylboronic acid, constructing a new C–C bond in 65% yield (Scheme 2, II). These successful transformations highlight the value of the α,β-unsaturated lactams as versatile synthetic intermediates and further establish the broad utility of the present carbonylative cascade strategy.

**Scheme 2:**
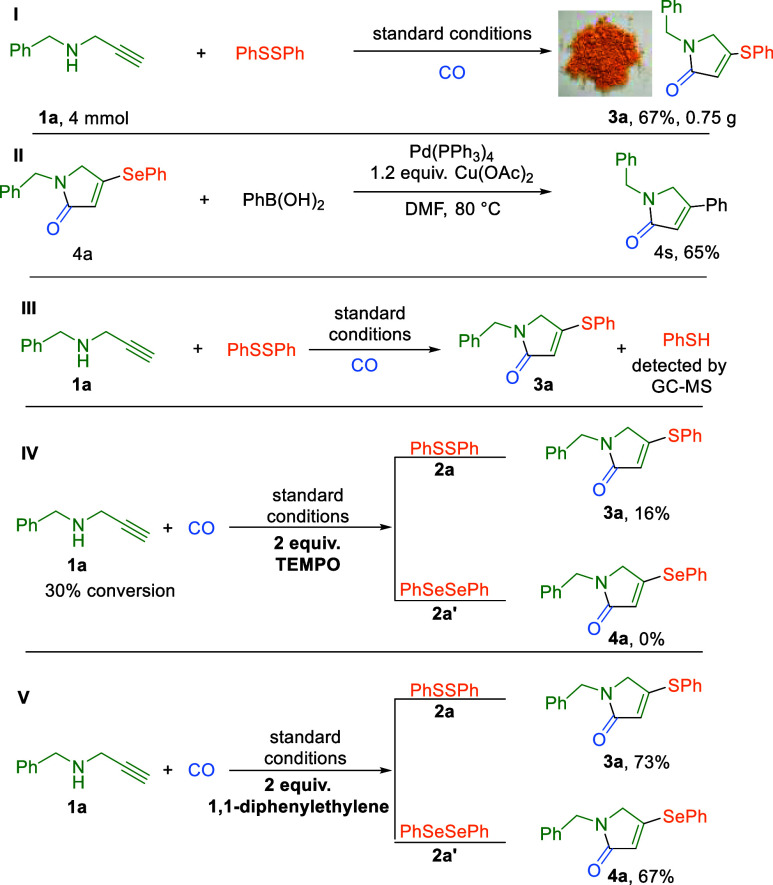
Large-Scale Synthesis, Synthetic Application, and Control Experiments

To gain deeper mechanistic insight into the carbonylative transformation, we performed a series of control experiments (Scheme 2, III–V). Under the standard conditions, the key intermediate benzenethiol, derived from S–S bond cleavage of **2a**, was successfully detected by GC–MS analysis (Scheme 2, III). The reaction pathway was further probed using radical scavengers, including TEMPO and 1,1-diphenylethylene (Scheme 2, IV). In the reactions with TEMPO, the yield of the desired products was inhibited significantly. However, due to the oxidizing property of TEMPO, we could not fully confirm the presence of the radical intermediate. Notably, the addition of 1,1-diphenylethylene did not suppress the transformation, affording products **3a** and **4a** in 73% and 67% yields, respectively, thereby ruling out the involvement of radical intermediates (Scheme 2, V).

Based on these experimental results and relevant literature precedents,^6h,11^ a plausible catalytic cycle is proposed in Figure 1. The cycle is initiated by oxidative addition of the S–S bond in **2a** to in situ-generated Pd(0), furnishing Pd­(II) thiolate complex **Int-1**. Subsequent coordination and regioselective insertion of the terminal alkyne into the Pd–S bond afford vinyl-palladium species **Int-2**. Carbon monoxide coordination and insertion then yield acyl-palladium intermediate **Int-3**, which undergoes stereoselective isomerization to give (*E*)-**Int-5**. Finally, the intramolecular cyclization of (*E*)-**Int-5** releases the lactam product along with benzenethiol, regenerating the active Pd(0) catalyst to close the cycle.

**Figure 1:**
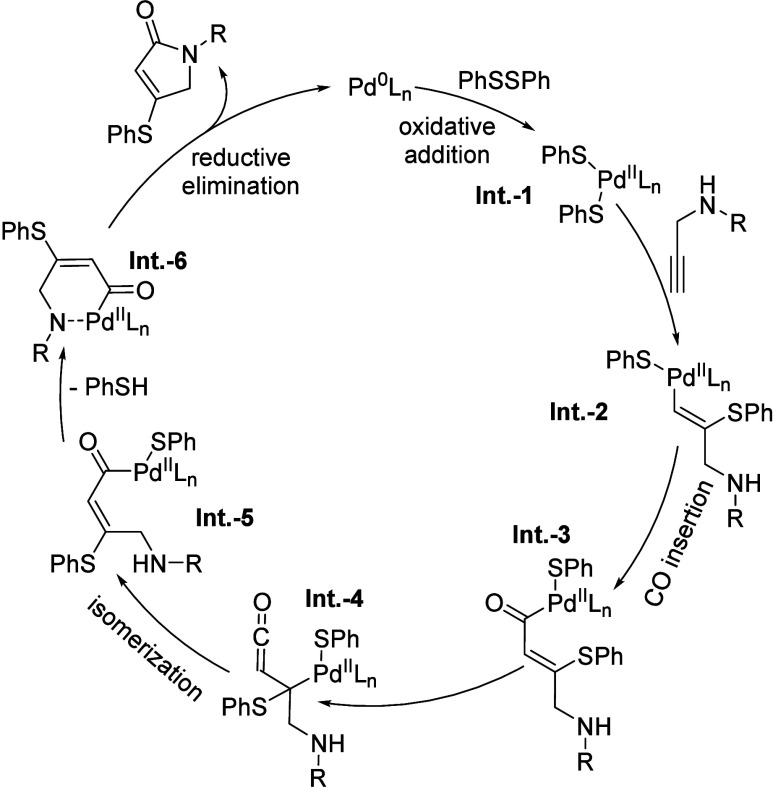
Proposed catalytic cycle.

In conclusion, we have developed a unified Pd-catalyzed carbonylative procedure that divergently delivers α,β-unsaturated thiolated or selenolated lactams from readily available propargylamines and dichalcogenides. This transformation establishes a distinct α-selective cyclization mode, contrasting with conventional β-selective carbonylative cyclization pathways, and concurrently introduces synthetically versatile PhS or PhSe groups at the alkenyl position. The method exhibits a broad substrate scope and scalable performance and enables further structural diversification of the lactam products through cross-coupling transformation. Beyond providing direct access to previously challenging heterocyclic architectures, this work introduces a programmable and multifunctionalization-enabled approach to carbonylative cyclization, opening new avenues for the efficient assembly of bioactive molecule scaffolds.

## Supplementary Material



## Data Availability

The data underlying this study are available in the published article and its Supporting Information.

## Notes

General information, general procedure, analytical data, and NMR spectra (PDF)
