# Supplementary material for: Divergent Access to α,β-Unsaturated Thiolated and Selenolated Lactams via a Unified Palladium-Catalyzed Carbonylative Transformation
Source: Org Lett. 2026 Jan 21;28(5):1832–7. doi: 10.1021/acs.orglett.5c05383 (PMC12887994; doi:10.1021/acs.orglett.5c05383)
Supplement: Supplementary file 1 [file ol5c05383_si_001.pdf]

Supporting Information

Divergent Access to α,β-Unsaturated Thiolated and Selenolated Lactams via a Unified Palladium-Catalysed Carbonylative Transformation

Zhiping Yin,*[a] Shuhui Sun,[a] Xiaowen Qin,[a] Heng Li,[a] Fengxiang Zhu,*[b] Xiao-Feng Wu,*[c]

[a] School of Pharmacy, Jiangsu University, Zhenjiang 212013, China, E-mail: zhiping_yin@ujs.edu.cn
[b] School of Chemistry and Chemical Engineering Shanxi University, Wucheng Road 92, Taiyuan 030006, China, E-mail: zfx201989@sxu.edu.cn
[c] Dalian National Laboratory for Clean Energy, Dalian Institute of Chemical Physics, Chinese Academy of Science, 116023 Dalian, Liaoning, China, E-mail: xwu2020@dicp.ac.cn

Contents

1. **General Comments** 2
2. **Experimental Setup** 3
   - **2.1 General Procedures for the synthesis of substrate 1a:** 3
   - **2.2 General Procedures for the synthesis of substrate 1b-1l, 1p and 1q:** 3
   - **2.3 Optimization of reaction conditions for substrate 2a** 4
   - **2.4 Optimization of reaction conditions for substrate 2a'** 7
   - **2.5 General Procedures for the synthesis of 3a-4s:** 14
   - **2.6 Gas Chromatography-mass spectrometry results.** 15
   - **2.7 Alkyne amine substrates of 1a-1s:** 16
   - **2.8 Substrate of 2a-2f,2a'** 16
3. **Characterization Data of Products** 17
4. **References** 28
5. **Copies of NMR Spectra of Products** 29

1. General Comments

All chemicals were purchased from Adamas, Energy Chemical, Bidepharm., TCI, Aladdin and used as such unless stated otherwise. All solvents like acetonitrile, tetrahydrofuran, N, N-dimethylmethanamide, 1,4-dioxane were purchased from Adamas (Water ≤ 30 ppm (by K.F.), 99.9%, SafeDry, with molecular sieves, Safeseal). NMR spectra were recorded on Bruker AV 400 or Bruker Fourier 300 spectrometer. Chemical shifts (ppm) are given relative to TMS (0.00 ppm) for 1H and $CDCl_3$ (77.0 ppm), DMSO-d6 (39.5 ppm) for 13C solvent. Multiplets were assigned as s (singlet), d (doublet), t (triplet), q (quartet), p (pentet), dd (doublet of doublet), m (multiplet) and br.s (broad singlet). High-resolution mass spectra HRMS spectra were recorded on a Thermo Scientific Exactive Orbitrap Mass Spectrometer under Electron Spray Ionization conditions preparing sample solution in methanol. The data are given as mass units per charge (m/z). GC yields were calculated using dodecane as an internal standard. Gas chromatography analysis was performed on an Agilent 6820 instrument with an FID detector and HP-5 capillary column (polydimethylsiloxane with 5% phenyl groups, 30 m, 0.32 mm i.d. 0.25 μm film thickness) using nitrogen as carrier gas. The products were isolated from the reaction mixture by column chromatography on silica gel., 54-74 μm, 200-300 mesh (Yucheng Chemical CO., LTD, Shanghai).
NOTE: Due to the reaction involving carbon monoxide (CO) gas, the reactions should only be handled in a well-ventilated fume hood and the laboratory should be well-equipped with a CO detector and alarm system.

2. Experimental Setup

2.1 General Procedures for the synthesis of substrate 1a[1]:

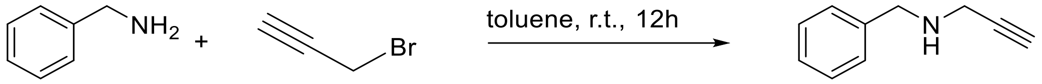

To a 100 mL round-bottom flask equipped with a stir bar were added benzylamine (120 mmol, 12.86 g) and toluene (30 mL). The solution was cooled to 0 °C in an ice bath, and propargyl bromide (20 mmol, 2.38 g) was added dropwise. After the addition, the reaction mixture was stirred at room temperature for 12 h, and the reaction progress was monitored by TLC. Upon completion, the mixture was diluted with water (20 mL) and extracted with ethyl acetate (3 × 20 mL). The combined organic layers were dried over anhydrous Na_2SO_4, filtered, and concentrated under reduced pressure. The crude residue was purified by flash column chromatography (petroleum ether/ethyl acetate) to afford the desired product **1a**.

2.2 General Procedures for the synthesis of substrate 1b-1l, 1p and 1q [2]:

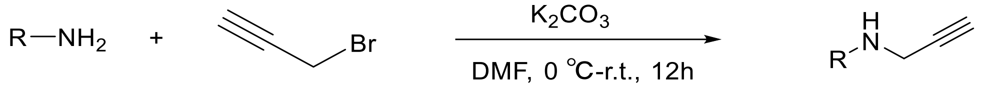

A solution of the substituted amine (9 mmol) and K_2CO_3 (622 mg, 4.5 mmol) in DMF (10 mL) was stirred at 0 °C for 5 minutes. To this mixture was added a solution of 3-bromopropyne (536 mg, 4.5 mmol) or 3-bromobutyne (598 mg, 4.5 mmol) in DMF (10 mL) dropwise. The resulting mixture was stirred at room temperature for 12 h, and the reaction progress was monitored by TLC. After completion, the reaction was quenched with water (20 mL) and extracted with ethyl acetate (3 × 20 mL). The combined organic extracts were washed sequentially with water (2 × 20 mL) and brine (20 mL), dried over anhydrous Na_2SO_4, filtered, and concentrated under reduced pressure. The crude residue was purified by flash column chromatography (petroleum ether/ethyl acetate) to afford the corresponding product.

2.3 Optimization of reaction conditions for substrate 2a

2.3.1 Ligand Screen [a]

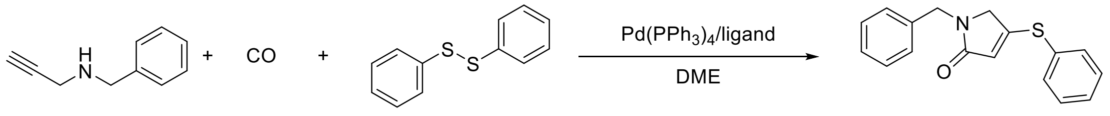

Entry	Ligand	Yield (%)
**1**	**ligand 1**	**70**
2	ligand 2	24
3	ligand 3	28
4	ligand 4	16
5	ligand 5	52
6	ligand 6	64
7	ligand 7	68
8	ligand 8	69
9	$BuPAd_2$	67

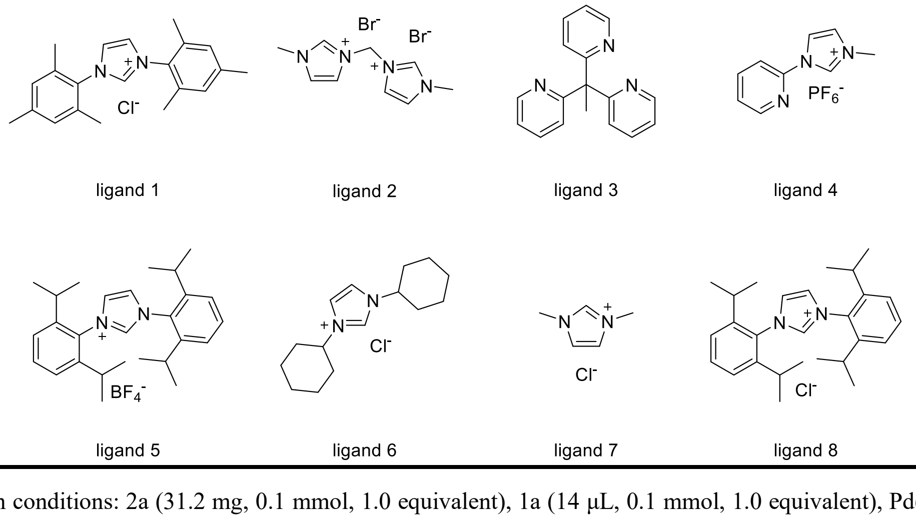

([a] Reaction conditions: 2a (31.2 mg, 0.1 mmol, 1.0 equivalent), 1a (14 µL, 0.1 mmol, 1.0 equivalent), $Pd(PPh_3)_4$ 2 mol%, ligand 10 mol%, Molecular sieve 50 mg, DME 1 mL, CO 5 bar, 90 °C, 24 h, GC yields.)

2.3.2 Catalyst Screen [a]

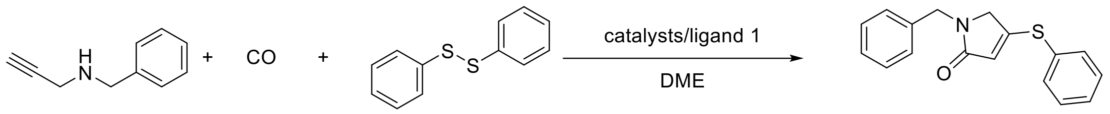

1a 2a 3a

Entry	Catalysts	Yield (%)
1	$PdCl_2(PPh_3)_2$	50
2	Pd_2dba_3	55
**3**	**$Pd(PPh_3)_4$**	**70**
4	$Pd(PPh_3)_4$, $[Rh(Cp^*)Cl_2]_2$	64
5	PEPPSI-IPr	59
6	$Pt(PPh_3)_4$	Trace
7	$P(tBu)_3PdG_3$	62
8	$Pd(TFA)_2$	23
9	$Co(acac)_2$	0
10	Pd-G4	26
11	K_2PdCl_4	25

([a] Reaction conditions: 2a (21.8 mg, 0.1 mmol, 1.0 equivalent), 1a (14 μL, 0.1 mmol, 1.0 equivalent), Catalysts 2 mol%, ligand 1 10 mol%, Molecular sieve 50 mg, DME 1 mL, CO 5 bar, 90 °C, 24 h, GC yields. PEPPSI-IPr: 1,3-Bis(2,6-Diisopropylphenyl)imidazol-2-ylidene-(3-chloropyridyl)palladium(II) dichloride, Pd-G4: t-BuXphos Palladacycle Gen. 4.)

2.3.3 Solvent Screen [a]

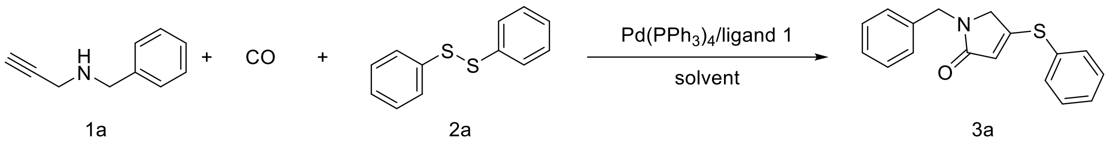

Entry	Solvent	Yield(%)
**1**	**DME**	**70**
2	toluene：DME 4:1	64
3	toluene：DME 1:4	68
4	toluene：DME 1:1	67
5	toluene：DMSO 4:1	66
6	toluene	64
7	DMSO	60

([a] Reaction conditions: 2a (21.8 mg, 0.1 mmol, 1.0 equivalent), 1a (14 μL, 0.1 mmol, 1.0 equivalent), $Pd(PPh_3)_4$ 2 mol%, ligand 1 10 mol%, Molecular sieve 50 mg, Solvent 1 mL, CO 5 bar, 90 °C, 24 h, GC yields.)

2.3.4 Stoichiometry and Concentration Screen [a]

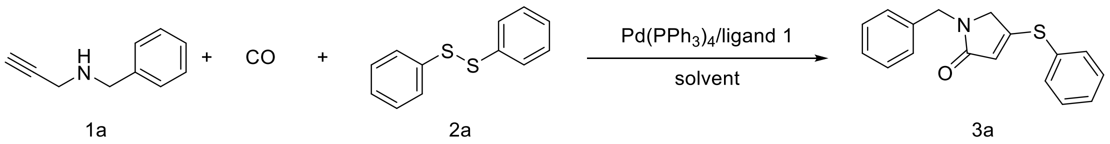

Entry	Variable	Yield (%)
1	$Pd(PPh_3)_4$/ligand 1 (0.1/0.2)	46
2	$Pd(PPh_3)_4$/ligand 1 (1/2)	54
3	$Pd(PPh_3)_4$/ligand 1 (2/5)	66
4	$Pd(PPh_3)_4$/ligand 1 (5/10)	62
5[b]	$Pd(PPh_3)_4$/ligand 1 (2/5)	67
6[b]	$Pd(PPh_3)_4$/ligand 1 (2/10)	70
7[c]	MS: 50 mg	70
8[c]	MS: 100 mg	75
9[c]	MS: 200 mg	66
10[d]	2a: 1.0eq	75

**11[d]**	**2a: 0.85eq**	**78**
12[d]	2a: 0.75eq	70

([a] Reaction conditions: 2a (21.8 mg, 0.1 mmol, 1.0 equivalent), 1a (14 μL, 0.1 mmol, 1.0 equivalent), $Pd(PPh_3)_4$ 0.1-5 mol%, ligand 1 0.2-10 mol%, Molecular sieve 50 mg, toluene 0.8 mL, DMSO 0.2 mL, CO 5 bar, 90 °C, 24 h, GC yields. [b]2a (21.8 mg, 0.1 mmol, 1.0 equivalent), 1a (14 μL, 0.1 mmol, 1.0 equivalent), $Pd(PPh_3)_4$ 2 mol%, ligand 1 5-10 mol%, Molecular sieve 50 mg, DME 1 mL, CO 5 bar, 90 °C, 24 h, GC yields. [c]2a (21.8 mg, 0.1 mmol, 1.0 equivalent), 1a (14 μL, 0.1 mmol, 1.0 equivalent), $Pd(PPh_3)_4$ 2 mol%, ligand 1 10 mol%, DME 1 mL, CO 5 bar, 90 °C, 24 h, GC yields. [d]1a (14 μL, 0.1 mmol, 1.0 equivalent), $Pd(PPh_3)_4$ 2 mol%, ligand 1 10 mol%, Molecular sieve 100mg. DME 1 mL, CO 5 bar, 90 °C, 24 h, GC yields. MS: molecular sieve.)

2.3.5 Control Experiments [a]

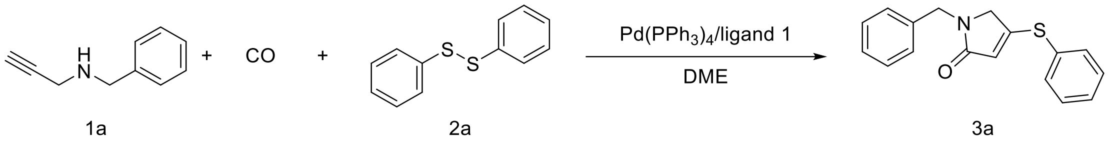

Entry	Variable	Yield (%)
1	Without $Pd(PPh_3)_4$	0
2	Without ligand 1	26
3	Without MS	40

([a] Reaction conditions: 2a (18.5 mg, 0.085 mmol, 0.85 equivalent), 1a (14 μL, 0.1 mmol, 1.0 equivalent), $Pd(PPh_3)_4$ 2 mol%, ligand 1 10 mol%, Molecular sieve 100 mg, DME 1 mL, CO 5 bar, 90 °C, 24 h, GC yields. MS: molecular sieve.)

2.4 Optimization of reaction conditions for substrate 2a'

2.4.1 Ligand Screen [a]

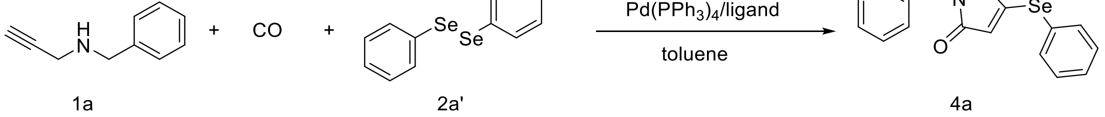

Entry	Ligand	Yield (%)
1	Xphos	12

2	PPh_3	22
3	Bupad	24
4	DPPPy	26
5	TPAP	33
6	TFPP	28
7	TFP	28
8	tBuxphos	11
9	$P(OPh_3)$	21
**10**	**ligand 1**	**36**
11	Nixantphos	16
12	1,10-phen	22
13	DPEphos	11
14	DPPE	0

([a] Reaction conditions: 2a (31.2 mg, 0.1 mmol, 1.0 equivalent), 1a (14 µL, 0.1 mmol, 1.0 equivalent), $Pd(PPh_3)_4$ 5 mol%, ligand 10 mol%, toluene 1 mL, CO 5 bar, 110 °C, 24 h, GC yields. DPPPy: 2-(diphenylphosphino)pyridine, TPAP: Tris(4-methoxyphenyl)phosphine, TFPP: Tris(4-fluorophenyl)phosphine, TFP: Tri(fur-2-yl)phosphine)

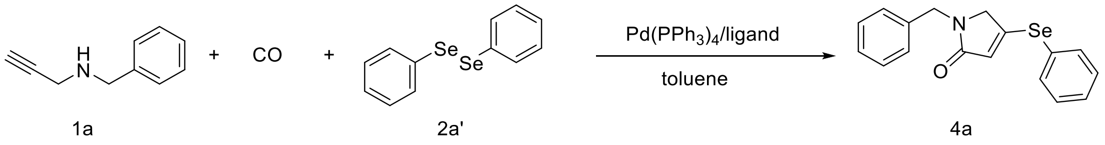

Entry	Ligand	Yield (%)
1	ligand 1	61
2	ligand 2	52
3	ligand 3	40

4	ligand 4	43
5	ligand 5	62
6	ligand 6	58
**7**	**ligand 7**	**68**
8	ligand 8	45

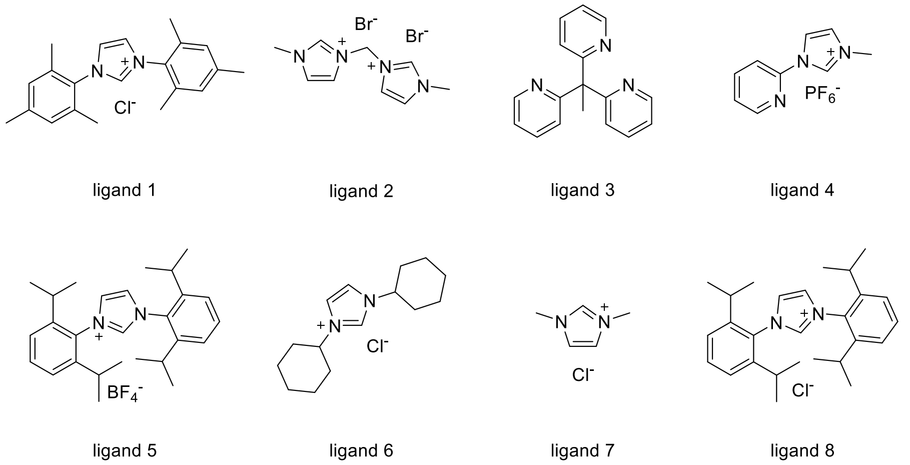

([a] Reaction conditions: 2a (31.2 mg, 0.1 mmol, 1.0 equivalent), 1a (14 μL, 0.1 mmol, 1.0 equivalent), $Pd(PPh_3)_4$ 5 mol%, ligand 10 mol%, Molecular sieve 50 mg, toluene 0.8 mL, DMSO 0.2 mL, CO 5 bar, 90 °C, 24 h, GC yields.)

2.4.2 Catalysts Screen [a]

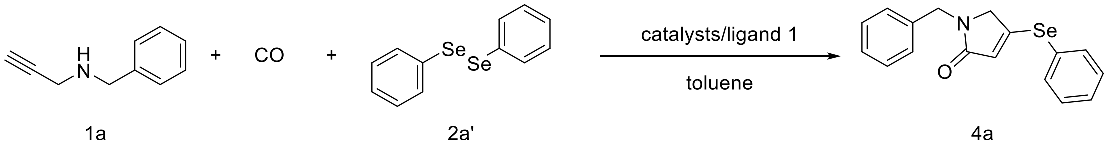

Entry	catalysts	Yield (%)
1	$NiBr_2$	10
2	$Mn(acac)_3$	Trace
3	Ferrocene	15
4	$Co(acac)_2$	14
5	$[Ir(dtbbpy)(ppy)_2][PF_6]$	0
6	$[Rh(Cp^*)Cl_2]_2$	0
7	CuCl	0
8	$Ru(phen)_3Cl_2$	0
9	$Pd(OAc)_2$	20

10	$PdCl_2$	Trace
**11**	**$Pd(PPh_3)_4$**	**36**
12	$PdCl_2(PPh_3)_2$	30
13	Pd_2dba_3	28
14	$(PhCN)_2PdCl_2$	Trace

([a] Reaction conditions: 2a (31.2 mg, 0.1 mmol, 1.0 equivalent), 1a (14 μL, 0.1 mmol, 1.0 equivalent), Catalysts 5 mol%, ligand 1 10 mol%, toluene 1 mL, CO 5 bar, 110 °C, 24 h, GC yields.)

2.4.3 Solvent Screen [a]

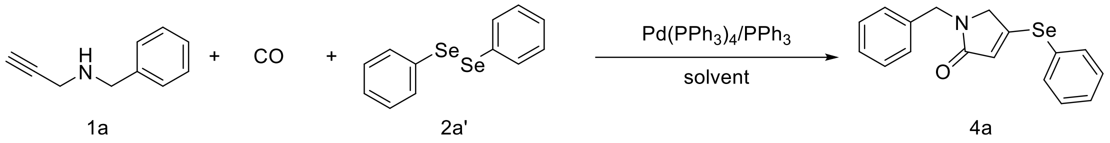

Entry	Solvent	Yield (%)
**1**	**toluene**	**25**
2	DMF	Trace
3	DCE	Trace
4	CH_3CN	20
5	1,4-Dioxane	Trace
6	THF	22
7	Ph-Cl	22
8	DMSO	24
9	Ph-CF_3	23

([a] Reaction conditions: 2a (31.2 mg, 0.1 mmol, 1.0 equivalent), 1a (14 μL, 0.1 mmol, 1.0 equivalent), $Pd(PPh_3)_4$ 5 mol%, PPh_3 10 mol%, Solvent 1 mL, CO 5 bar, 110 °C, 24 h, GC yields.)

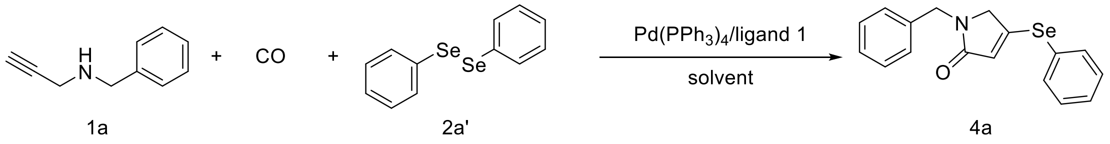

Entry	Solvent	Yield (%)
**1**	**toluene：DMSO 4：1**	**61**

2	toluene：DMSO 1：4	50
3	toluene：DMSO 1：1	52
4	DME	51
5	DMSO	50
6	toluene	52

([a] Reaction conditions: 2a (15.6 mg, 0.05 mmol, 0.5 equivalent), 1a (14 µL, 0.1 mmol, 1.0 equivalent), $Pd(PPh_3)_4$ 5 mol%, ligand 1 10 mol%, Molecular sieve 50 mg, Solvent 1 mL, CO 5 bar, 90 °C, 24 h, GC yields.)

2.4.4 Temperature Screen [a]

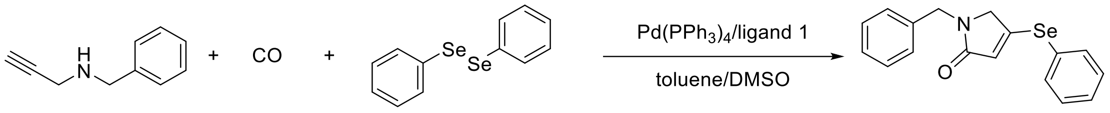

Entry	Temperature (℃)	Yield (%)
1	80	48
**2**	**90**	**61**
3	110	36

([a] Reaction conditions: 2a (15.6 mg, 0.05 mmol, 0.5 equivalent), 1a (14 µL, 0.1 mmol, 1.0 equivalent), $Pd(PPh_3)_4$ 5 mol%, ligand 1 10 mol%, Molecular sieve 50 mg, toluene 0.8 mL, DMSO 0.2 mL, CO 5 bar, 24 h, GC yields.)

2.4.5 Stoichiometry and Concentration Screen [a]

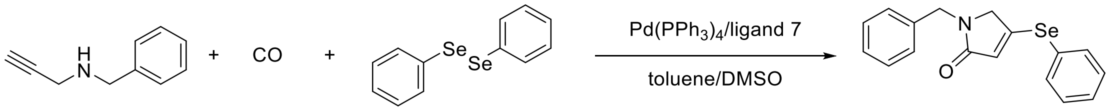

Entry	Variable	Yield (%)
1	$Pd(PPh_3)_4$/ligand 7 (5/10), 1.0 eq, 50mg	68
2	$Pd(PPh_3)_4$/ligand 7 (5/10), 1.0 eq, 100mg	70
3	$Pd(PPh_3)_4$/ligand 7 (5/10), 1.0 eq, 200mg	60
4	$Pd(PPh_3)_4$/ligand 7 (2/10), 1.0 eq, 100mg	49
6	$Pd(PPh_3)_4$/ligand 7 (5/20), 1.0 eq, 100mg	71

**7**	**$Pd(PPh_3)_4$/ligand 7 (5/20), 0.85 eq, 100mg**	**74**
8	$Pd(PPh_3)_4$/ligand 7 (5/20), 0.5 eq, 100mg	62

([a] Reaction conditions: 2a (31.2 mg, 0.1 mmol, 1.0 equivalent), 1a (14 μL, 0.1 mmol, 1.0 equivalent), $Pd(PPh_3)_4$ 2-5 mol%, ligand 7 10-20 mol%, Molecular sieve 50-200 mg, toluene 0.8 mL, DMSO 0.2 mL, CO 5 bar, 90 °C, 24 h, GC yields.)

2.4.6 Additive Screen [a]

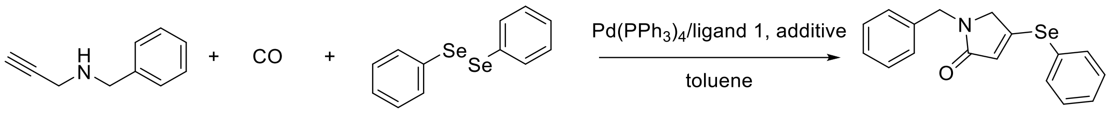

Entry	Additive	Yield (%)
1	KI	35
2	TBAI	30
3	NH_4I	24
4	NaI	41
5	TBAB	35
6	TEAB	34
7	LiBr	32
8	K_3PO_4	Trace
9	Cs_2CO_3	37
10	t-BuOK	39
11	$AgSbF_6$	Trace
12	$AgCF_3SO_3$	Trace
**13**	**MS**	**52**
14[b]	DTBP	48
15[b]	NaI	40
16[b]	KI	45
17[b]	TBAB	44

([a] Reaction conditions: 2a (15.6 mg, 0.05 mmol, 0.5 equivalent), 1a (14 μL, 0.1 mmol, 1.0 equivalent), $Pd(PPh_3)_4$ 5 mol%, ligand 1 10 mol%, Additive (1.0 equivalent), toluene 1 mL, CO 5 bar, 90 °C, 24 h, GC yields. [b] Reaction

conditions: 2a (15.6 mg, 0.05 mmol, 0.5 equivalent), 1a (14 µL, 0.1 mmol, 1.0 equivalent), $Pd(PPh_3)_4$ 5 mol%, ligand 1 10 mol%, Molecular sieve 50 mg, Additive (1.0 equivalent), toluene 1 mL, CO 5 bar, 90 °C, 24 h, GC yields.)

2.4.7 Control Experiments [a]

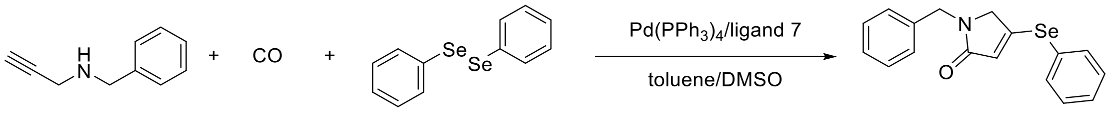

Entry	Variable	Yield (%)
1	Without $Pd(PPh_3)_4$	0
12	Without ligand 7	12
3	Without MS	39

([a] Reaction conditions: 2a (26.5 mg, 0.85 mmol, 1.0 equivalent), 1a (14 µL, 0.1 mmol, 1.0 equivalent), $Pd(PPh_3)_4$ 5 mol%, ligand 7 20 mol%, Molecular sieve 50 mg, toluene 0.8 mL, DMSO 0.2 mL, CO 5 bar, 90 °C, 24 h, GC yields. MS: molecular sieve.)

2.5 General procedures for the synthesis of 3a-4s:

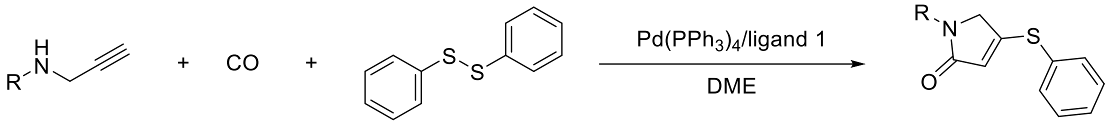

A flame-dried borosilicate glass reaction tube (5 mL) was charged with 1,2-diphenyldisulfane (**2a-2f**, 0.85 equivalent, 0.085 mmol), $Pd(PPh_3)_4$ (2 mol%, 2.3 mg), 1,3-bis(2,4,6-trimethylphenyl)imidazol-2-ylidene (ligand 1 10 mol%, 3.4 mg), and activated molecular sieves (MS, 100 mg). The tube was sealed with a PTFE-lined silicone septum, and the headspace was evacuated and refilled with nitrogen (three cycles). A solution of the corresponding alkynyl amine (**1a–1s**, 0.1 mmol, 1.0 equivalent) in 1,2-dimethoxyethane (DME, 1 mL) was then injected via syringe through the septum. The sealed tube was placed in an aluminum heating block, and a vent needle was inserted through the septum. The assembly was then enclosed in a stainless steel high-pressure reaction vessel. The autoclave was purged with nitrogen (three cycles), pressurized with carbon monoxide to 5 bar, and heated to 90 °C with stirring for 24 h. After cooling to room temperature, the reaction mixture was diluted with ethyl acetate and concentrated under reduced pressure. The crude residue was purified by flash column chromatography on silica gel (eluent: petroleum ether/ethyl acetate) to afford the desired products **3a–3x**.

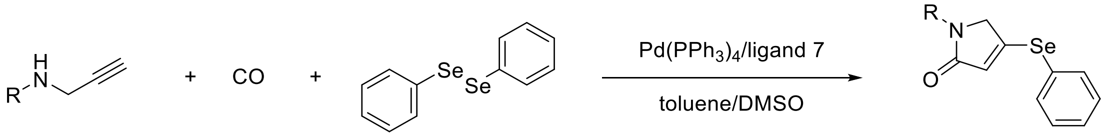

A flame-dried borosilicate glass reaction tube (5 mL) was charged with 1,2-diphenyldiseulfane (**2a'**, 0.85 equivalent, 0.085 mmol, 26.5 mg), $Pd(PPh_3)_4$ (5 mol%, 5.8 mg), 1,3-dimethylimidazolium chloride (ligand 7 20 mol%, 2.6 mg), and activated molecular sieves (MS, 100 mg). The tube was sealed with a PTFE-lined silicone septum, and the headspace was evacuated and refilled with nitrogen (three cycles). A solution of the corresponding alkynyl amine (**1a–1s**, 0.1 mmol, 1.0 equivalent) in toluene and DMSO (0.8 + 0.2 mL) was then injected via syringe through the septum. The sealed tube was placed in an aluminum heating block, and a vent needle was inserted through the septum. The assembly was then enclosed in a stainless steel high-pressure reaction vessel. The autoclave was purged with nitrogen (three cycles), pressurized with carbon monoxide to 5 bar, and heated to 90 °C with stirring for 24 h. After cooling to room temperature, the reaction mixture was diluted with ethyl acetate and concentrated under

reduced pressure. The crude residue was purified by flash column chromatography on silica gel (eluent: petroleum ether/ethyl acetate) to afford the desired products **4a–4s**.

2.6 Gas chromatography-mass spectrometry results.

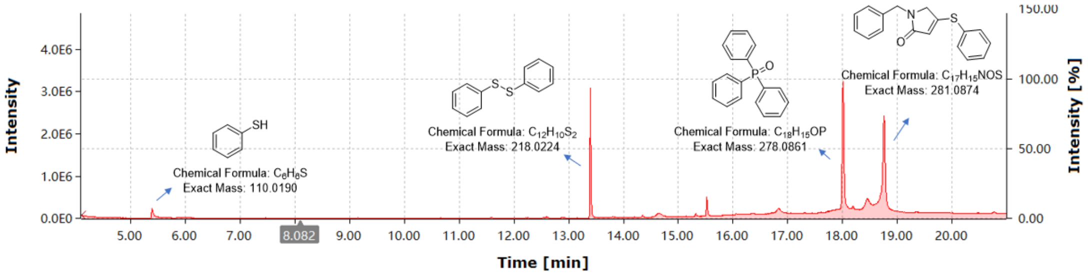

Scan: 243 | RT: 5.348 | RI: 0 | Detector: MS1 | Type: Centroid | Signal: 17780

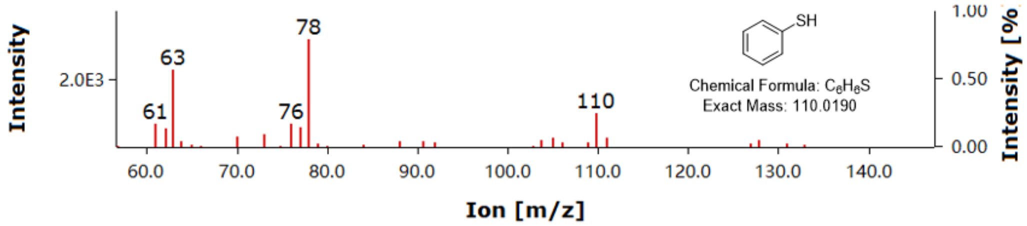

Scan: 1791 | RT: 13.38 | RI: 0 | Detector: MS1 | Type: Centroid | Signal: 406029

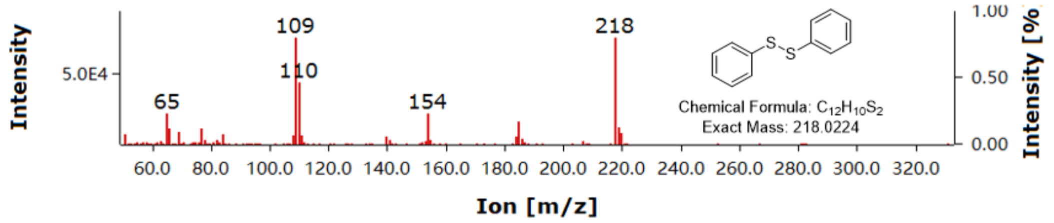

Scan: 2680 | RT: 17.993 | RI: 0 | Detector: MS1 | Type: Centroid | Signal: 1619525

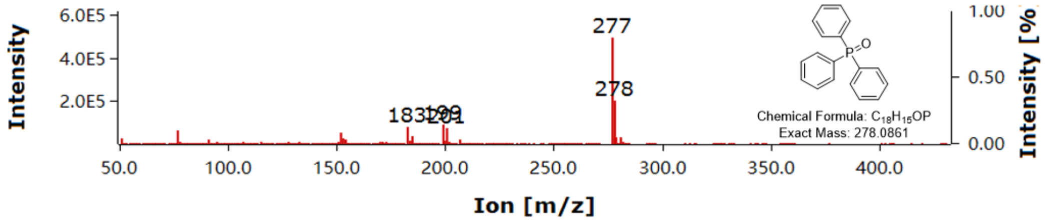

Scan: 2827 | RT: 18.756 | RI: 0 | Detector: MS1 | Type: Centroid | Signal: 2433408

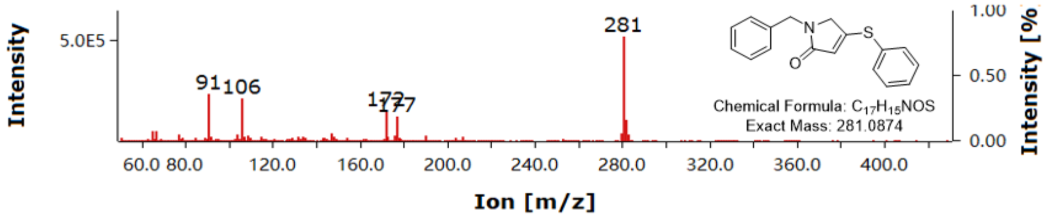

2.7 Alkyne amine substrates of 1a-1s:

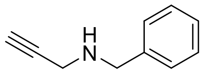
1a

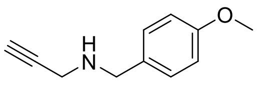
1b

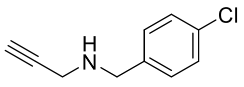
1c

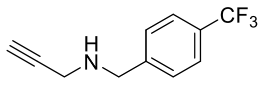
1d

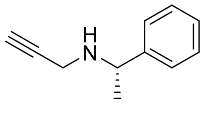
1e

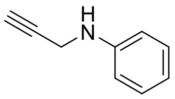
1f

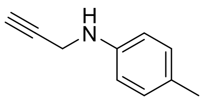
1g

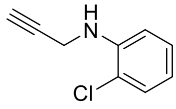
1h

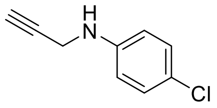
1i

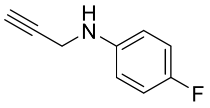
1j

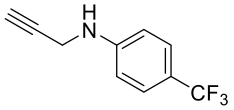
1k

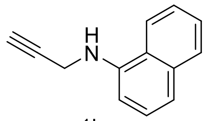
1l

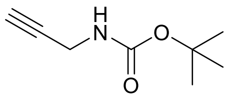
1m

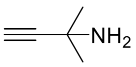
1n

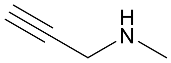
1o

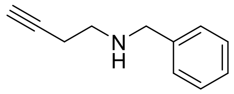
1p

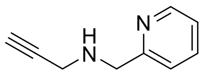
1q

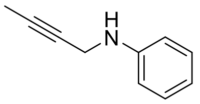
1r

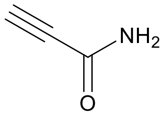
1s

2.8 Substrate of 2a-2f,2a’

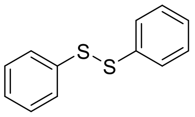
2a

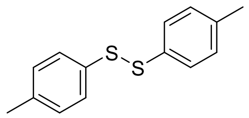
2b

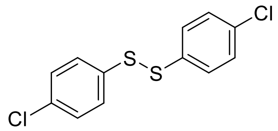
2c

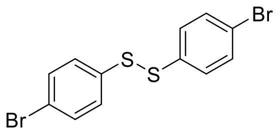
2d

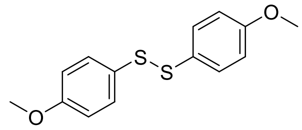
2e

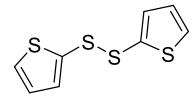
2f

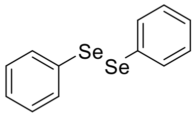
2a'

3. Characterization Data of Products

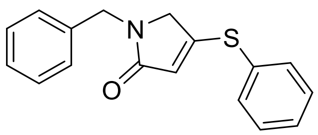

1-benzyl-4-(phenylthio)-1,5-dihydro-2H-pyrrol-2-one (3a): (51.2 mg, yellow solid, m.p. = 76-78 °C, yield: 78%)

^{1}H NMR (400 MHz, $CDCl_3$) δ 7.51 (dd, *J* = 7.4, 2.1 Hz, 2H), 7.45 – 7.39 (m, 3H), 7.35 – 7.27 (m, 3H), 7.22 (d, *J* = 6.6 Hz, 2H), 5.59 (s, 1H), 4.56 (s, 2H), 3.84 (s, 2H).

^{13}C NMR (101 MHz, $CDCl_3$) δ 170.4, 157.1, 137.1, 134.5, 129.8, 129.7, 128.7, 128.5, 127.8, 127.5, 118.6, 53.4, 45.8.

HRMS (ESI-TOF) Calc. for $C_{17}H_{15}NOS$ $[M+H]^+$: 282.0947; found: 282.0950.

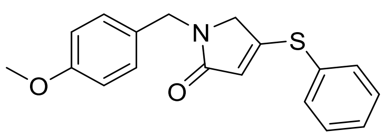

1-(4-methoxybenzyl)-4-(phenylthio)-1,5-dihydro-2H-pyrrol-2-one(3b): (40.4 mg, white solid, m.p. = 106-108 °C, yield: 65%)

^{1}H NMR (500 MHz, $CDCl_3$) δ 7.54 – 7.45 (m, 2H), 7.39 (d, *J* = 7.0 Hz, 3H), 7.14 (d, *J* = 8.5 Hz, 2H), 6.84 (d, *J* = 8.5 Hz, 2H), 5.57 (s, 1H), 4.48 (s, 2H), 3.81 (s, 2H), 3.77 (s, 3H).

^{13}C NMR (75 MHz, $CDCl_3$) δ 170.3, 159.0, 157.0, 134.5, 129.8, 129.8, 129.2, 129.2, 128.6, 118.7, 114.0, 55.2, 53.3, 45.3.

HRMS (ESI-TOF) Calc. for $C_{18}H_{17}NO_2S$ $[M+H]^+$: 312.1053; found: 312.1054.

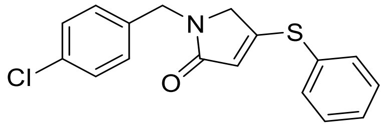

1-(4-chlorobenzyl)-4-(phenylthio)-1,5-dihydro-2H-pyrrol-2-one(3c): (51.2 mg, white solid, m.p. = 86-88 °C, yield: 67%)

^{1}H NMR (500 MHz, $CDCl_3$) δ 7.51 (dd, *J* = 7.7, 1.8 Hz, 2H), 7.47 – 7.37 (m, 3H), 7.28 (d, *J* = 7.9 Hz, 2H), 7.14 (d, *J* = 8.4 Hz, 2H), 5.58 (s, 1H), 4.51 (s, 2H), 3.82 (s, 2H).

^{13}C NMR (75 MHz, $CDCl_3$) δ 170.4, 157.3, 135.7, 134.5, 133.4, 129.9, 129.8, 129.2, 128.9, 128.4, 118.5, 53.4, 45.2.

HRMS (ESI-TOF) Calc. for $C_{17}H_{14}ClNOS$ $[M+H]^+$: 316.0557; found: 316.0561.

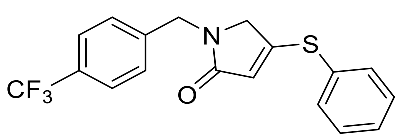

4-(phenylthio)-1-(4-(trifluoromethyl)benzyl)-1,5-dihydro-2H-pyrrol-2-one(3d): (45.4 mg, white solid, m.p. = 115-117 °C, yield: 65%)

^{1}H NMR (500 MHz, $CDCl_3$) δ 7.58 (d, *J* = 8.2 Hz, 2H), 7.52 (dd, *J* = 7.6, 1.7 Hz, 2H), 7.46 – 7.39 (m, 3H), 7.33 (d, *J* = 7.8 Hz, 2H), 5.61 (s, 1H), 4.61 (s, 2H), 3.85 (s, 2H).
^{13}C NMR (75 MHz, $CDCl_3$) δ 170.5, 157.6, 141.3, 134.6, 130.0, 129.9, 128.4, 128.1, 125.7 (q, $J_{C\text{-}F}$ = 3.8 Hz), 124.7 (q, $J_{C\text{-}F}$ = 22.5 Hz), 123.7, 122.2, 118.4, 53.5, 45.5.
^{19}F NMR (565 MHz, $CDCl_3$) δ -62.6.
HRMS (ESI-TOF) Calc. for C18H14F3NOS $[M+H]^+$: 350.0821; found: 350.0821.

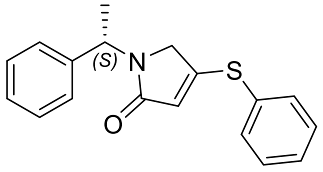

(S)-1-(1-phenylethyl)-4-(phenylthio)-1,5-dihydro-2H-pyrrol-2-one(3e): (36.1 mg, yellow solid, m.p. = 79-81 °C, yield: 61%)

^{1}H NMR (500 MHz, $CDCl_3$) δ 7.50 (dd, *J* = 7.6, 2.0 Hz, 2H), 7.45 – 7.37 (m, 3H), 7.36 – 7.31 (m, 2H), 7.28 (d, *J* = 7.6 Hz, 3H), 5.51 (d, *J* = 4.0 Hz, 2H), 3.92 (d, *J* = 18.4 Hz, 1H), 3.62 (d, *J* = 18.4 Hz, 1H), 1.56 (d, *J* = 7.2 Hz, 3H).
^{13}C NMR (75 MHz, $CDCl_3$) δ 169.7, 157.1, 140.8, 134.5, 129.9, 129.8, 128.6, 127.5, 126.9, 118.8, 105.4, 49.9, 48.9, 17.5.
HRMS (ESI-TOF) Calc. for $C_{18}H_{17}NOS$ $[M+H]^+$: 296.1104; found: 296.1105.

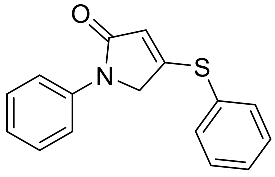

1-phenyl-4-(phenylthio)-1,5-dihydro-2H-pyrrol-2-one(3f): (29.4 mg, white solid, m.p. = 114-116 °C, yield: 55%)

^{1}H NMR (500 MHz, $CDCl_3$) δ 7.59 (td, *J* = 5.7, 3.1 Hz, 4H), 7.45 (d, *J* = 7.1 Hz, 3H), 7.33 (t, *J* = 8.0 Hz, 2H), 7.09 (t, *J* = 7.4 Hz, 1H), 5.67 (s, 1H), 4.38 (s, 2H).
^{13}C NMR (126 MHz, $CDCl_3$) δ 169.0, 156.8, 139.0, 134.7, 130.1, 129.9, 129.0, 128.3, 123.8, 120.0, 118.5, 54.2.
HRMS (ESI-TOF) Calc. for $C_{16}H_{13}NOS$ $[M+H]^+$: 268.0791; found: 268.0792.

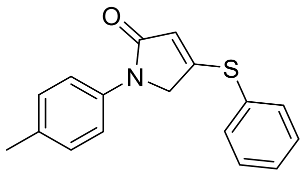

4-(phenylthio)-1-(p-tolyl)-1,5-dihydro-2H-pyrrol-2-one(3g): (35.4 mg, white solid, m.p. = 121-123 °C, yield: 63%)

^{1}H NMR (500 MHz, $CDCl_3$) δ 7.58 (dd, *J* = 7.4, 2.0 Hz, 2H), 7.50 – 7.40 (m, 5H), 7.13 (d, *J* = 8.5 Hz, 2H), 5.66 (s, 1H), 4.35 (s, 2H), 2.30 (s, 3H).

^{13}C NMR (126 MHz, $CDCl_3$) δ 168.9, 156.4, 136.4, 134.6, 133.5, 130.0, 129.9, 129.5, 128.3, 120.0, 118.7, 54.3, 20.7.

HRMS (ESI-TOF) Calc. for $C_{17}H_{15}NOS$ $[M+H]^+$: 282.0947; found: 282.0949.

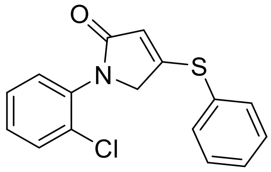

1-(2-chlorophenyl)-4-(phenylthio)-1,5-dihydro-2H-pyrrol-2-one(3h): (38 mg, yellow liquid, yield: 63%; 95% purity)

^{1}H NMR (500 MHz, $CDCl_3$) δ 7.60 (dd, *J* = 7.0, 2.5 Hz, 2H), 7.44 (dd, *J* = 5.0, 2.0 Hz, 4H), 7.34 – 7.23 (m, 4H), 5.72 (s, 1H), 4.36 (s, 2H).

^{13}C NMR (75 MHz, $CDCl_3$) δ 158.4, 135.0, 134.5, 132.2, 130.2, 129.9, 129.7, 129.6, 128.8, 128.5, 128.0, 127.4, 118.1, 55.4.

HRMS (ESI-TOF) Calc. for $C_{16}H_{12}ClNOS$ $[M+H]^+$: 302.0409; found: 302.0407.

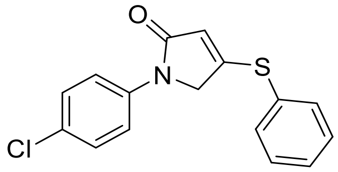

1-(4-chlorophenyl)-4-(phenylthio)-1,5-dihydro-2H-pyrrol-2-one(3i): (28.9 mg, white solid, m.p. = 148-150 °C, yield: 48%)

^{1}H NMR (500 MHz, $CDCl_3$) δ 7.58 (dd, *J* = 7.6, 2.0 Hz, 2H), 7.57 – 7.53 (m, 2H), 7.52 – 7.43 (m, 3H), 7.31 – 7.26 (m, 2H), 5.64 (s, 1H), 4.35 (d, *J* = 1.3 Hz, 2H).

^{13}C NMR (126 MHz, $CDCl_3$) δ 168.9, 157.2, 137.6, 134.7, 130.2, 130.0, 129.0, 128.8, 128.1, 119.7, 119.4, 54.1.

HRMS (ESI-TOF) Calc. for $C_{16}H_{12}ClNOS$ $[M+H]^+$: 302.0401; found: 302.0402.

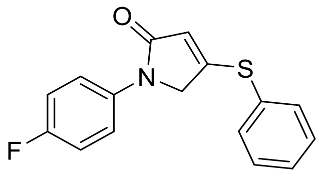

1-(4-fluorophenyl)-4-(phenylthio)-1,5-dihydro-2H-pyrrol-2-one(3j): (34.2 mg, white solid, m.p. = 132-134 °C, yield: 60%)

^{1}H NMR (500 MHz, $CDCl_3$) δ 7.58 (dd, *J* = 7.5, 2.0 Hz, 2H), 7.56 – 7.51 (m, 2H), 7.50 – 7.39 (m, 3H), 7.11 – 6.95 (m, 2H), 5.65 (s, 1H), 4.35 (d, *J* = 1.2 Hz, 2H).
^{13}C NMR (126 MHz, $CDCl_3$) δ 168.9, 159.1 (d, *J* = 252 Hz), 156.9, 135.1 (d, *J* = 2.5 Hz), 134.7, 130.2, 129.9, 128.2, 120.3 (d, *J* = 7.6 Hz), 119.7, 115.7 (d, *J* = 25.2 Hz), 54.4.
^{19}F NMR (471 MHz, $CDCl_3$) δ -118.6.
HRMS (ESI-TOF) Calc. for $C_{16}H_{12}FNOS$ $[M+H]^+$: 286.0696; found: 286.0700.

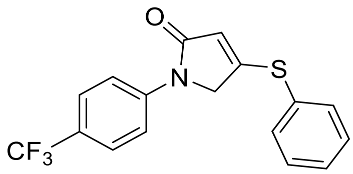

4-(phenylthio)-1-(4-(trifluoromethyl)phenyl)-1,5-dihydro-2H-pyrrol-2-one(3k): (30.1 mg, white solid, m.p. = 168-170 °C, yield: 45%)

^{1}H NMR (500 MHz, $CDCl_3$) δ 7.74 (d, *J* = 8.9 Hz, 2H), 7.64 – 7.54 (m, 4H), 7.48 (t, *J* = 7.2 Hz, 3H), 5.65 (s, 1H), 4.42 (s, 2H).
^{13}C NMR (126 MHz, $CDCl_3$) δ 169.2, 158.0, 142.0, 134.8, 130.4, 130.1, 127.9, 126.3 (q, *J* = 3.8 Hz), 125.3 (q, *J* = 32.7 Hz), 124.16 (q, *J* = 272.2 Hz), 119.6, 117.5, 53.9.
^{19}F NMR (471 MHz, $CDCl_3$) δ -62.1.
HRMS (ESI-TOF) Calc. for $C_{17}H_{12}F_3NOS$ $[M+H]^+$: 336.0665; found: 336.0668.

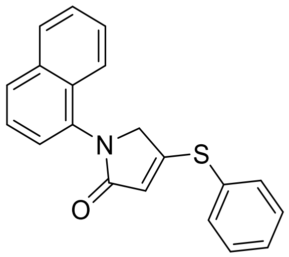

1-(naphthalen-1-yl)-4-(phenylthio)-1,5-dihydro-2H-pyrrol-2-one(3l): (31.7 mg, yellow liquid, yield: 50%; 95% purity)

^{1}H NMR (300 MHz, $CDCl_3$) δ 7.94 – 7.81 (m, 2H), 7.75 – 7.68 (m, 1H), 7.64 (dd, *J* = 6.5, 3.1 Hz, 2H), 7.52 (dd, *J* = 6.5, 2.4 Hz, 2H), 7.49 – 7.43 (m, 4H), 7.38 (d, *J* = 7.3 Hz, 1H), 5.83 (s, 1H), 4.43 (s, 2H).
^{13}C NMR (75 MHz, $CDCl_3$) δ 170.5, 158.3, 134.8, 134.5, 134.4, 130.5, 130.1, 129.9, 128.6, 128.5, 128.3, 126.9, 126.4, 125.5, 125.5, 122.9, 118.7, 57.7.
HRMS (ESI-TOF) Calc. for $C_{20}H_{15}NOS$ $[M+H]^+$: 318.0947; found: 318.0950.

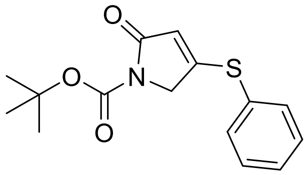

tert-butyl 2-oxo-4-(phenylthio)-2,5-dihydro-1H-pyrrole-1-carboxylate(3m): (39.1 mg, yellow liquid, yield: 67%)

^{1}H NMR (500 MHz, CDCl$_3$) δ 7.53 (dd, *J* = 7.7, 1.7 Hz, 2H), 7.45 (t, *J* = 7.7 Hz, 3H), 5.47 (s, 1H), 4.29 (s, 2H), 1.51 (s, 9H).

^{13}C NMR (126 MHz, CDCl$_3$) δ 167.4, 161.0, 149.2, 134.7, 130.4, 130.0, 127.5, 117.8, 82.8, 52.5, 28.0.

HRMS (ESI-TOF) Calc. for $C_{15}H_{17}NO_3S$ [M+Na]$^+$: 314.0821; found: 314.0819.

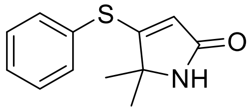

5,5-dimethyl-4-(phenylthio)-1,5-dihydro-2H-pyrrol-2-one(3n): (33.8 mg, white solid, m.p. = 146-148 °C, yield: 77%)

^{1}H NMR (500 MHz, CDCl$_3$) δ 7.54 (dd, *J* = 6.3, 3.3 Hz, 2H), 7.43 (dd, *J* = 5.1, 1.9 Hz, 3H), 6.75 (s, 1H), 5.16 (s, 1H), 1.47 (s, 6H).

^{13}C NMR (126 MHz, CDCl$_3$) δ 171.1, 134.8, 131.9, 129.9, 129.8, 128.8, 115.5, 62.6, 27.2.

HRMS (ESI-TOF) Calc. for $C_{12}H_{13}NOS$ [M+H]$^+$: 220.0791; found: 220.0791.

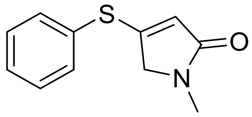

1-methyl-4-(phenylthio)-1,5-dihydro-2H-pyrrol-2-one(3o): (32.0 mg, yellow liquid, yield: 78%; 95% purity)

^{1}H NMR (300 MHz, CDCl$_3$) δ 7.54 – 7.48 (m, 2H), 7.40 (dd, *J* = 5.2, 1.9 Hz, 3H), 5.58 (s, 1H), 3.88 (s, 2H), 2.92 (s, 3H).

^{13}C NMR (75 MHz, CDCl$_3$) δ 170.7, 156.2, 134.5, 129.8, 129.7, 128.6, 119.1, 55.9, 28.9.

HRMS (ESI-TOF) Calc. for $C_{11}H_{11}NOS$ [M+H]$^+$: 206.0634; found: 206.0634.

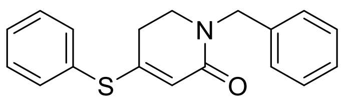

1-benzyl-4-(phenylthio)-5,6-dihydropyridin-2(1H)-one(3p): (47.2 mg, yellow liquid, yield: 80%)

^{1}H NMR (500 MHz, CDCl$_3$) δ 7.54 – 7.47 (m, 2H), 7.42 (t, *J* = 4.5 Hz, 3H), 7.30 (d, *J* = 7.1 Hz, 2H), 7.27 – 7.24 (m, 3H), 5.41 (s, 1H), 4.59 (s, 2H), 3.34 (t, *J* = 6.9 Hz, 2H), 2.47 (t, *J* = 6.9 Hz, 2H).

^{13}C NMR (126 MHz, CDCl$_3$) δ 163.9, 153.1, 137.4, 135.3, 129.9, 129.8, 128.6, 128.4, 128.0, 127.3, 115.5, 49.4, 44.6, 29.0.

HRMS (ESI-TOF) Calc. for $C_{18}H_{17}NOS$ [M+H]$^+$: 296.1104; found: 296.1106.

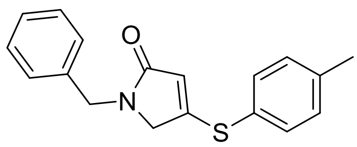

1-benzyl-4-(p-tolylthio)-1,5-dihydro-2H-pyrrol-2-one(3t): (44.9 mg, white solid, m.p. = 99-101 °C, yield: 76%)

1H NMR (600 MHz, $CDCl_3$) δ 7.43 (d, J = 8.0 Hz, 2H), 7.36 (t, J = 7.4 Hz, 2H), 7.30 (t, J = 7.3 Hz, 1H), 7.25 (t, J = 7.2 Hz, 4H), 5.62 (s, 1H), 4.59 (s, 2H), 3.86 (s, 2H), 2.41 (s, 3H).

13C NMR (151 MHz, $CDCl_3$) δ 170.5, 157.7, 140.2, 137.1, 134.5, 130.5, 128.7, 127.8, 127.5, 124.9, 118.3, 53.4, 45.8, 21.2.

HRMS (ESI-TOF) Calc. for $C_{18}H_{17}NOS$ $[M+H]^+$: 296.1104; found: 296.1106.

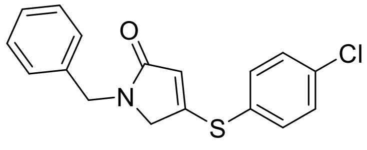

1-benzyl-4-((4-chlorophenyl)thio)-1,5-dihydro-2H-pyrrol-2-one(3u): (35.3 mg, white solid, m.p. = 80-82 °C, yield: 56%)

1H NMR (600 MHz, $CDCl_3$) δ 7.48 (d, J = 8.3 Hz, 2H), 7.42 (d, J = 8.3 Hz, 2H), 7.36 (t, J = 7.3 Hz, 2H), 7.31 (t, J = 7.0 Hz, 1H), 7.25 (d, J = 7.3 Hz, 2H), 5.65 (s, 1H), 4.59 (s, 2H), 3.87 (s, 2H).

13C NMR (151 MHz, $CDCl_3$) δ 170.1, 156.2, 137.0, 136.3, 135.7, 130.0, 128.7, 127.9, 127.5, 127.0, 119.1, 53.3, 45.9.

HRMS (ESI-TOF) Calc. for C17H14ClNOS $[M+H]^+$: 315.0485; found: 315.0481.

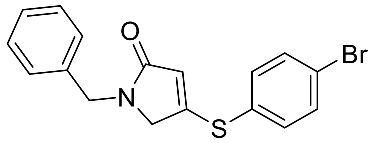

1-benzyl-4-((4-bromophenyl)thio)-1,5-dihydro-2H-pyrrol-2-one(3v): (45.3 mg, white solid, m.p. = 102-104 °C, yield: 63%)

1H NMR (600 MHz, $CDCl_3$) δ 7.57 (d, J = 7.7 Hz, 2H), 7.41 (d, J = 7.7 Hz, 2H), 7.38 – 7.33 (m, 2H), 7.32 (d, J = 6.7 Hz, 1H), 7.25 (d, J = 6.8 Hz, 2H), 5.66 (s, 1H), 4.60 (s, 2H), 3.87 (s, 2H).

13C NMR (151 MHz, $CDCl_3$) δ 170.1, 156.0, 137.0, 135.9, 133.0, 128.7, 127.9, 127.7, 127.6, 124.5, 119.2, 53.4, 45.9.

HRMS (ESI-TOF) Calc. for C17H14BrNOS $[M+H]^+$: 358.9979; found: 358.9978.

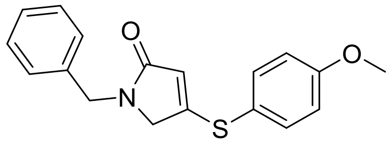

1-benzyl-4-((4-methoxyphenyl)thio)-1,5-dihydro-2H-pyrrol-2-one(3w): (50.4 mg, white solid, m.p. = 90-92 °C, yield: 81%)

1H NMR (600 MHz, $CDCl_3$) δ 7.46 (d, J = 8.8 Hz, 2H), 7.36 (t, J = 7.4 Hz, 2H), 7.32 – 7.28 (m, 1H), 7.25 (d, J = 7.3 Hz, 2H), 6.95 (d, J = 8.7 Hz, 2H), 5.58 (s, 1H), 4.59 (s, 2H), 3.84 (s, 5H).

13C NMR (151 MHz, $CDCl_3$) δ 170.5, 161.0, 158.4, 137.2, 136.3, 128.7, 127.8, 127.5, 118.9, 118.0, 115.3, 55.4, 53.3, 45.8.

HRMS (ESI-TOF) Calc. for $C_{18}H_{17}NO_2S$ $[M+H]^+$: 311.0980; found: 311.0980.

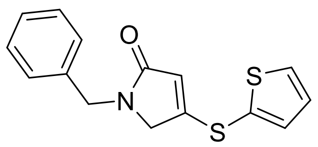

1-benzyl-4-(thiophen-2-ylthio)-1,5-dihydro-2H-pyrrol-2-one(3x): (34.5 mg, white solid, m.p. = 82-84 °C, yield: 60%)

1H NMR (600 MHz, $CDCl_3$) δ 7.58 (d, J = 5.3 Hz, 1H), 7.36 (t, J = 7.3 Hz, 2H), 7.31 (t, J = 5.6 Hz, 2H), 7.25 (d, J = 7.3 Hz, 2H), 7.15 – 7.09 (m, 1H), 5.77 (s, 1H), 4.60 (s, 2H), 3.86 (s, 2H).

13C NMR (151 MHz, $CDCl_3$) δ 170.1, 157.1, 137.0, 136.9, 132.5, 128.7, 128.1, 127.8, 127.5, 125.2, 119.2, 53.0, 45.8.

HRMS (ESI-TOF) Calc. for $C_{15}H_{13}NOS_2$ $[M+H]^+$: 287.0439; found: 287.0433.

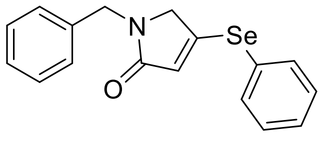

1-benzyl-4-(phenylselanyl)-1,5-dihydro-2H-pyrrol-2-one(4a): (48.7 mg, yellow solid, m.p. = 73-75 °C, yield: 74%)

^{1}H NMR (400 MHz, $CDCl_3$) δ 7.6 (d, *J* = 7.2 Hz, 2H), 7.4 – 7.2 (m, 6H), 7.2 (d, *J* = 7.2 Hz, 2H), 5.8 (s, 1H), 4.5 (s, 2H), 3.8 (s, 2H).

^{13}C NMR (151 MHz, $CDCl_3$) δ 170.3, 152.1, 137.1, 135.8, 129.8, 129.5, 128.7, 127.8, 127.5, 124.9, 123.8, 55.2, 45.9.

HRMS (ESI-TOF) Calc. for $C_{17}H_{15}NOSe$ $[M-H]^+$: 328.0247; found: 328.0241.

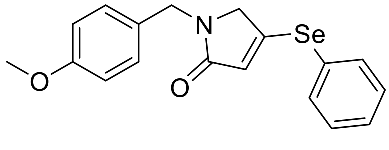

1-(4-methoxybenzyl)-4-(phenylselanyl)-1,5-dihydro-2H-pyrrol-2-one(4b): (46.0 mg, white solid, m.p. = 88-90 °C, yield: 64%)

^{1}H NMR (500 MHz, $CDCl_3$) δ 7.59 (d, *J* = 7.1 Hz, 2H), 7.38 (dt, *J* = 27.9, 7.2 Hz, 3H), 7.13 (d, *J* = 8.6 Hz, 2H), 6.84 (d, *J* = 8.6 Hz, 2H), 5.84 (s, 1H), 4.49 (s, 2H), 3.81 (s, 2H), 3.78 (s, 3H).

^{13}C NMR (75 MHz, $CDCl_3$) δ 170.2, 159.0, 152.0, 135.9, 129.8, 129.5, 129.2, 129.2, 125.0, 123.9, 114.1, 55.2, 55.1, 45.3.

HRMS (ESI-TOF) Calc. for $C_{18}H_{17}NO_2Se$ $[M+H]^+$: 360.0498; found: 360.0497.

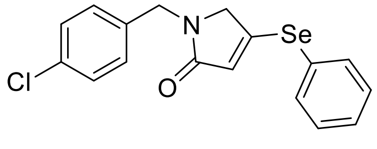

1-(4-chlorobenzyl)-4-(phenylselanyl)-1,5-dihydro-2H-pyrrol-2-one(4c): (45.1 mg, white solid, m.p. = 79-81 °C, yield: 62%)

^{1}H NMR (500 MHz, $CDCl_3$) δ 7.59 (d, *J* = 7.0 Hz, 2H), 7.41 (t, *J* = 7.3 Hz, 1H), 7.36 (t, *J* = 7.3 Hz, 2H), 7.27 (d, *J* = 8.4 Hz, 2H), 7.13 (d, *J* = 8.3 Hz, 2H), 5.85 (s, 1H), 4.51 (s, 2H), 3.81 (s, 2H).

^{13}C NMR (75 MHz, $CDCl_3$) δ 170.3, 152.5, 135.9, 135.7, 133.4, 129.9, 129.6, 129.2, 128.9, 124.8, 123.6, 55.1, 45.3.
HRMS (ESI-TOF) Calc. for $C_{17}H_{14}ClNOSe$ $[M+H]^+$: 364.0000; found: 363.9997

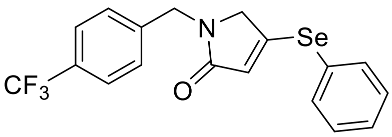

4-(phenylselanyl)-1-(4-(trifluoromethyl)benzyl)-1,5-dihydro-2H-pyrrol-2-one(4d): (50.9 mg, white solid, m.p. = 88-90 °C, yield: 64%)

^{1}H NMR (500 MHz, $CDCl_3$) δ 7.58 (dd, *J* = 16.1, 7.5 Hz, 4H), 7.42 (t, *J* = 7.4 Hz, 1H), 7.36 (t, *J* = 7.3 Hz, 2H), 7.31 (d, *J* = 8.0 Hz, 2H), 5.87 (s, 1H), 4.60 (s, 2H), 3.84 (s, 2H).
^{13}C NMR (75 MHz, $CDCl_3$) δ 167.4, 152.7, 141.2, 136.0, 130.1, 129.9, 129.7, 128.0, 125.7 (q, $J_{C\text{-}F}$ = 3.75 Hz), 125.2 (q, $J_{C\text{-}F}$ = 17.3 Hz), 124.7, 123.5, 122.2, 55.2, 45.5.
^{19}F NMR (565 MHz, $CDCl_3$) δ -62.6.
HRMS (ESI-TOF) Calc. for $C_{18}H_{14}F_3NOSe$ $[M+H]^+$: 398.0266; found: 398.0263.

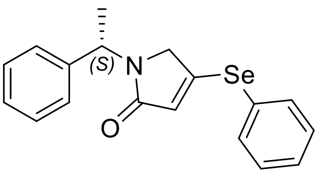

(S)-1-(1-phenylethyl)-4-(phenylselanyl)-1,5-dihydro-2H-pyrrol-2-one(4e): (42.6 mg, yellow solid, m.p. = 78-80 °C, yield: 62%)

^{1}H NMR (500 MHz, $CDCl_3$) δ 7.58 (d, *J* = 6.9 Hz, 2H), 7.40 (t, *J* = 7.3 Hz, 1H), 7.37 – 7.30 (m, 4H), 7.29 – 7.24 (m, 3H), 5.78 (s, 1H), 5.51 (q, *J* = 7.1 Hz, 1H), 3.91 (d, *J* = 19.9 Hz, 1H), 3.62 (d, *J* = 19.9 Hz, 1H), 1.55 (d, *J* = 7.1 Hz, 3H).
^{13}C NMR (75 MHz, $CDCl_3$) δ 169.9, 152.1, 140.7, 135.8, 129.8, 129.5, 128.6, 127.4, 126.9, 125.0, 123.9, 51.5, 49.0, 17.5.
HRMS (ESI-TOF) Calc. for $C_{18}H_{17}NOSe$ $[M+H]^+$: 344.0549; found: 344.0547.

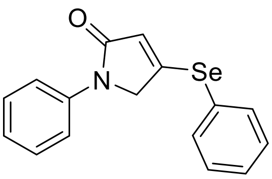

1-phenyl-4-(phenylselanyl)-1,5-dihydro-2H-pyrrol-2-one(4f): (32.1 mg, white solid, m.p. = 138-140 °C, yield: 52%)

^{1}H NMR (500 MHz, $CDCl_3$) δ 7.67 (d, *J* = 7.1 Hz, 2H), 7.57 (d, *J* = 7.7 Hz, 2H), 7.45 (d, *J* = 7.2 Hz, 1H), 7.41 (t, *J* = 7.4 Hz, 2H), 7.33 (t, *J* = 8.0 Hz, 2H), 7.09 (t, *J* = 7.3 Hz, 1H), 5.94 (s, 1H), 4.37 (s, 2H).
^{13}C NMR (126 MHz, $CDCl_3$) δ 168.9, 151.9, 138.9, 136.0, 130.0, 129.8, 129.0, 125.1, 124.7, 123.9, 118.6, 56.0.
HRMS (ESI-TOF) Calc. for $C_{16}H_{13}NOSe$ $[M+H]^+$: 316.0236; found: 316.0232.

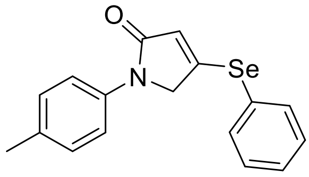

4-(phenylselanyl)-1-(p-tolyl)-1,5-dihydro-2H-pyrrol-2-one(4g): (43.5 mg, white solid, m.p. = 123-125 °C, yield: 66%)

^{1}H NMR (500 MHz, $CDCl_3$) δ 7.66 (d, *J* = 7.1 Hz, 2H), 7.49 – 7.33 (m, 5H), 7.13 (d, *J* = 8.3 Hz, 2H), 5.93 (s, 1H), 4.34 (s, 2H), 2.30 (s, 3H).

^{13}C NMR (75 MHz, $CDCl_3$) δ 168.8, 151.6, 136.4, 136.0, 133.6, 129.9, 129.7, 129.5, 125.1, 124.7, 118.8, 56.2, 20.7.

HRMS (ESI-TOF) Calc. for $C_{17}H_{15}NOSe$ $[M+H]^+$: 330.0392; found: 330.0395.

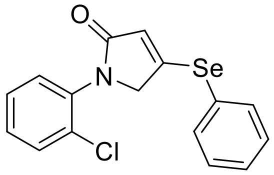

1-(2-chlorophenyl)-4-(phenylselanyl)-1,5-dihydro-2H-pyrrol-2-one(4h): (44.7 mg, yellow liquid, yield: 64%)

^{1}H NMR (500 MHz, $CDCl_3$) δ 7.71 – 7.65 (m, 2H), 7.47 – 7.37 (m, 4H), 7.35 – 7.20 (m, 4H), 5.99 (d, *J* = 1.5 Hz, 1H), 4.34 (d, *J* = 1.5 Hz, 2H).

^{13}C NMR (75 MHz, $CDCl_3$) δ 169.8, 153.9, 136.1, 135.2, 132.4, 130.5, 130.0, 129.9, 129.8, 129.1, 127.7, 124.6, 123.3, 57.4.

HRMS (ESI-TOF) Calc. for $C_{16}H_{12}ClNOSe$ $[M+H]^+$: 349.9843; found: 349.9841.

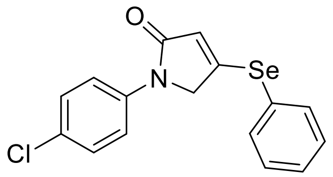

1-(4-chlorophenyl)-4-(phenylselanyl)-1,5-dihydro-2H-pyrrol-2-one(4i): (43.1 mg, white solid, m.p. = 79-81 °C, yield: 62%)

^{1}H NMR (500 MHz, $CDCl_3$) δ 7.67 (d, *J* = 7.1 Hz, 2H), 7.53 (d, *J* = 8.9 Hz, 2H), 7.47 (t, *J* = 7.3 Hz, 1H), 7.42 (t, *J* = 7.4 Hz, 2H), 7.27 (d, *J* = 8.9 Hz, 2H), 5.92 (s, 1H), 4.34 (s, 2H).

^{13}C NMR (75 MHz, $CDCl_3$) δ 168.6, 152.4, 137.5, 136.1, 130.0, 129.9, 129.0, 128.9, 124.8, 124.5, 119.5, 55.8.

HRMS (ESI-TOF) Calc. for $C_{16}H_{12}ClNOSe$ $[M+H]^+$: 349.9843; found: 349.9842.

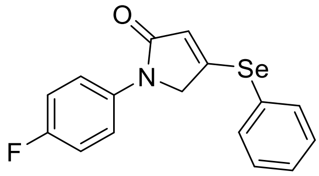

1-(4-fluorophenyl)-4-(phenylselanyl)-1,5-dihydro-2H-pyrrol-2-one(4j): (43.3 mg, white solid, m.p. = 124-126 °C, yield: 65%)

^{1}H NMR (500 MHz, $CDCl_3$) δ 7.66 (d, J = 7.2 Hz, 2H), 7.52 (dd, J = 9.1, 4.7 Hz, 2H), 7.46 (t, J = 7.3 Hz, 1H), 7.41 (t, J = 7.4 Hz, 2H), 7.01 (t, J = 8.7 Hz, 2H), 5.92 (s, 1H), 4.33 (s, 2H).

^{13}C NMR (75 MHz, $CDCl_3$) δ 168.8, 159.1 (d, J = 403.2 Hz), 152.1, 136.0, 135.0 (d, $J_{C\text{-}F}$ = 2.8 Hz), 130.0, 129.8, 124.7, 124.5, 120.4 (d, $J_{C\text{-}F}$ = 7.8 Hz), 115.7 (d, J =37.8Hz), 56.2.

^{19}F NMR (565 MHz, $CDCl_3$) δ -118.4.

HRMS (ESI-TOF) Calc. for $C_{16}H_{12}FNOSe$ $[M+H]^+$: 334.0142; found: 334.0140.

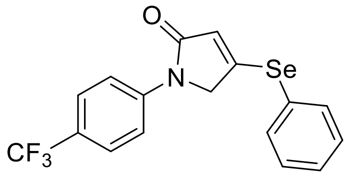

4-(phenylselanyl)-1-(4-(trifluoromethyl)phenyl)-1,5-dihydro-2H-pyrrol-2-one(4k): (36.1 mg, white solid, m.p. = 103-105 °C, yield: 47%)

^{1}H NMR (500 MHz, $CDCl_3$) δ 7.72 (d, J = 8.7 Hz, 2H), 7.68 (d, J = 7.1 Hz, 2H), 7.57 (d, J = 8.6 Hz, 2H), 7.49 (t, J = 7.3 Hz, 1H), 7.43 (t, J = 7.3 Hz, 2H), 5.93 (s, 1H), 4.40 (s, 2H).

^{13}C NMR (75 MHz, $CDCl_3$) δ 168.0, 153.4, 141.9, 136.2, 136.2, 130.2, 130.1, 126.3 (q, J = 3.75 Hz), 125.6 (q, J = 214 Hz), 124.7, 124.4, 117.6, 55.7.

^{19}F NMR (565 MHz, $CDCl_3$) δ -62.1.

HRMS (ESI-TOF) Calc. for $C_{17}H_{12}F_3NOSe$ $[M+H]^+$: 384.0101; found: 384.0101.

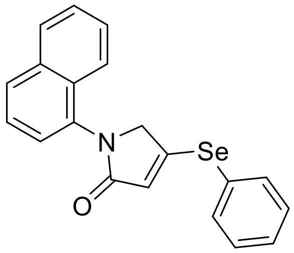

1-(naphthalen-1-yl)-4-(phenylselanyl)-1,5-dihydro-2H-pyrrol-2-one(4l): (38.7 mg, yellow liquid, yield: 53%)

^{1}H NMR (300 MHz, $CDCl_3$) δ 7.92 – 7.81 (m, 2H), 7.75 – 7.67 (m, 3H), 7.55 – 7.47 (m, 3H), 7.46 – 7.33 (m, 4H), 6.10 (s, 1H), 4.41 (d, J = 1.3 Hz, 2H).

^{13}C NMR (75 MHz, $CDCl_3$) δ 170.3, 153.5, 136.1, 134.5, 134.3, 130.4, 129.9, 129.7, 128.5, 128.4, 126.8, 126.4, 125.5, 124.5, 123.6, 122.8, 59.4.

HRMS (ESI-TOF) Calc. for $C_{20}H_{15}NOSe$ $[M+H]^+$: 366.0393; found: 366.0396.

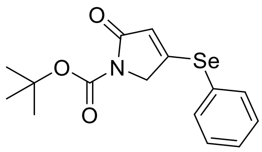

tert-butyl 2-oxo-4-(phenylselanyl)-2,5-dihydro-1H-pyrrole-1-carboxylate(4m): (38.7 mg, yellow liquid, yield: 57%)

^{1}H NMR (500 MHz, $CDCl_3$) δ 7.62 (d, *J* = 6.9 Hz, 2H), 7.46 (t, *J* = 7.2 Hz, 1H), 7.40 (t, *J* = 7.4 Hz, 2H), 5.74 (s, 1H), 4.29 (s, 2H), 1.51 (s, 9H).

^{13}C NMR (126 MHz, $CDCl_3$) δ 167.3, 157.1, 149.2, 136.2, 130.1, 130.0, 124.0, 122.8, 82.9, 54.3, 28.1.

HRMS (ESI-TOF) Calc. for $C_{15}H_{17}NO_3Se$ $[M+Na]^+$: 362.0266; found: 362.0264.

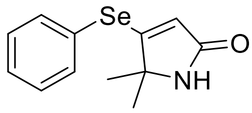

5,5-dimethyl-4-(phenylselanyl)-1,5-dihydro-2H-pyrrol-2-one(4n): (35.3 mg, white solid, m.p. > 270 °C, yield: 66%)

^{1}H NMR (300 MHz, $CDCl_3$) δ 7.61 (dd, *J* = 7.8, 1.7 Hz, 3H), 7.37 (q, *J* = 6.7, 6.2 Hz, 3H), 5.33 (s, 1H), 1.42 (s, 6H).

^{13}C NMR (75 MHz, $CDCl_3$) δ 171.3, 168.7, 136.1, 129.8, 129.5, 125.1, 120.5, 63.7, 27.4.

HRMS (ESI-TOF) Calc. for $C_{12}H_{13}NOSe$ $[M+H]^+$: 268.0236; found: 268.0233.

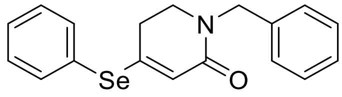

1-benzyl-4-(phenylselanyl)-5,6-dihydropyridin-2(1H)-one(4o): (39.7 mg, yellow liquid, yield: 58%)

^{1}H NMR (600 MHz, $CDCl_3$) δ 7.6 – 7.6 (m, 2H), 7.4 (t, *J* = 7.3 Hz, 2H), 7.4 – 7.3 (m, 3H), 7.3 – 7.3 (m, 3H), 5.8 (s, 1H), 4.6 (s, 2H), 3.3 (t, *J* = 7.0 Hz, 2H), 2.5 (t, *J* = 7.0 Hz, 2H).

^{13}C NMR (151 MHz, $CDCl_3$) δ 163.5, 150.4, 137.9, 136.5, 129.9, 129.6, 129.2, 128.6, 128.0, 127.4, 120.5, 49.5, 44.9, 30.2.

HRMS (ESI-TOF) Calc. for $C_{18}H_{17}NOSe$ $[M+H]^+$: 344.0549; found: 344.0545.

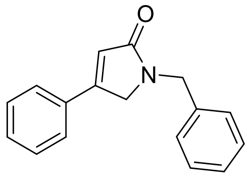

1-benzyl-4-phenyl-1,5-dihydro-2H-pyrrol-2-one (4s)[3]: (32.5 mg, yellow solid, m.p. =162-163 °C, yield: 65%)

^{1}H NMR (300 MHz, $CDCl_3$) δ 7.47 – 7.41 (m, 2H), 7.36 (dd, *J* = 7.3, 4.1 Hz, 4H), 7.33 (s, 1H), 7.31 – 7.27 (m, 3H), 6.48 (s, 1H), 4.70 (s, 2H), 4.23 (s, 2H).

^{13}C NMR (75 MHz, $CDCl_3$) δ 171.6, 154.4, 137.2, 131.7, 130.1, 128.9, 128.8, 127.9, 127.6, 125.7, 120.1, 51.7, 45.9.

4. References

(1) Joyce, L. M.; Moggach, S. A.; Hyland, C. J. T.; Pyne, S. G.; Stewart, S. G. Nickel Phosphite-Catalyzed Tetradehydro-Diels–Alder Reactions of (E)-3-Ene-1,8-Diynes. *J. Org. Chem.* 2023, *88* (9), 5391–5402. https://doi.org/10.1021/acs.joc.2c03040.

(2) Guo, J.; Zhang, Y.; Zhang, C.; Yao, C.; Zhang, J.; Jiang, X.; Zhong, Z.; Ge, J.; Zhou, T.; Bai, R.; Xie, Y. N-Propargylamine-Hydroxypyridinone Hybrids as Multitarget Agents for the Treatment of Alzheimer's Disease. *Bioorganic Chemistry* 2021, *113*, 105013. https://doi.org/10.1016/j.bioorg.2021.105013.

(3) Ding, Z.; Luo, Y.; Yuan, Q.; Wang, G.; Yu, Z.; Zhao, M.; Liu, D.; Zhang, W. Ru-Catalyzed Asymmetric Hydrogenation of α, β-Unsaturated γ-Lactams. *J. Am. Chem. Soc.* 2024. https://doi.org/10.1021/jacs.4c09794.

5. Copies of NMR Spectra of Products

lzr-yzp-0623-14-H.6.fid

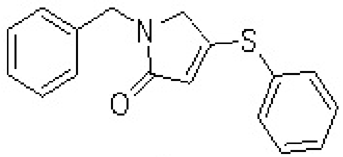

3a

400 MHz, $CDCl_3$

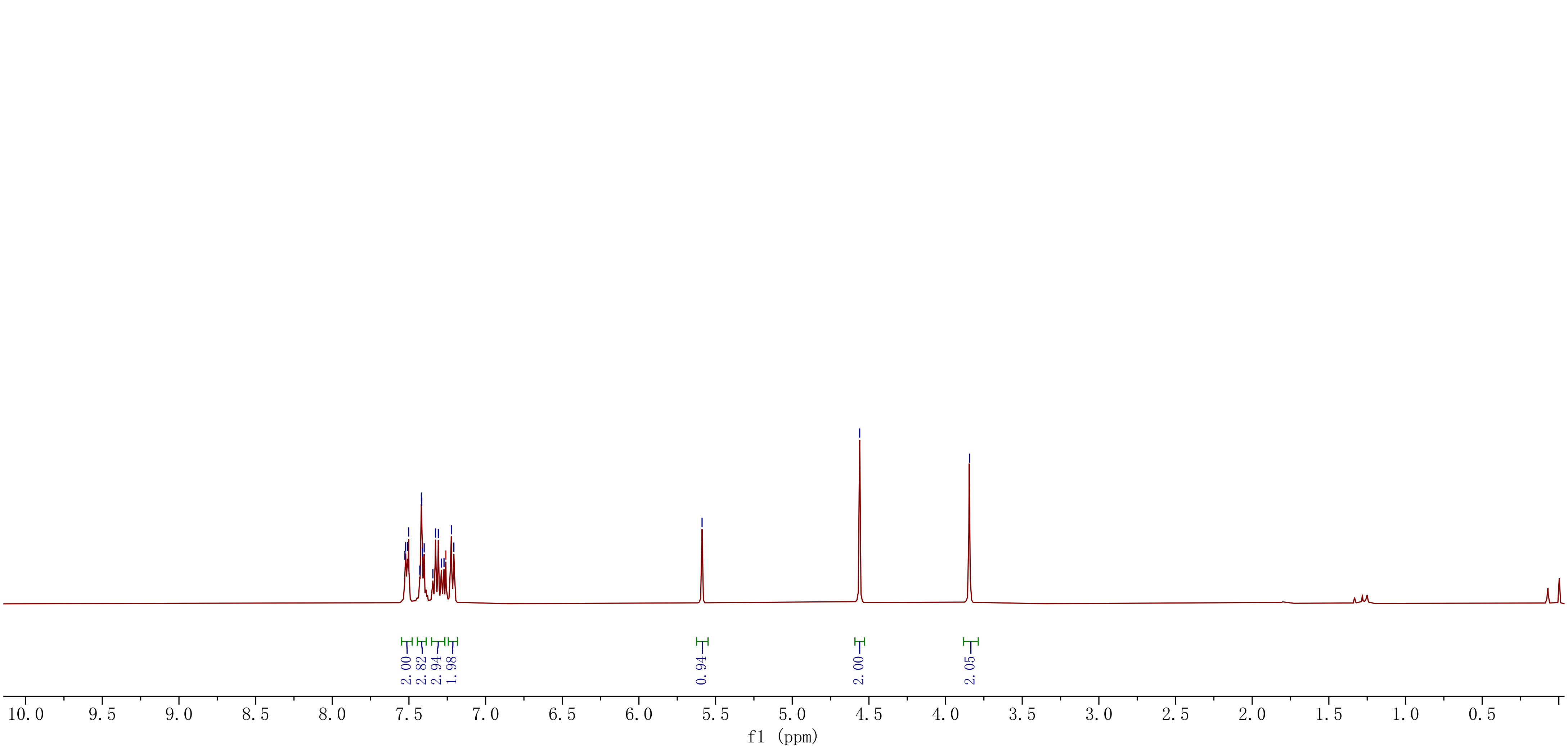

lzr-y-0625-14-C.3.fid

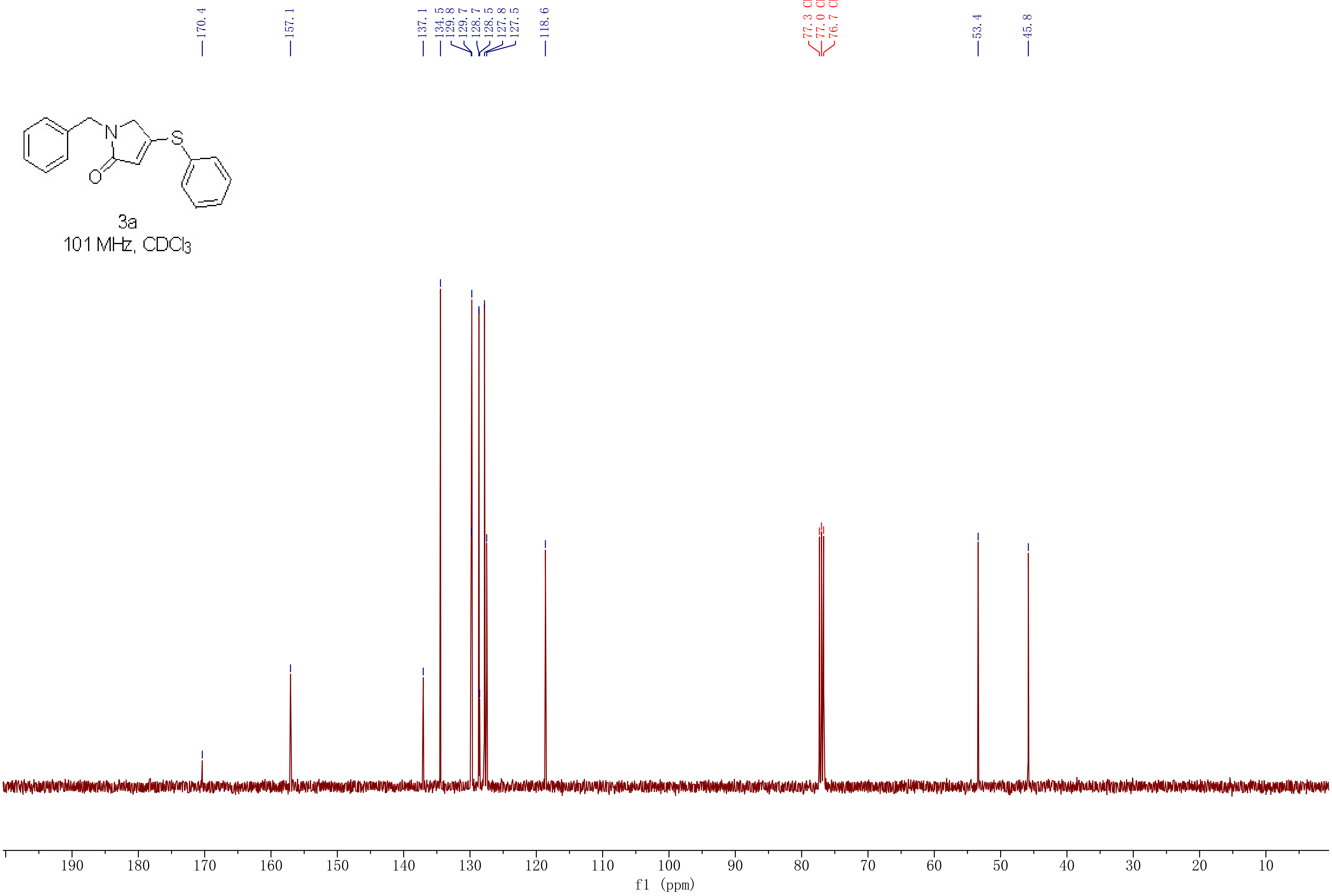

3a

101 MHz, $CDCl_3$

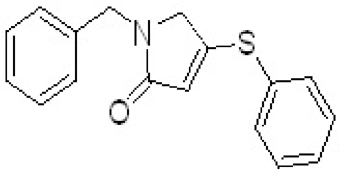

Qualitative Compound Identification Report

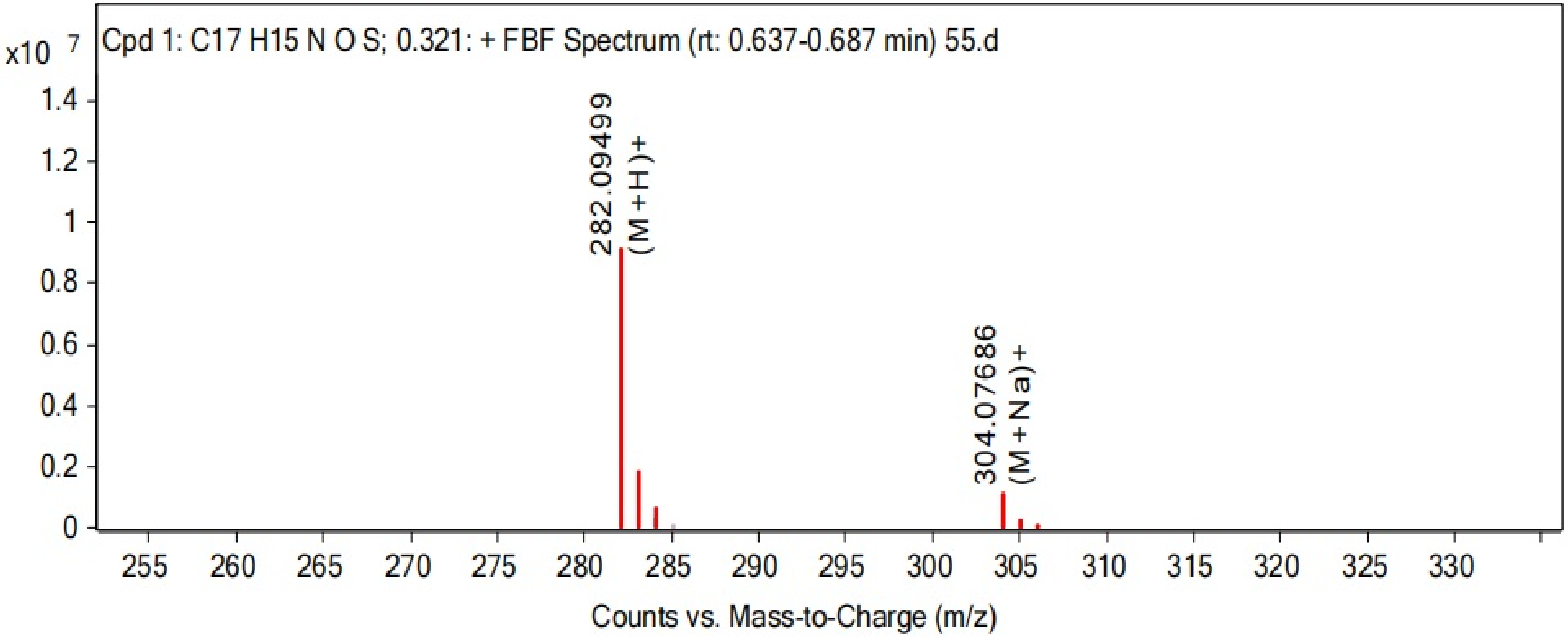

MS Spectrum Peak List

m/z	z	Abund	Ion
282.09499	1	9181640	(M+H)+
283.09773	1	1311441.63	(M+H)+
284.09068	1	335554.88	(M+H)+
304.07686	1	1117880.38	(M+Na)+
305.07975	1	213889.39	(M+Na)+
306.0727	1	55400.3	(M+Na)+

MS Spectrum

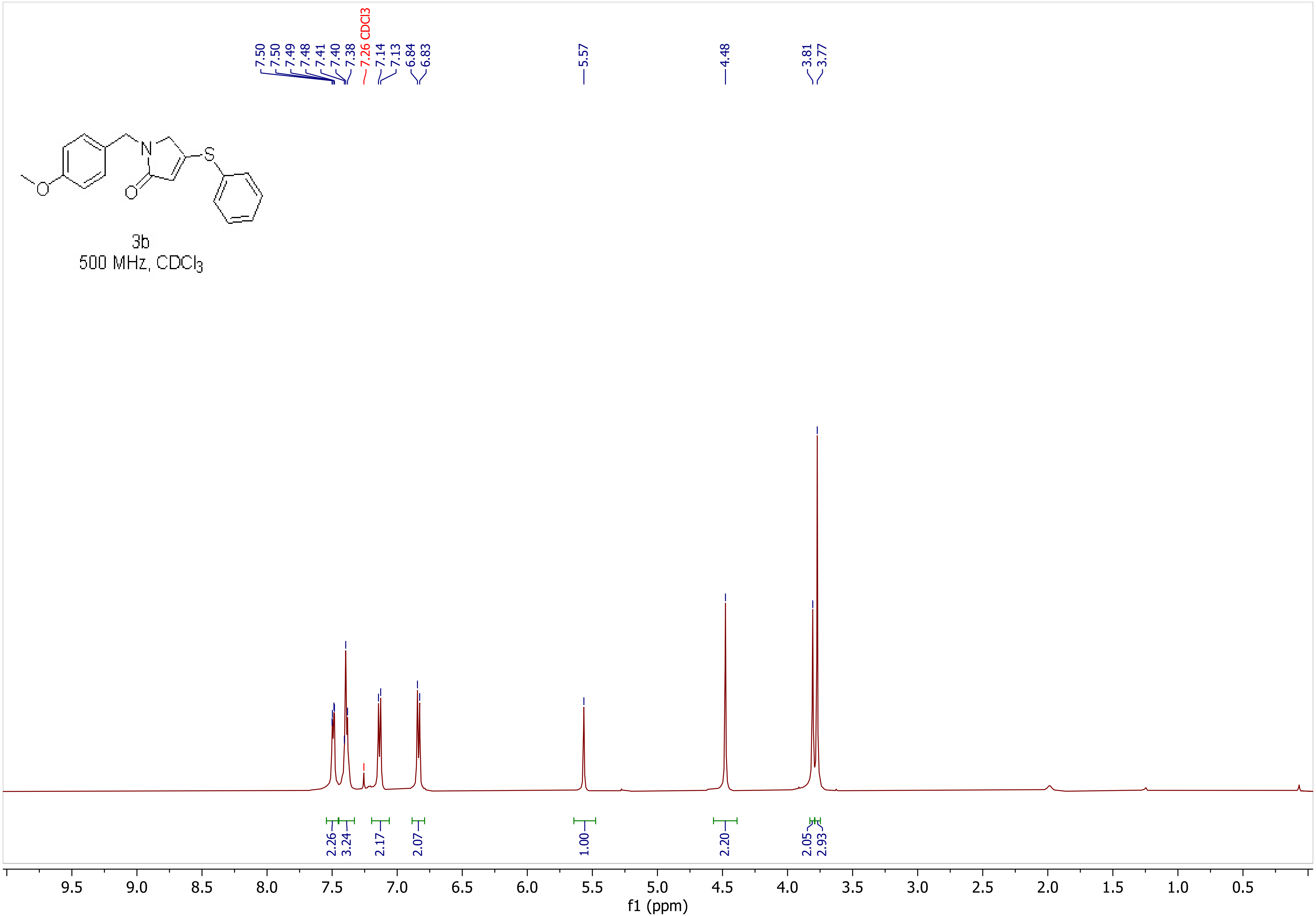

3b

500 MHz, $CDCl_3$

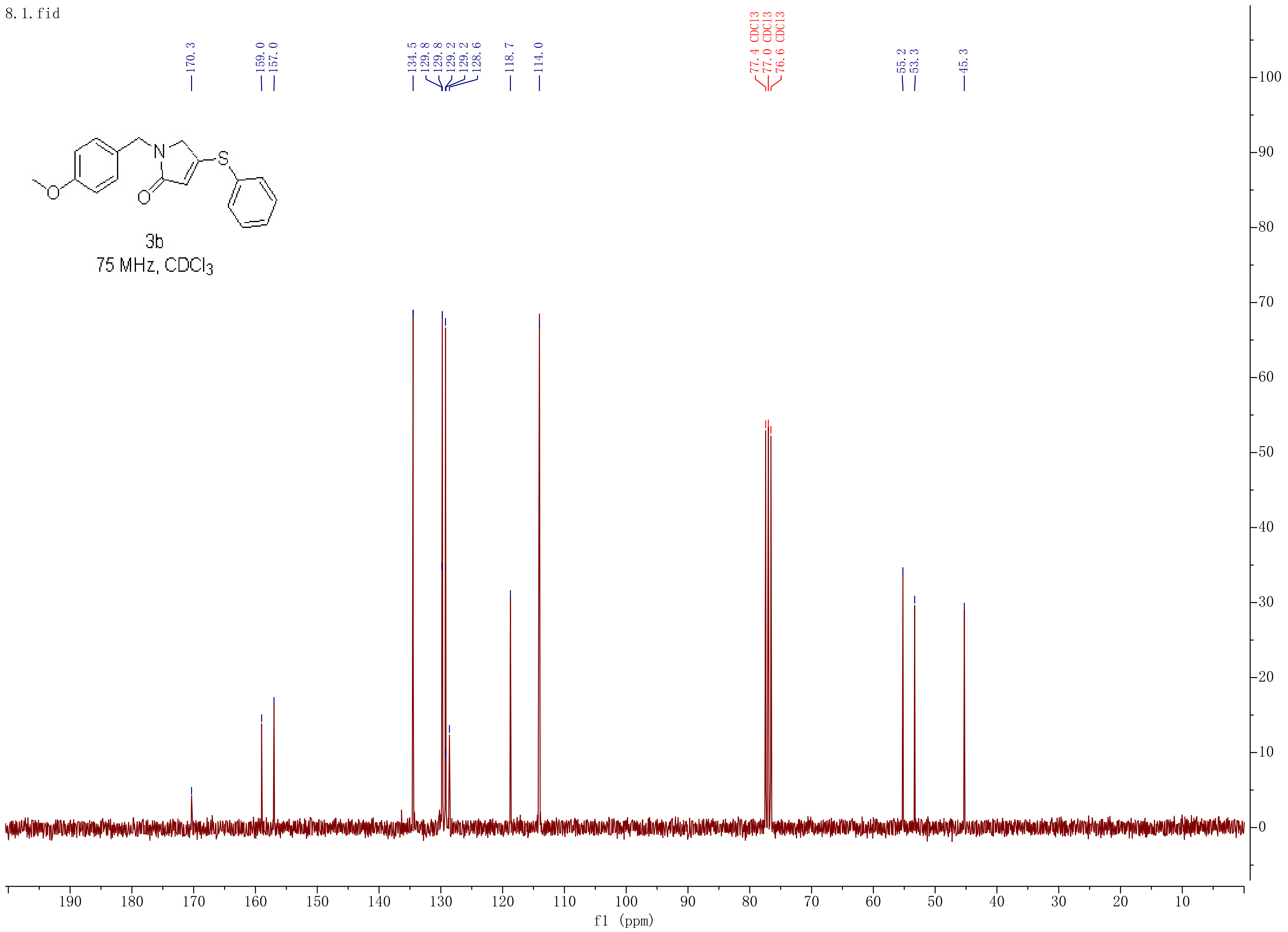
8.1.fid
170.3
159.0
157.0
134.5
129.8
129.8
129.2
129.2
128.6
118.7
114.0
77.4 CDCl3
77.0 CDCl3
76.6 CDCl3
55.2
53.3
45.3
3b
75 MHz, CDCl3
f1 (ppm)

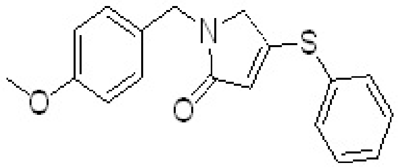

Qualitative Compound Identification Report

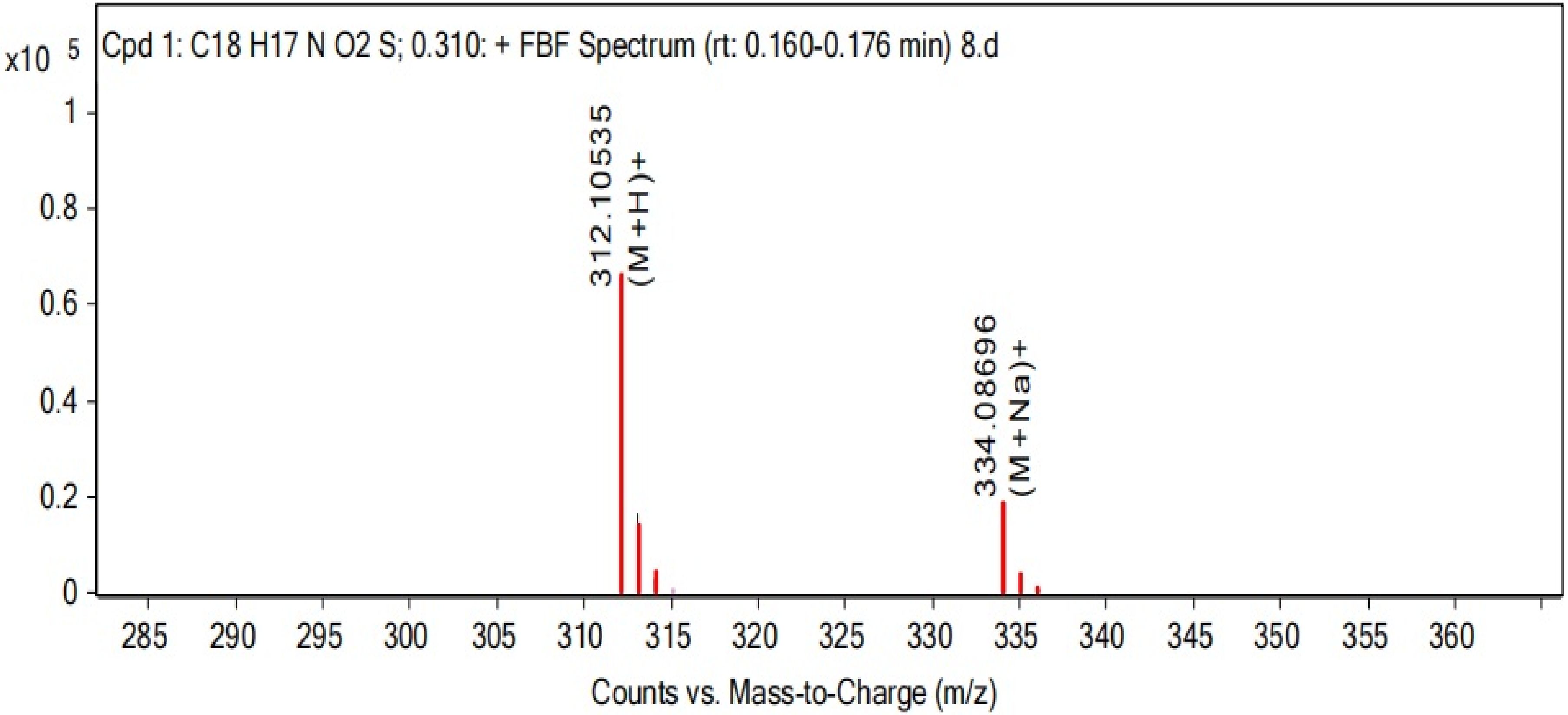

MS Spectrum Peak List

m/z	z	Abund	Ion
312.10535	1	65692.65	(M+H)+
313.1082	1	16479.03	(M+H)+
314.10248	1	3027.96	(M+H)+
334.08696	1	18724.93	(M+Na)+
335.09069	1	4290.88	(M+Na)+

MS Spectrum

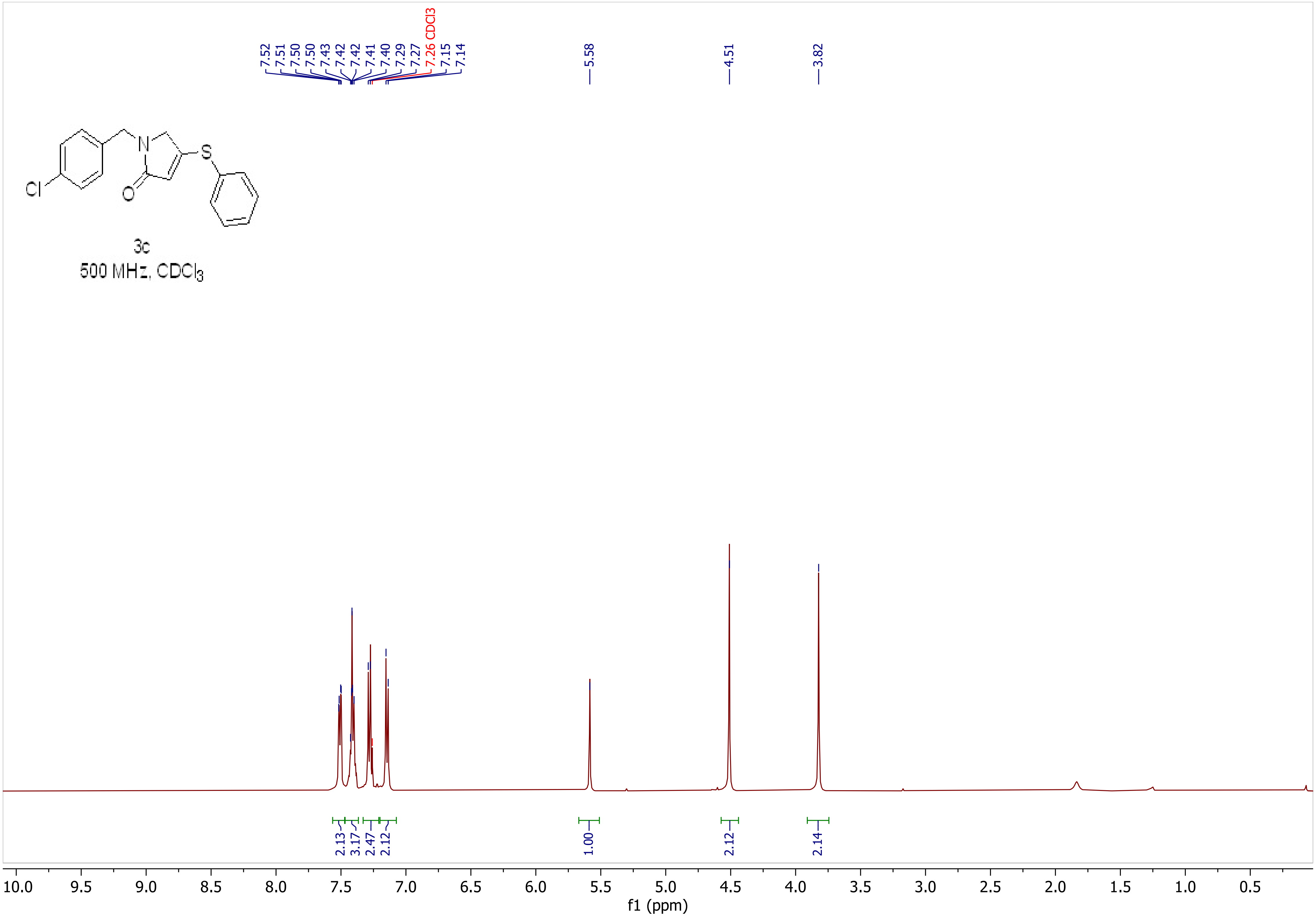
7.52
7.51
7.50
7.50
7.43
7.42
7.42
7.41
7.40
7.29
7.27
7.26 CDCl3
7.15
7.14
5.58
4.51
3.82
Cl
N
O
S
3c
500 MHz, CDCl3
2.13
3.17
2.47
2.12
1.00
2.12
2.14
10.0
9.5
9.0
8.5
8.0
7.5
7.0
6.5
6.0
5.5
5.0
4.5
4.0
3.5
3.0
2.5
2.0
1.5
1.0
0.5
f1 (ppm)

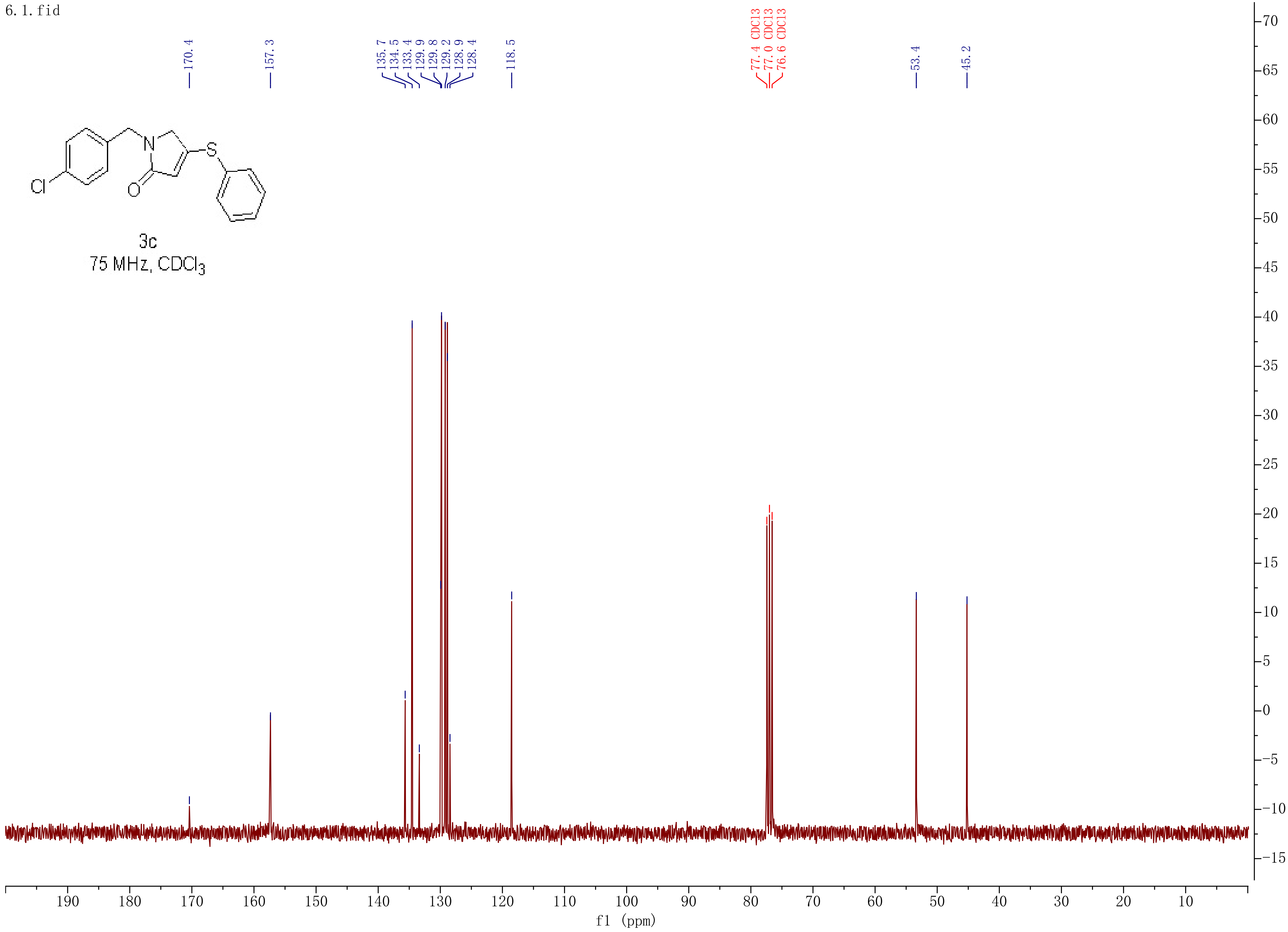
6.1.fid
170.4
157.3
135.7
134.5
133.4
129.9
129.8
129.2
128.9
128.4
118.5
77.4 CDCl3
77.0 CDCl3
76.6 CDCl3
53.4
45.2
Cl
N
S
O
3c
75 MHz, CDCl3
f1 (ppm)

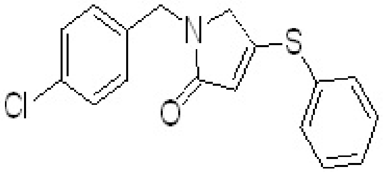

Qualitative Compound Identification Report

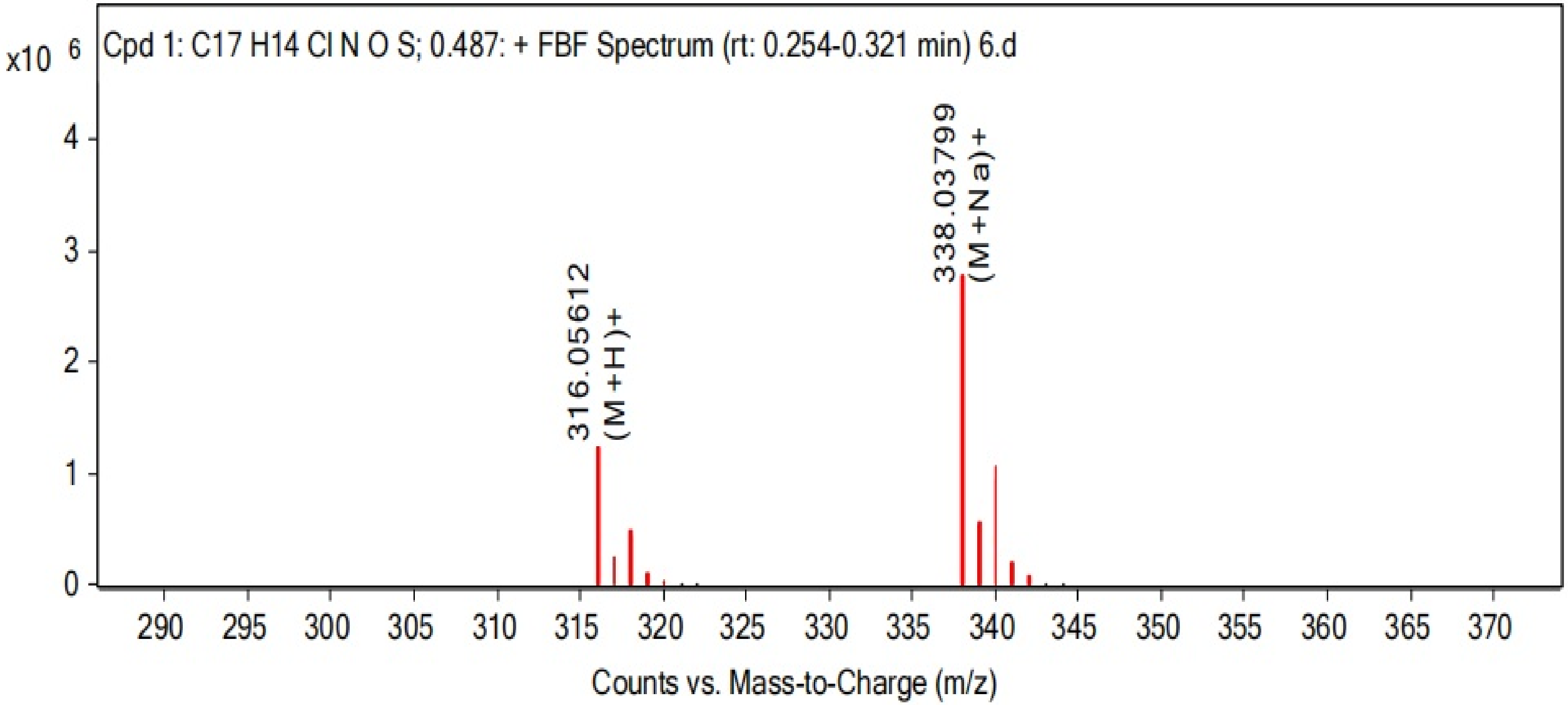

MS Spectrum Peak List

m/z	z	Abund	Ion
316.05612	1	1223768.25	(M+H)+
318.05281	1	426165.69	(M+H)+
338.03799	1	2778774	(M+Na)+
339.04101	1	524590.63	(M+Na)+
340.03482	1	945509.19	(M+Na)+

MS Spectrum

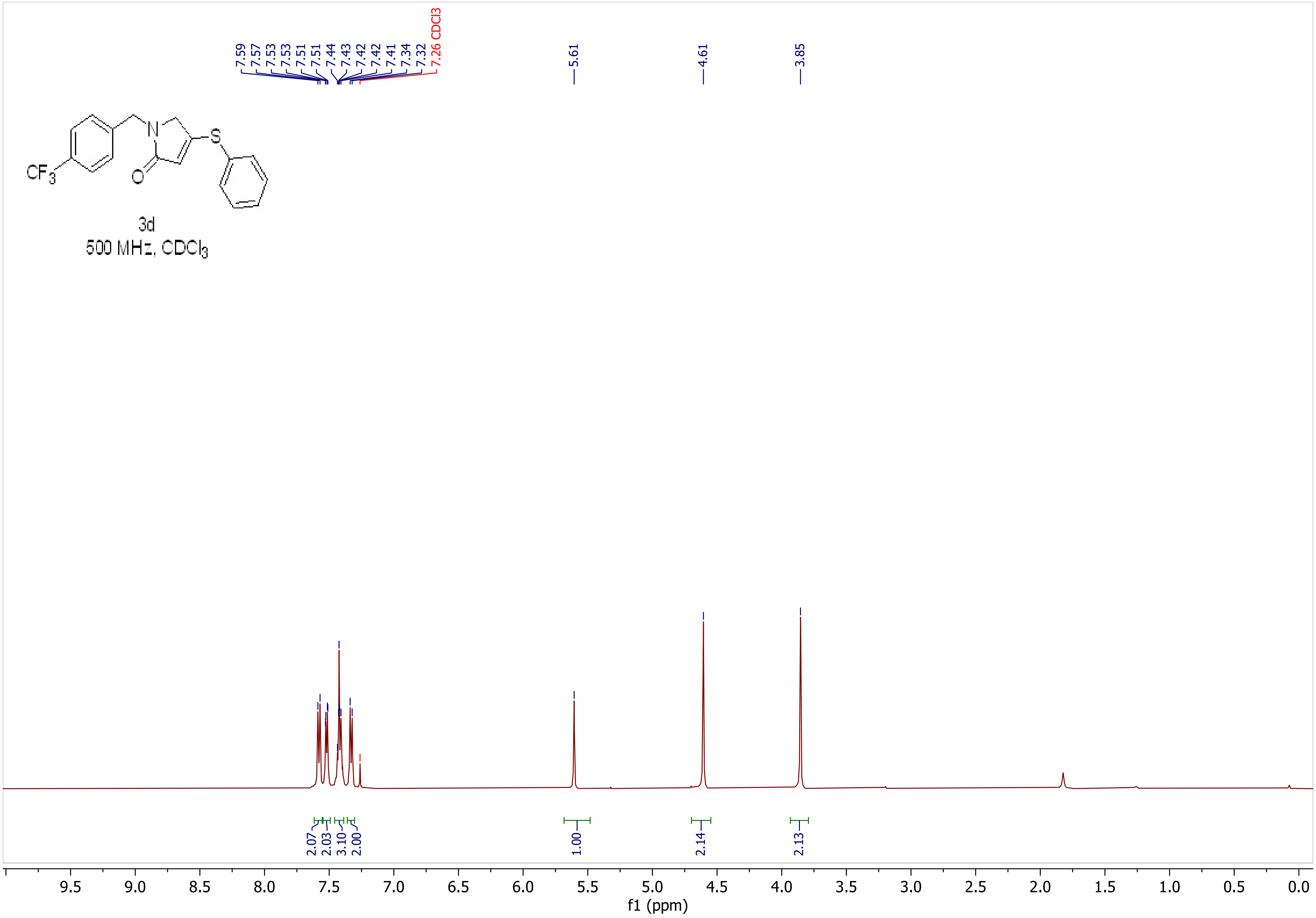
7.59
7.57
7.53
7.53
7.51
7.51
7.44
7.43
7.42
7.42
7.41
7.34
7.32
7.26 CDCl3
5.61
4.61
3.85
CF3
N
O
S
3d
500 MHz, CDCl3
2.07
2.03
3.10
2.00
1.00
2.14
2.13
9.5
9.0
8.5
8.0
7.5
7.0
6.5
6.0
5.5
5.0
4.5
4.0
3.5
3.0
2.5
2.0
1.5
1.0
0.5
0.0
f1 (ppm)

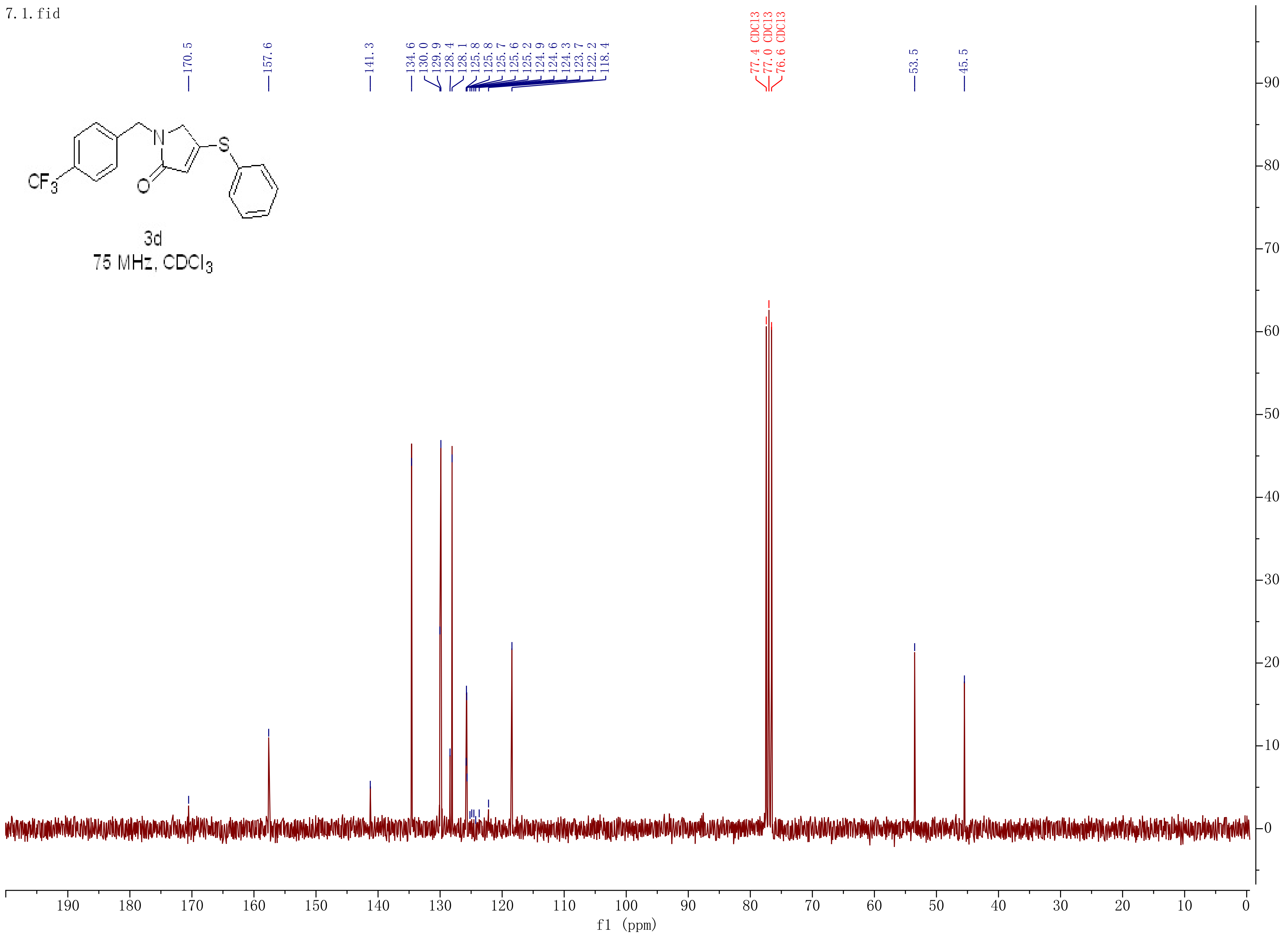

7.1.fid
170.5
157.6
141.3
134.6
130.0
129.9
128.4
128.1
125.8
125.8
125.7
125.6
125.2
124.9
124.6
124.3
123.7
122.2
118.4
77.4 CDCl3
77.0 CDCl3
76.6 CDCl3
53.5
45.5
CF3
N
S
O
3d
75 MHz, CDCl3
f1 (ppm)

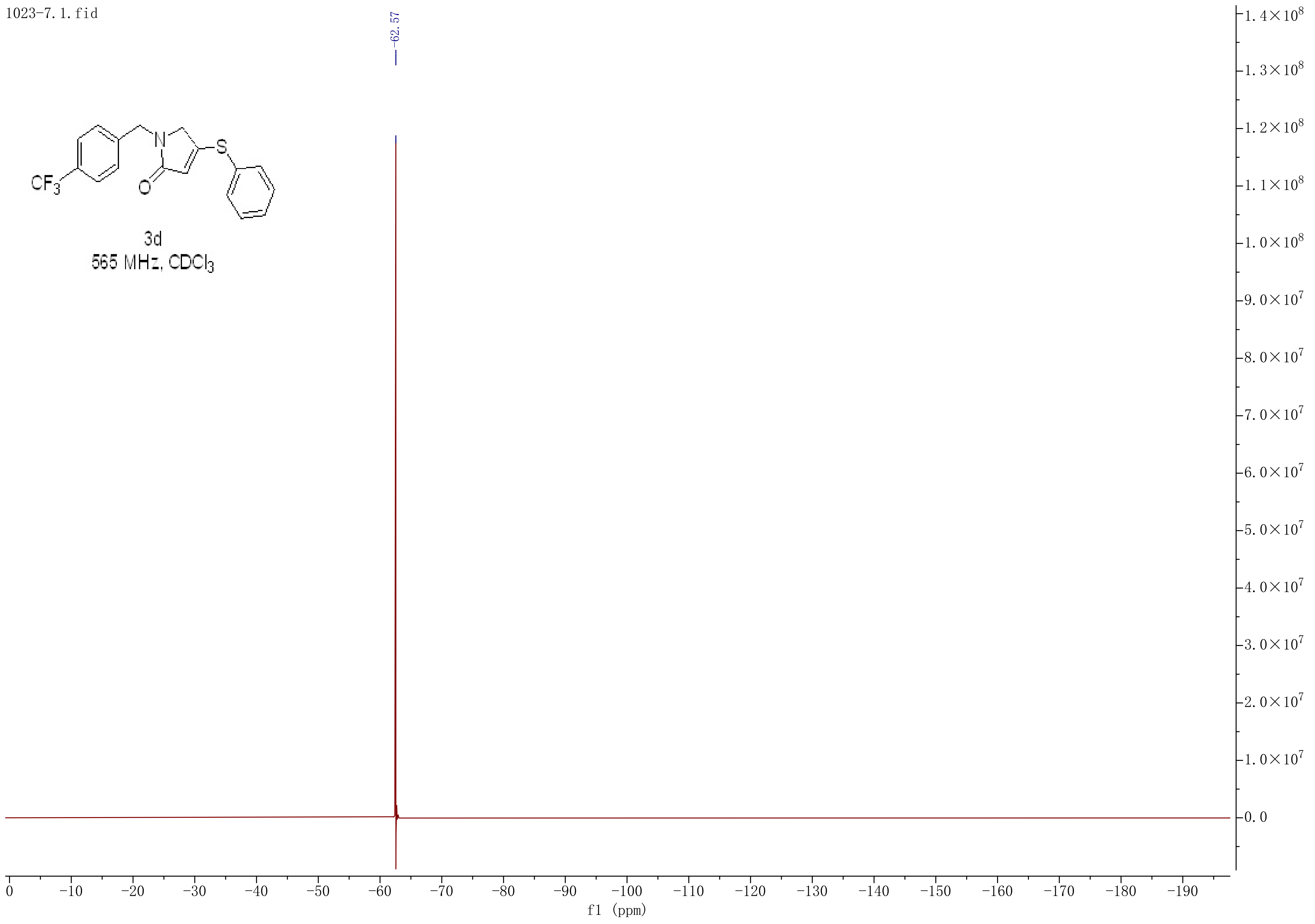
1023-7.1.fid
-62.57
CF3
N
S
O
3d
565 MHz, CDCl3
1.4×10^8
1.3×10^8
1.2×10^8
1.1×10^8
1.0×10^8
9.0×10^7
8.0×10^7
7.0×10^7
6.0×10^7
5.0×10^7
4.0×10^7
3.0×10^7
2.0×10^7
1.0×10^7
0.0
0
−10
−20
−30
−40
−50
−60
−70
−80
−90
−100
−110
−120
−130
−140
−150
−160
−170
−180
−190
f1 (ppm)

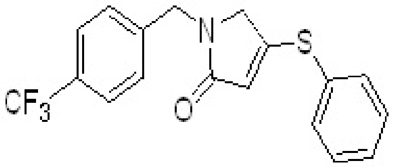

Qualitative Compound Identification Report

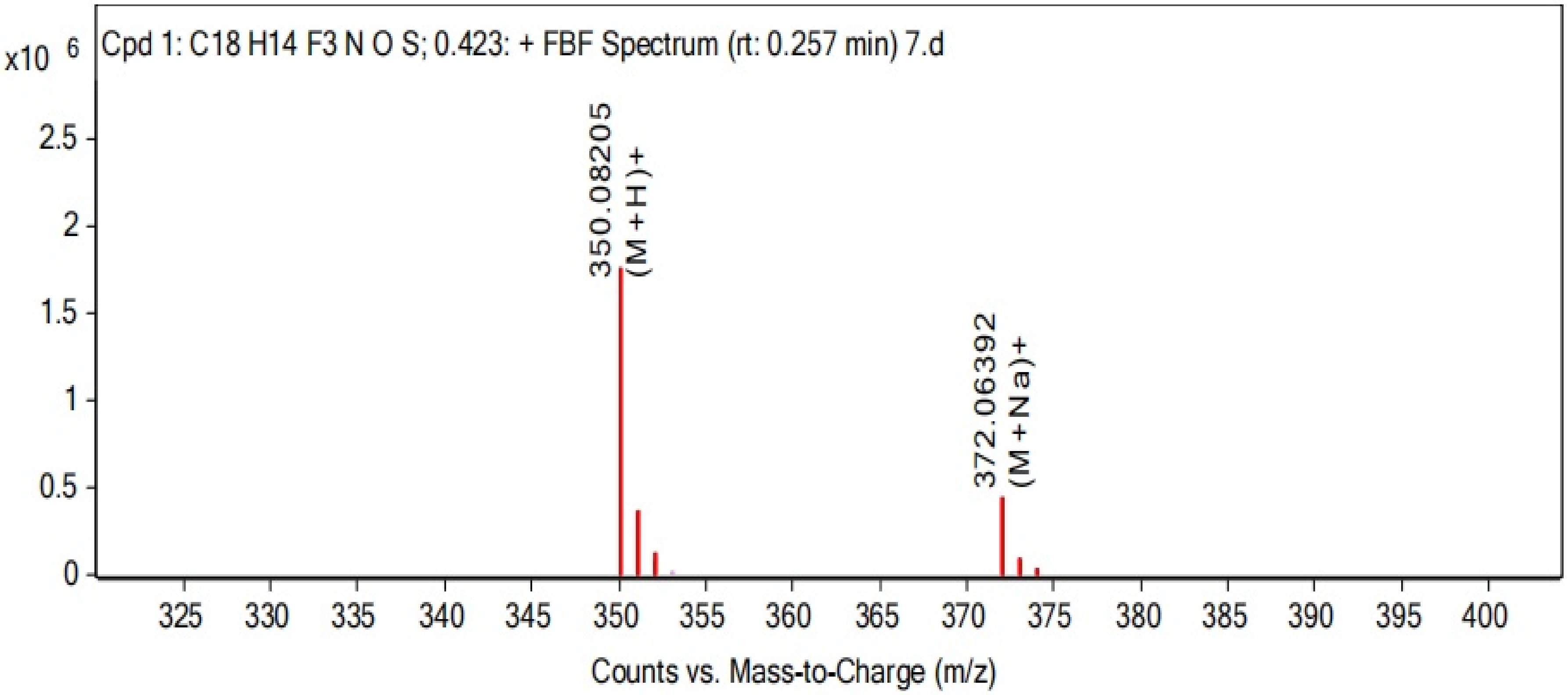

MS Spectrum Peak List

m/z	z	Abund	Ion
350.08205	1	1750775.63	(M+H)+
351.08531	1	356055.75	(M+H)+
352.07872	1	81394.92	(M+H)+
372.06392	1	442216.06	(M+Na)+
373.06717	1	83652.88	(M+Na)+

MS Spectrum

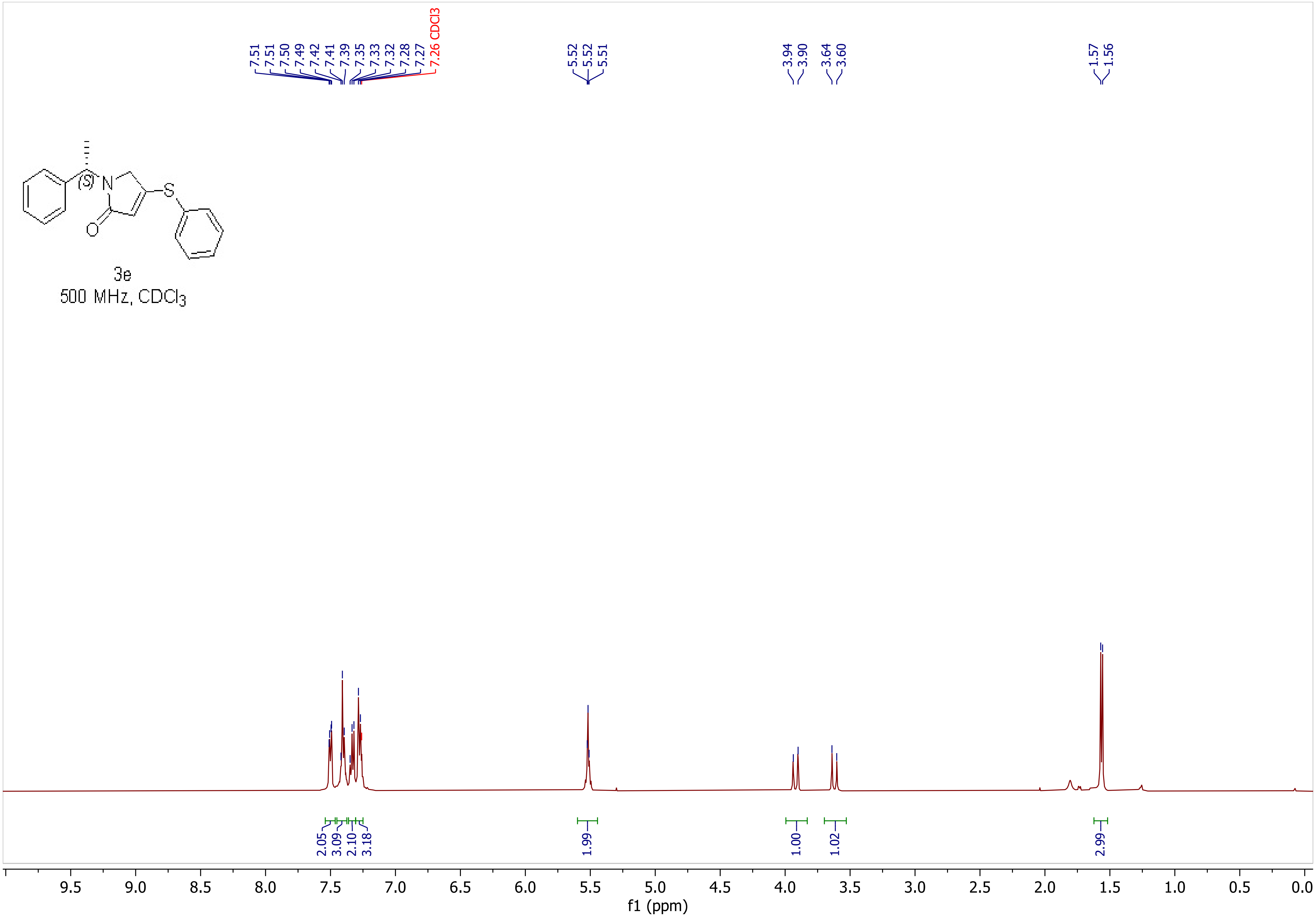

7.51
7.51
7.50
7.49
7.42
7.41
7.39
7.35
7.33
7.32
7.28
7.27
7.26 CDCl3
5.52
5.52
5.51
3.94
3.90
3.64
3.60
1.57
1.56
(S)
N
S
O
3e
500 MHz, CDCl3
2.05
3.09
2.10
3.18
1.99
1.00
1.02
2.99
9.5
9.0
8.5
8.0
7.5
7.0
6.5
6.0
5.5
5.0
4.5
4.0
3.5
3.0
2.5
2.0
1.5
1.0
0.5
0.0
f1 (ppm)

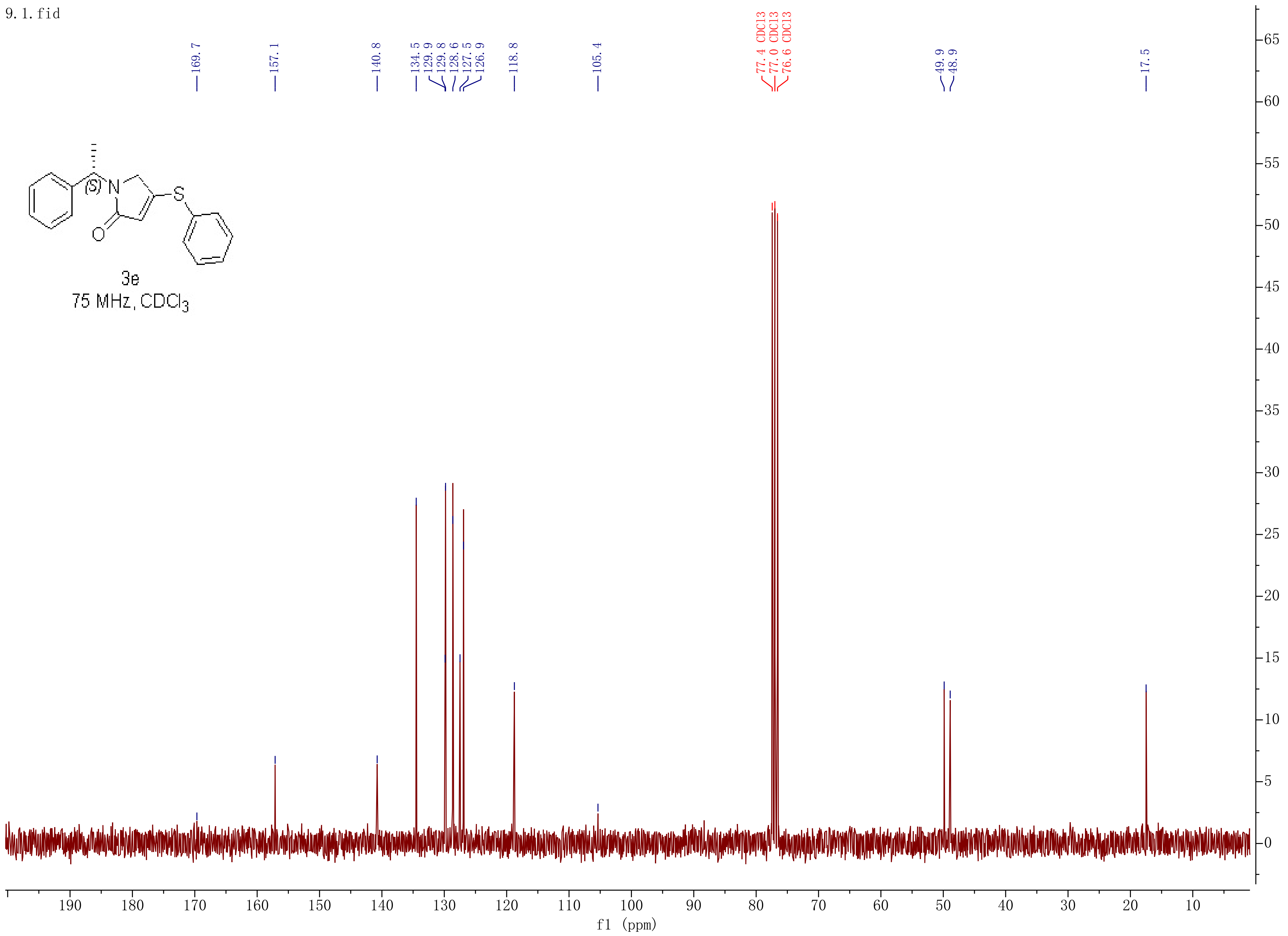
9.1.fid
169.7
157.1
140.8
134.5
129.9
129.8
128.6
127.5
126.9
118.8
105.4
77.4 CDCl3
77.0 CDCl3
76.6 CDCl3
49.9
48.9
17.5
(S)
N
S
O
3e
75 MHz, CDCl3
f1 (ppm)

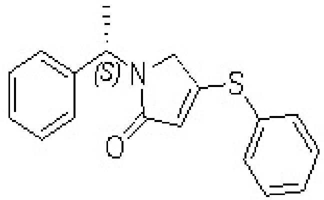

Qualitative Compound Identification Report

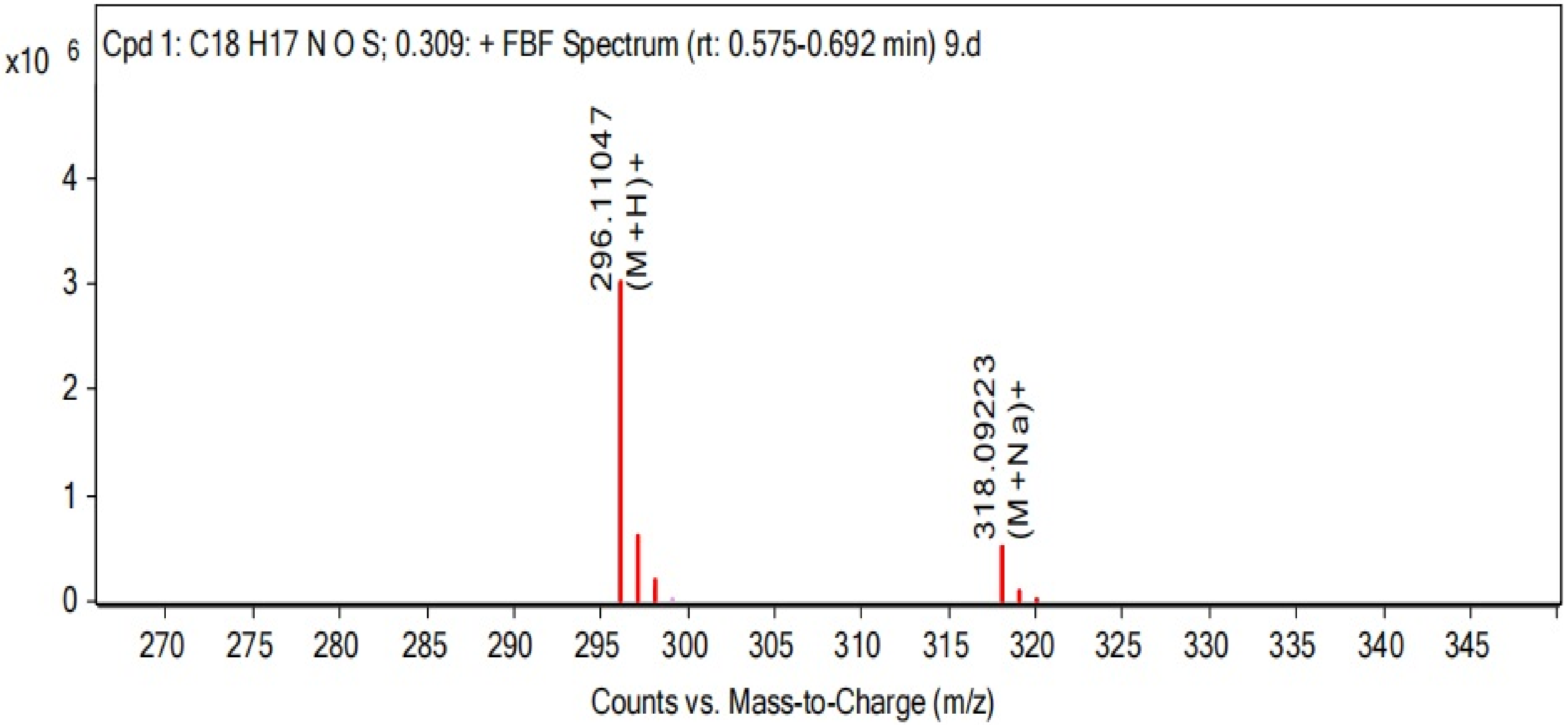

MS Spectrum Peak List

m/z	z	Abund	Ion
296.11047	1	3019620.75	(M+H)+
297.1135	1	570202.56	(M+H)+
298.10652	1	132124.84	(M+H)+
318.09223	1	517228.78	(M+Na)+
319.09556	1	105443.8	(M+Na)+

MS Spectrum

Y-0824-2-H.1.fid

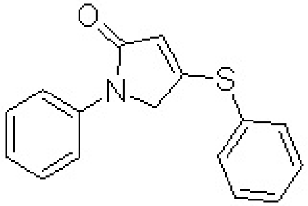

3f

500 MHz, $CDCl_3$

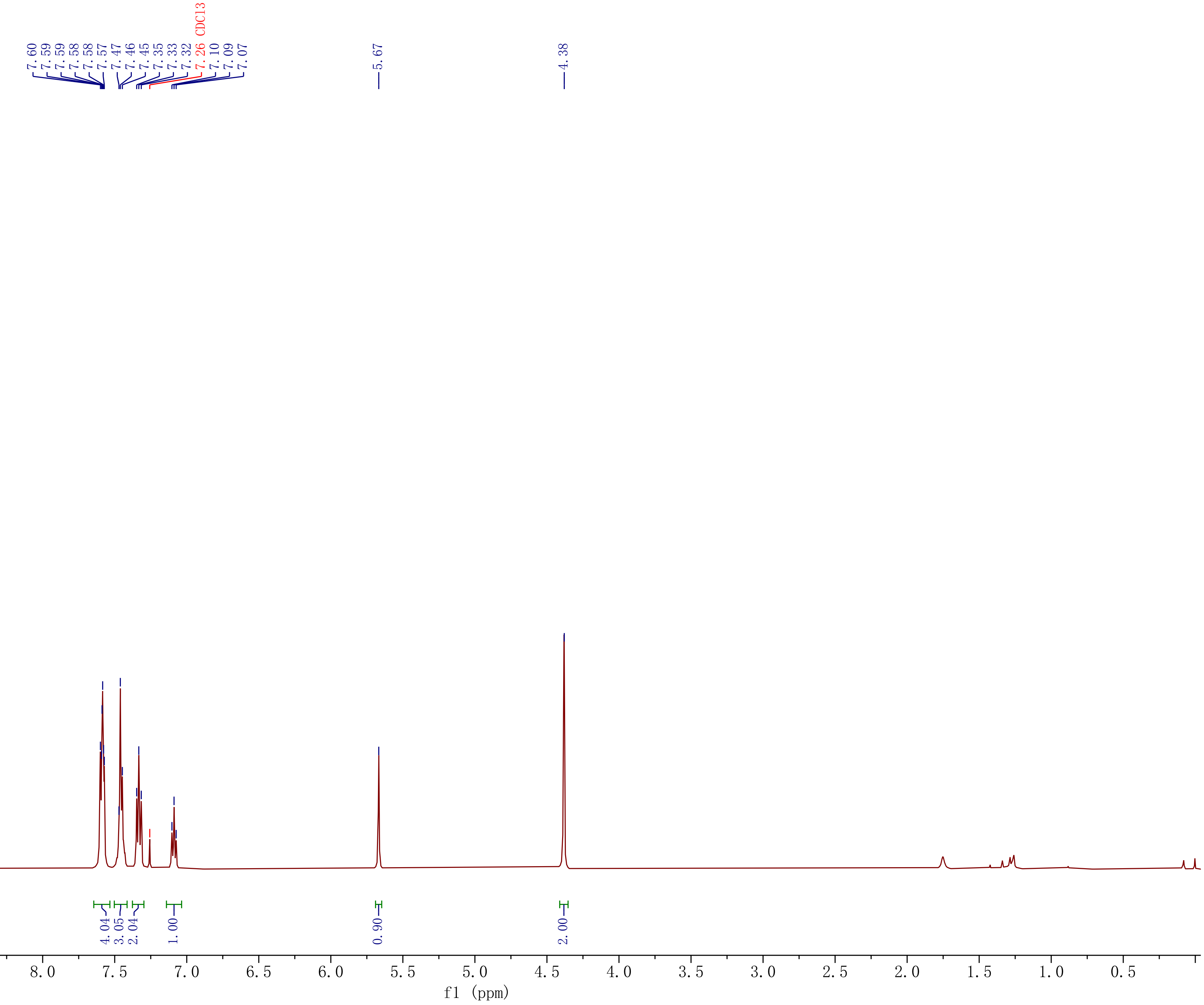

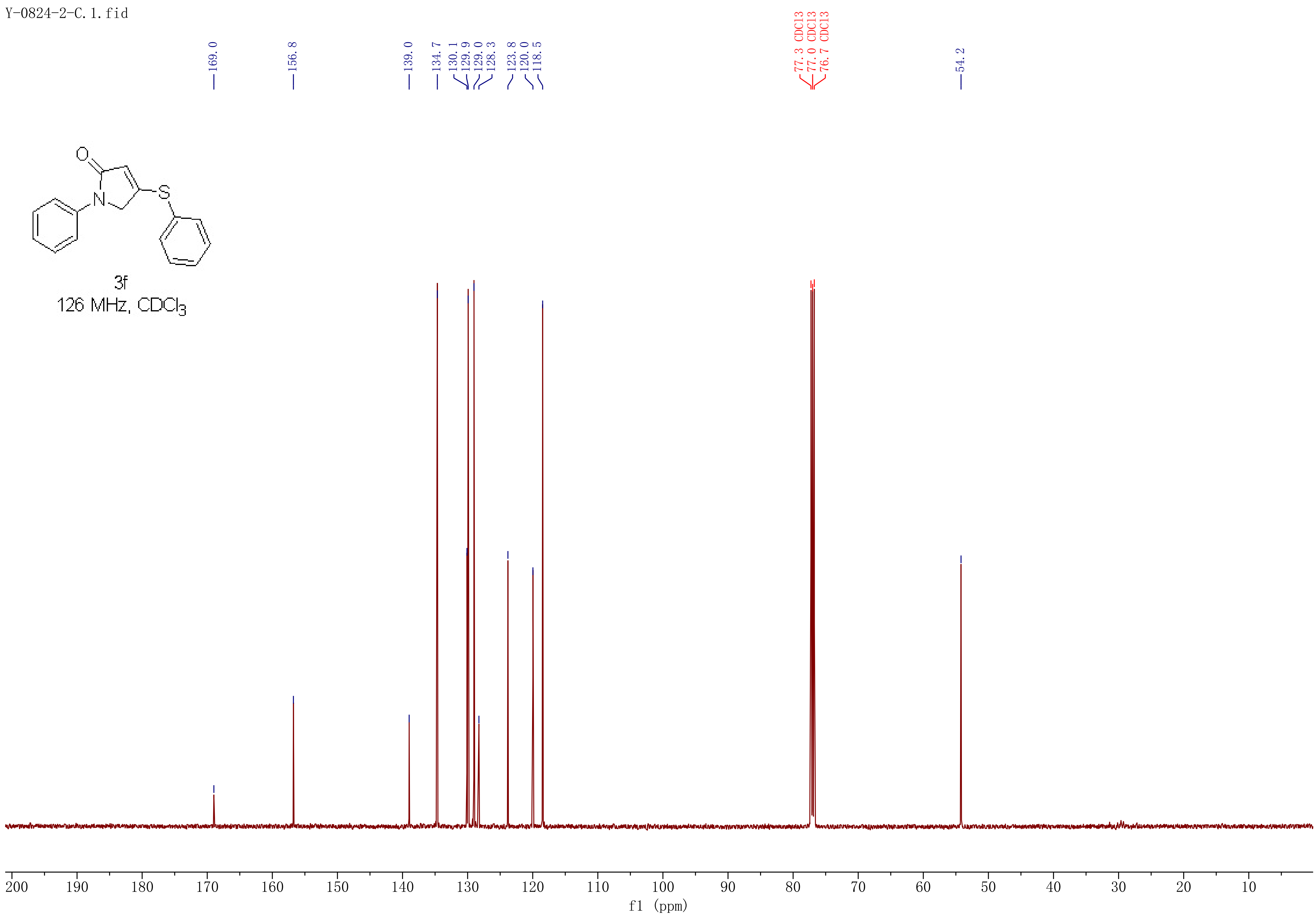
Y-0824-2-C.1.fid
169.0
156.8
139.0
134.7
130.1
129.9
129.0
128.3
123.8
120.0
118.5
77.3 CDCl3
77.0 CDCl3
76.7 CDCl3
54.2
O
N
S
3f
126 MHz, $CDCl_3$
200
190
180
170
160
150
140
130
120
110
100
90
80
70
60
50
40
30
20
10
f1 (ppm)

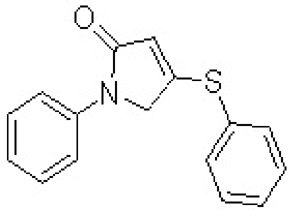

Qualitative Compound Identification Report

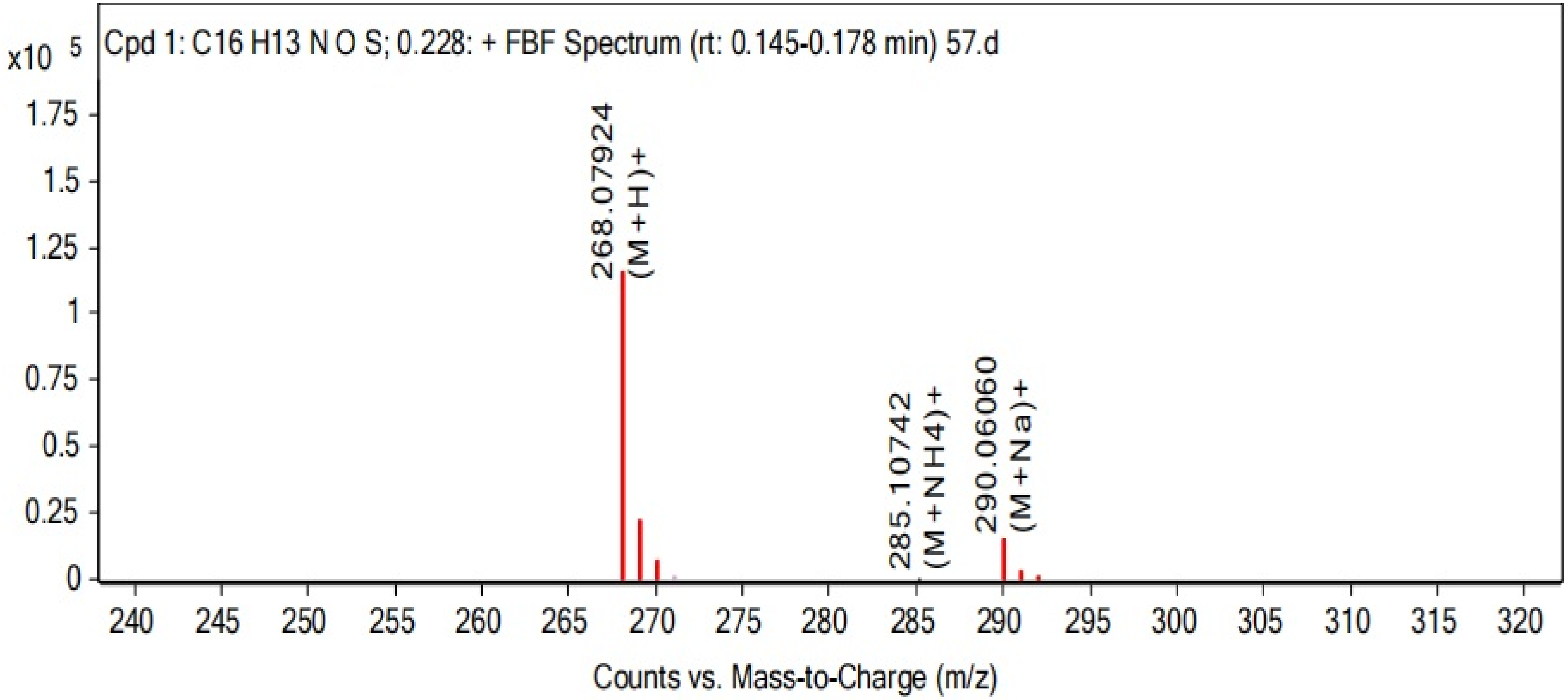

MS Spectrum Peak List

m/z	z	Abund	Ion
268.07924	1	115827.25	(M+H)+
269.08253	1	22165.59	(M+H)+
270.07519	1	6289.65	(M+H)+
285.10742	1	269.97	(M+NH4)+
290.0606	1	14821.95	(M+Na)+
291.06479	1	2439.54	(M+Na)+
292.05652	1	899.84	(M+Na)+

MS Spectrum

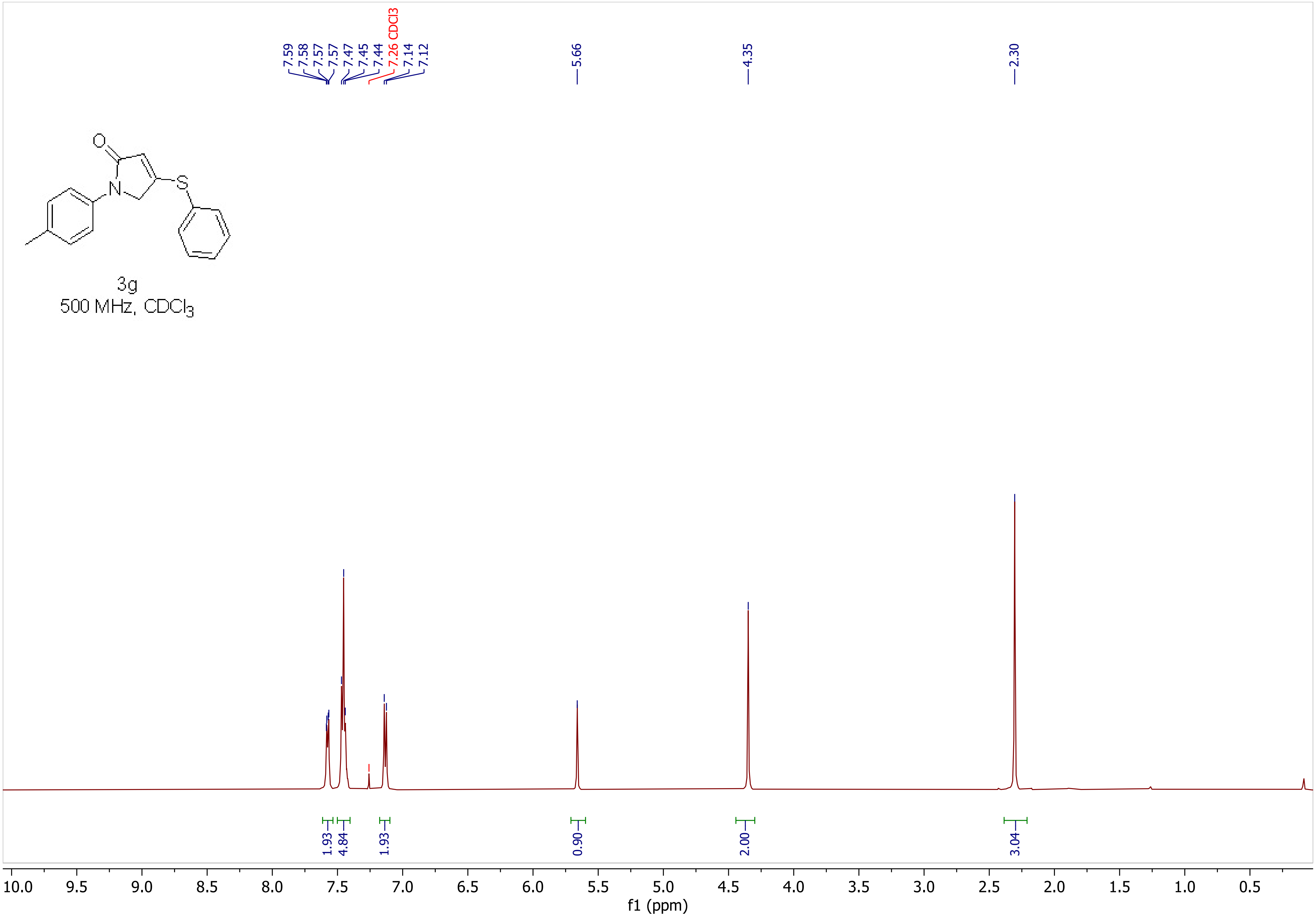
7.59
7.58
7.57
7.57
7.47
7.45
7.44
7.26 CDCl3
7.14
7.12
5.66
4.35
2.30
O
N
S
3g
500 MHz, CDCl$_3$
1.93
4.84
1.93
0.90
2.00
3.04
10.0
9.5
9.0
8.5
8.0
7.5
7.0
6.5
6.0
5.5
5.0
4.5
4.0
3.5
3.0
2.5
2.0
1.5
1.0
0.5
f1 (ppm)

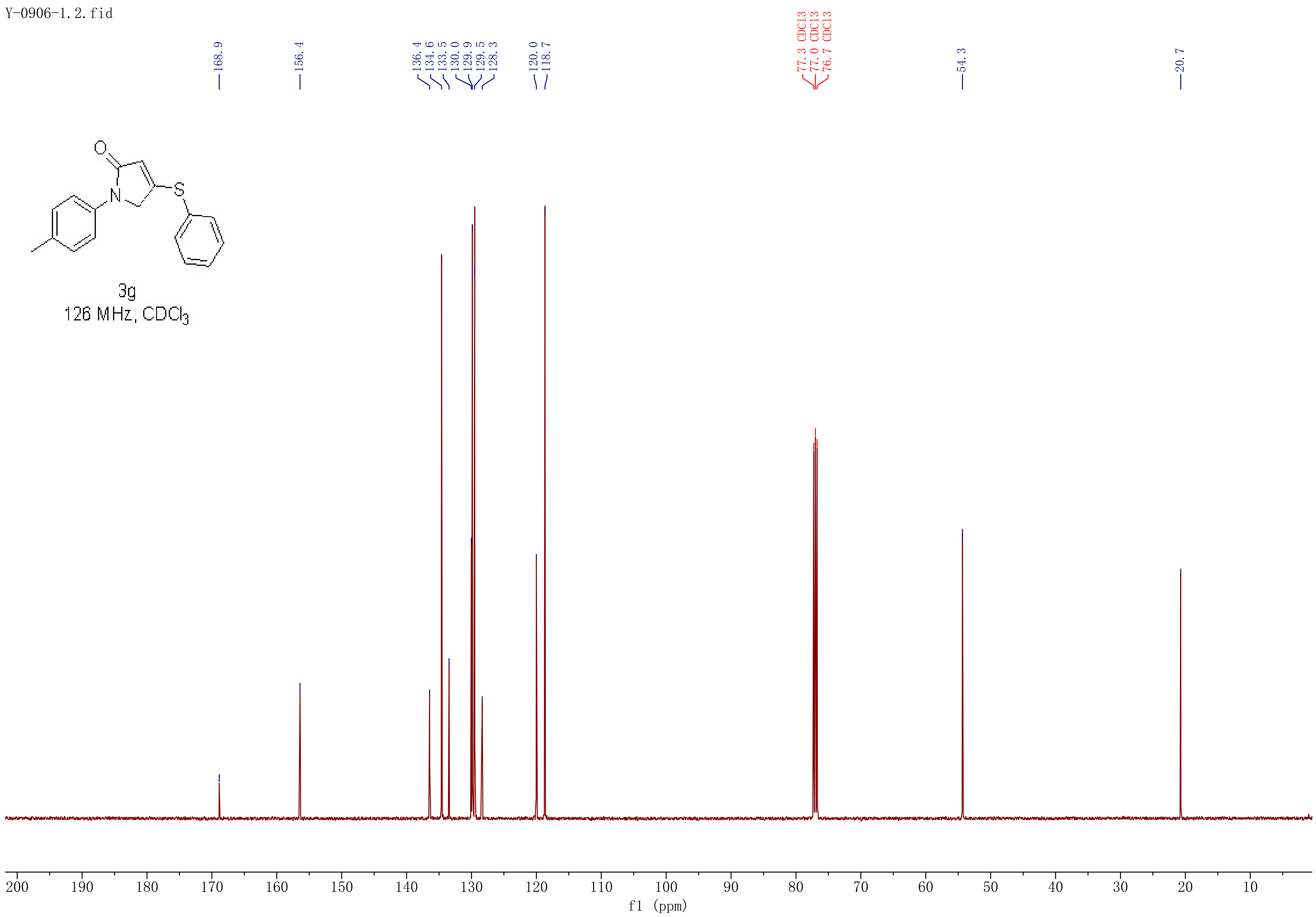

Y-0906-1.2.fid
168.9
156.4
136.4
134.6
133.5
130.0
129.9
129.5
128.3
120.0
118.7
77.3 CDCl3
77.0 CDCl3
76.7 CDCl3
54.3
20.7
O
N
S
3g
126 MHz, CDCl3
200
190
180
170
160
150
140
130
120
110
100
90
80
70
60
50
40
30
20
10
f1 (ppm)

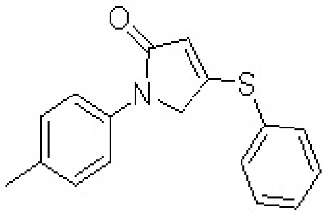

Qualitative Compound Identification Report

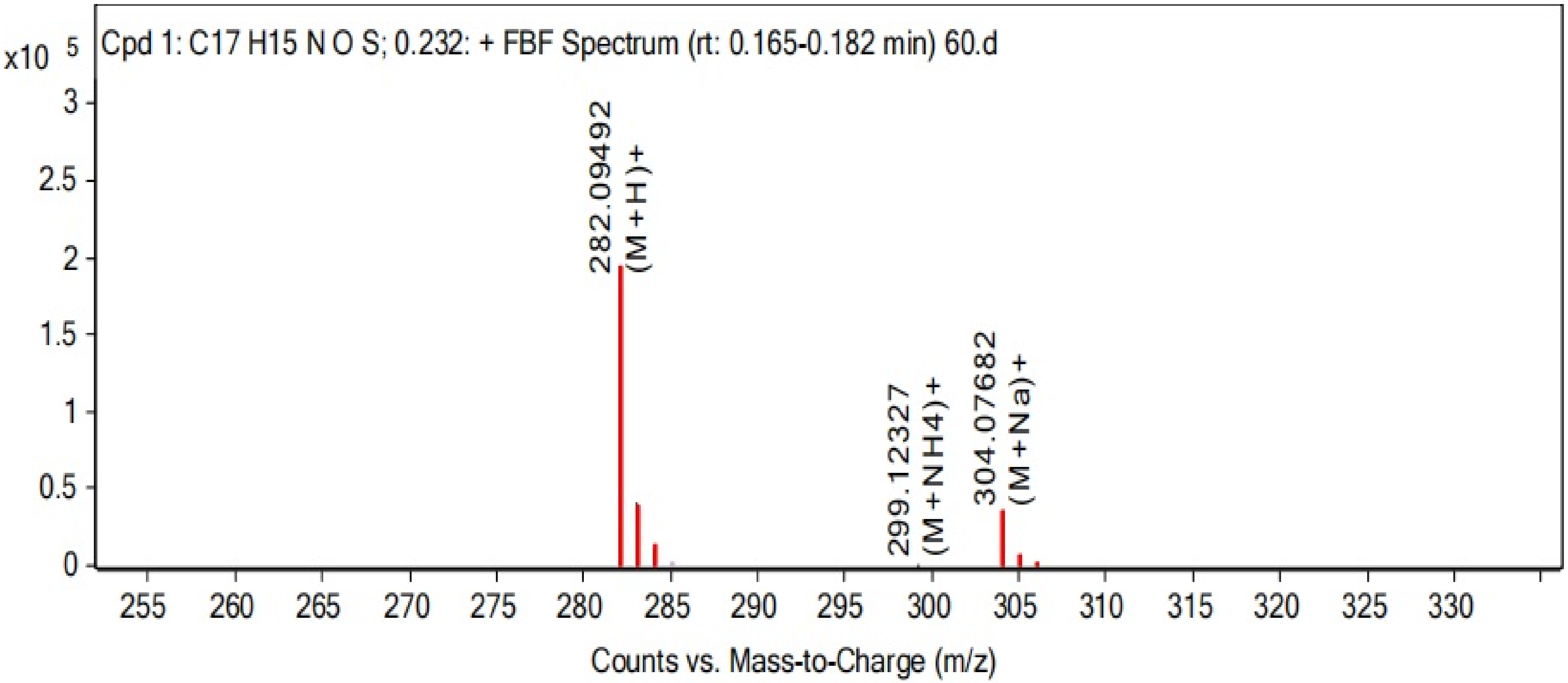

MS Spectrum Peak List

m/z	z	Abund	Ion
282.09492	1	194556.8	(M+H)+
283.09776	1	41113.02	(M+H)+
284.09038	1	10196.87	(M+H)+
299.12327	1	277.86	(M+NH4)+
304.07682	1	35375.48	(M+Na)+
305.0798	1	6052.29	(M+Na)+
306.0735	1	1406.23	(M+Na)+

MS Spectrum

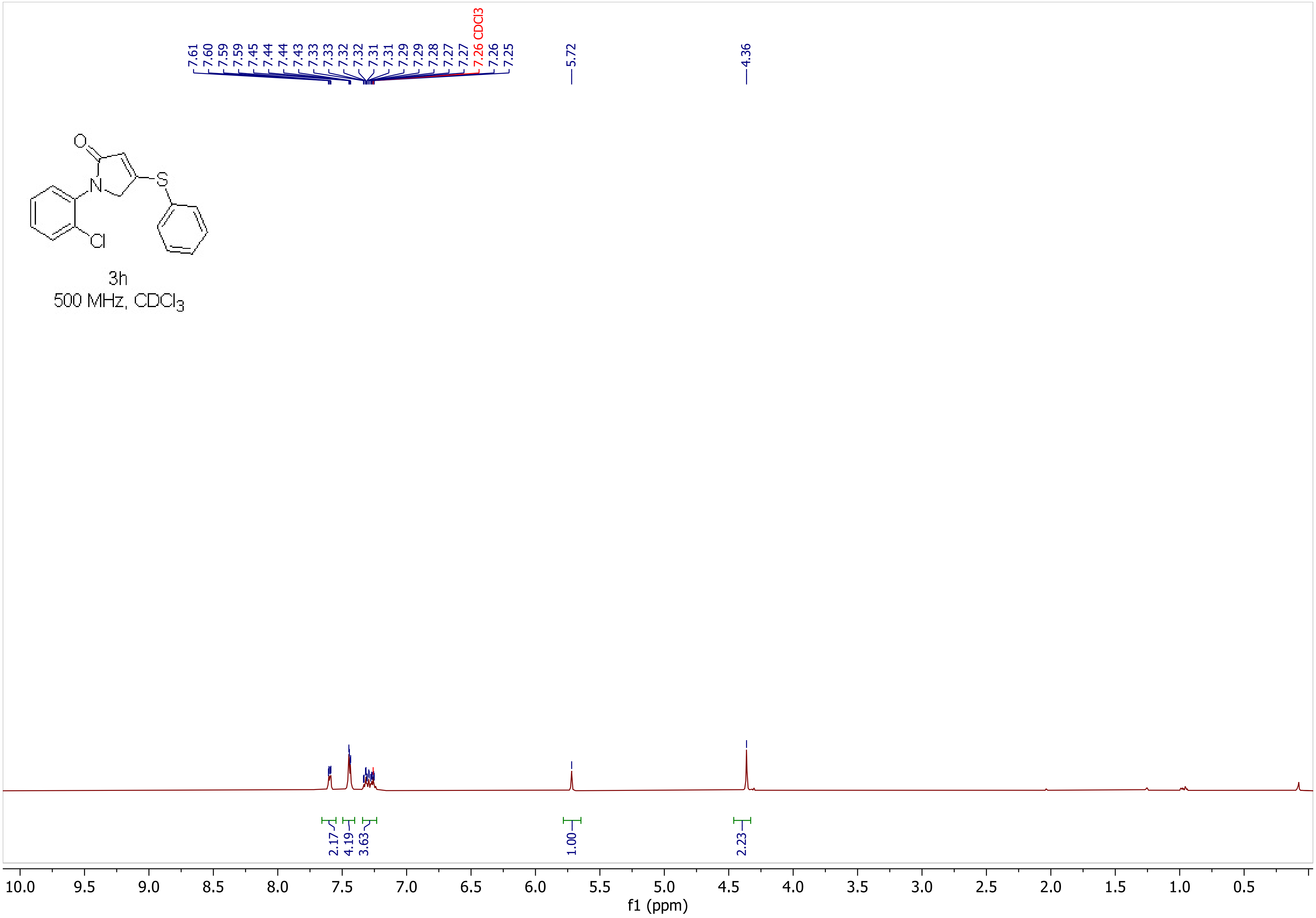
7.61
7.60
7.59
7.59
7.45
7.44
7.44
7.43
7.33
7.33
7.32
7.32
7.31
7.31
7.29
7.29
7.28
7.27
7.27
7.26 CDCl3
7.26
7.25
5.72
4.36
3h
500 MHz, CDCl3
2.17
4.19
3.63
1.00
2.23
10.0
9.5
9.0
8.5
8.0
7.5
7.0
6.5
6.0
5.5
5.0
4.5
4.0
3.5
3.0
2.5
2.0
1.5
1.0
0.5
f1 (ppm)

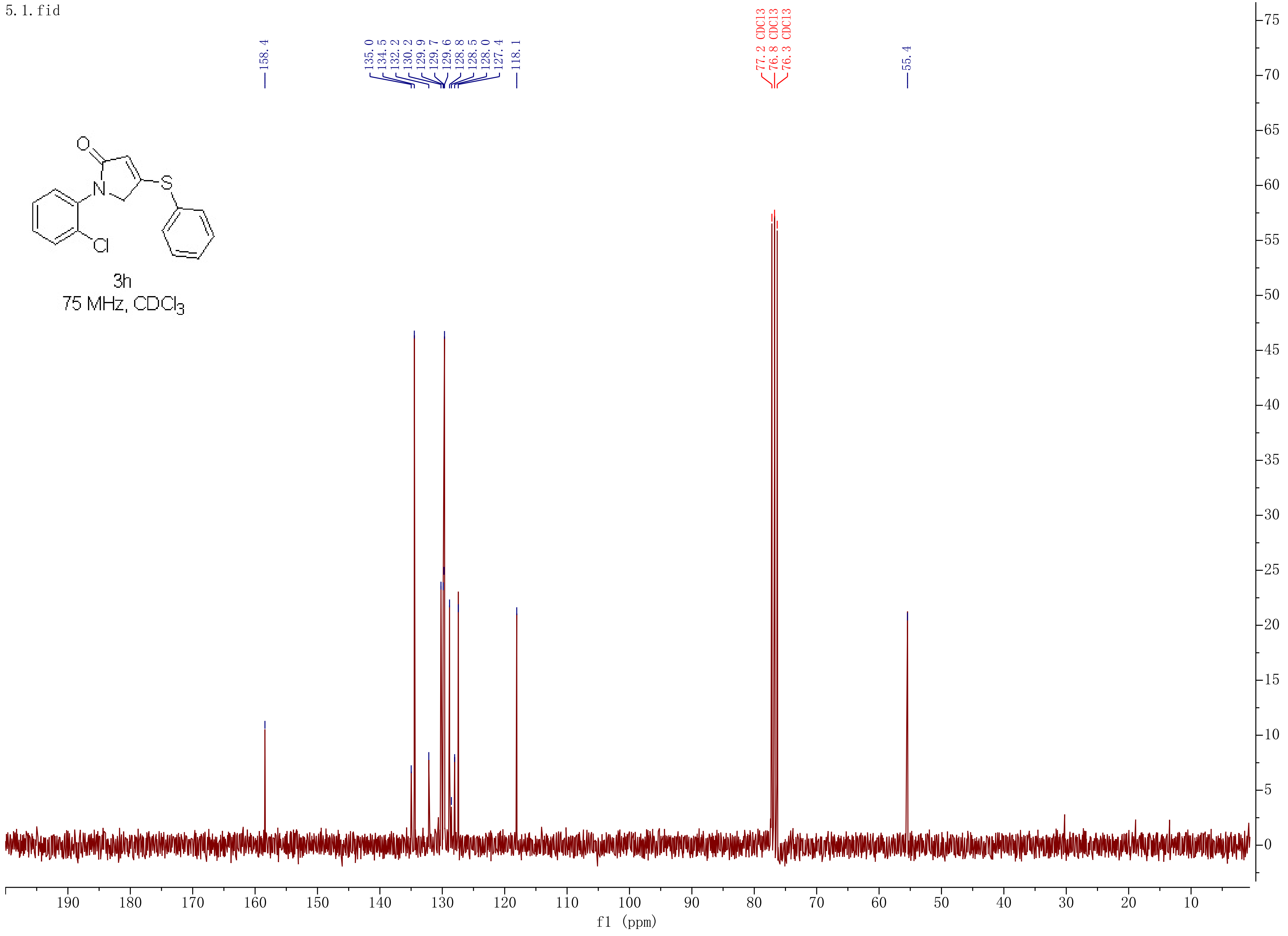

5.1.fid
158.4
135.0
134.5
132.2
130.2
129.9
129.7
129.6
128.8
128.5
128.0
127.4
118.1
77.2 CDCl3
76.8 CDCl3
76.3 CDCl3
55.4
O
N
S
Cl
3h
75 MHz, CDCl3
190 180 170 160 150 140 130 120 110 100 90 80 70 60 50 40 30 20 10
f1 (ppm)
75 70 65 60 55 50 45 40 35 30 25 20 15 10 5 0

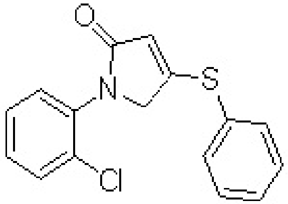

Qualitative Compound Identification Report

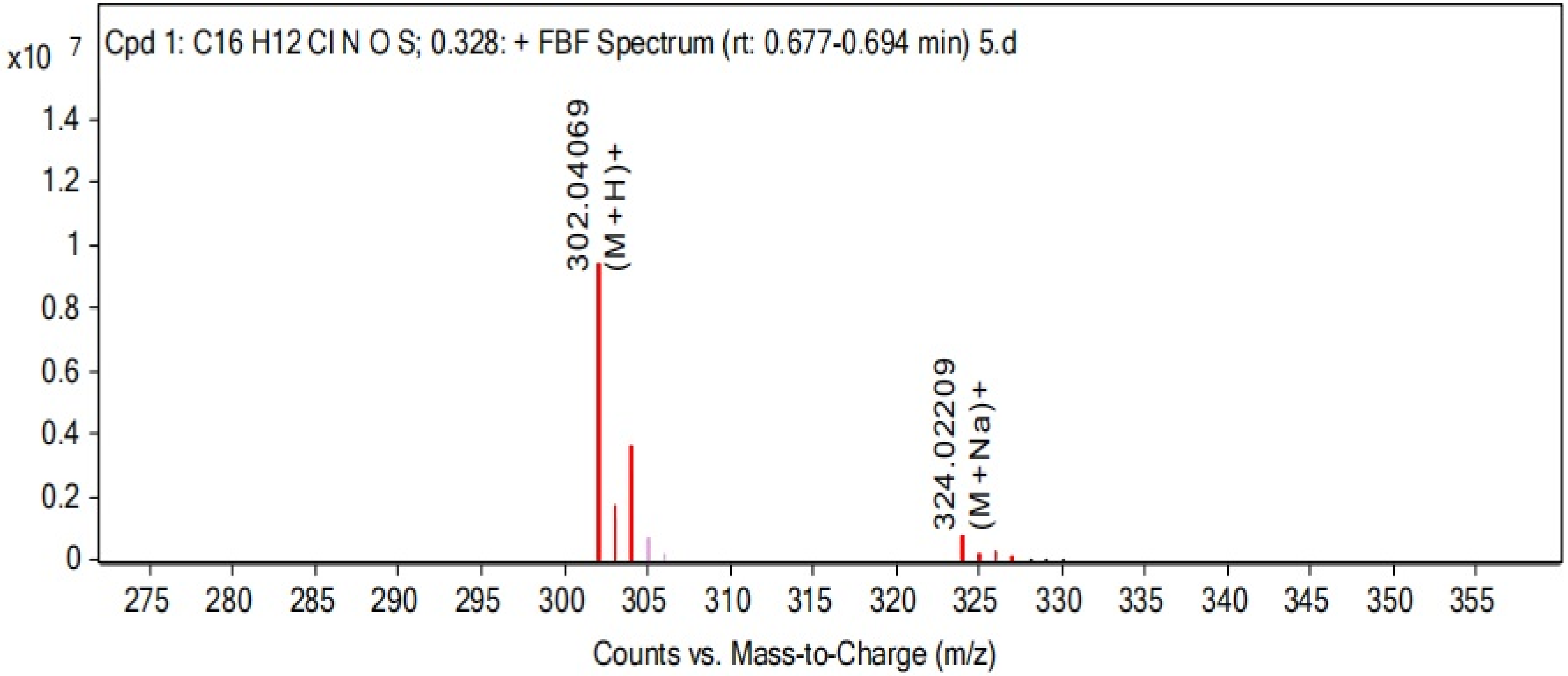

MS Spectrum Peak List

m/z	z	Abund	Ion
302.04069	1	9425690	(M+H)+
303.04339	1	1278191.5	(M+H)+
304.03727	1	2804521.75	(M+H)+
324.02209	1	712817.88	(M+Na)+
325.02536	1	134004.75	(M+Na)+
326.01945	1	271356.06	(M+Na)+
327.02241	1	51010.38	(M+Na)+
328.0151	1	15471.57	(M+Na)+
329.01199	1	2840.4	(M+Na)+
329.99918	1	7810.94	(M+Na)+

MS Spectrum

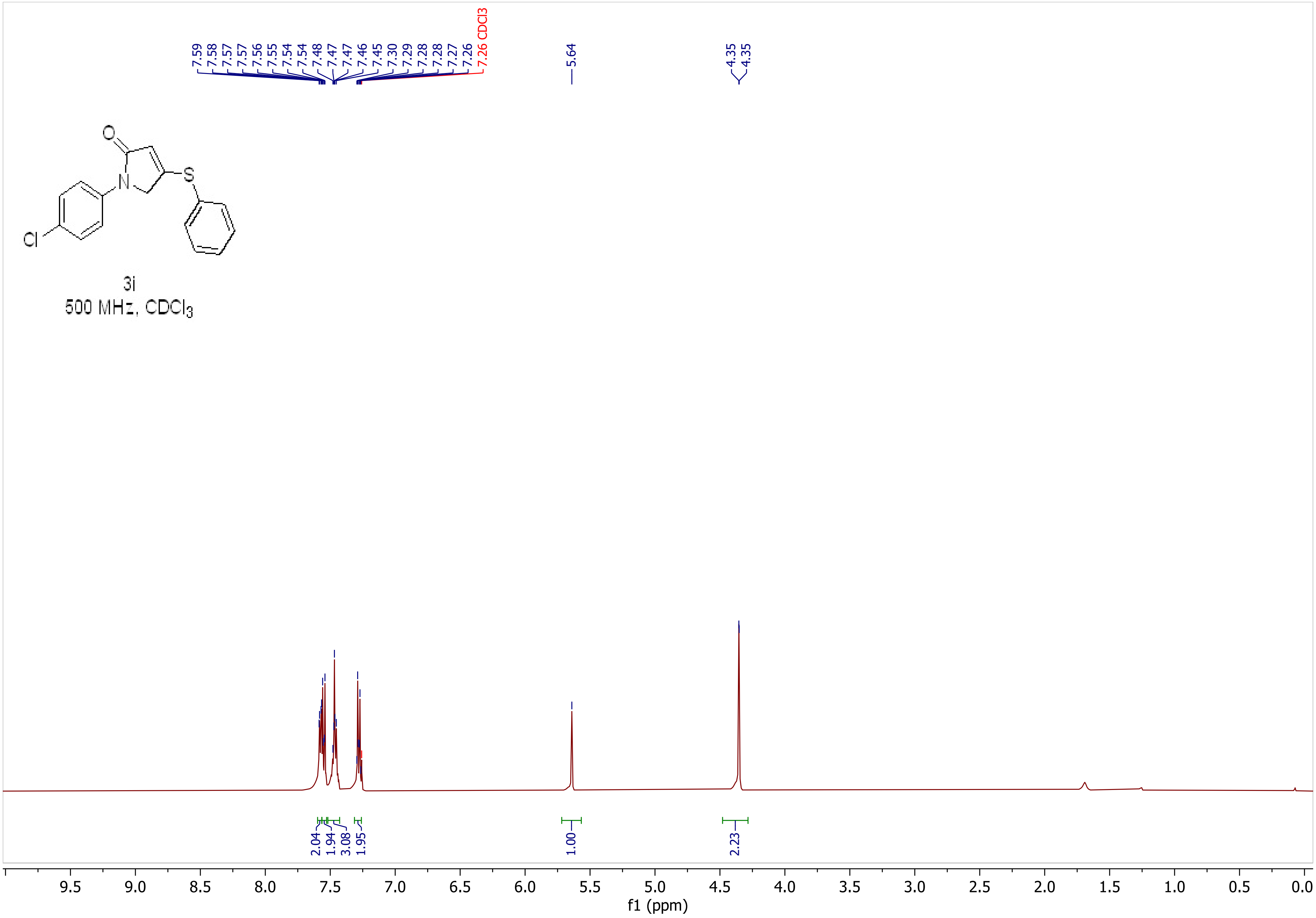
7.59
7.58
7.57
7.57
7.56
7.55
7.54
7.54
7.48
7.47
7.47
7.46
7.45
7.30
7.29
7.28
7.28
7.27
7.26
7.26 CDCl3
5.64
4.35
4.35
O
N
S
Cl
3i
500 MHz, CDCl3
2.04
1.94
3.08
1.95
1.00
2.23
9.5
9.0
8.5
8.0
7.5
7.0
6.5
6.0
5.5
5.0
4.5
4.0
3.5
3.0
2.5
2.0
1.5
1.0
0.5
0.0
f1 (ppm)

Y-0906-2.2.fid

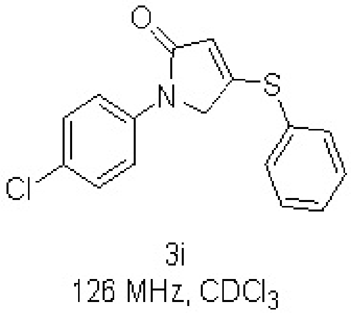
O
N
S
Cl
3i
126 MHz, CDCl3

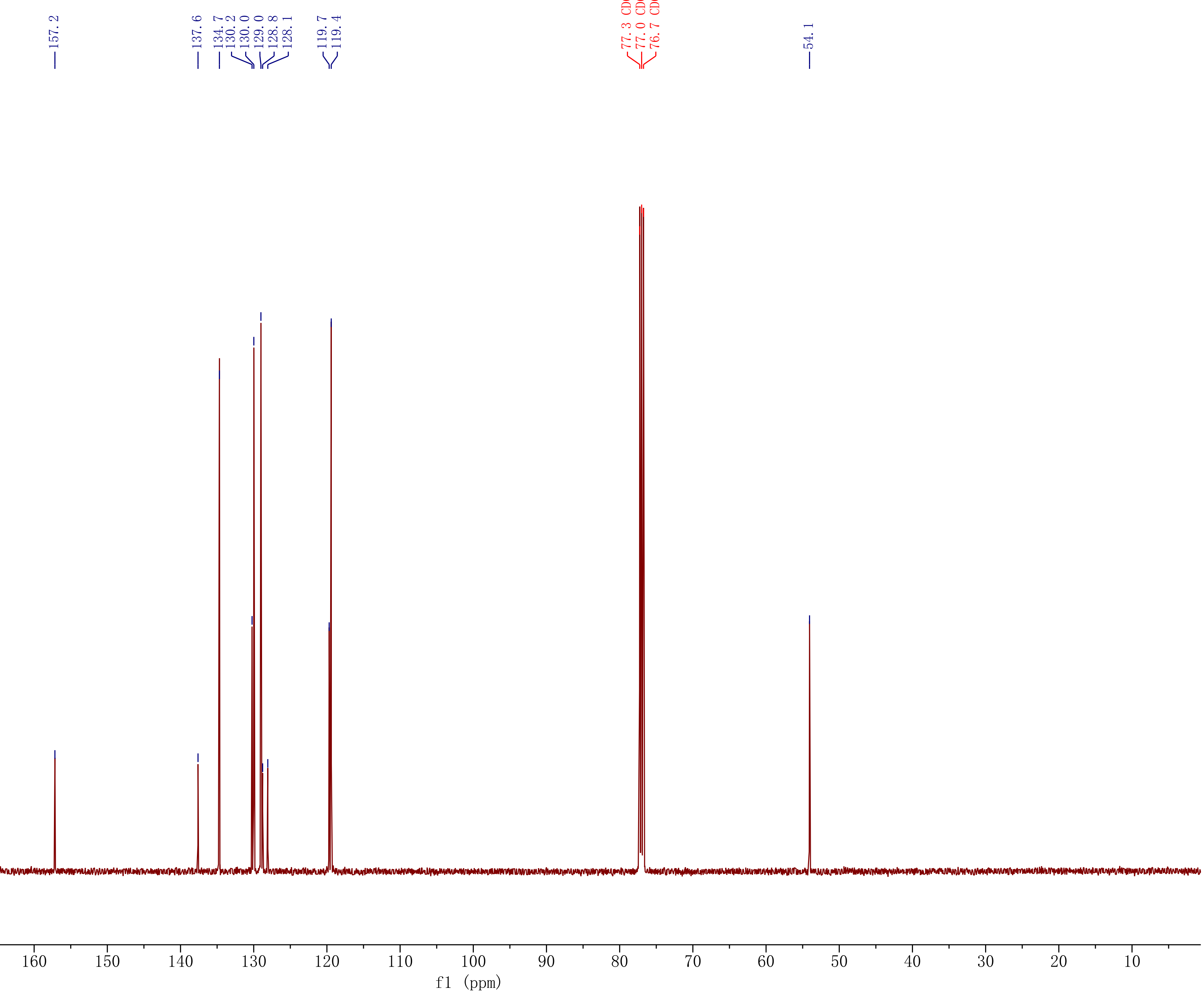
168.9
157.2
137.6
134.7
130.2
130.0
129.0
128.8
128.1
119.7
119.4
77.3 CDCl3
77.0 CDCl3
76.7 CDCl3
54.1
190
180
170
160
150
140
130
120
110
100
90
80
70
60
50
40
30
20
10
f1 (ppm)

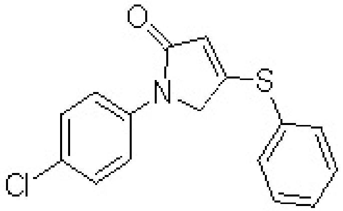

Qualitative Compound Identification Report

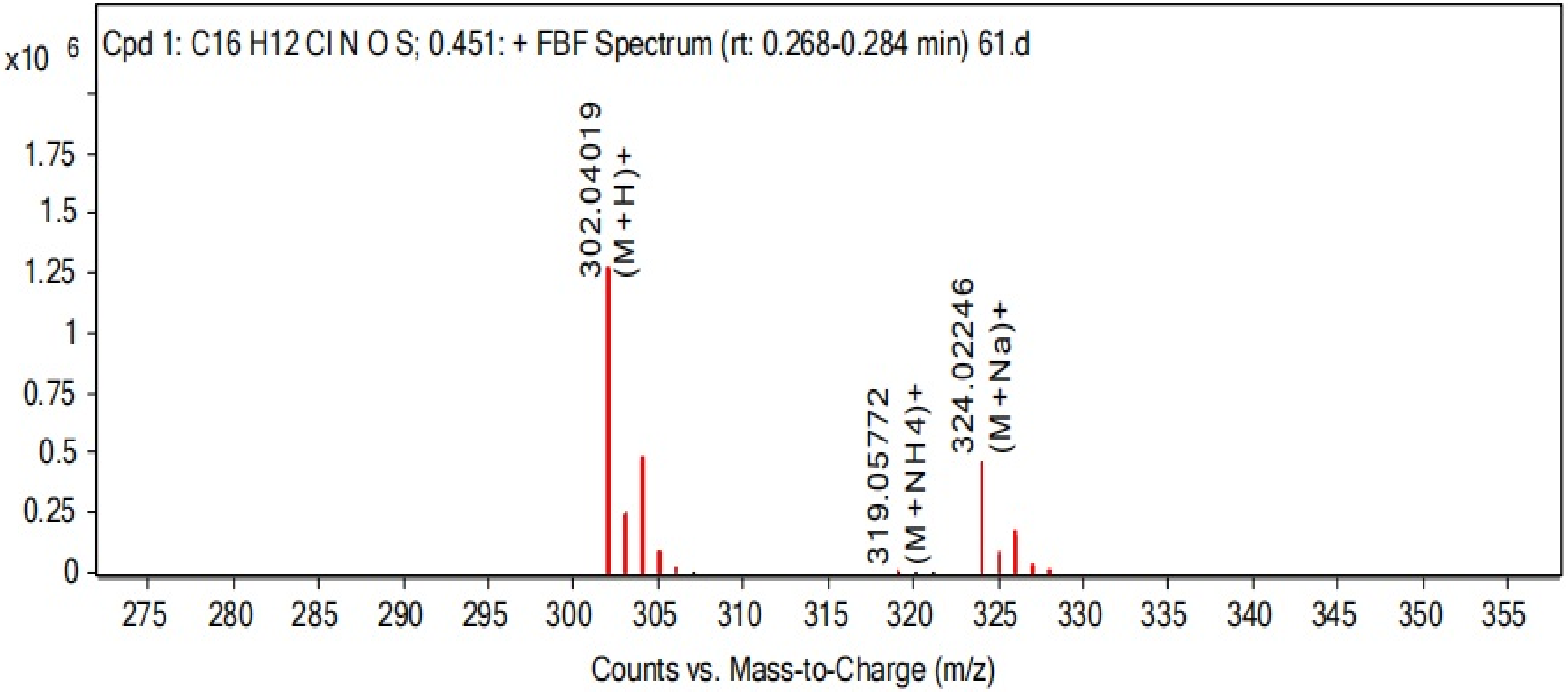

MS Spectrum Peak List

m/z	z	Abund	Ion
302.04019	1	1265754.38	(M+H)+
303.04308	1	254232.48	(M+H)+
304.03675	1	467150.13	(M+H)+
305.04039	1	92736.64	(M+H)+
306.03293	1	24591.56	(M+H)+
324.02246	1	464224.06	(M+Na)+
325.02494	1	76630.25	(M+Na)+
326.01885	1	150578.59	(M+Na)+
327.02205	1	32932.18	(M+Na)+
328.01801	1	13631.9	(M+Na)+

MS Spectrum

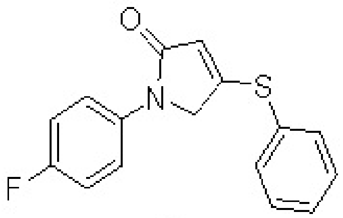

3j
500 MHz, $CDCl_3$

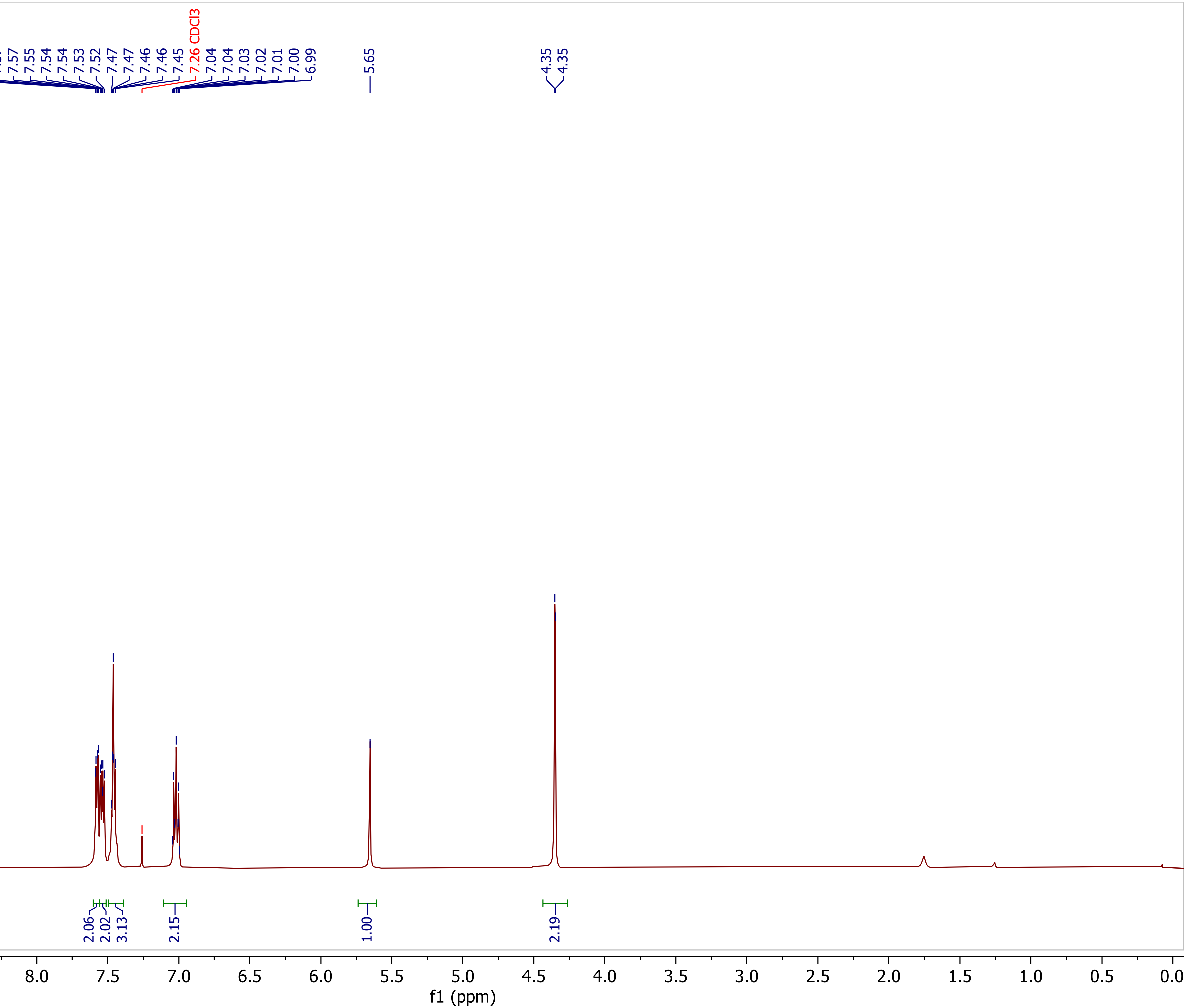

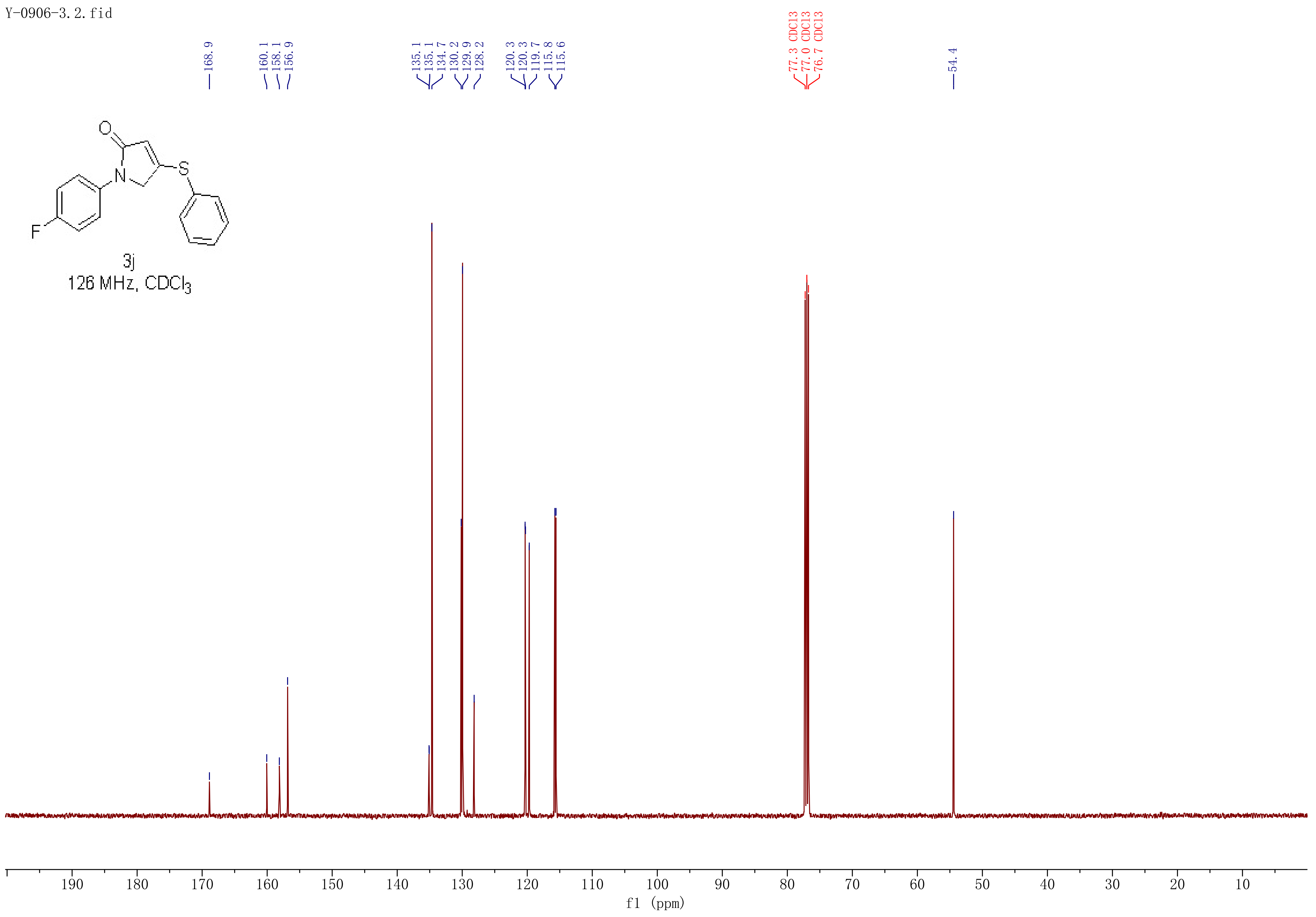

Y-0906-3.2.fid
168.9
160.1
158.1
156.9
135.1
135.1
134.7
130.2
129.9
128.2
120.3
120.3
119.7
115.8
115.6
77.3 CDCl3
77.0 CDCl3
76.7 CDCl3
54.4
O
N
S
F
3j
126 MHz, CDCl3
190
180
170
160
150
140
130
120
110
100
90
80
70
60
50
40
30
20
10
f1 (ppm)

Y-0906-3-F.1.fid
F19

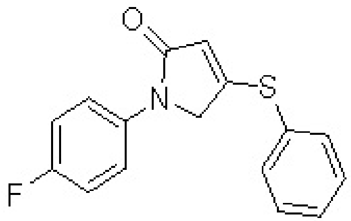

3j
471 MHz, $CDCl_3$

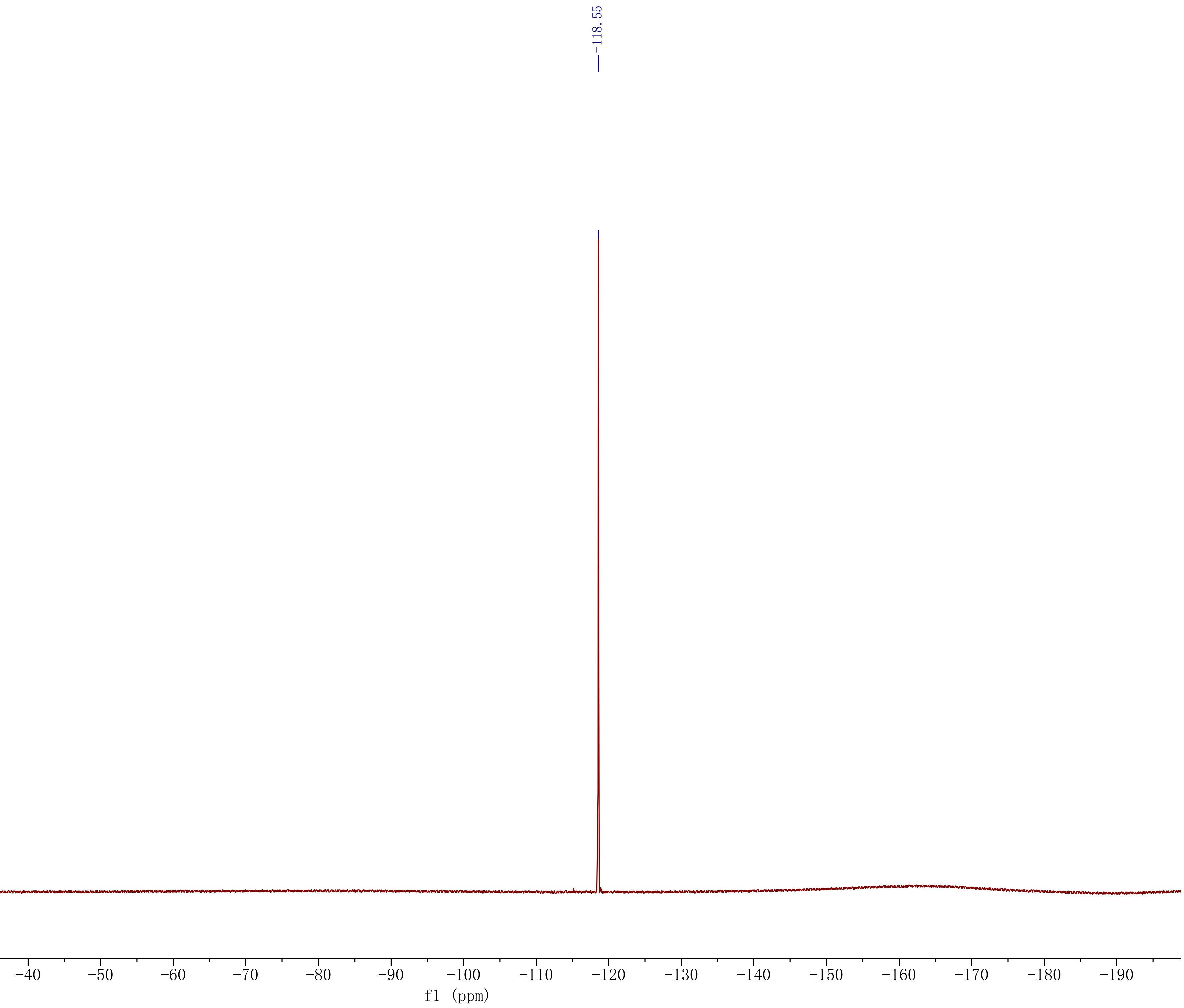

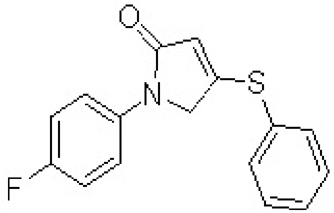

Qualitative Compound Identification Report

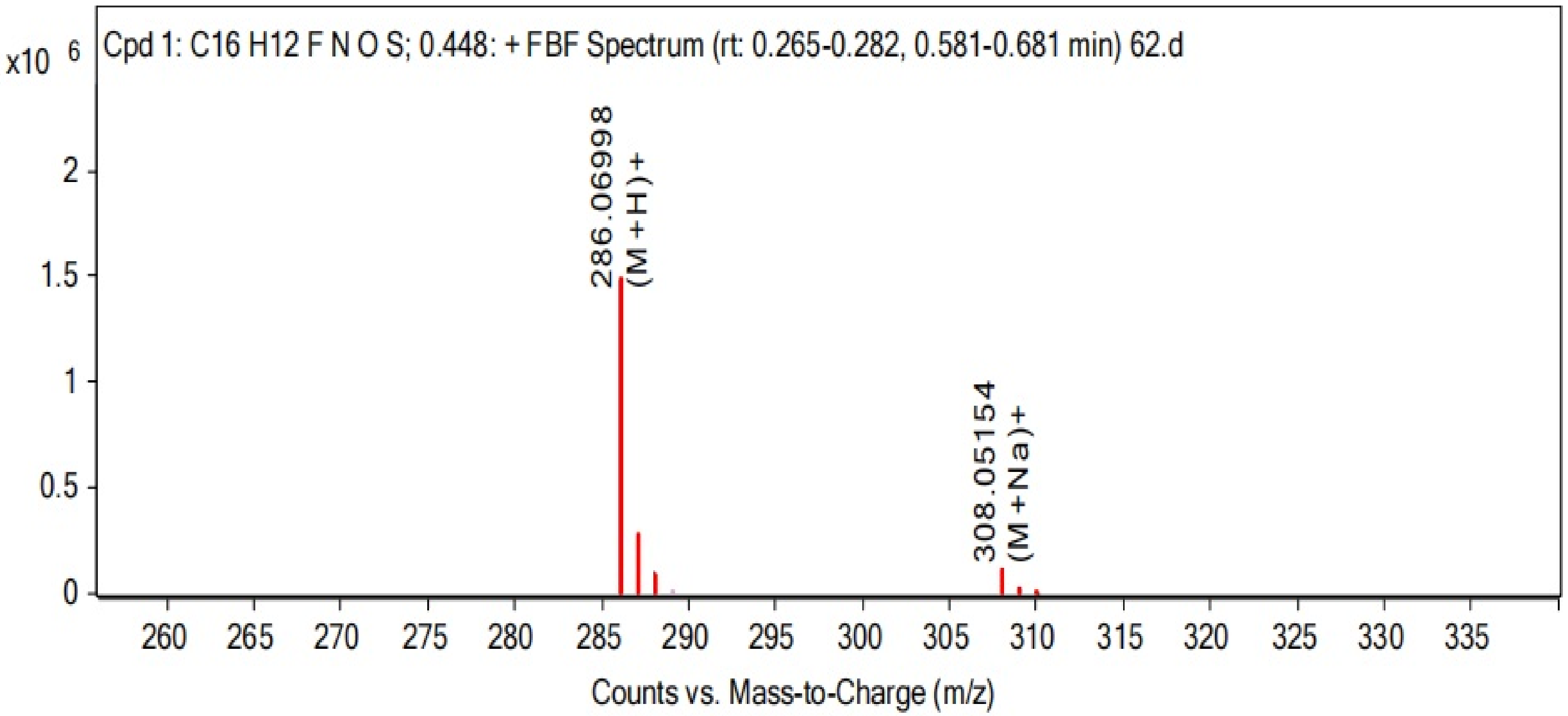

MS Spectrum Peak List

m/z	z	Abund	Ion
286.06998	1	1484045.38	(M+H)+
287.07324	1	273602.81	(M+H)+
288.06884	1	106315.73	(M+H)+
308.05154	1	115106.38	(M+Na)+
309.05452	1	22793.12	(M+Na)+
310.04769	1	6244.43	(M+Na)+

MS Spectrum

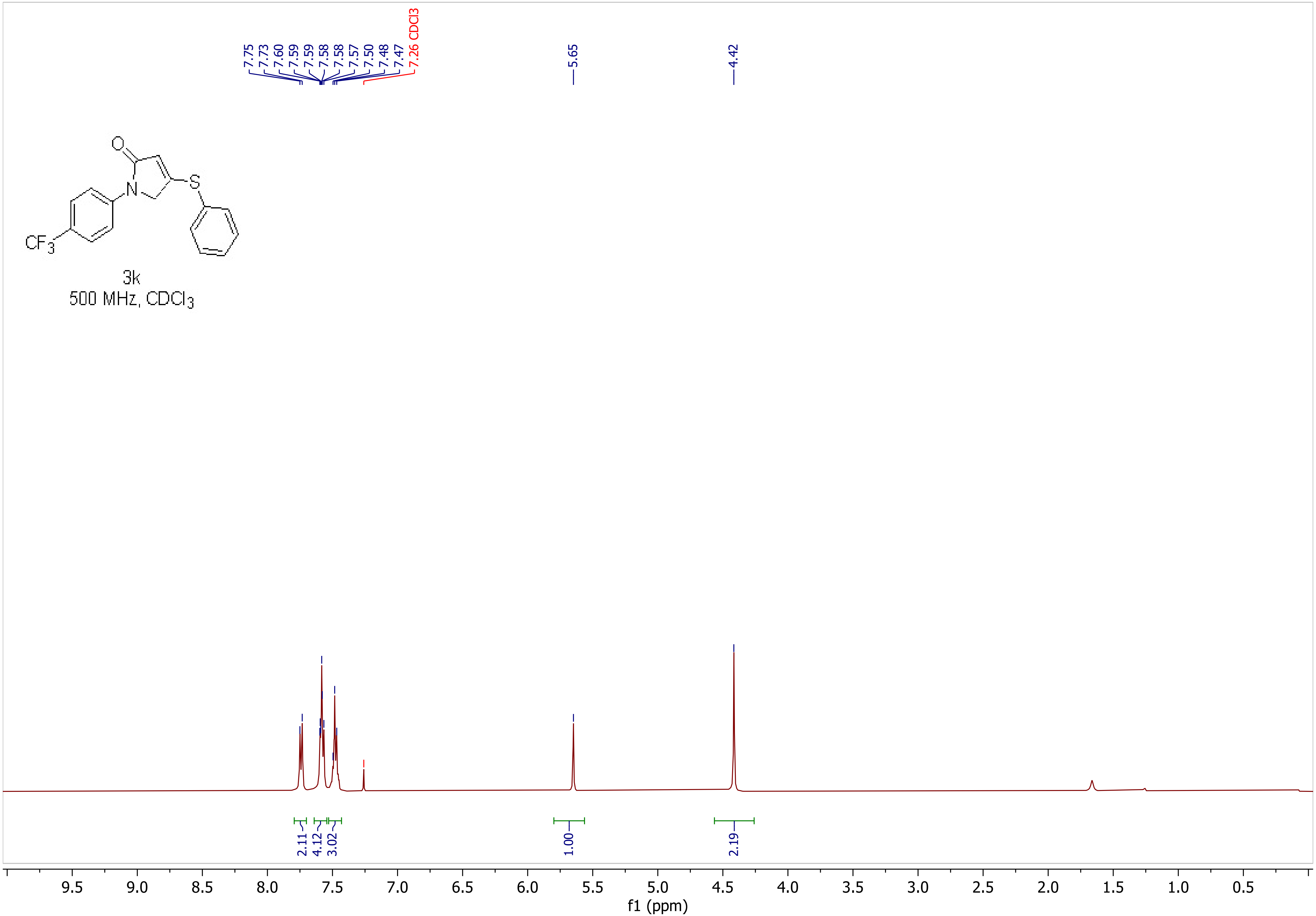

7.75
7.73
7.60
7.59
7.59
7.58
7.58
7.57
7.50
7.48
7.47
7.26 CDCl3
5.65
4.42
3k
500 MHz, CDCl3
2.11
4.12
3.02
1.00
2.19
9.5
9.0
8.5
8.0
7.5
7.0
6.5
6.0
5.5
5.0
4.5
4.0
3.5
3.0
2.5
2.0
1.5
1.0
0.5
f1 (ppm)

Y-0906-4.2.fid

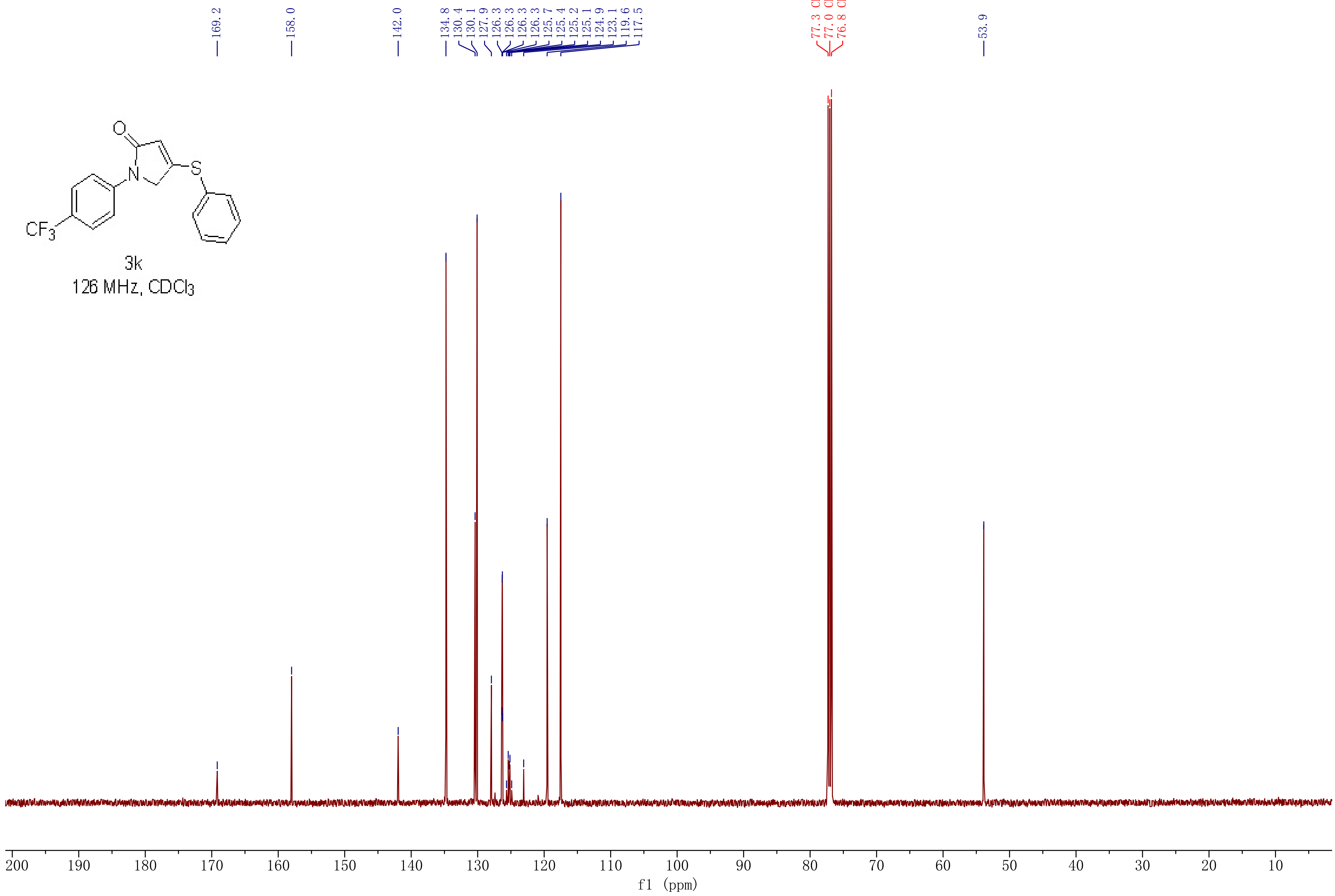
169.2
158.0
142.0
134.8
130.4
130.1
127.9
126.3
126.3
126.3
126.3
125.7
125.4
125.2
125.1
124.9
123.1
119.6
117.5
77.3 CDCl3
77.0 CDCl3
76.8 CDCl3
53.9
O
N
S
CF3
3k
126 MHz, CDCl3
200
190
180
170
160
150
140
130
120
110
100
90
80
70
60
50
40
30
20
10
f1 (ppm)

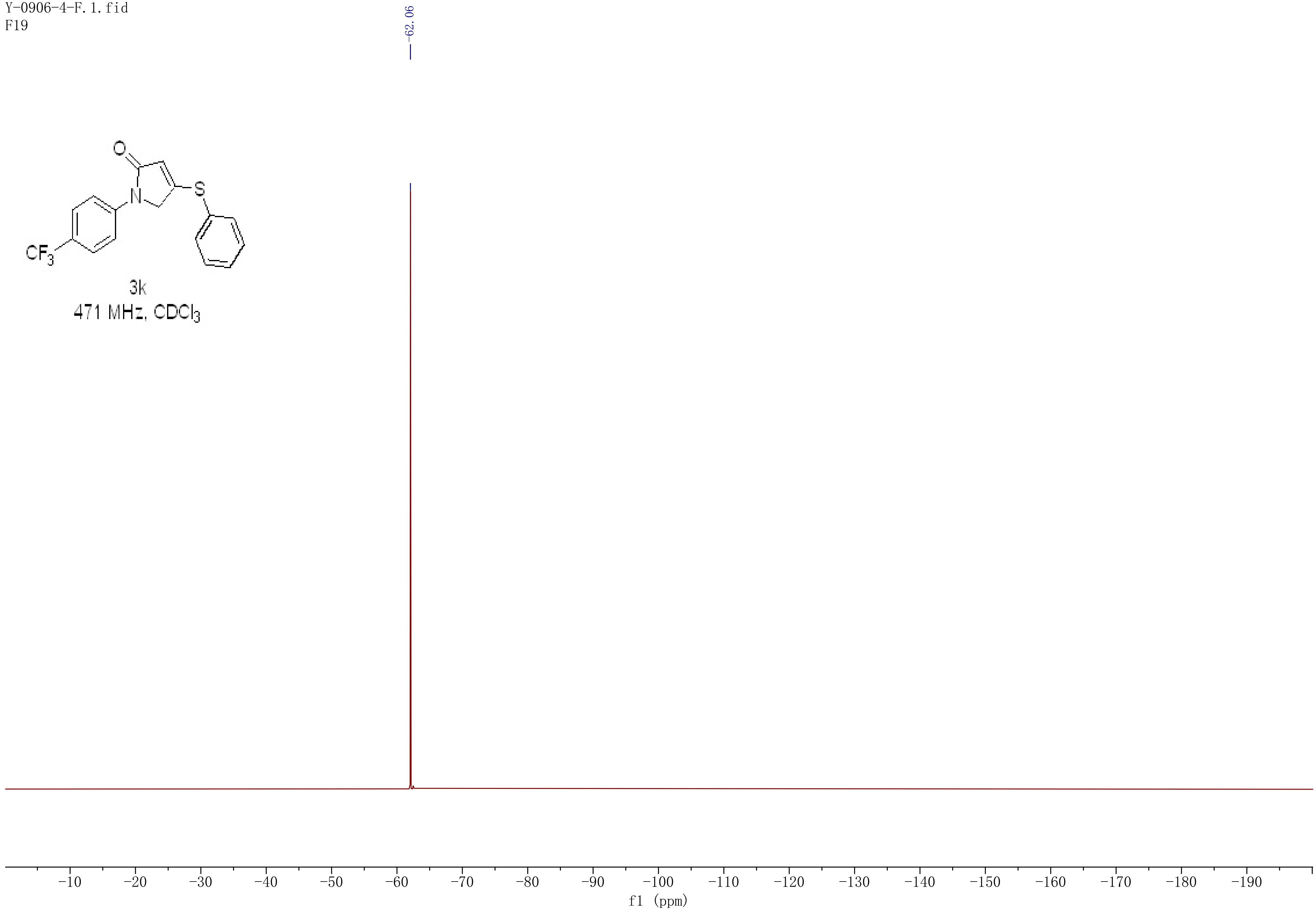
Y-0906-4-F.1.fid
F19
−62.06
CF_3
3k
471 MHz, $CDCl_3$
−10 −20 −30 −40 −50 −60 −70 −80 −90 −100 −110 −120 −130 −140 −150 −160 −170 −180 −190
f1 (ppm)

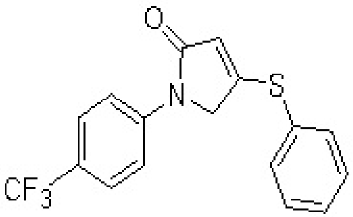

Qualitative Compound Identification Report

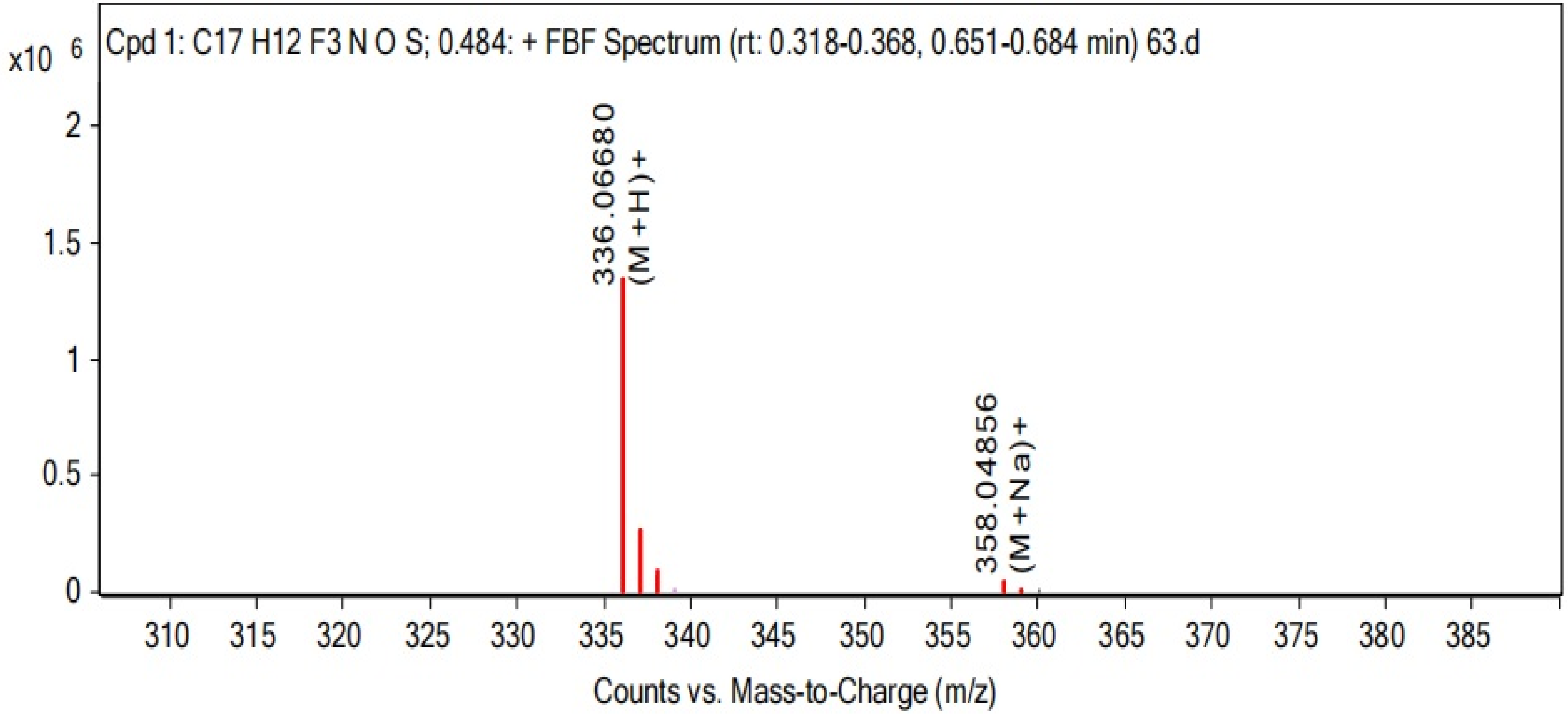

MS Spectrum Peak List

m/z	z	Abund	Ion
336.0668	1	1351915.88	(M+H)+
337.06969	1	262284.91	(M+H)+
338.06254	1	68971.39	(M+H)+
358.04856	1	43188.02	(M+Na)+
359.05198	1	7467.97	(M+Na)+
360.04952	1	7537.58	(M+Na)+

MS Spectrum

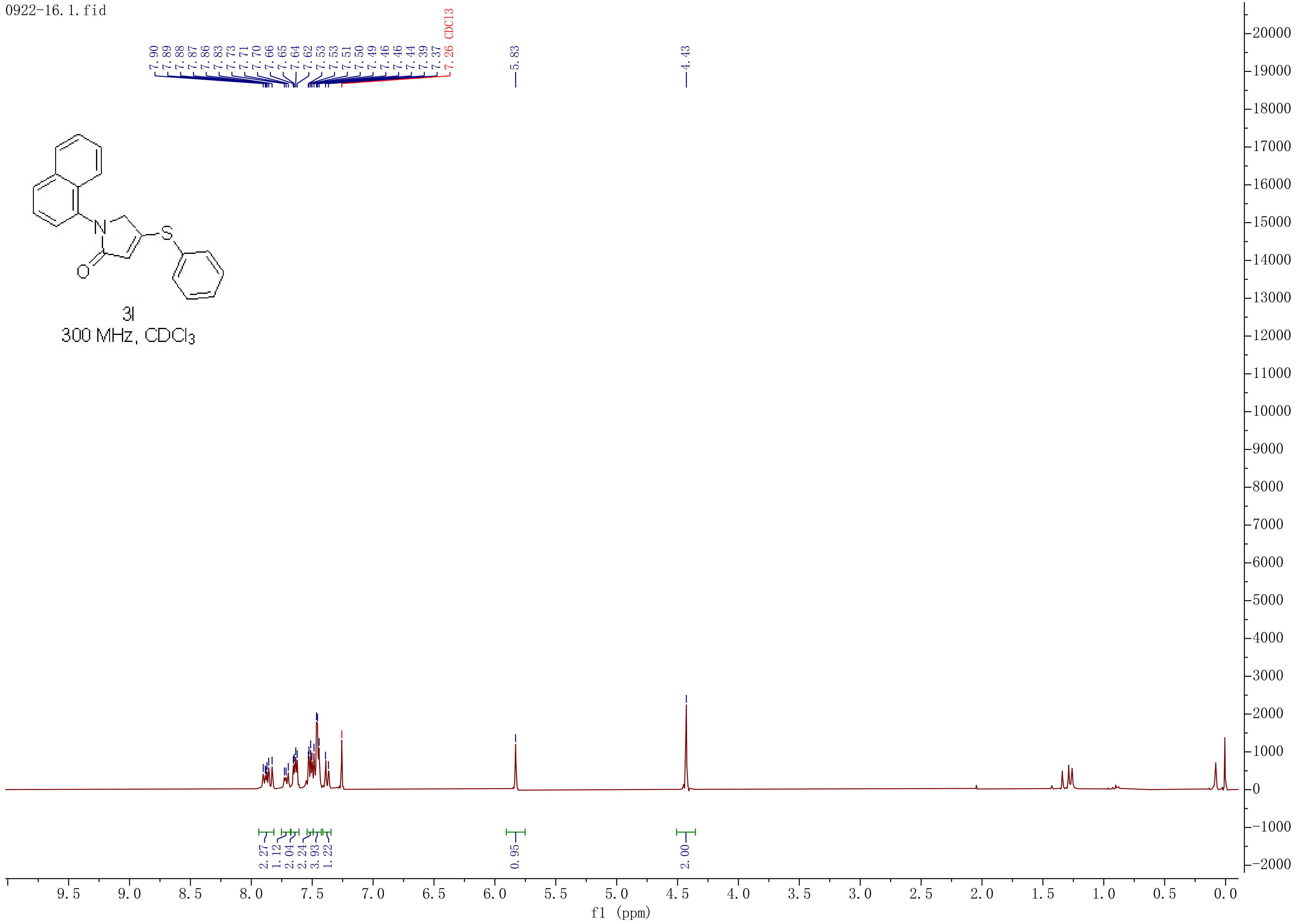

3l
300 MHz, $CDCl_3$

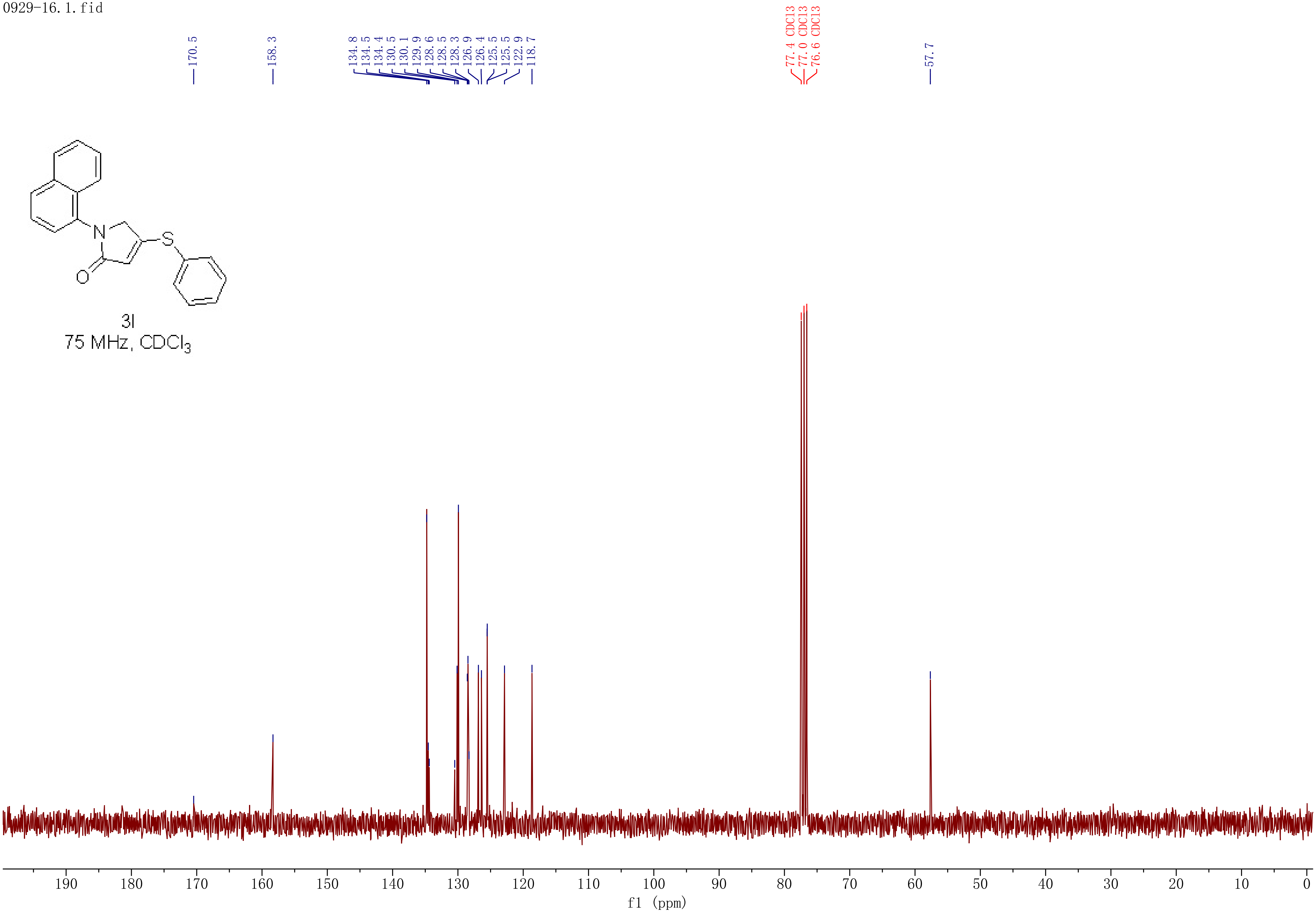
0929-16.1.fid
170.5
158.3
134.8
134.5
134.4
130.5
130.1
129.9
128.6
128.5
128.3
126.9
126.4
125.5
125.5
122.9
118.7
77.4 CDCl3
77.0 CDCl3
76.6 CDCl3
57.7
3l
75 MHz, CDCl3
f1 (ppm)

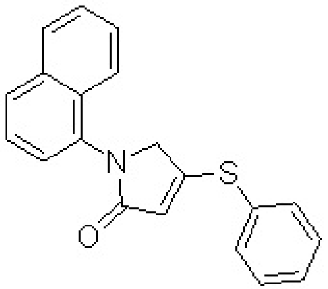

Qualitative Compound Identification Report

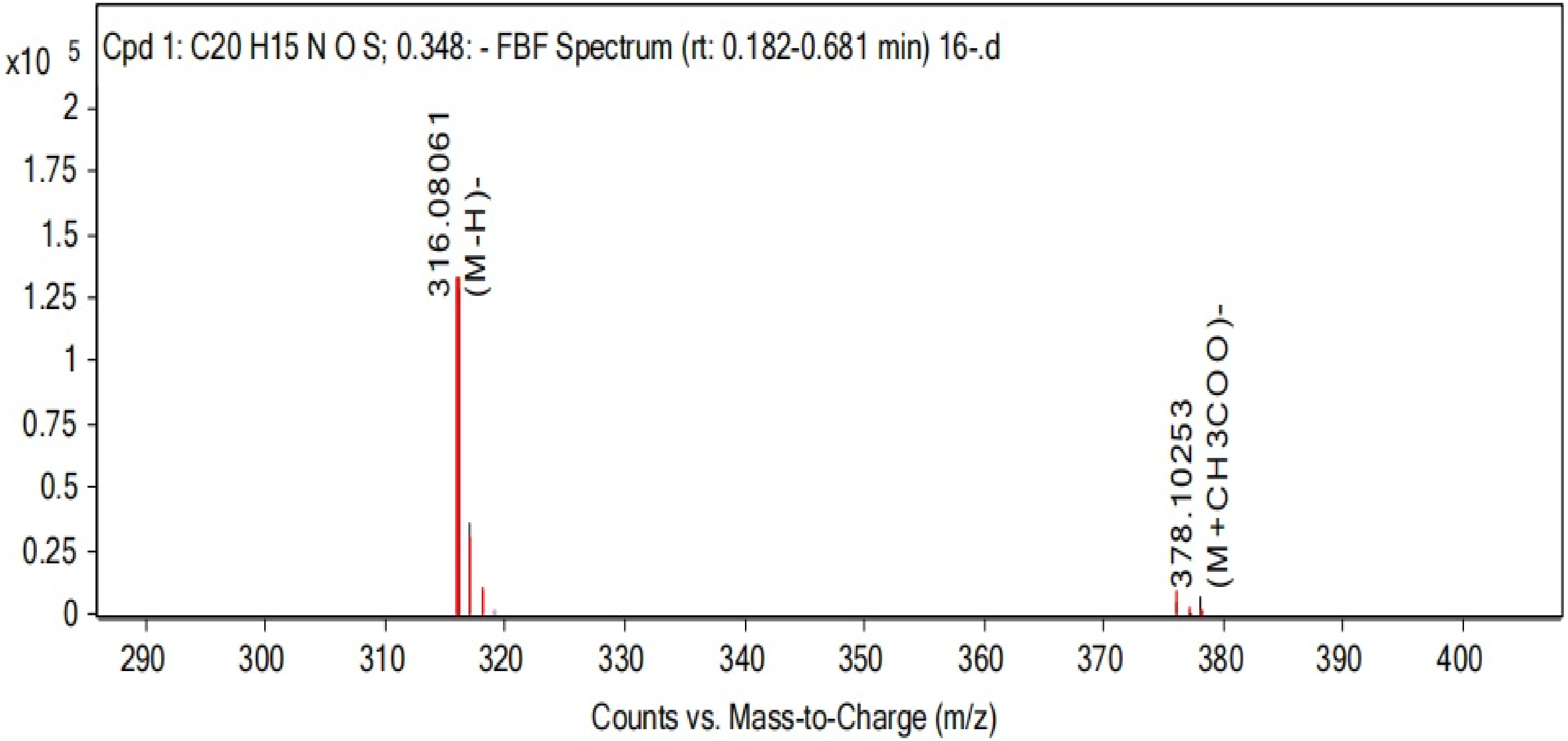

MS Spectrum Peak List

m/z	z	Abund	Ion
316.08061	1	128630.28	(M-H)-
317.084	1	36209.01	(M-H)-
318.07572	1	8484.57	(M-H)-
376.09769	1	5005.41	(M+CH3COO)-
378.10253	1	6819.1	(M+CH3COO)-

MS Spectrum

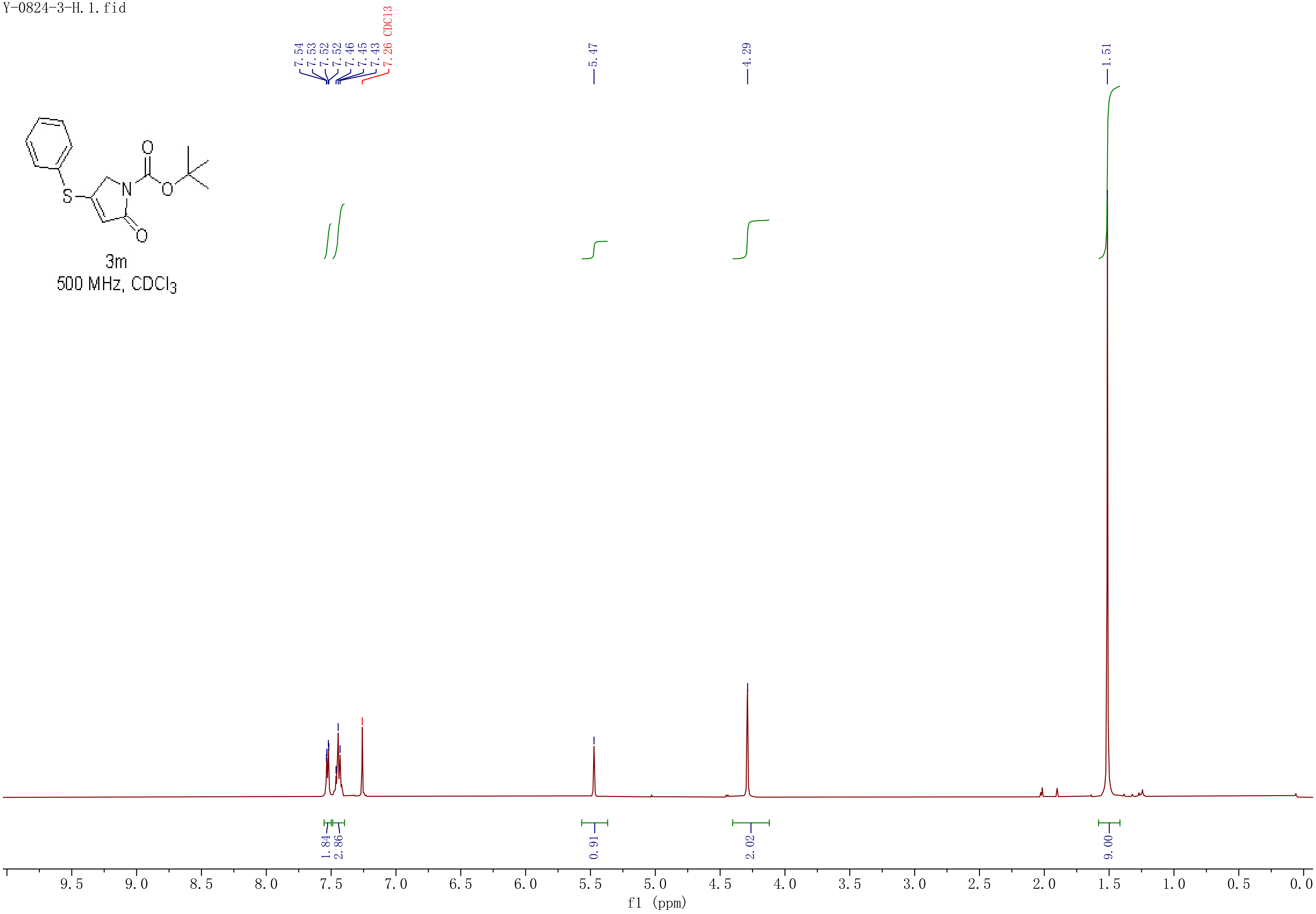
Y-0824-3-H.1.fid
7.54
7.53
7.52
7.52
7.46
7.45
7.43
7.26 CDCl3
5.47
4.29
1.51
S
N
O
O
O
3m
500 MHz, CDCl3
1.84
2.86
0.91
2.02
9.00
9.5
9.0
8.5
8.0
7.5
7.0
6.5
6.0
5.5
5.0
4.5
4.0
3.5
3.0
2.5
2.0
1.5
1.0
0.5
0.0
f1 (ppm)

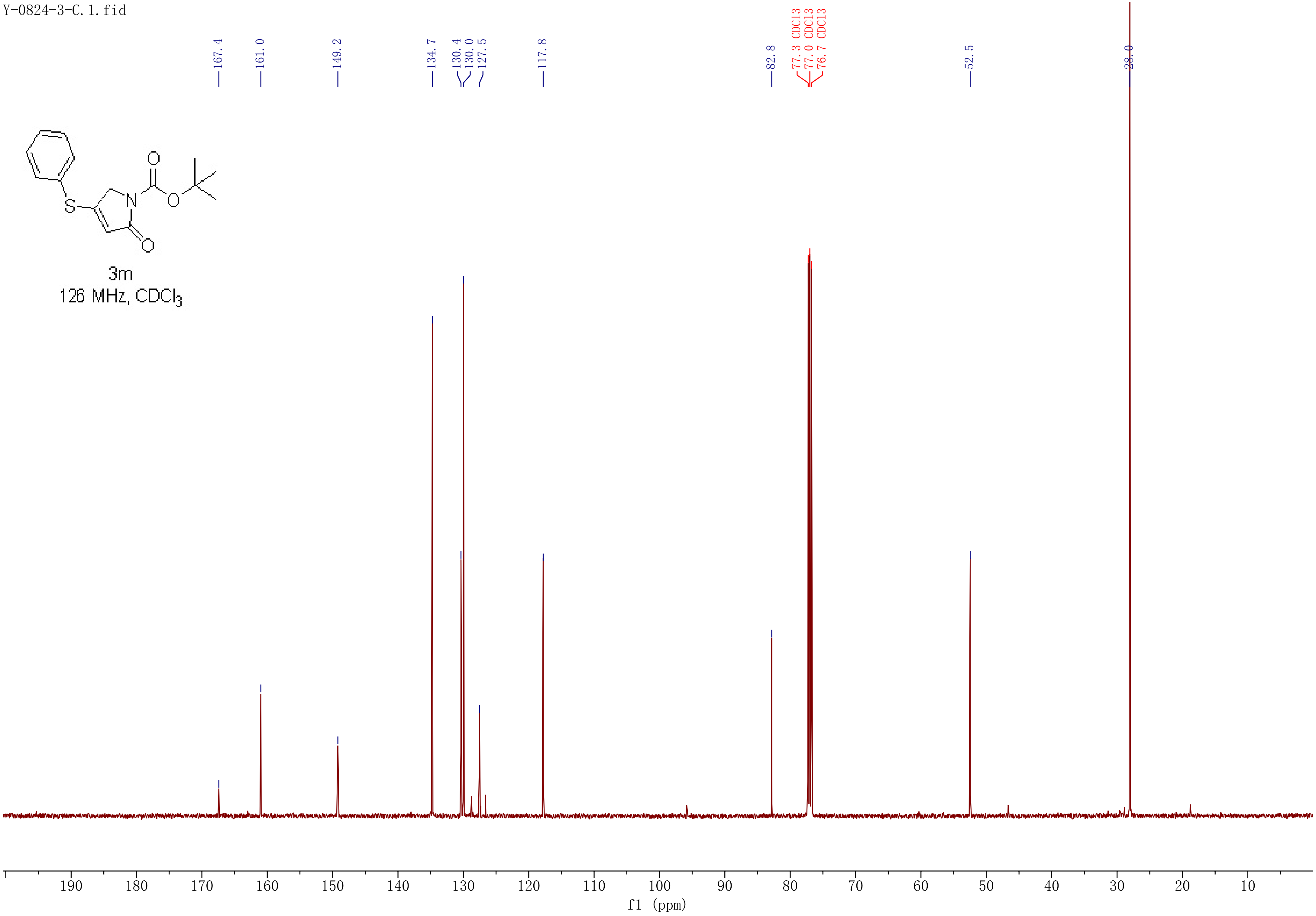

3m

126 MHz, $CDCl_3$

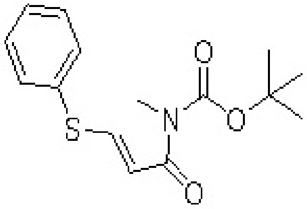

Qualitative Compound Identification Report

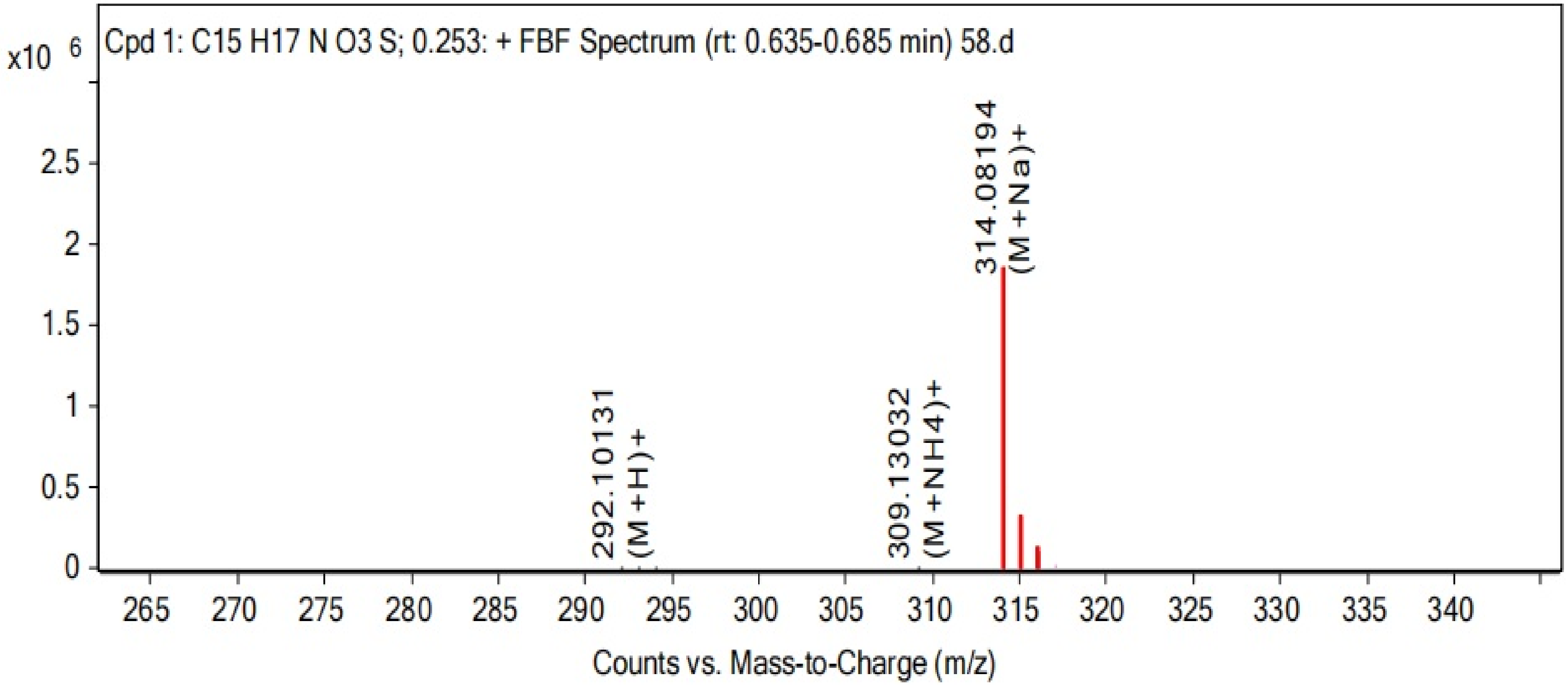

MS Spectrum Peak List

m/z	z	Abund	Ion
292.10131	1	4218.03	(M+H)+
293.10099	1	1393.4	(M+H)+
294.09372	1	697.93	(M+H)+
309.13032	1	444.69	(M+NH4)+
314.08194	1	1853212.5	(M+Na)+
315.08521	1	327228.69	(M+Na)+
316.07858	1	94058.63	(M+Na)+

MS Spectrum

Y-0824-5-H.1.fid

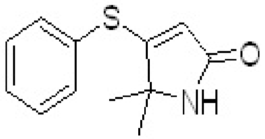

3n
500 MHz, $CDCl_3$

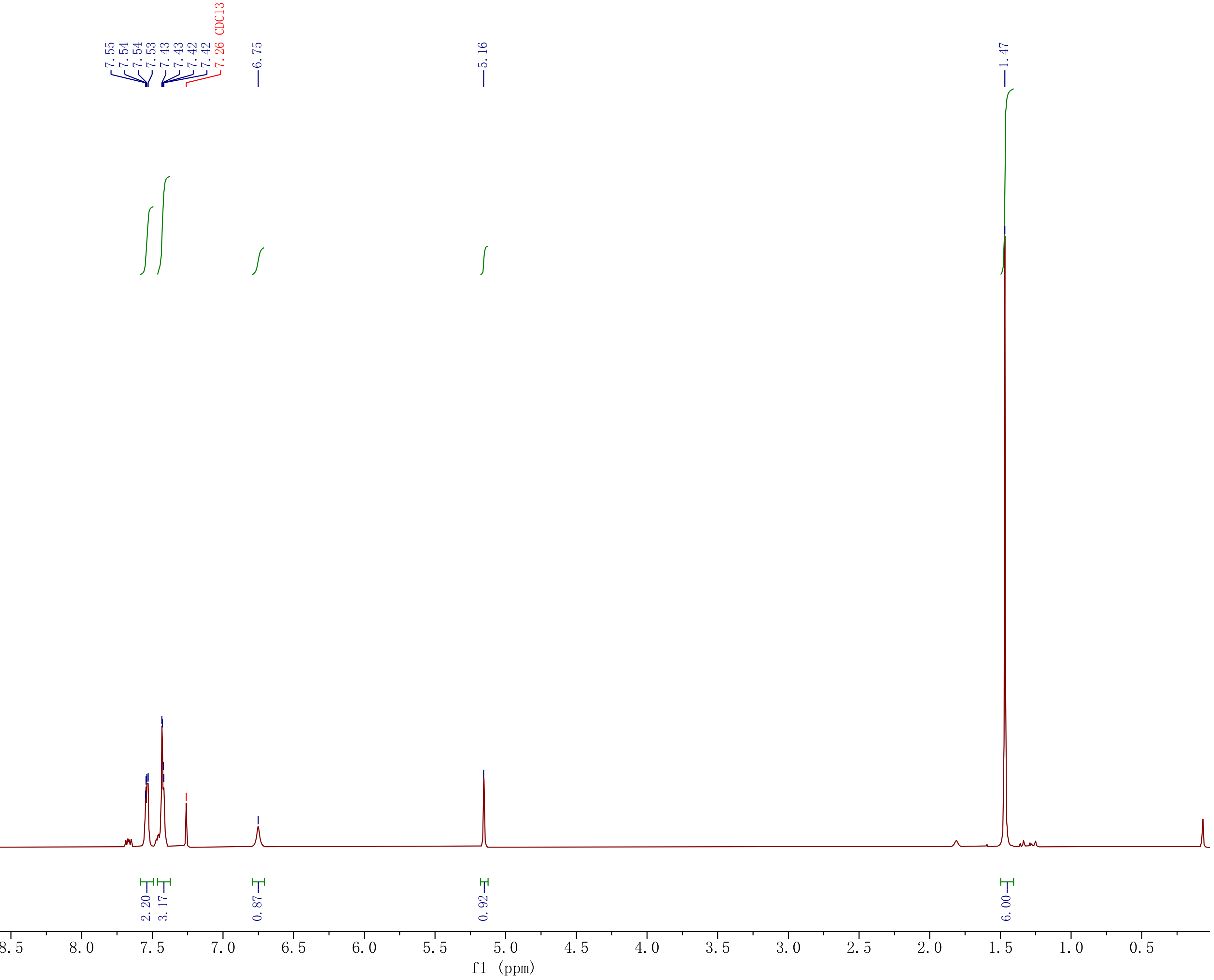

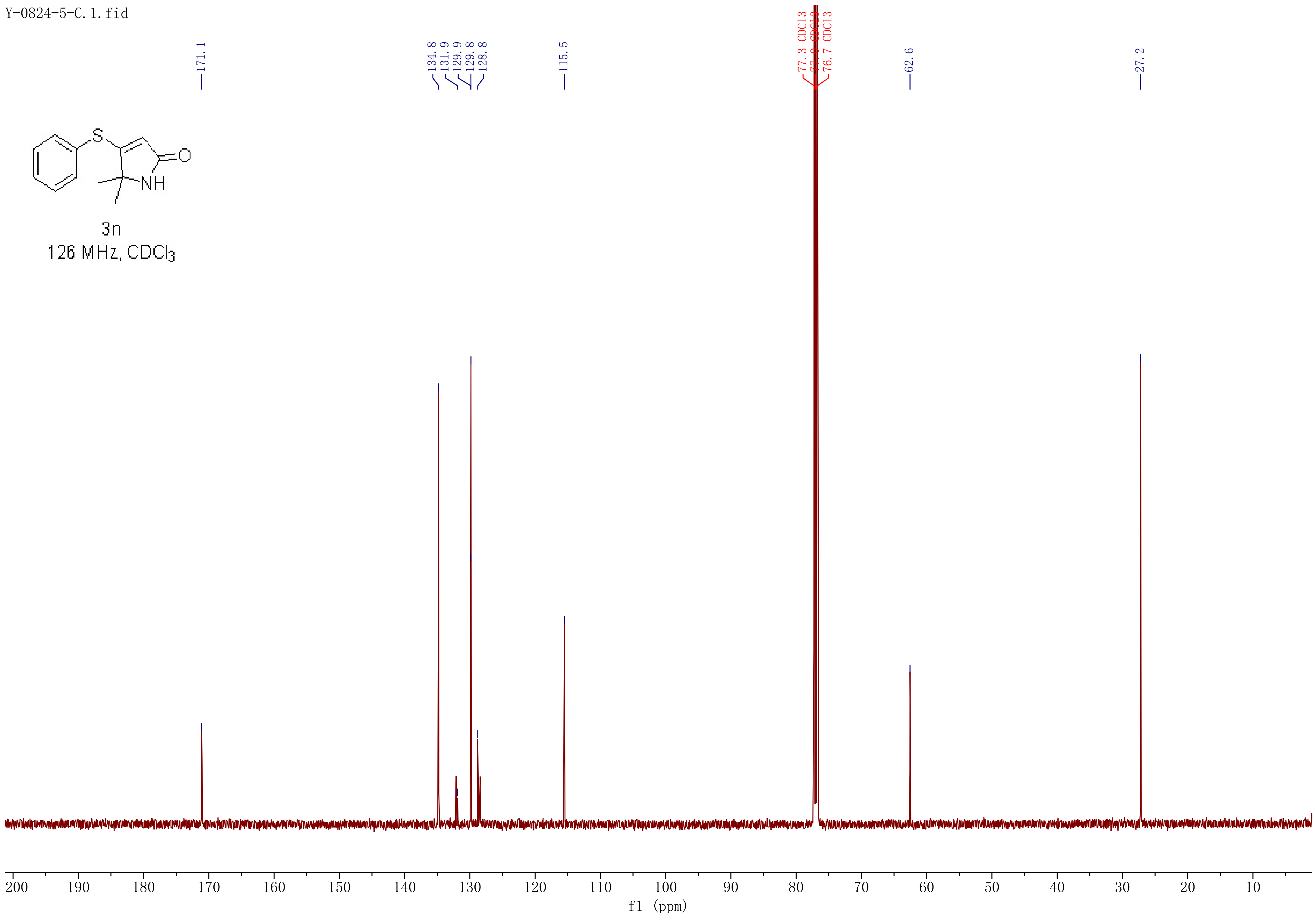
Y-0824-5-C.1.fid
171.1
134.8
131.9
129.9
129.8
128.8
115.5
77.3 CDCl3
76.7 CDCl3
62.6
27.2
3n
126 MHz, CDCl3
f1 (ppm)
200 190 180 170 160 150 140 130 120 110 100 90 80 70 60 50 40 30 20 10

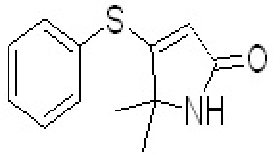

Qualitative Compound Identification Report

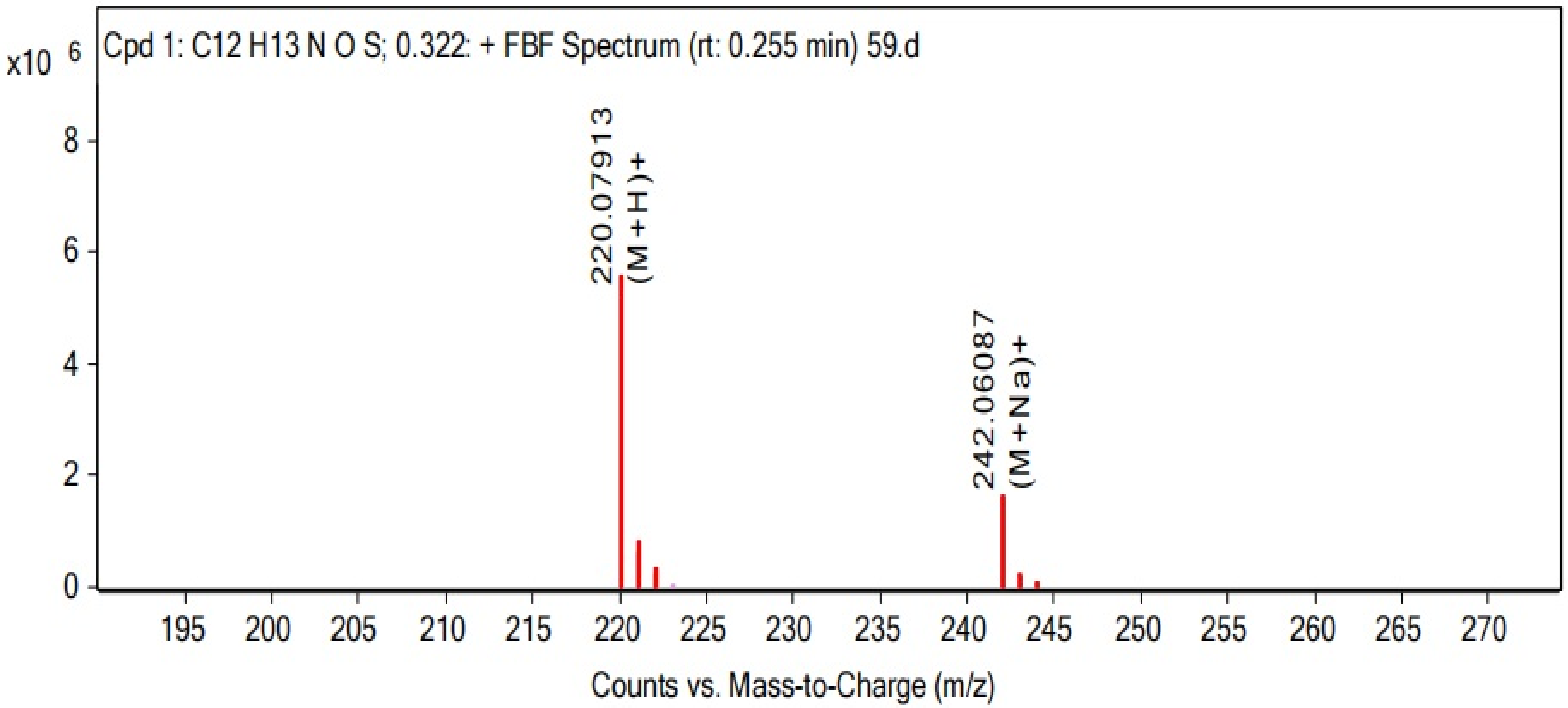

MS Spectrum Peak List

m/z	z	Abund	Ion
220.07913	1	5576327	(M+H)+
221.08214	1	614513.25	(M+H)+
222.07481	1	220228.44	(M+H)+
242.06087	1	1638462.13	(M+Na)+
243.06419	1	216411.05	(M+Na)+
244.05693	1	76272.35	(M+Na)+

MS Spectrum

1014-27.1.fid

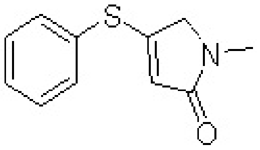

3o
300 MHz, $CDCl_3$

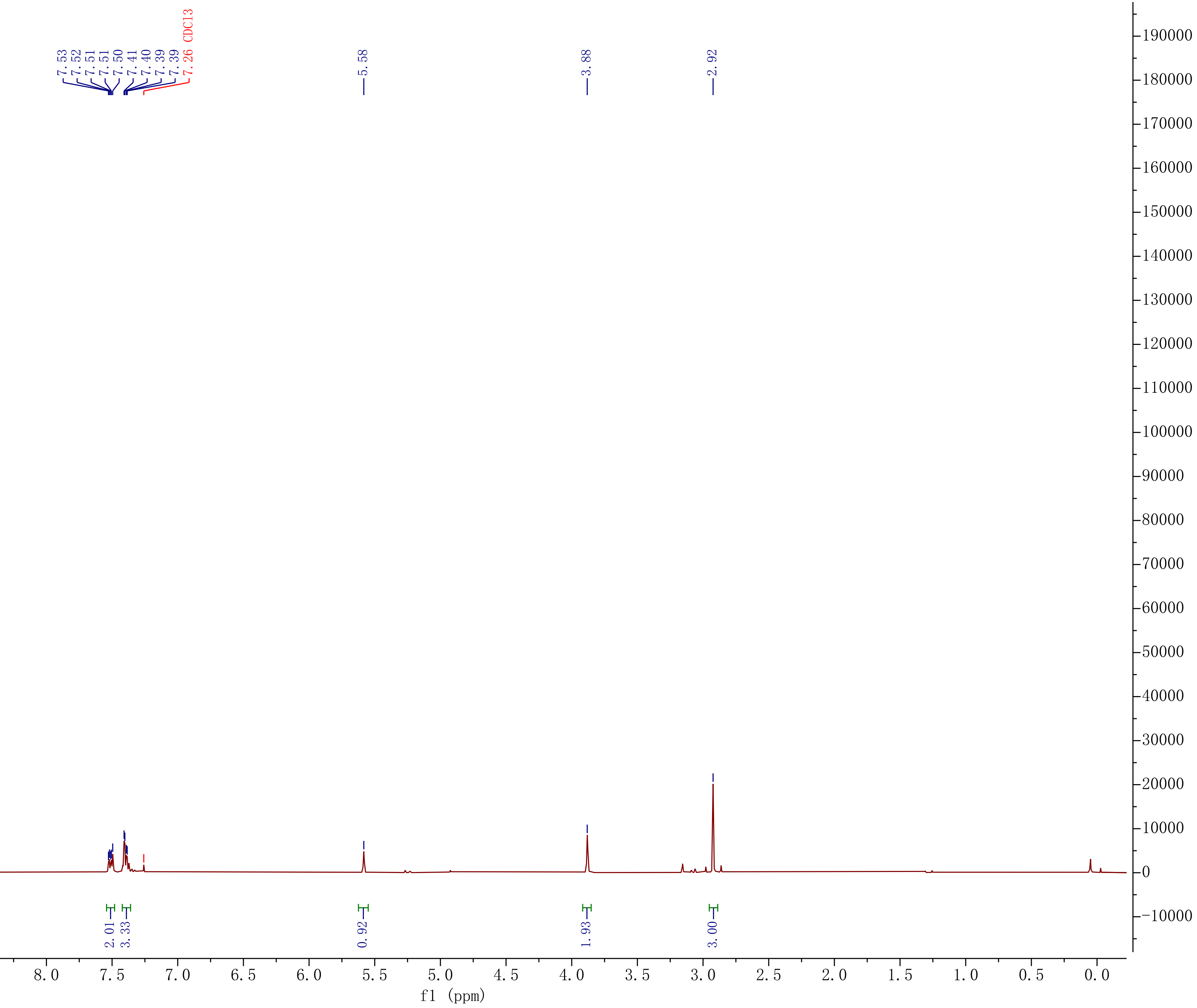

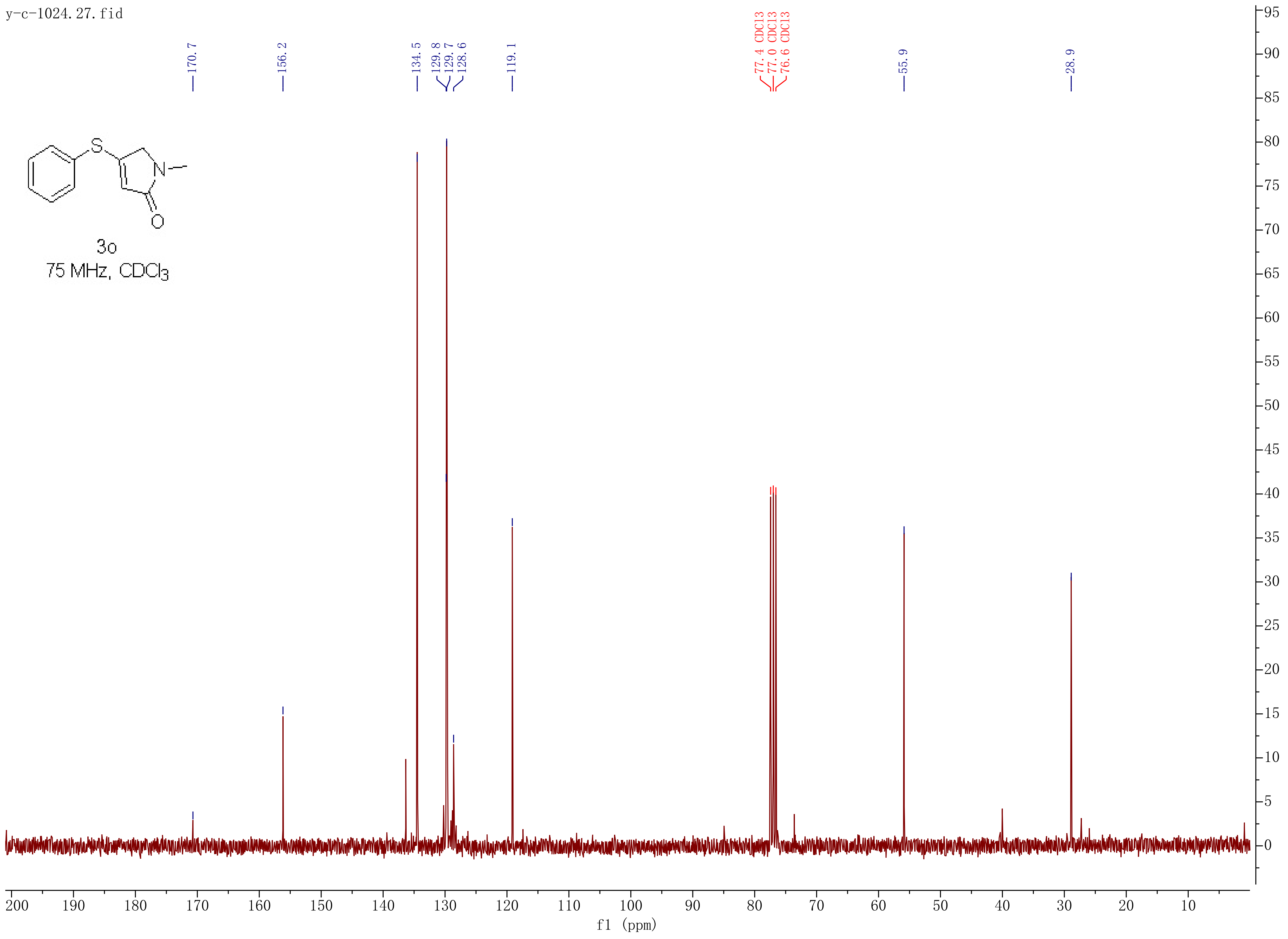

y-c-1024.27.fid
170.7
156.2
134.5
129.8
129.7
128.6
119.1
77.4 CDCl3
77.0 CDCl3
76.6 CDCl3
55.9
28.9
S
N
O
3o
75 MHz, CDCl3
f1 (ppm)

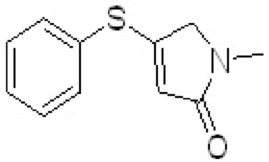

Qualitative Compound Identification Report

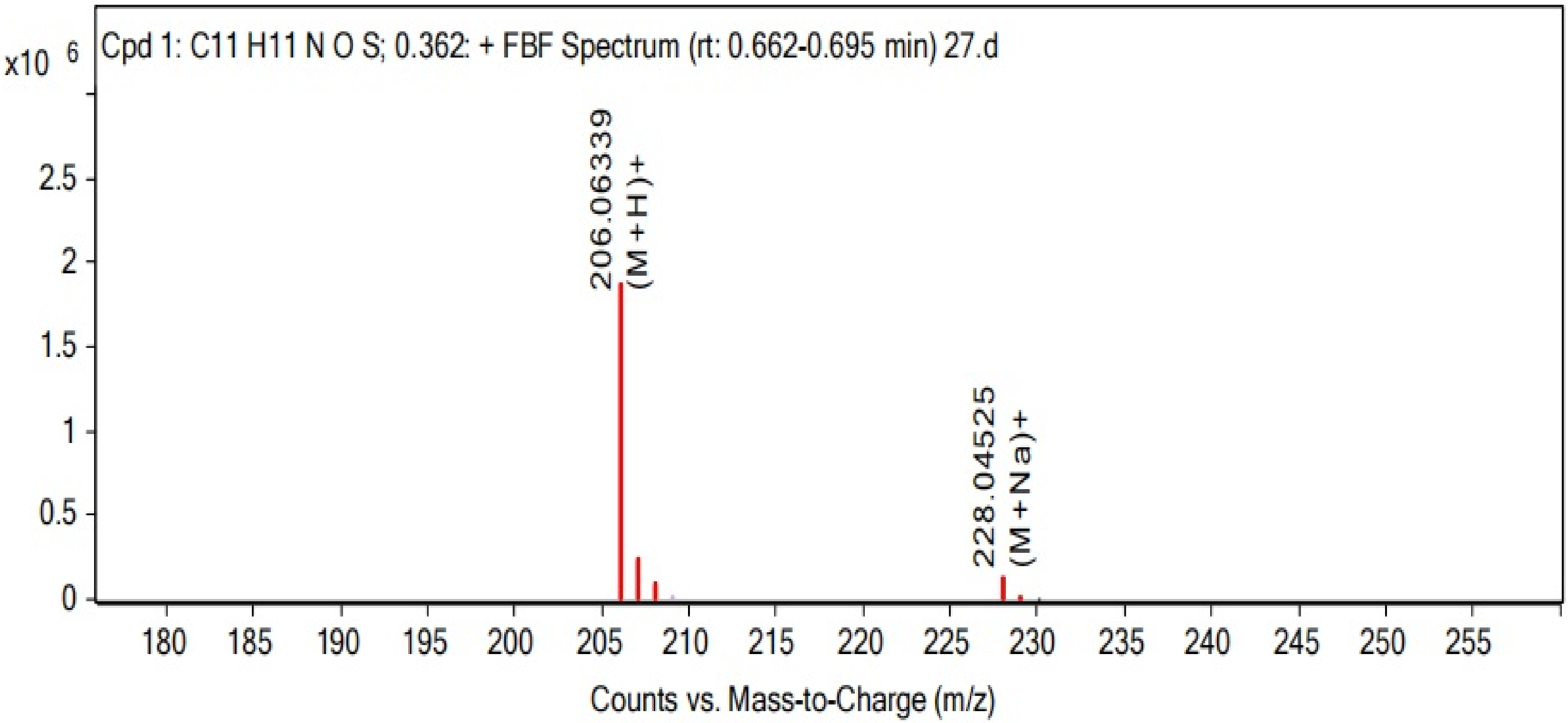

MS Spectrum Peak List

m/z	z	Abund	Ion
206.06339	1	1882740.63	(M+H)+
207.0673	1	235728.58	(M+H)+
208.05901	1	88742.5	(M+H)+
228.04525	1	133212.16	(M+Na)+
229.04855	1	20910.43	(M+Na)+
230.04113	1	7740.99	(M+Na)+

MS Spectrum

Y-0824-1-H.1.fid

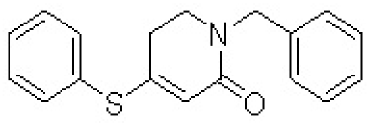

3p
500 MHz, $CDCl_3$

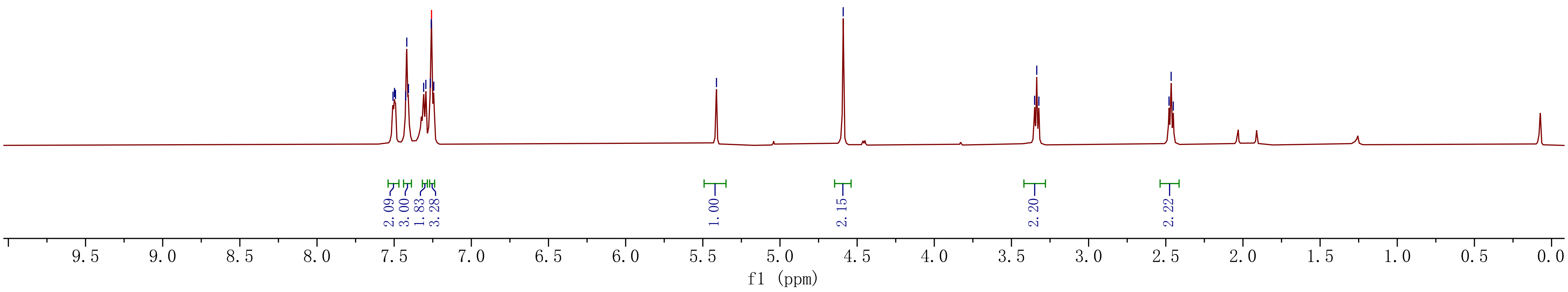

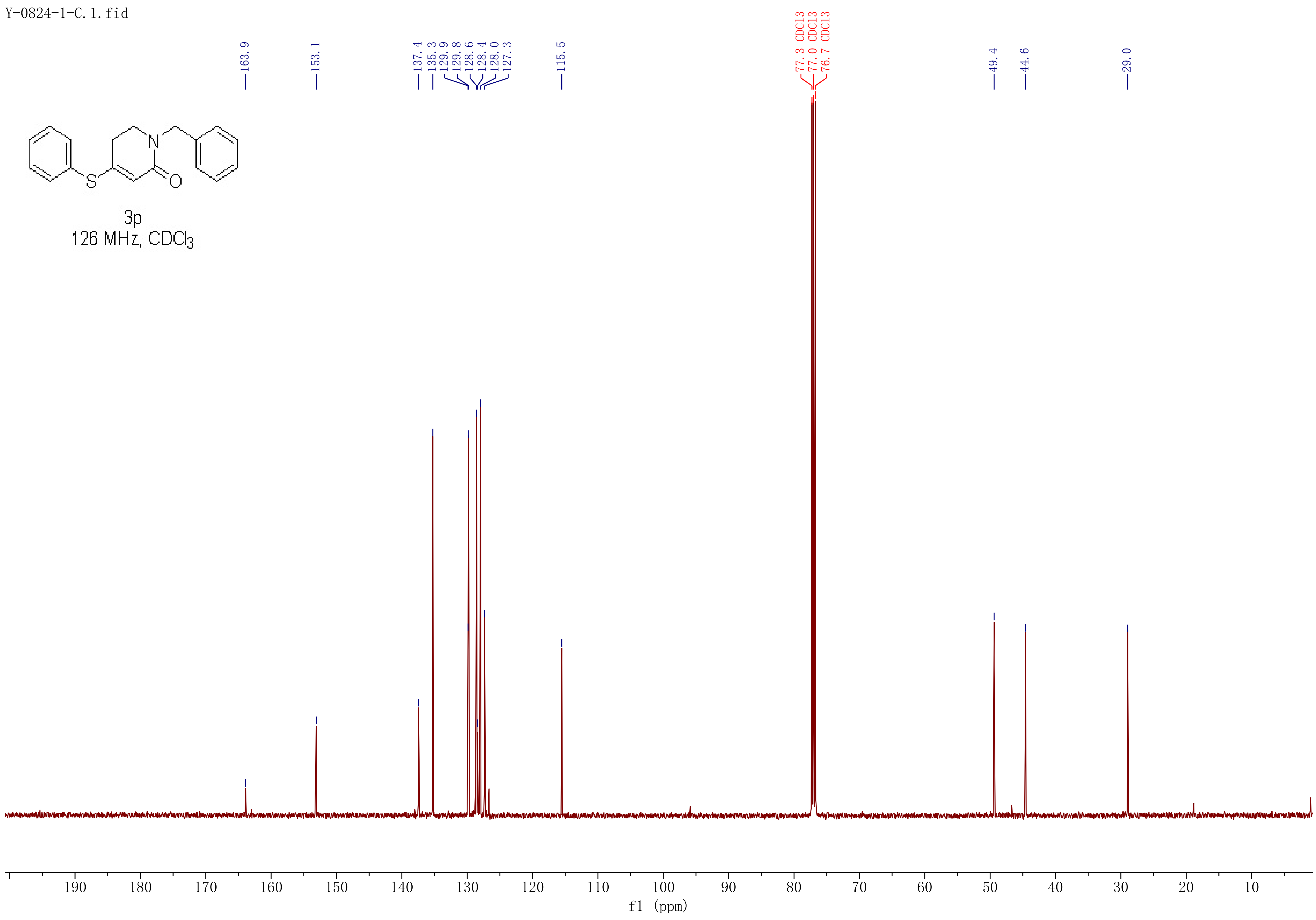

Y-0824-1-C.1.fid
163.9
153.1
137.4
135.3
129.9
129.8
128.6
128.4
128.0
127.3
115.5
77.3 CDCl3
77.0 CDCl3
76.7 CDCl3
49.4
44.6
29.0
S
N
O
3p
126 MHz, CDCl3
190
180
170
160
150
140
130
120
110
100
90
80
70
60
50
40
30
20
10
f1 (ppm)

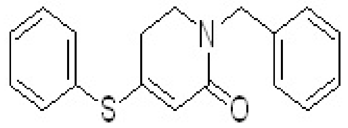

Qualitative Compound Identification Report

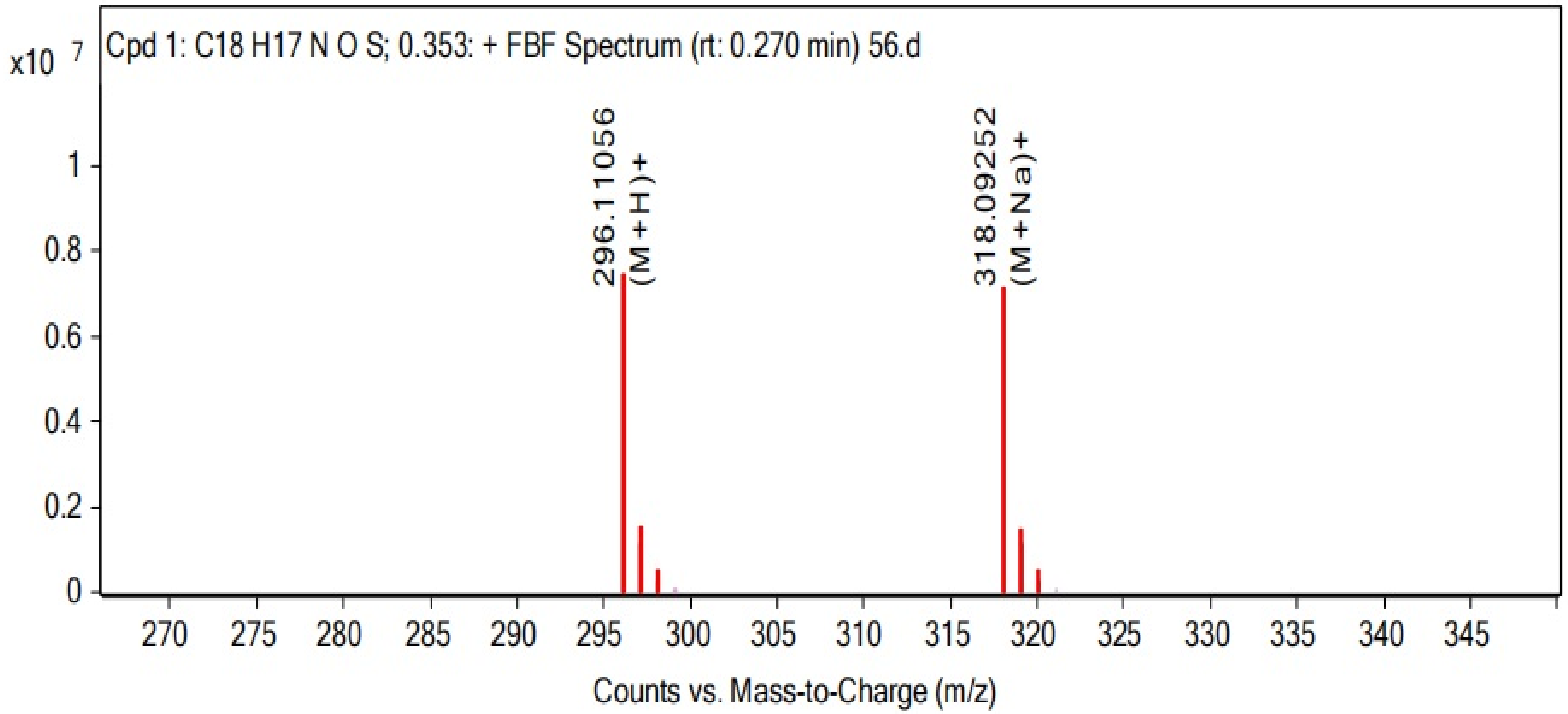

MS Spectrum Peak List

m/z	z	Abund	Ion
296.11056	1	7430712	(M+H)+
297.11336	1	1167504.63	(M+H)+
298.10645	1	268283.16	(M+H)+
318.09252	1	7132322.5	(M+Na)+
319.09537	1	1119961.5	(M+Na)+
320.08814	1	262951.84	(M+Na)+

MS Spectrum

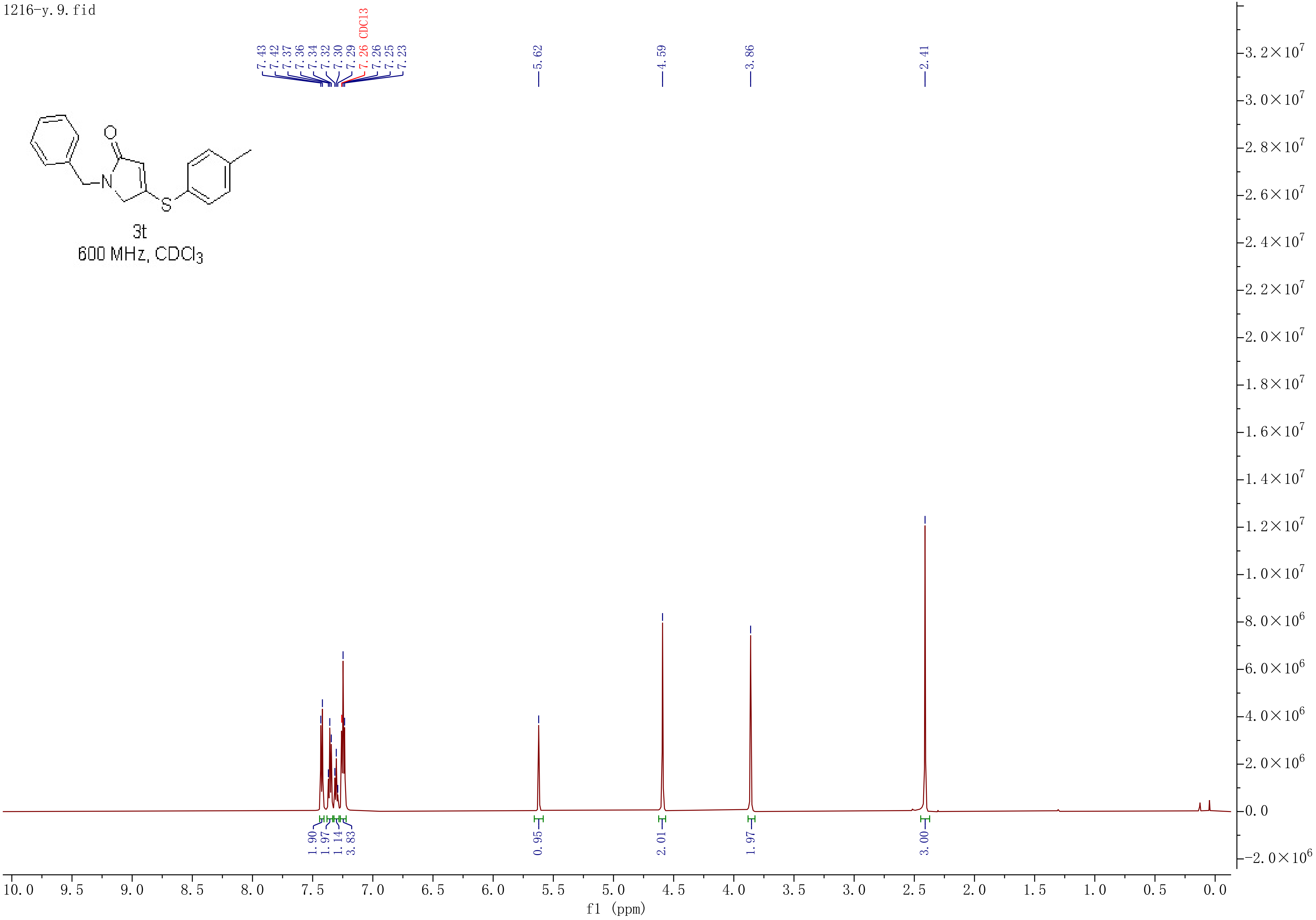

1216-y.9.fid
7.43
7.42
7.37
7.36
7.34
7.32
7.30
7.29
7.26 CDCl3
7.26
7.25
7.23
5.62
4.59
3.86
2.41
O
N
S
3t
600 MHz, CDCl3
1.90
1.97
1.14
3.83
0.95
2.01
1.97
3.00
f1 (ppm)

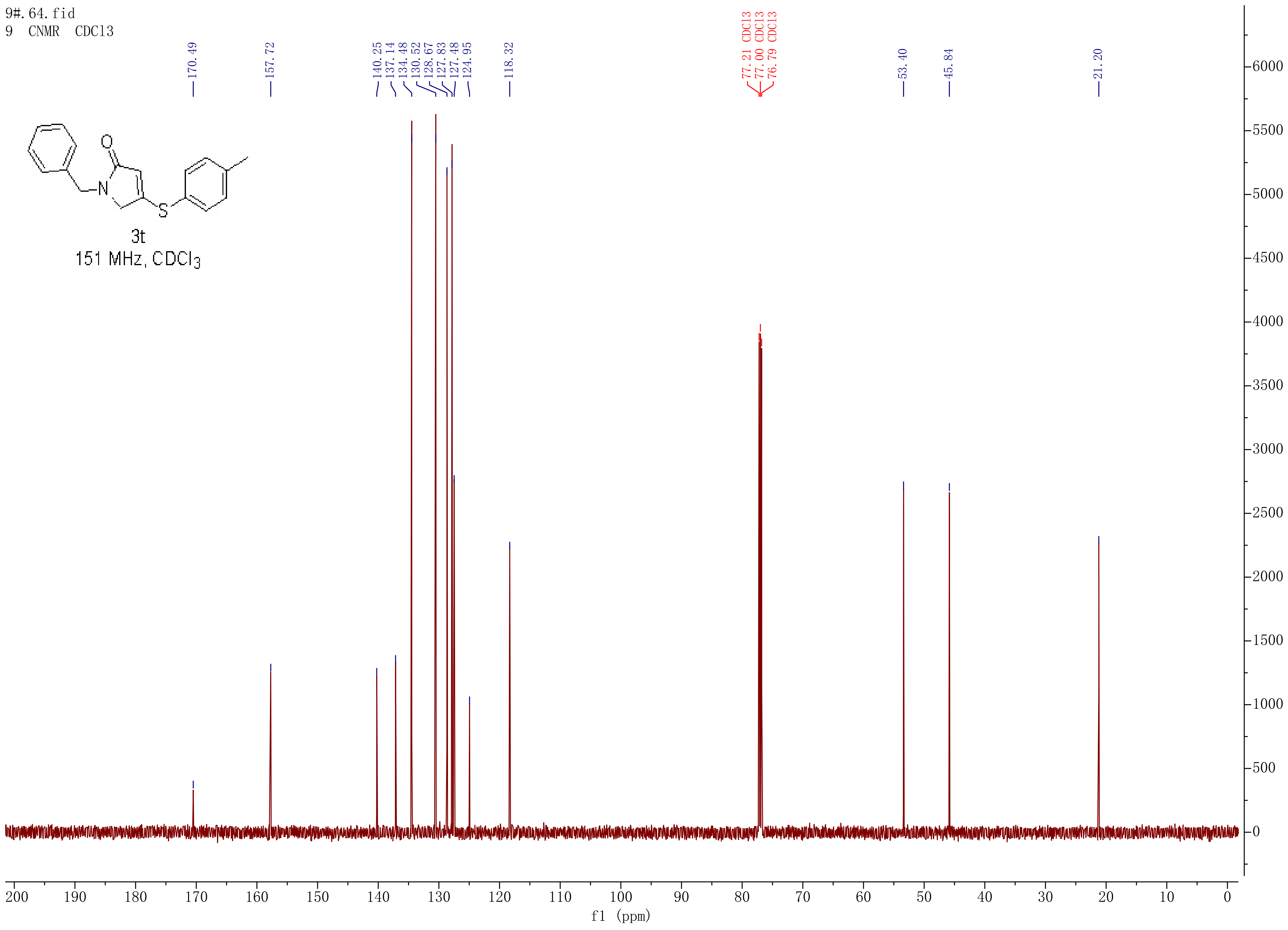
9#.64.fid
9 CNMR CDCl3
170.49
157.72
140.25
137.14
134.48
130.52
128.67
127.83
127.48
124.95
118.32
77.21 CDCl3
77.00 CDCl3
76.79 CDCl3
53.40
45.84
21.20
3t
151 MHz, CDCl3
f1 (ppm)

1216-y.10.fid

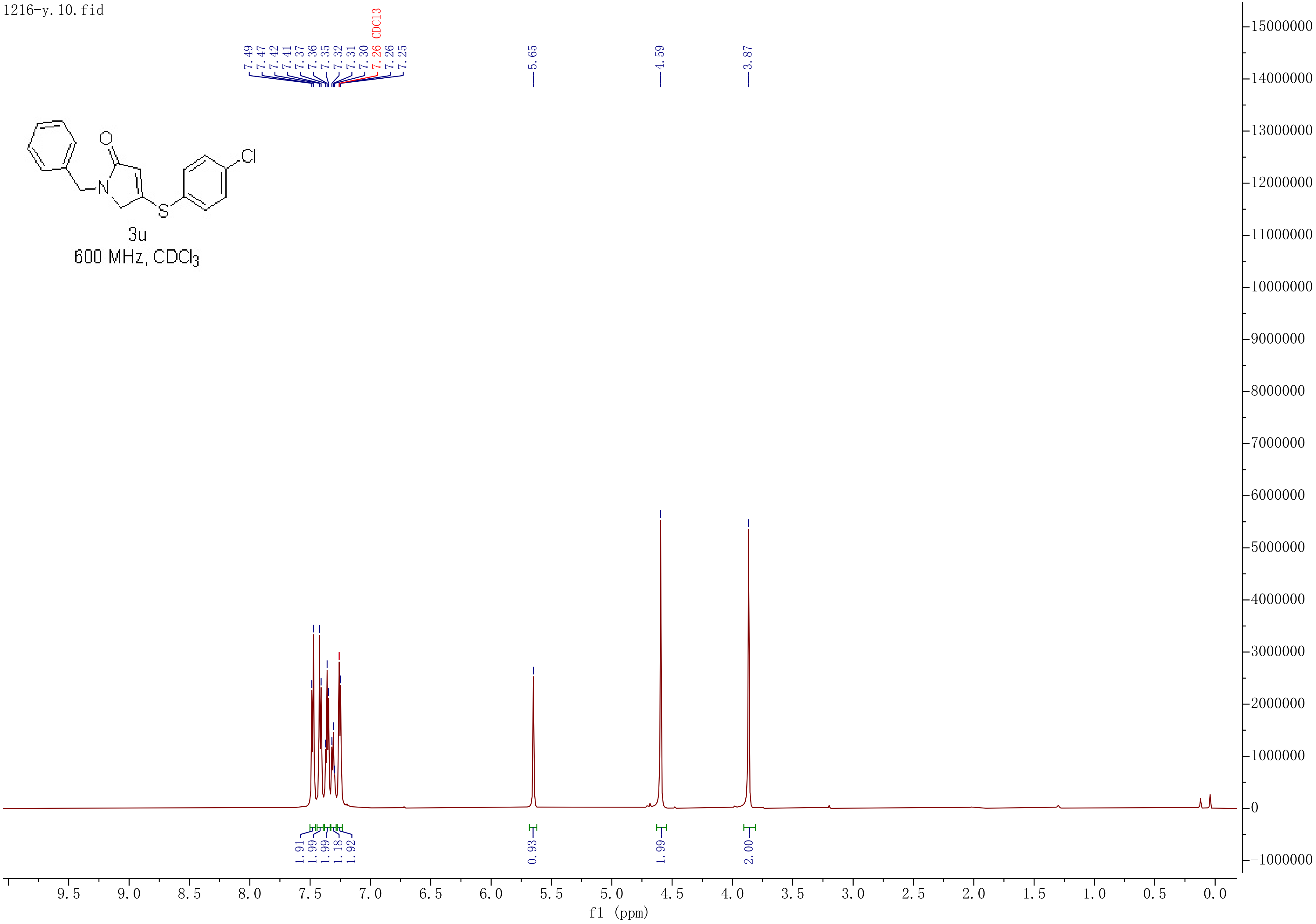

3u
600 MHz, CDCl3
7.49
7.47
7.42
7.41
7.37
7.36
7.35
7.32
7.31
7.30
7.26 CDCl3
7.26
7.25
5.65
4.59
3.87
1.91
1.99
1.99
1.18
1.92
0.93
1.99
2.00
f1 (ppm)

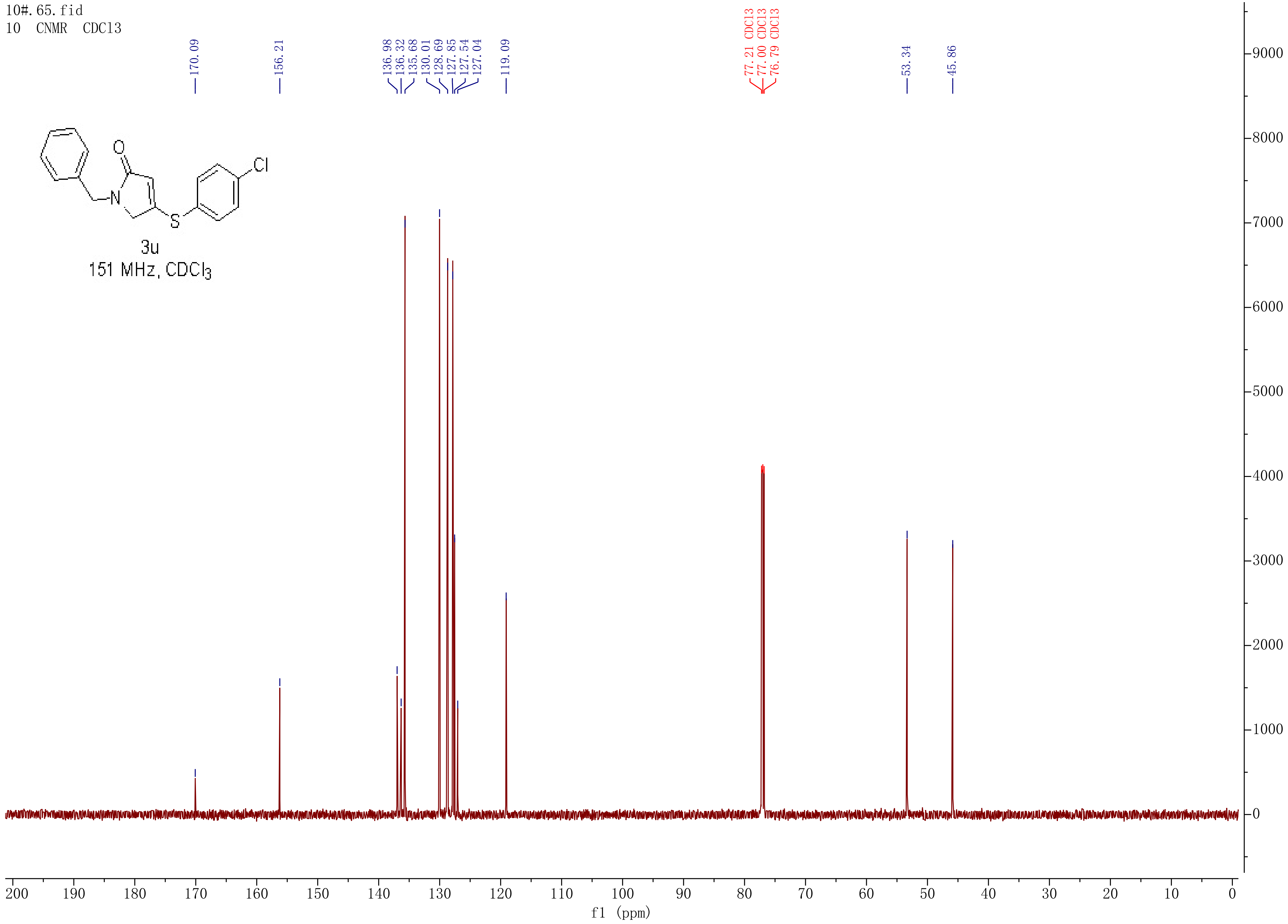

10#.65.fid
10 CNMR CDCl3
170.09
156.21
136.98
136.32
135.68
130.01
128.69
127.85
127.54
127.04
119.09
77.21 CDCl3
77.00 CDCl3
76.79 CDCl3
53.34
45.86
O
N
S
Cl
3u
151 MHz, CDCl3
f1 (ppm)

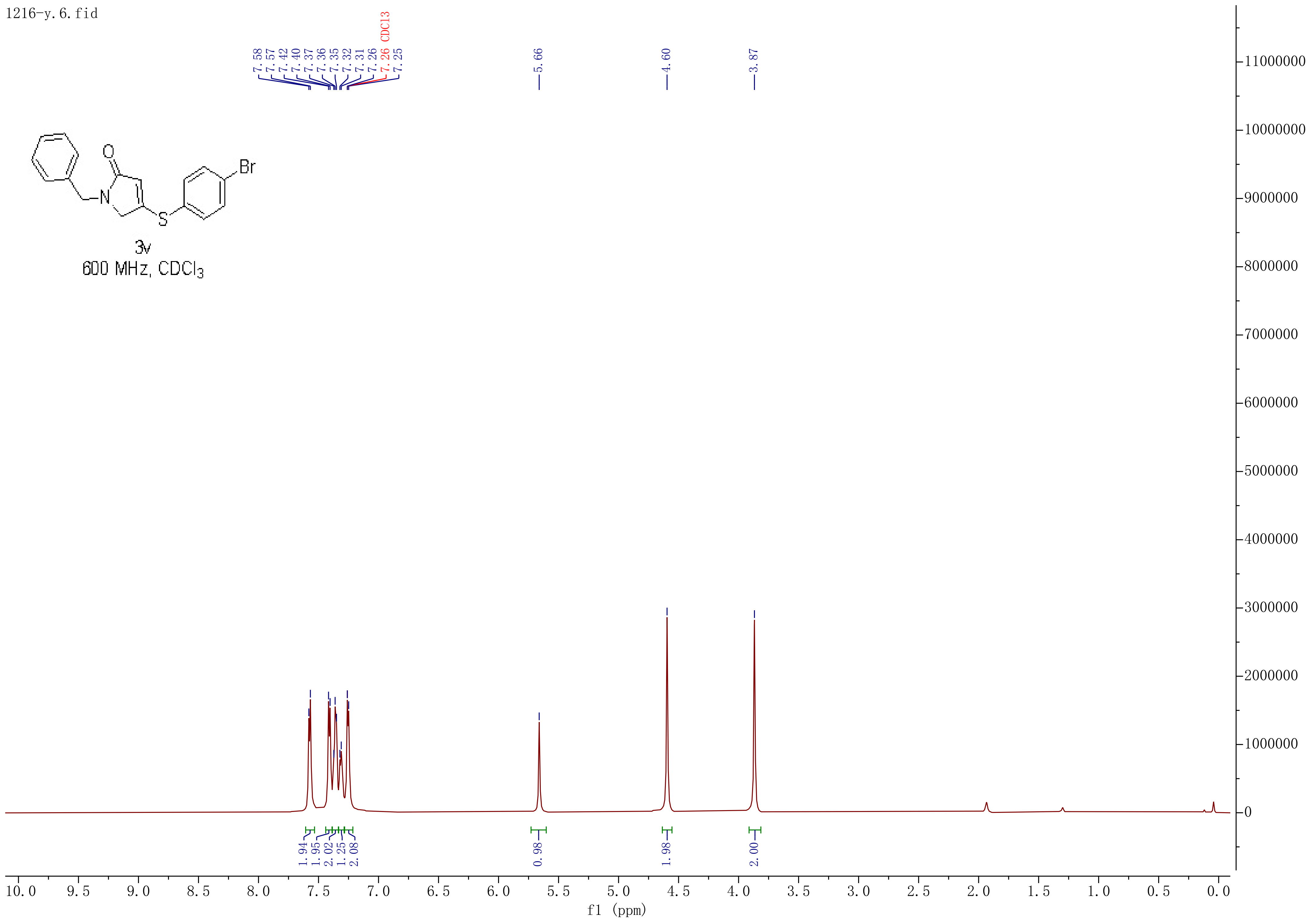
1216-y.6.fid
7.58
7.57
7.42
7.40
7.37
7.36
7.35
7.32
7.31
7.26
7.26 CDCl3
7.25
5.66
4.60
3.87
O
N
S
Br
3v
600 MHz, CDCl3
1.94
1.95
2.02
1.25
2.08
0.98
1.98
2.00
f1 (ppm)

6#.61.fid
6 CNMR CDCl3

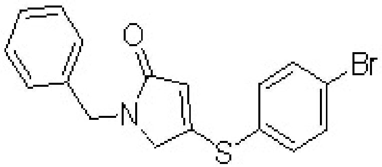

3v
151 MHz, $CDCl_3$

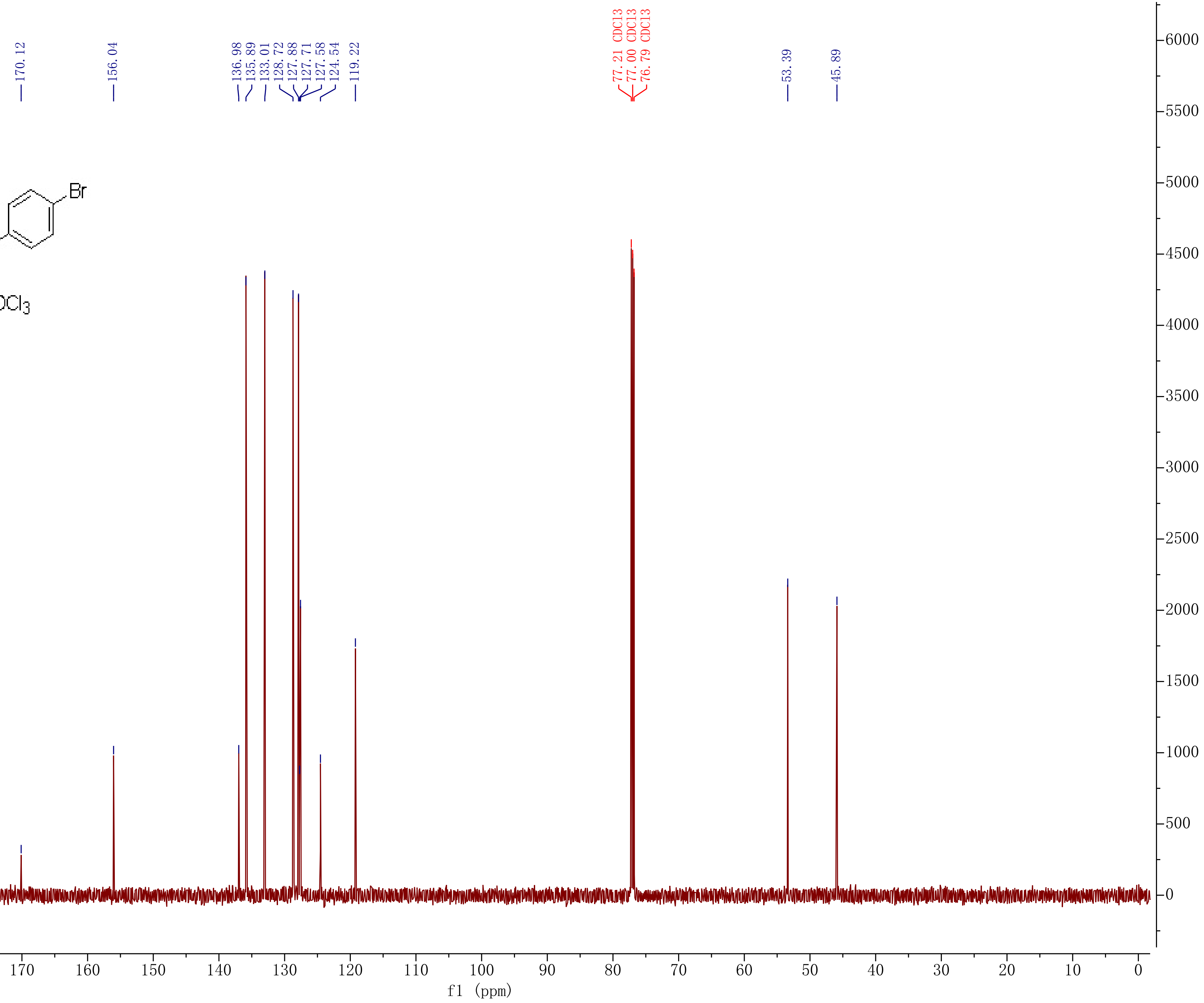

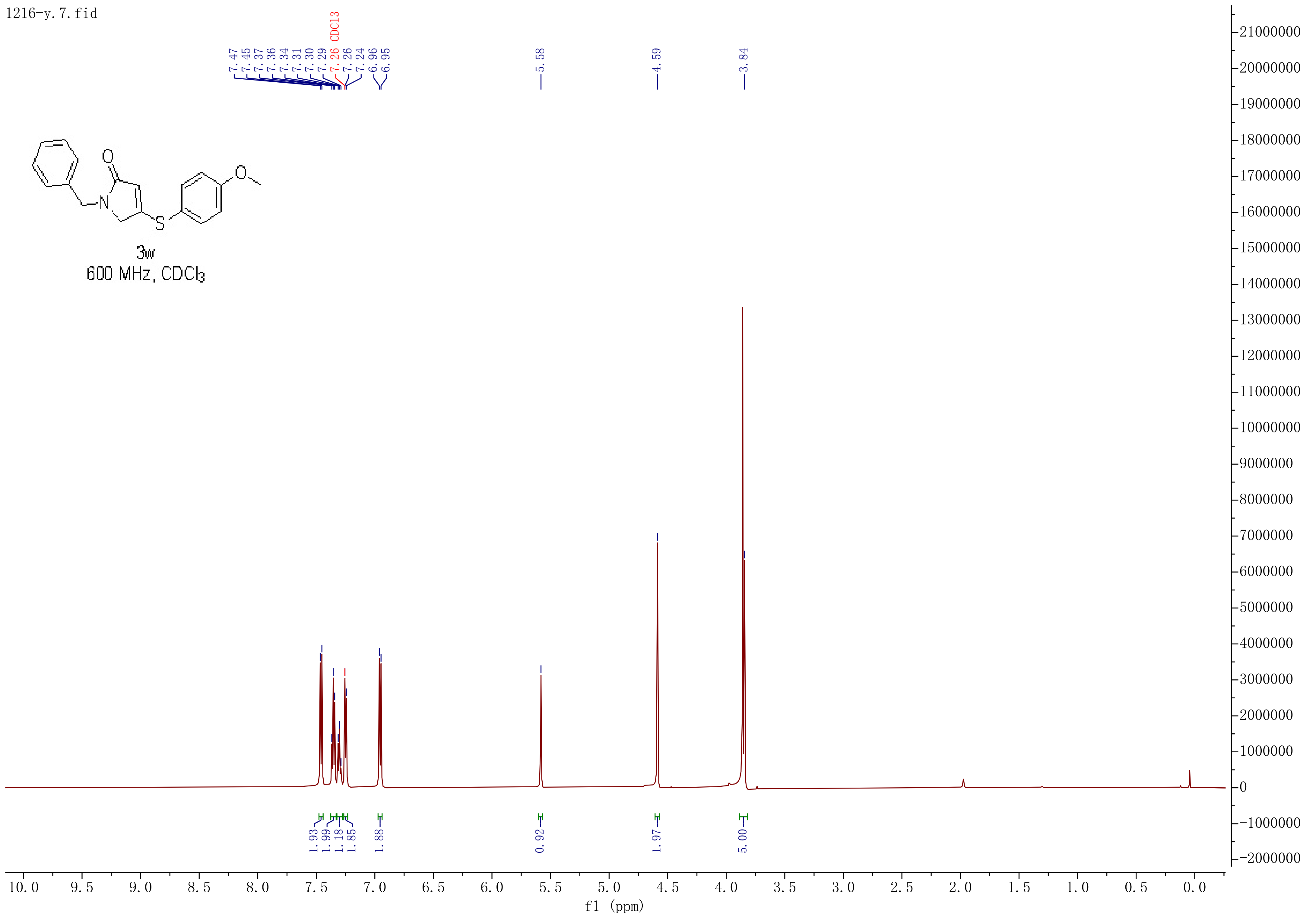
1216-y.7.fid
7.47
7.45
7.37
7.36
7.34
7.31
7.30
7.29
7.26 CDCl3
7.26
7.24
6.96
6.95
5.58
4.59
3.84
3w
600 MHz, CDCl3
1.93
1.99
1.18
1.85
1.88
0.92
1.97
5.00
f1 (ppm)

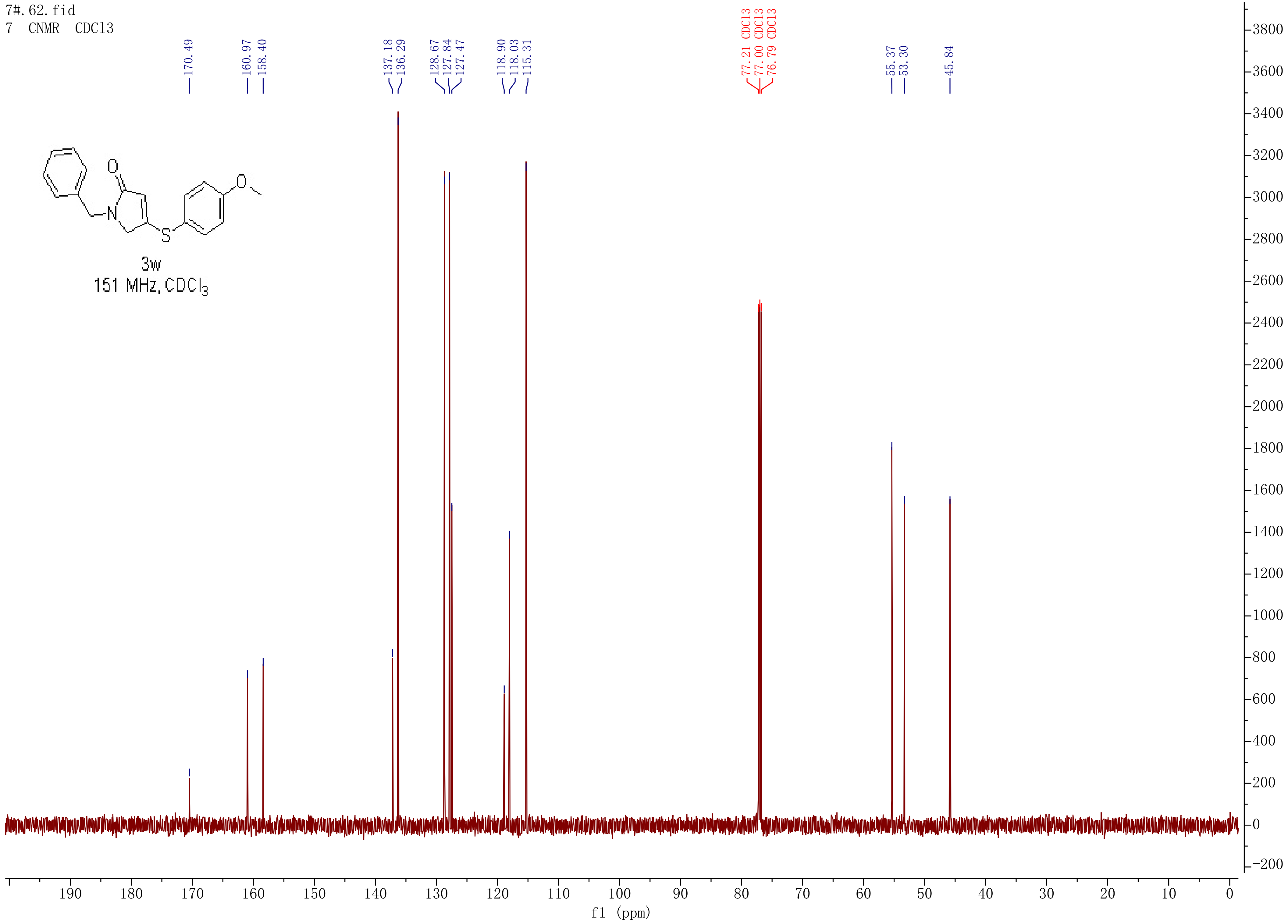
7#.62.fid
7 CNMR CDCl3
170.49
160.97
158.40
137.18
136.29
128.67
127.84
127.47
118.90
118.03
115.31
77.21 CDCl3
77.00 CDCl3
76.79 CDCl3
55.37
53.30
45.84
3w
151 MHz, CDCl3
f1 (ppm)

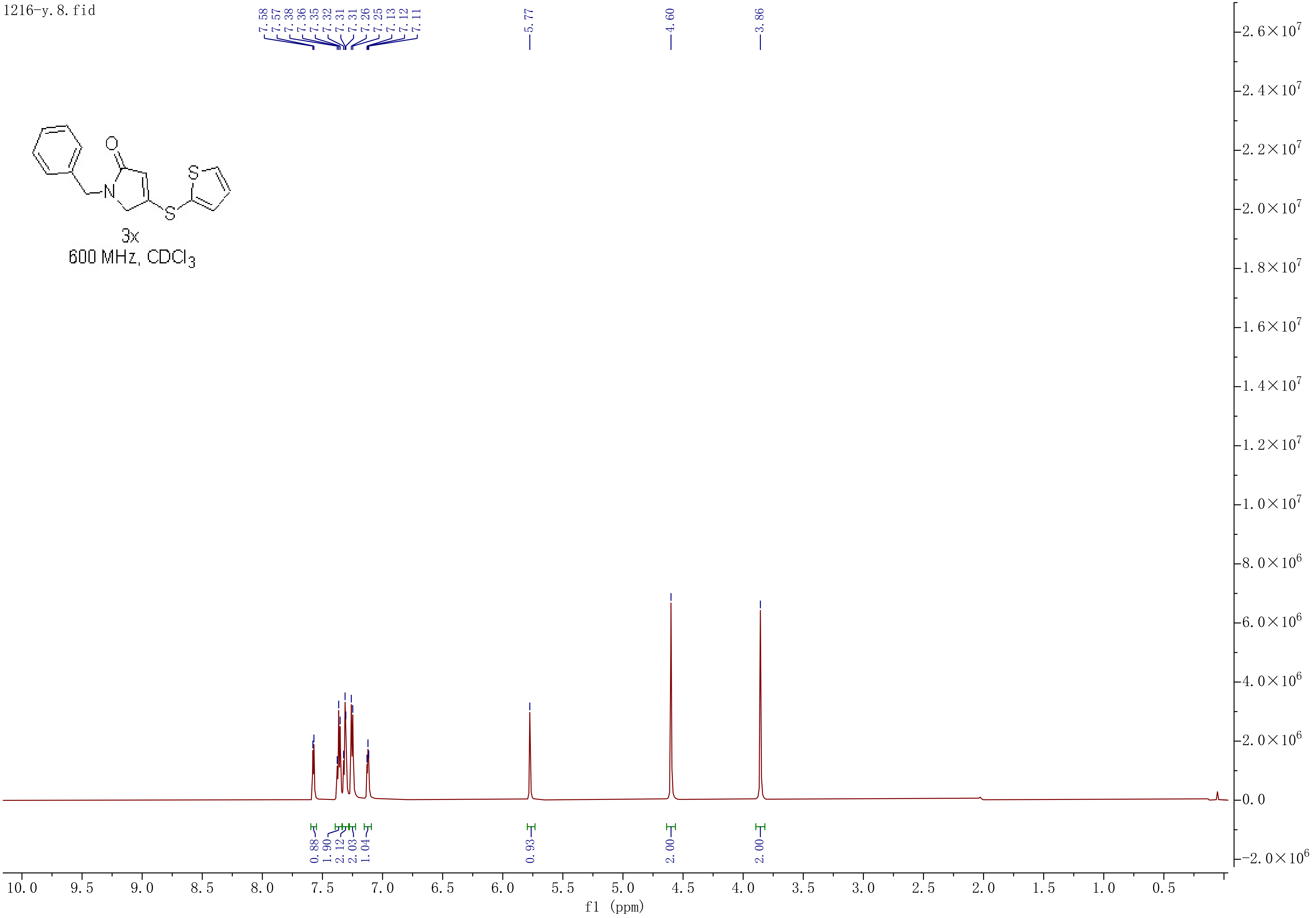
1216-y.8.fid
7.58 7.57 7.38 7.36 7.35 7.32 7.31 7.31 7.26 7.25 7.13 7.12 7.11
5.77
4.60
3.86
3x
600 MHz, CDCl3
0.88 1.90 2.12 2.03 1.04
0.93
2.00
2.00
10.0 9.5 9.0 8.5 8.0 7.5 7.0 6.5 6.0 5.5 5.0 4.5 4.0 3.5 3.0 2.5 2.0 1.5 1.0 0.5
f1 (ppm)
2.6×10^7 2.4×10^7 2.2×10^7 2.0×10^7 1.8×10^7 1.6×10^7 1.4×10^7 1.2×10^7 1.0×10^7 8.0×10^6 6.0×10^6 4.0×10^6 2.0×10^6 0.0 −2.0×10^6

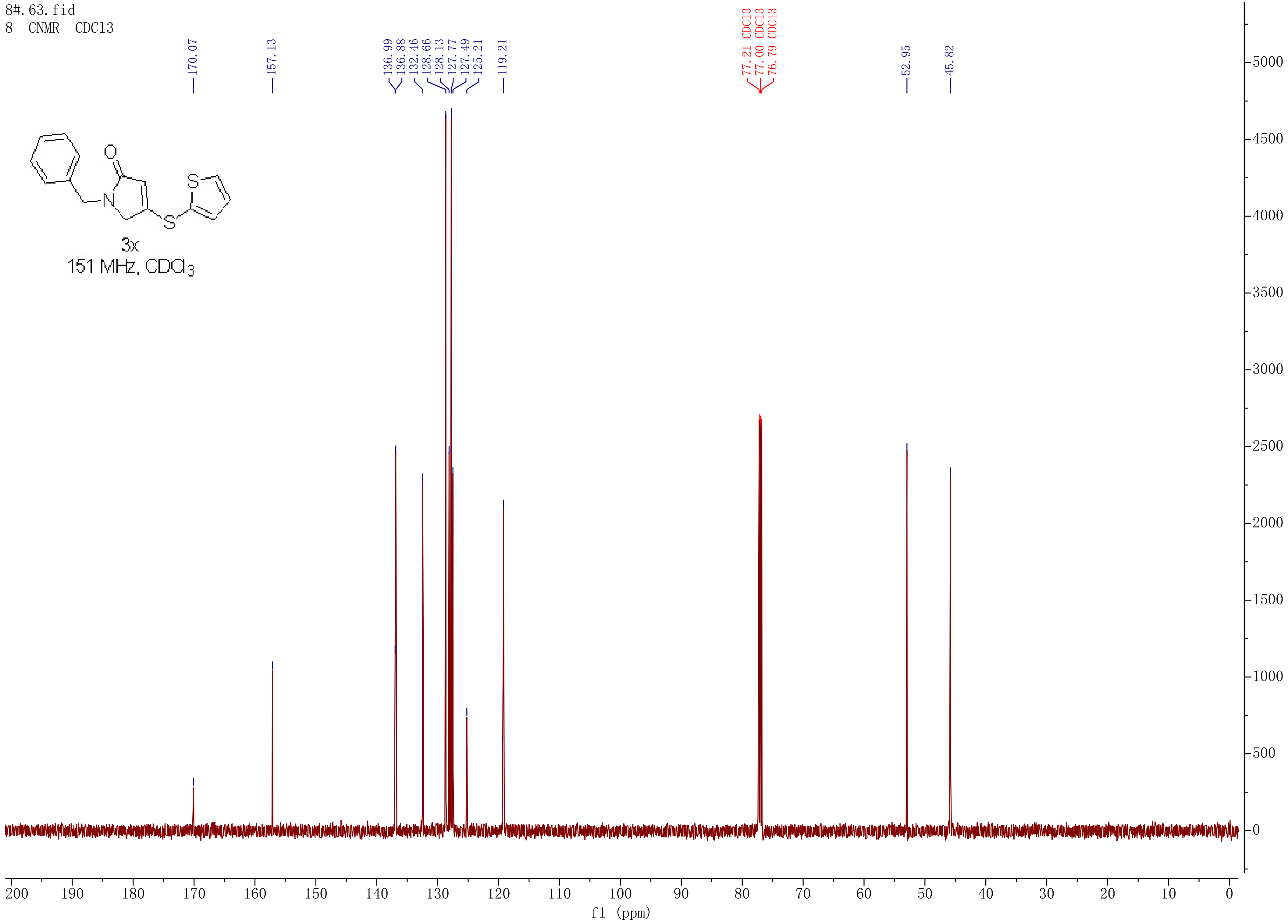

8#.63.fid
8 CNMR CDCl3
170.07
157.13
136.99
136.88
132.46
128.66
128.13
127.77
127.49
125.21
119.21
77.21 CDCl3
77.00 CDCl3
76.79 CDCl3
52.95
45.82
3x
151 MHz, CDCl3
f1 (ppm)

yzp-0418-7-H

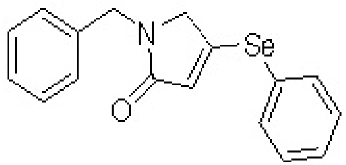

4a

400 MHz, $CDCl_3$

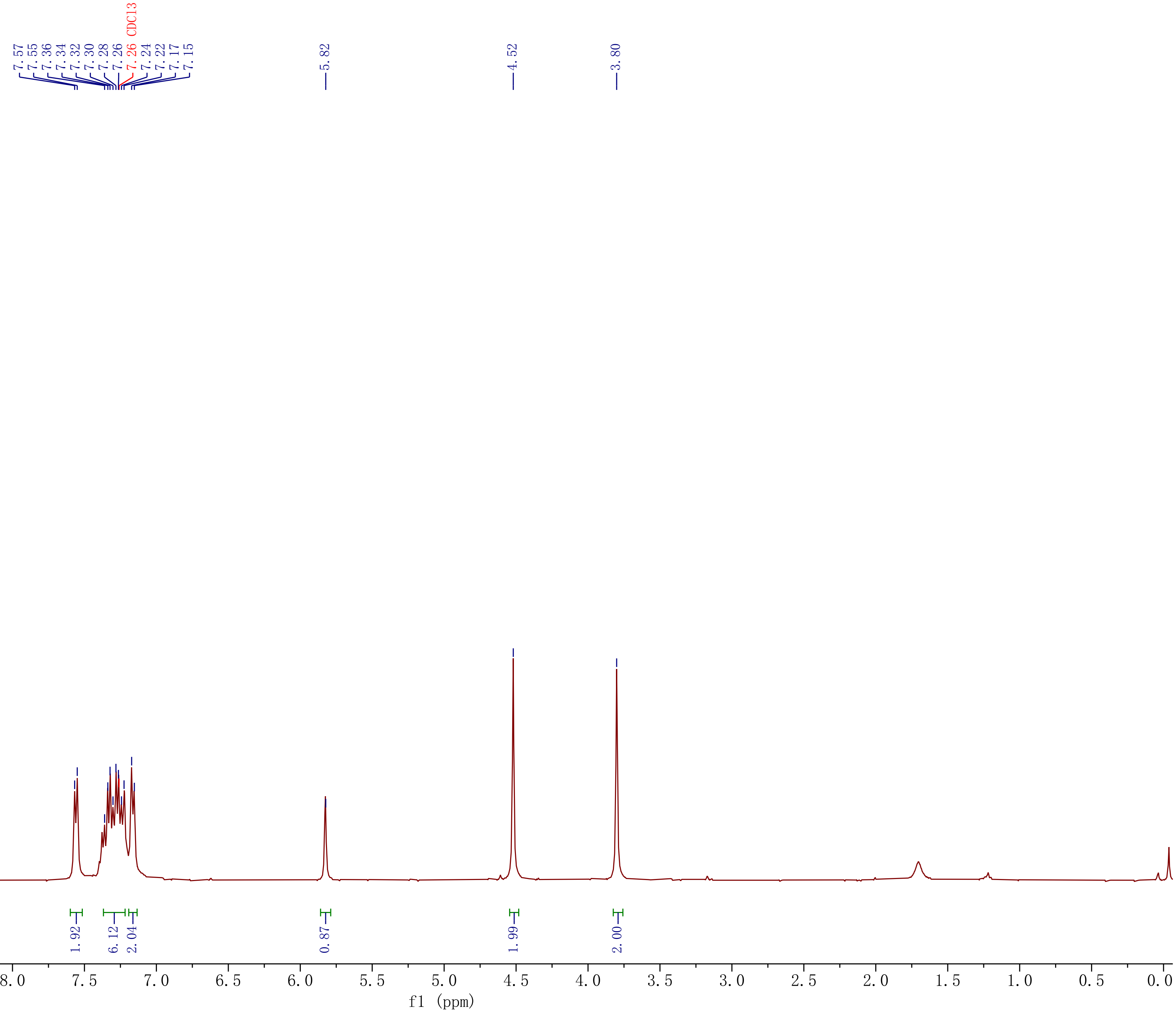

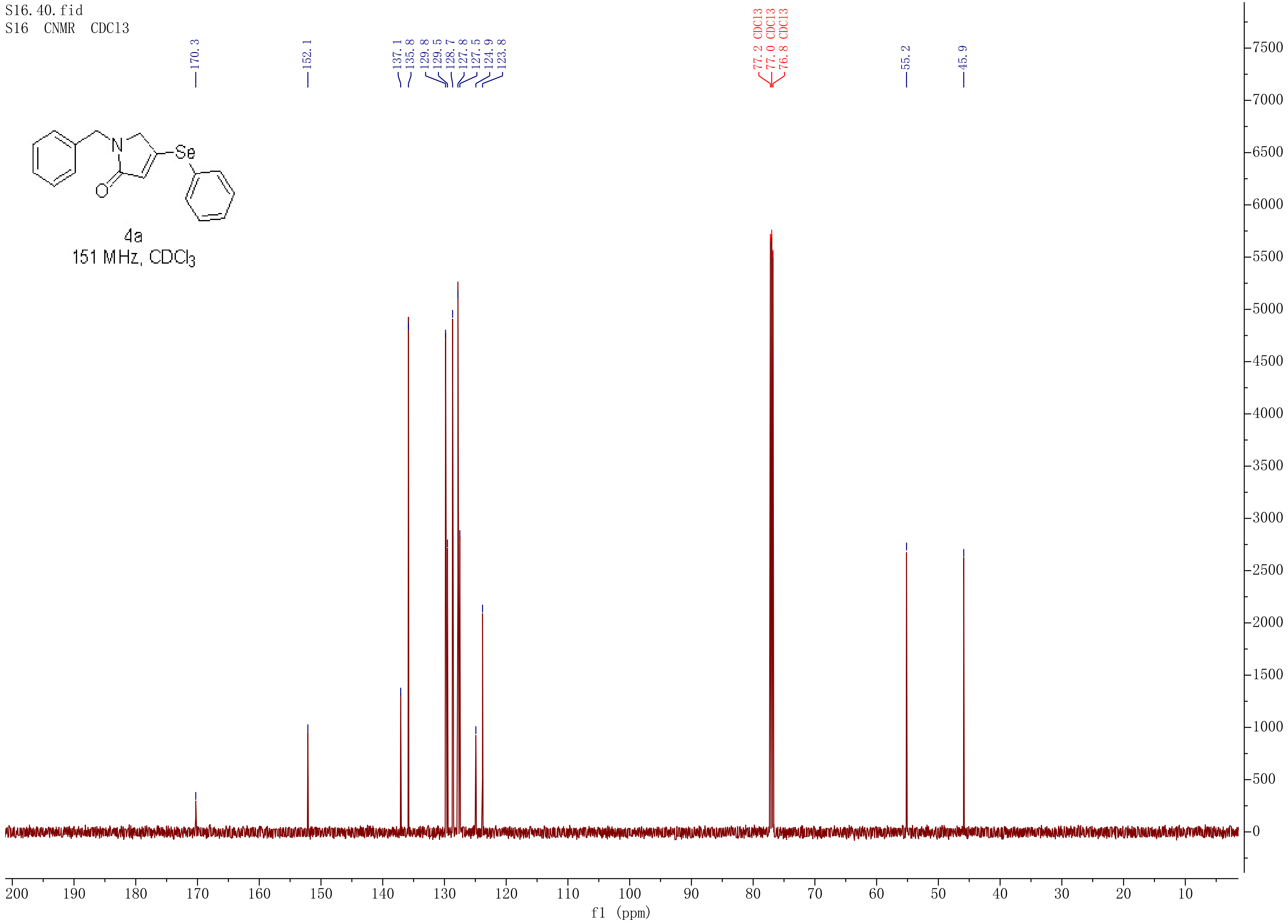
S16.40.fid
S16 CNMR CDCl3
170.3
152.1
137.1
135.8
129.8
129.5
128.7
127.8
127.5
124.9
123.8
77.2 CDCl3
77.0 CDCl3
76.8 CDCl3
55.2
45.9
Se
N
O
4a
151 MHz, CDCl3
f1 (ppm)

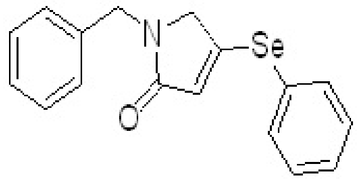

Qualitative Compound Identification Report

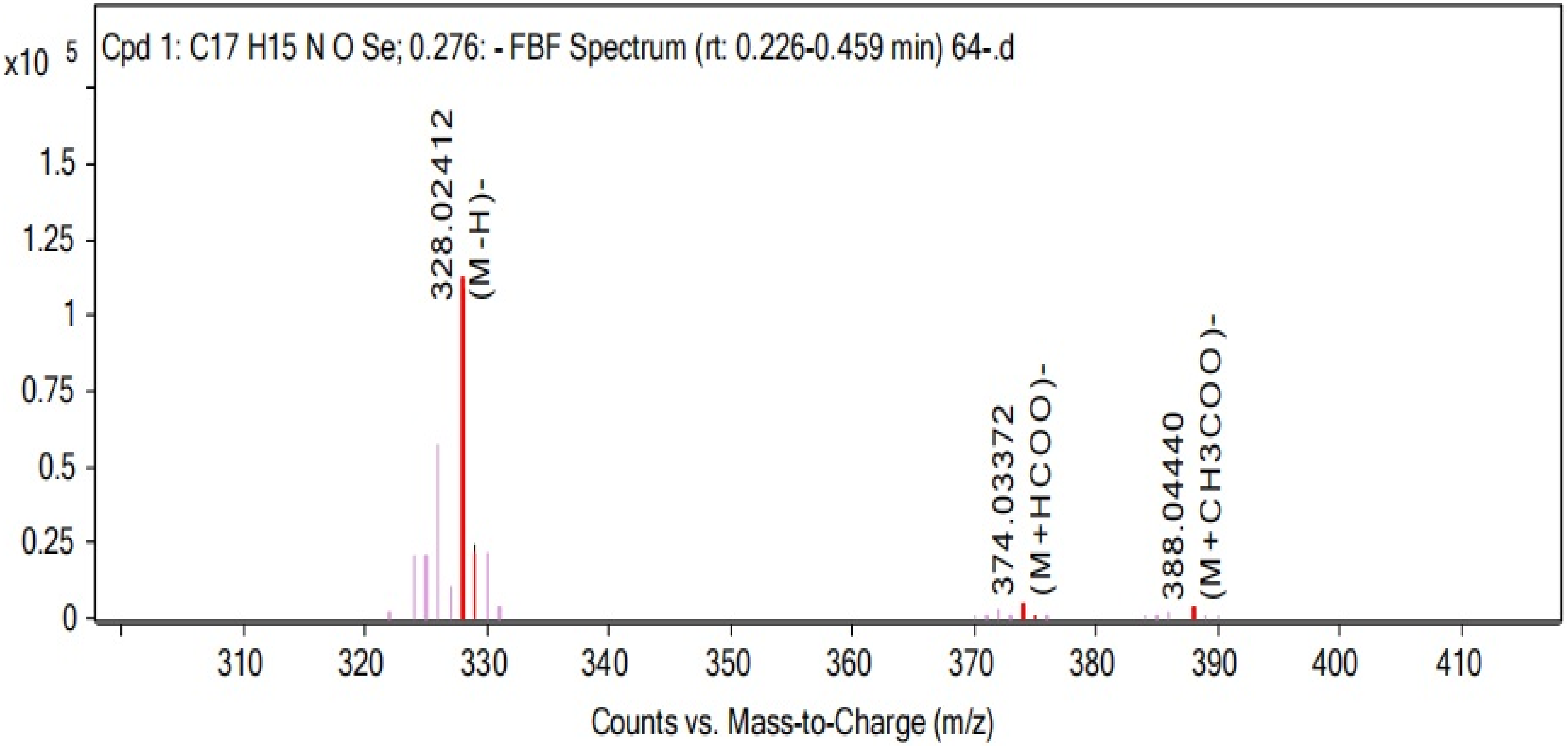

MS Spectrum Peak List

m/z	z	Abund	Ion
328.02412	1	108337.11	(M-H)-
329.02784	1	24893.69	(M-H)-
374.03372	1	5069.2	(M+HCOO)-
375.02964	1	1060.81	(M+HCOO)-
388.0444	1	3483.17	(M+CH3COO)-

MS Spectrum

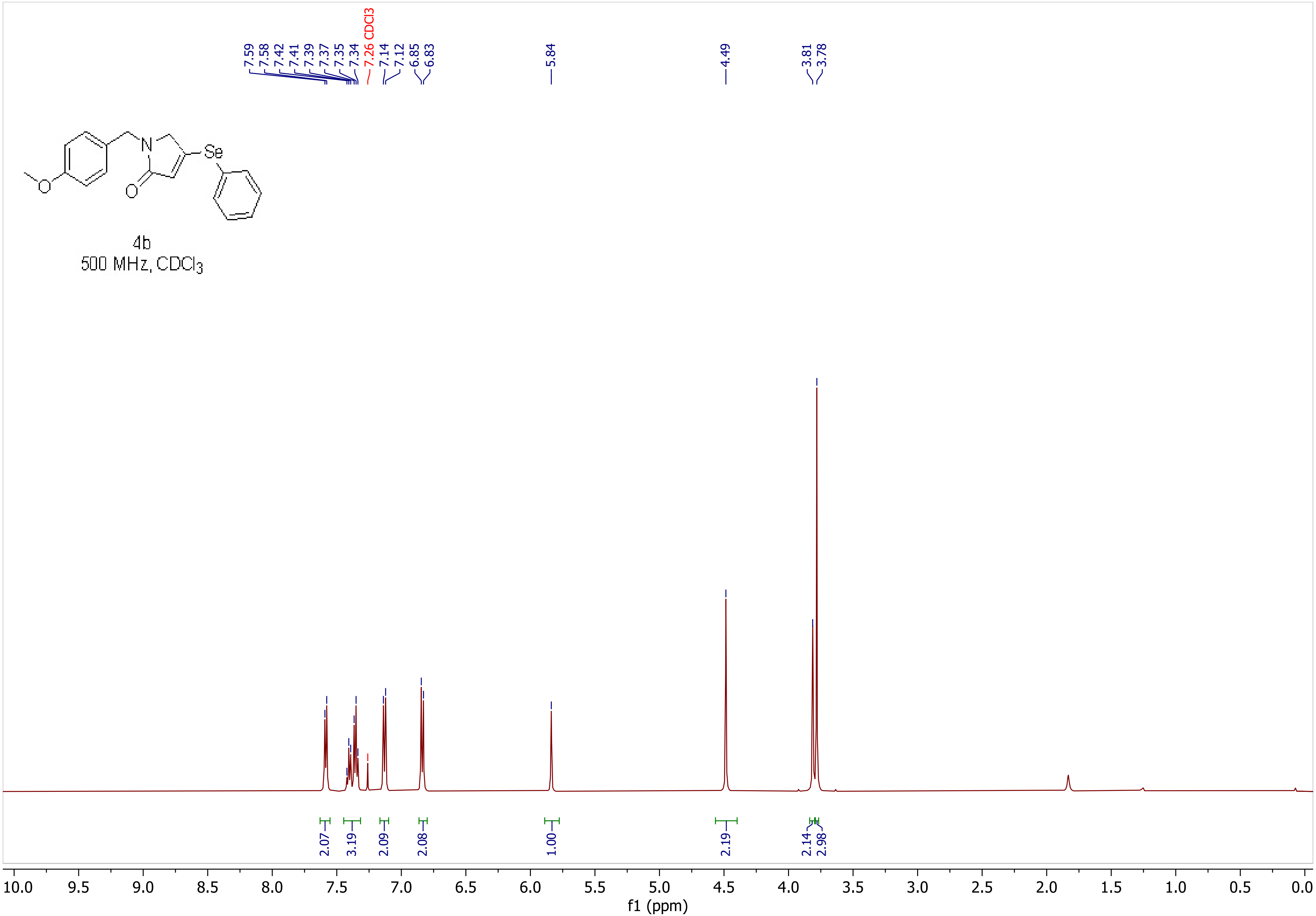
7.59
7.58
7.42
7.41
7.39
7.37
7.35
7.34
7.26 CDCl3
7.14
7.12
6.85
6.83
5.84
4.49
3.81
3.78
N
Se
O
O
4b
500 MHz, CDCl3
2.07
3.19
2.09
2.08
1.00
2.19
2.14
2.98
10.0
9.5
9.0
8.5
8.0
7.5
7.0
6.5
6.0
5.5
5.0
4.5
4.0
3.5
3.0
2.5
2.0
1.5
1.0
0.5
0.0
f1 (ppm)

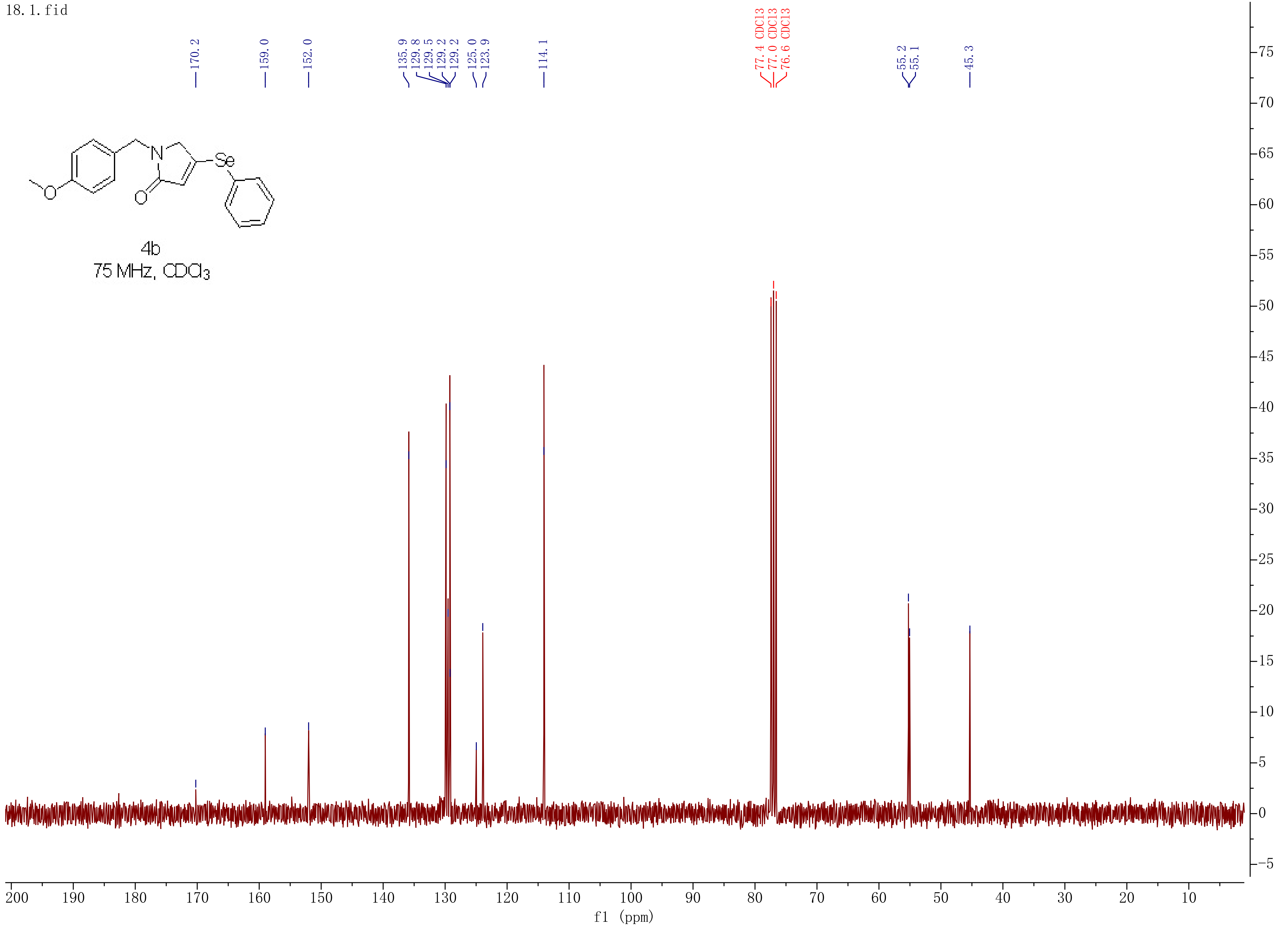
18.1.fid
170.2
159.0
152.0
135.9
129.8
129.5
129.2
129.2
125.0
123.9
114.1
77.4 CDCl3
77.0 CDCl3
76.6 CDCl3
55.2
55.1
45.3
N
Se
O
O
4b
75 MHz, CDCl3
f1 (ppm)

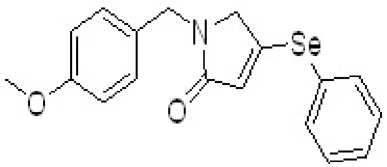

Qualitative Compound Identification Report

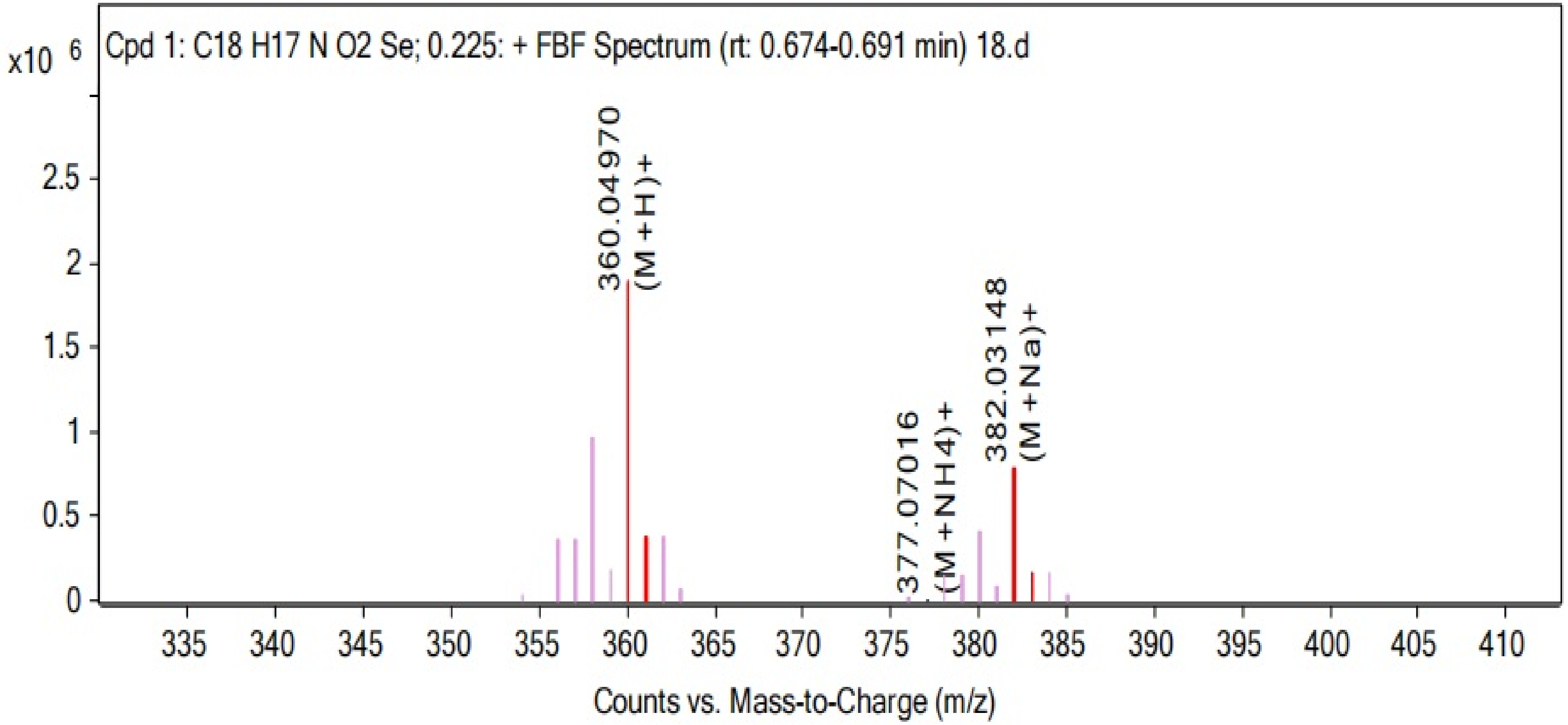

MS Spectrum Peak List

m/z	z	Abund	Ion
360.0497	1	1887032.25	(M+H)+
361.05318	1	384884.16	(M+H)+
377.07016	1	434.73	(M+NH4)+
382.03148	1	784757.75	(M+Na)+
383.03474	1	166834.73	(M+Na)+

MS Spectrum

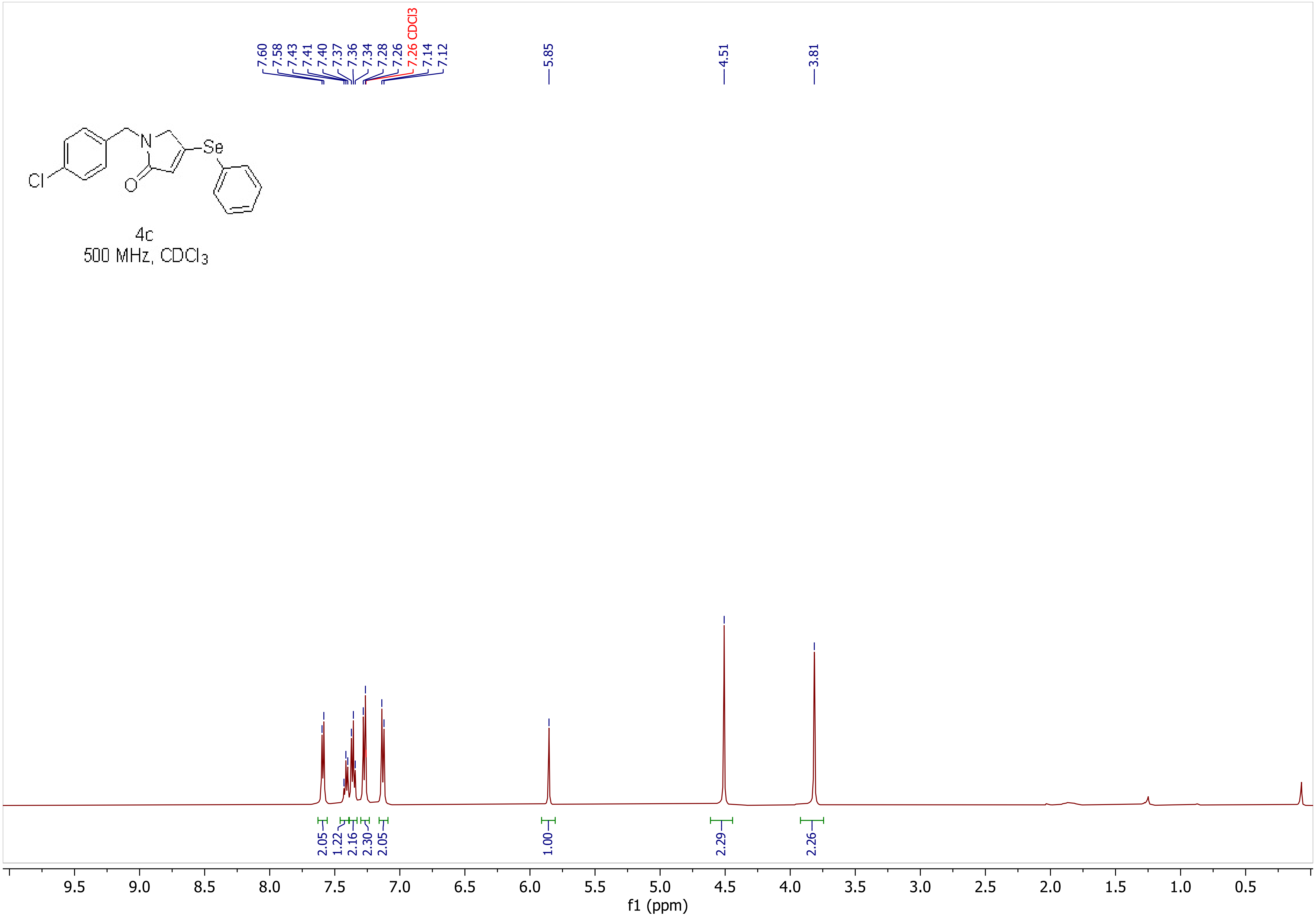

7.60
7.58
7.43
7.41
7.40
7.37
7.36
7.34
7.28
7.26
7.26 CDCl3
7.14
7.12
5.85
4.51
3.81
N
Se
Cl
O
4c
500 MHz, CDCl3
2.05
1.22
2.16
2.30
2.05
1.00
2.29
2.26
9.5
9.0
8.5
8.0
7.5
7.0
6.5
6.0
5.5
5.0
4.5
4.0
3.5
3.0
2.5
2.0
1.5
1.0
0.5
f1 (ppm)

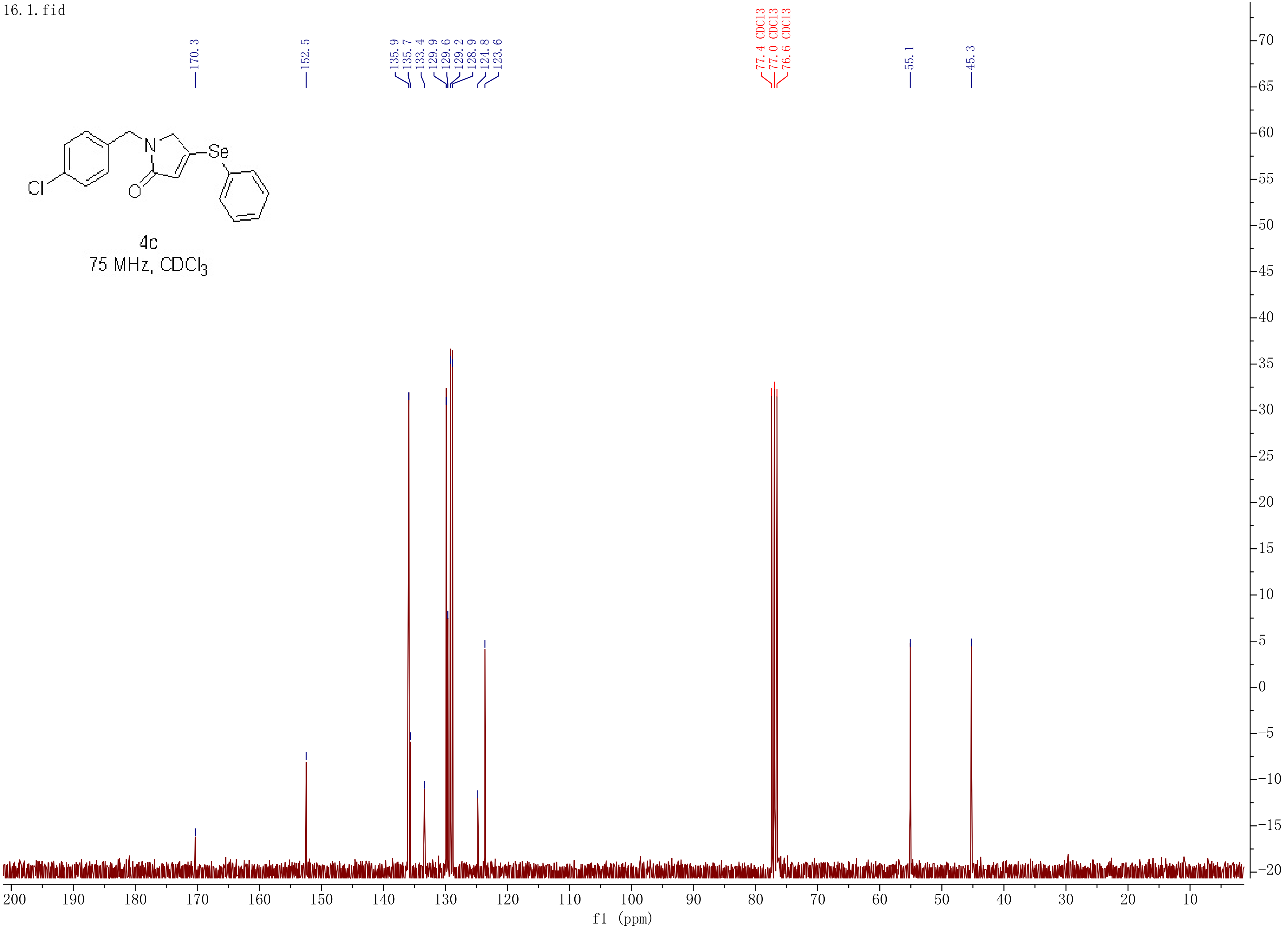

16.1.fid
170.3
152.5
135.9
135.7
133.4
129.9
129.6
129.2
128.9
124.8
123.6
77.4 CDC13
77.0 CDC13
76.6 CDC13
55.1
45.3
4c
75 MHz, CDCl3
f1 (ppm)

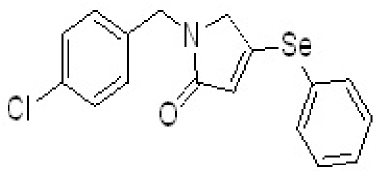

Qualitative Compound Identification Report

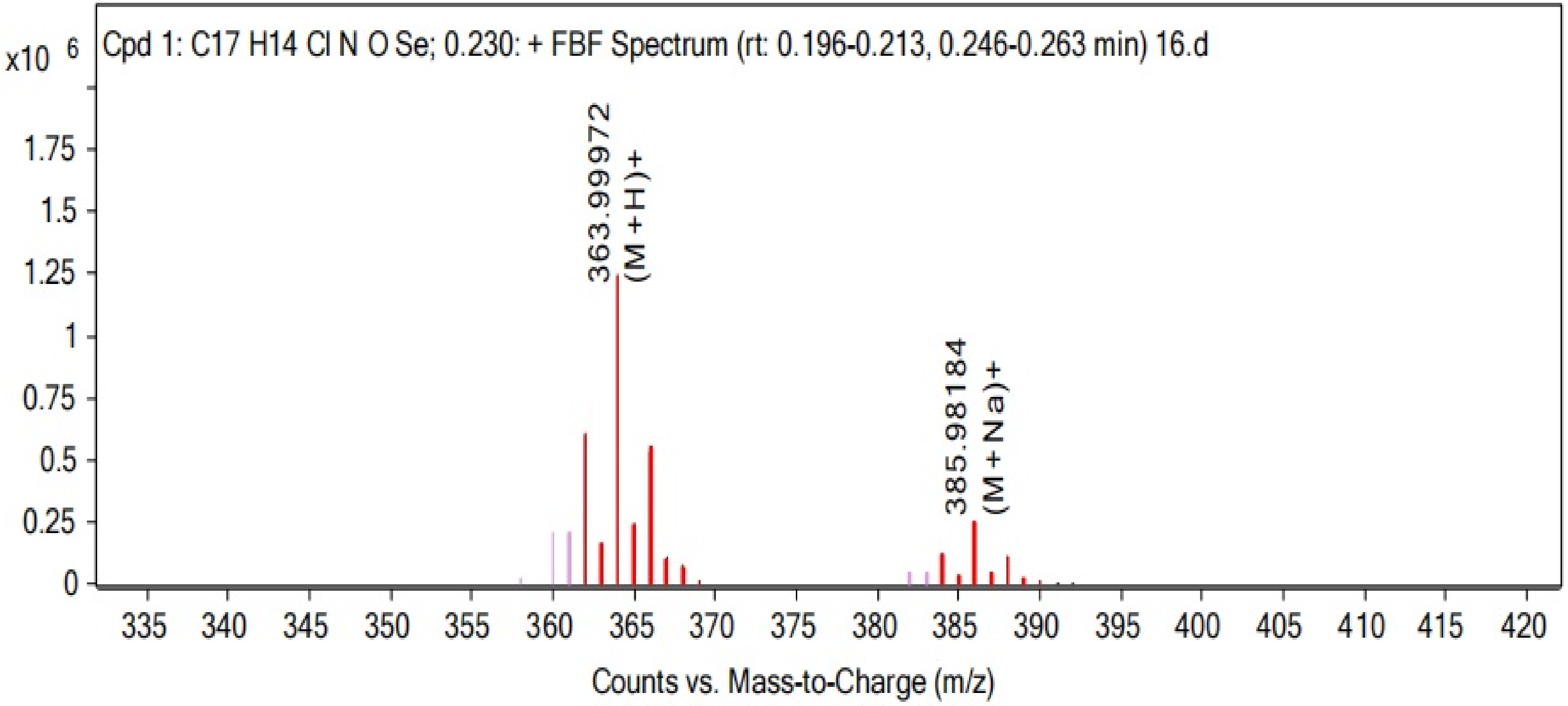

MS Spectrum Peak List

m/z	z	Abund	Ion
362.00069	1	596454.94	(M+H)+
363.99972	1	1246450.25	(M+H)+
365.00299	1	234039	(M+H)+
365.99814	1	527390.56	(M+H)+
385.98184	1	247638.63	(M+Na)+

MS Spectrum

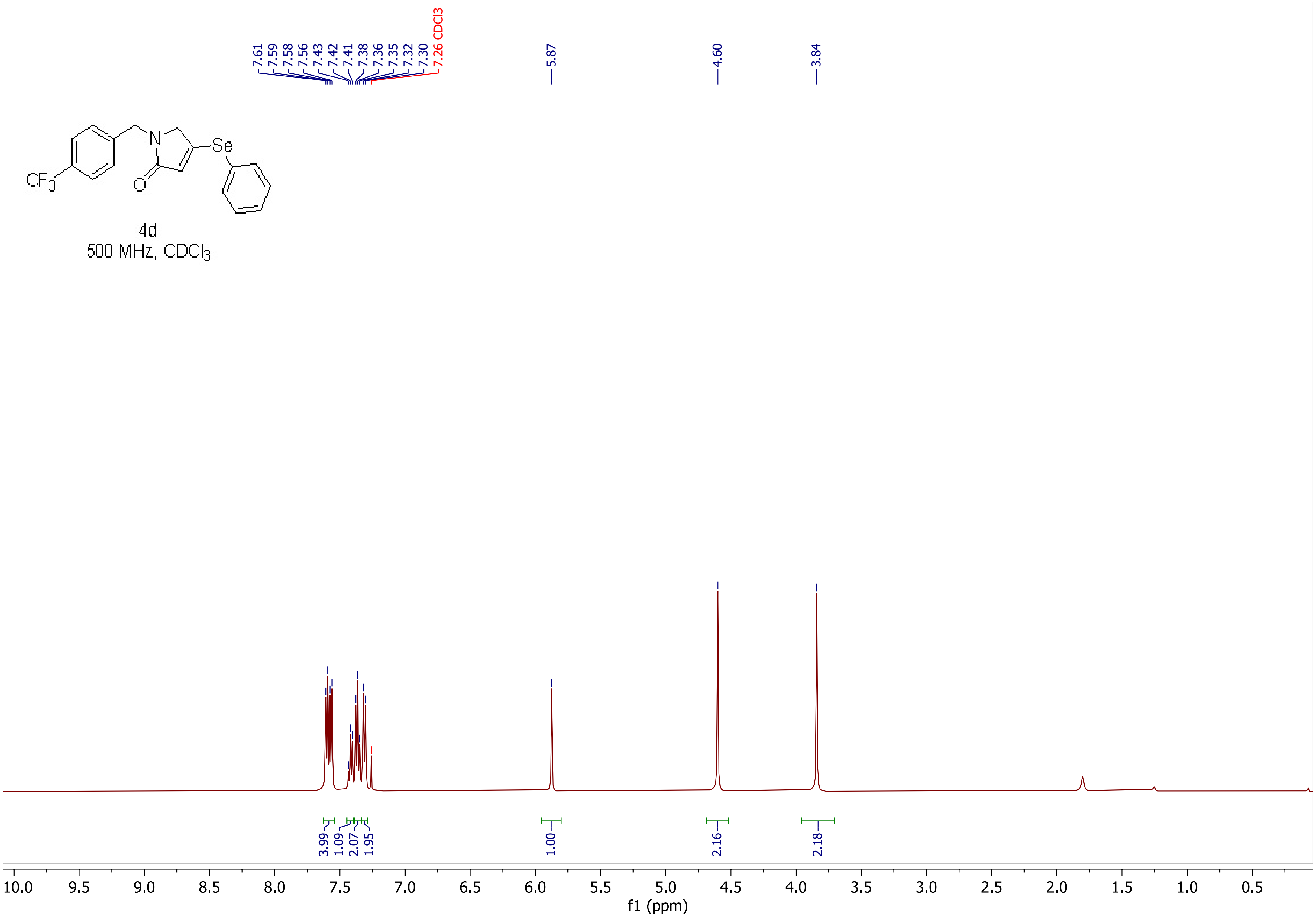

7.61
7.59
7.58
7.56
7.43
7.42
7.41
7.38
7.36
7.35
7.32
7.30
7.26 CDCl3
5.87
4.60
3.84
CF3
N
Se
O
4d
500 MHz, CDCl3
3.99
1.09
2.07
1.95
1.00
2.16
2.18
10.0
9.5
9.0
8.5
8.0
7.5
7.0
6.5
6.0
5.5
5.0
4.5
4.0
3.5
3.0
2.5
2.0
1.5
1.0
0.5
f1 (ppm)

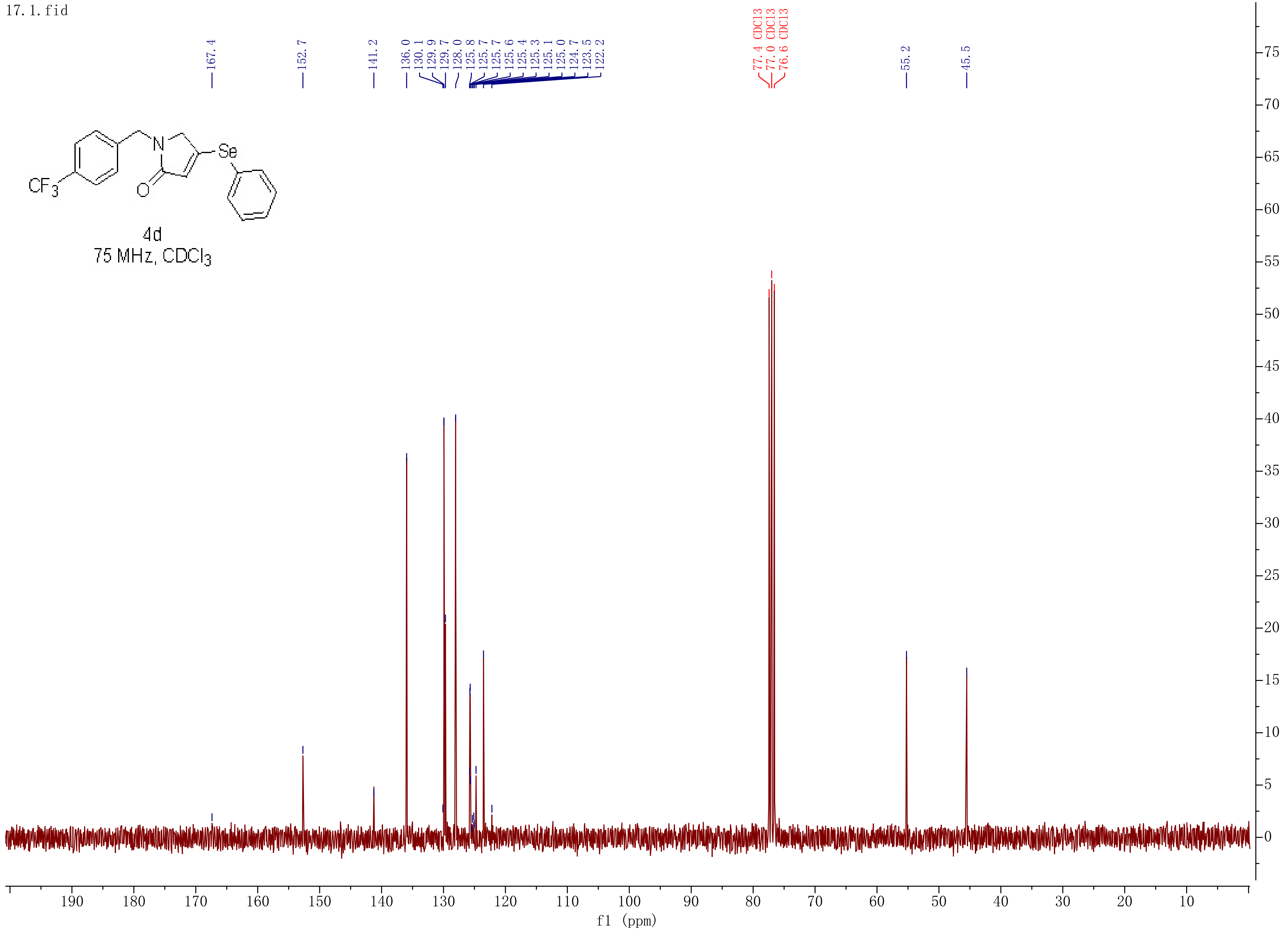
17.1.fid
167.4
152.7
141.2
136.0
130.1
129.9
129.7
128.0
125.8
125.7
125.7
125.6
125.4
125.3
125.1
125.0
124.7
123.5
122.2
77.4 CDCl3
77.0 CDCl3
76.6 CDCl3
55.2
45.5
CF_3
N
Se
O
4d
75 MHz, $CDCl_3$
f1 (ppm)

1023-17.1.fid

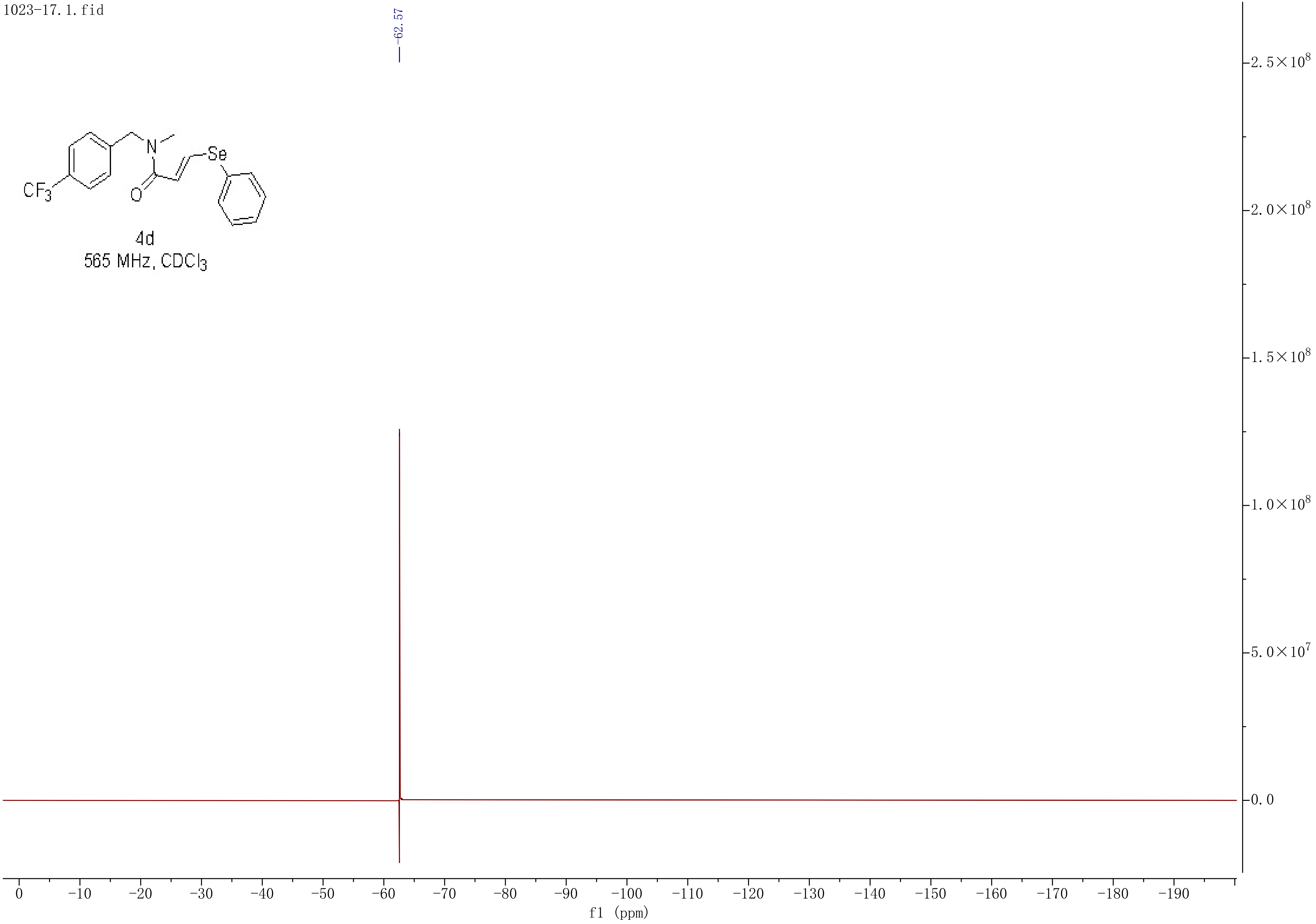

CF3
N
Se
O
4d
565 MHz, CDCl3
-62.57
2.5×10^8
2.0×10^8
1.5×10^8
1.0×10^8
5.0×10^7
0.0
0
-10
-20
-30
-40
-50
-60
-70
-80
-90
-100
-110
-120
-130
-140
-150
-160
-170
-180
-190
f1 (ppm)

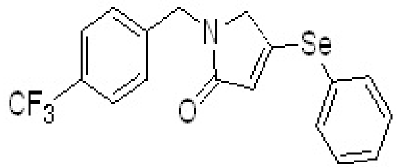

Qualitative Compound Identification Report

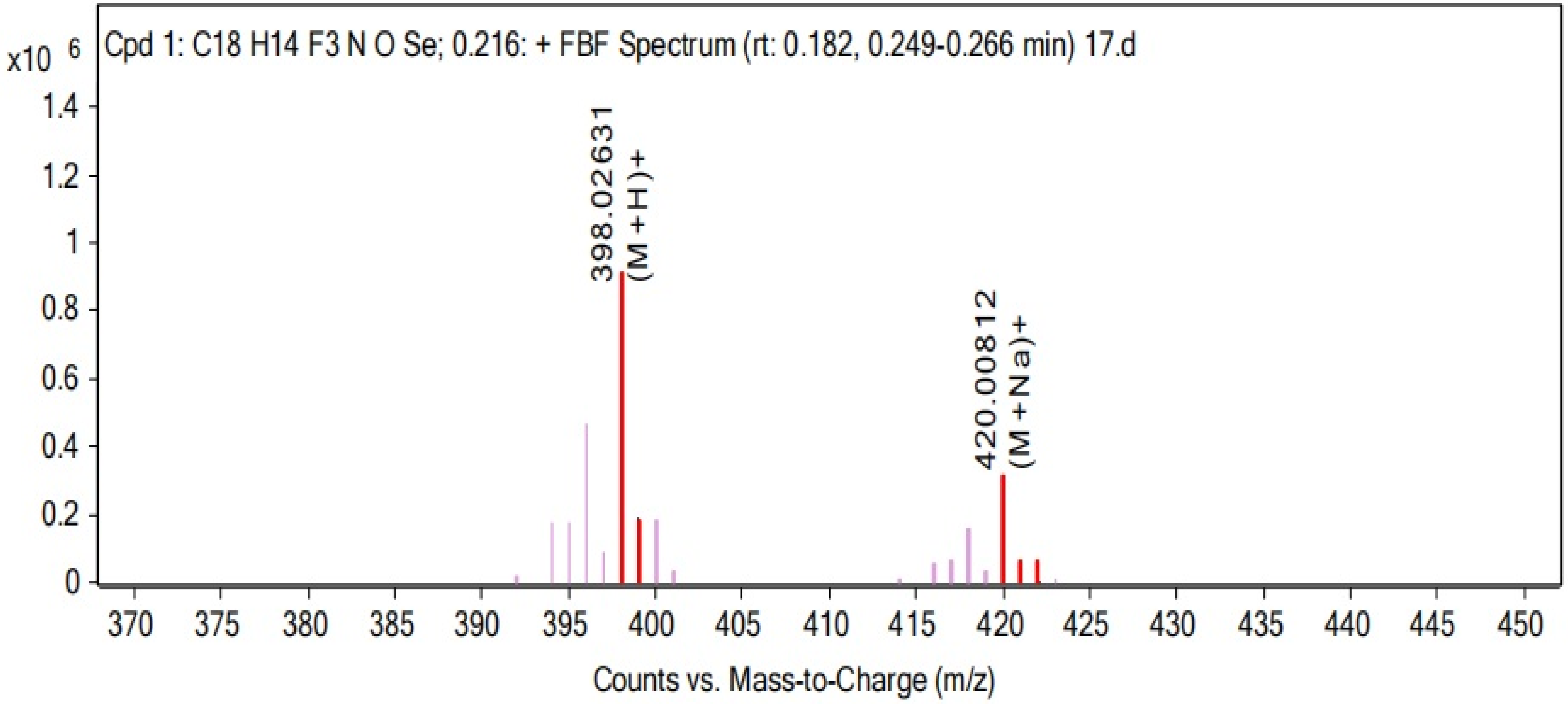

MS Spectrum Peak List

m/z	z	Abund	Ion
398.02631	1	907159.75	(M+H)+
399.02967	1	185888.19	(M+H)+
420.00812	1	315107.41	(M+Na)+
421.01155	1	64673.91	(M+Na)+
422.01499	1	329.92	(M+Na)+

MS Spectrum

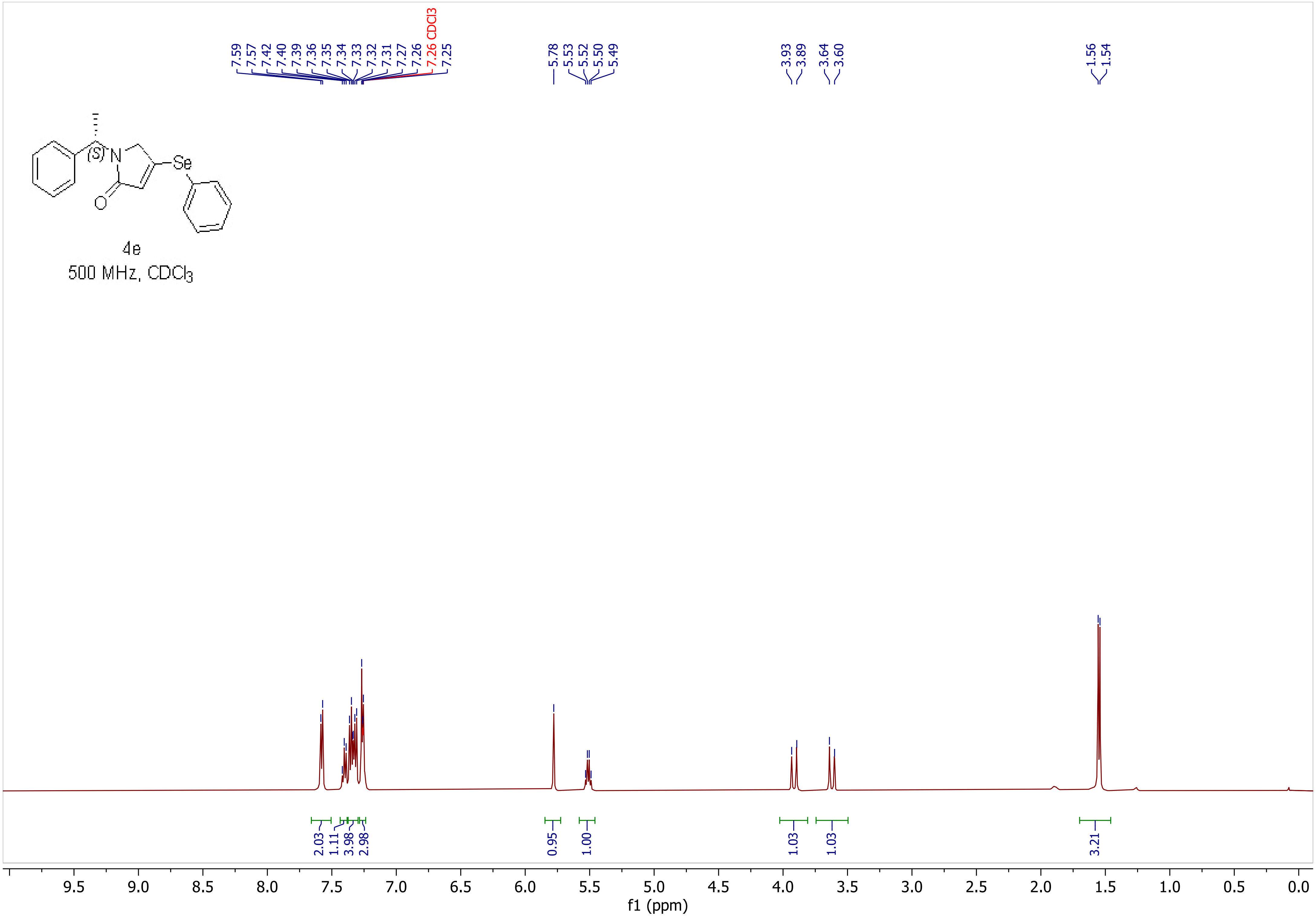
7.59
7.57
7.42
7.40
7.39
7.36
7.35
7.34
7.33
7.32
7.31
7.27
7.26
7.26 CDCl3
7.25
5.78
5.53
5.52
5.50
5.49
3.93
3.89
3.64
3.60
1.56
1.54
(S)
N
Se
O
4e
500 MHz, CDCl3
2.03
1.11
3.98
2.98
0.95
1.00
1.03
1.03
3.21
9.5
9.0
8.5
8.0
7.5
7.0
6.5
6.0
5.5
5.0
4.5
4.0
3.5
3.0
2.5
2.0
1.5
1.0
0.5
0.0
f1 (ppm)

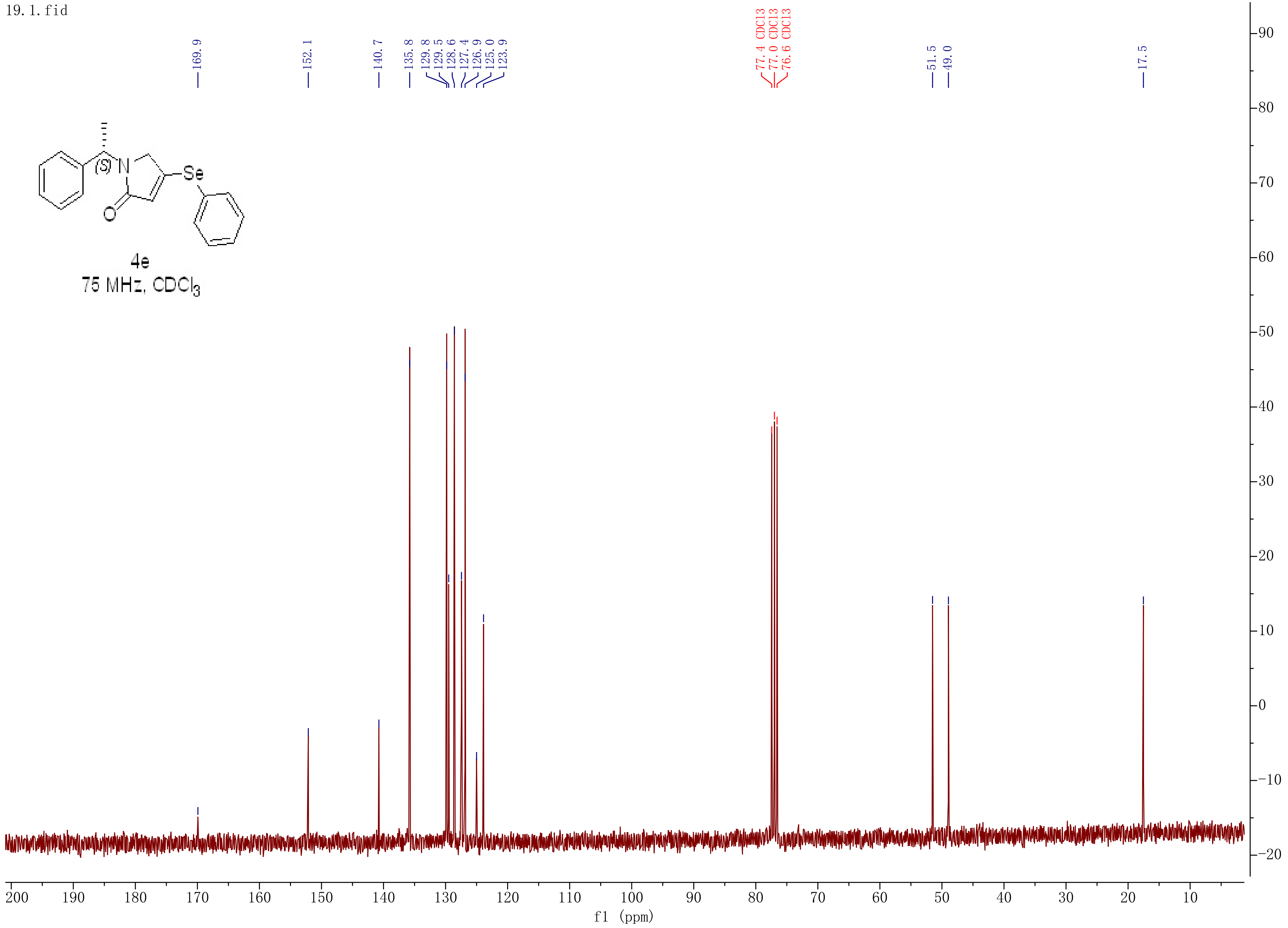
19.1.fid
169.9
152.1
140.7
135.8
129.8
129.5
128.6
127.4
126.9
125.0
123.9
77.4 CDCl3
77.0 CDCl3
76.6 CDCl3
51.5
49.0
17.5
(S)
N
Se
O
4e
75 MHz, CDCl3
f1 (ppm)

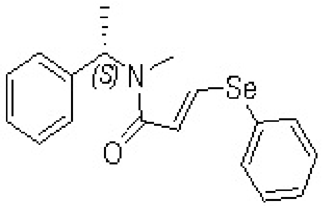

Qualitative Compound Identification Report

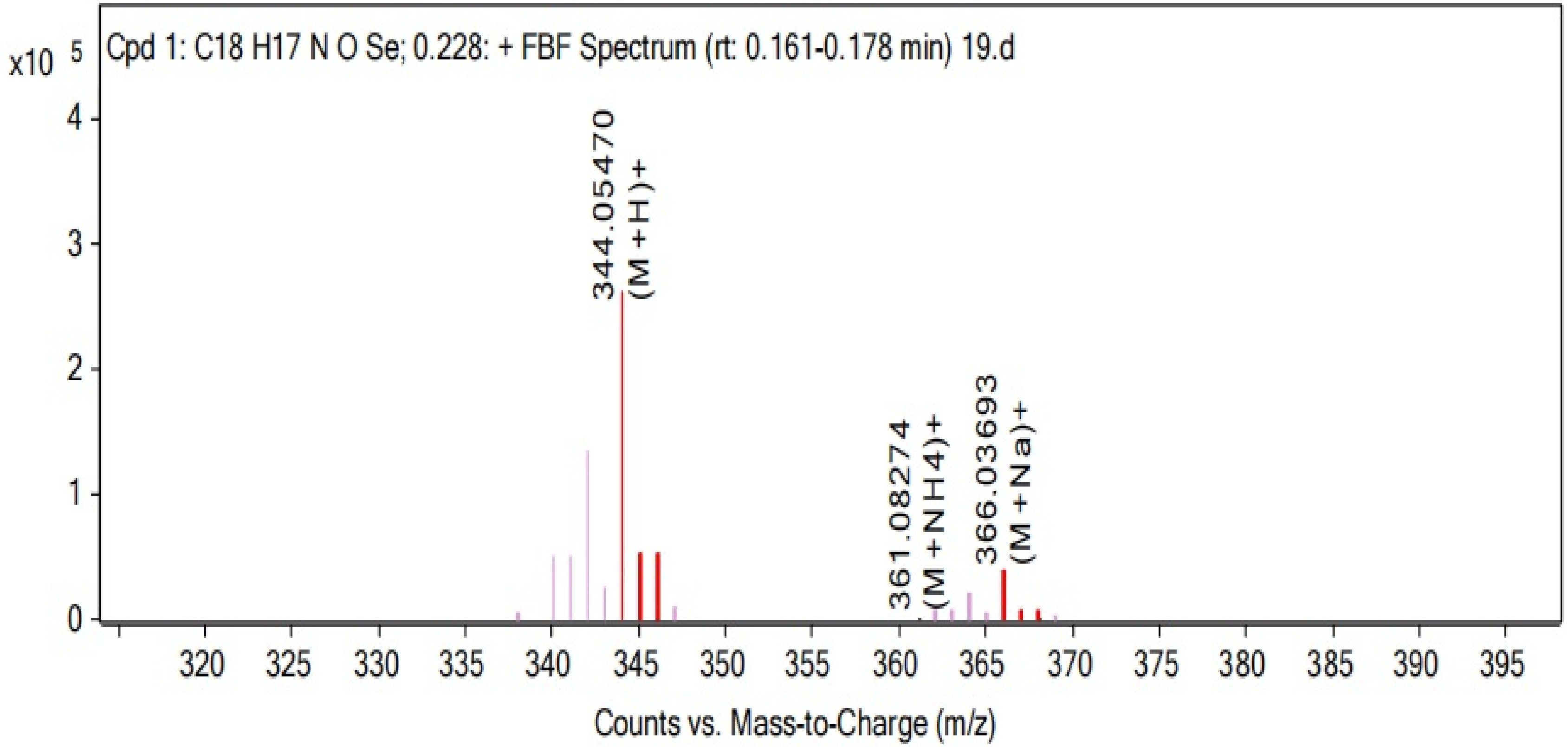

MS Spectrum Peak List

m/z	z	Abund	Ion
344.0547	1	261869.66	(M+H)+
345.05811	1	45969.41	(M+H)+
366.03693	1	38652.4	(M+Na)+
367.04054	1	7348.28	(M+Na)+
368.05217	1	1239.28	(M+Na)+

MS Spectrum

Y-0824-8-H.1.fid

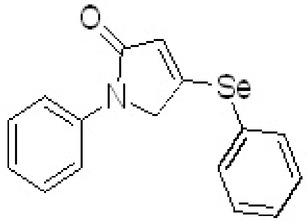

4f
500 MHz, $CDCl_3$

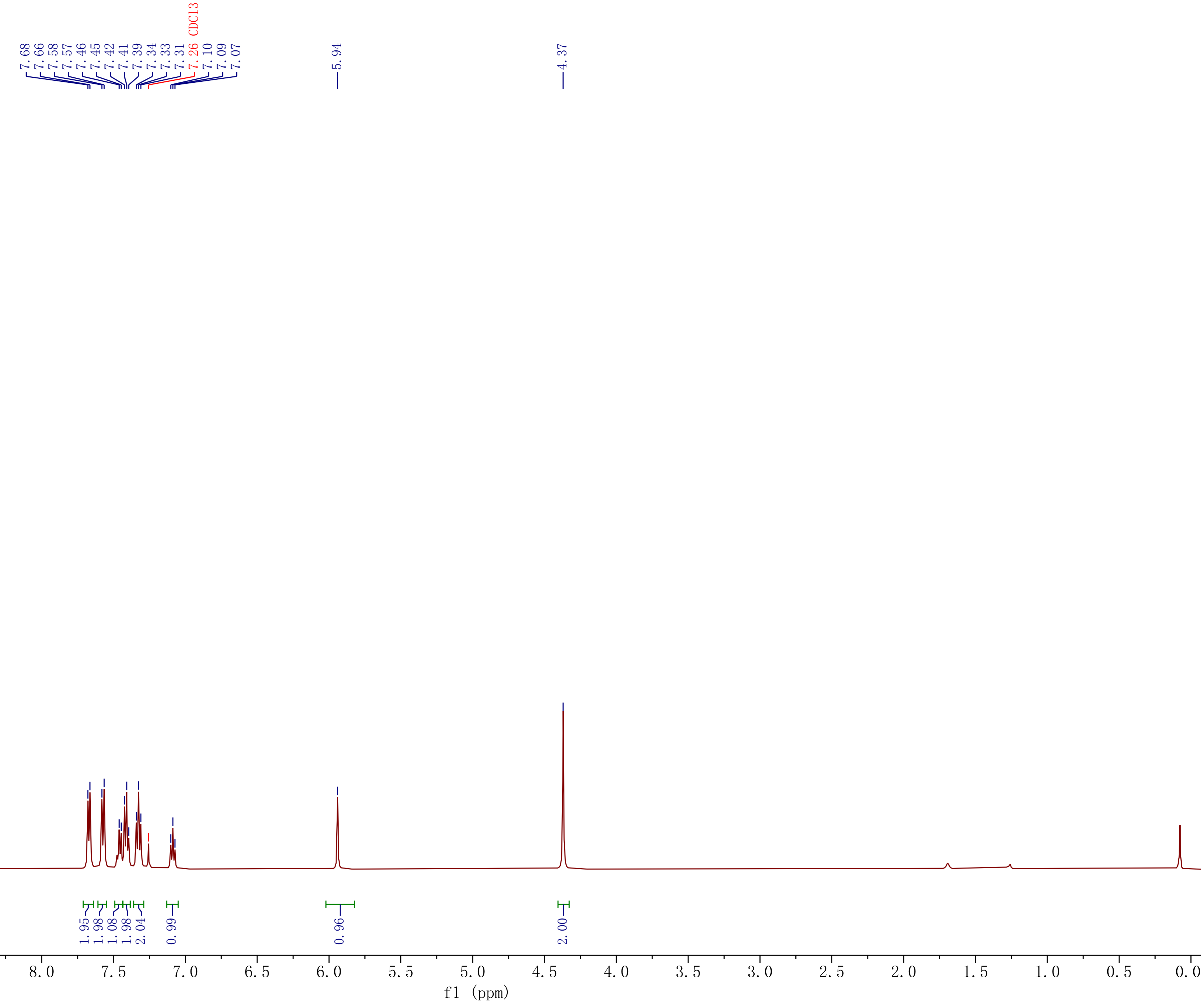

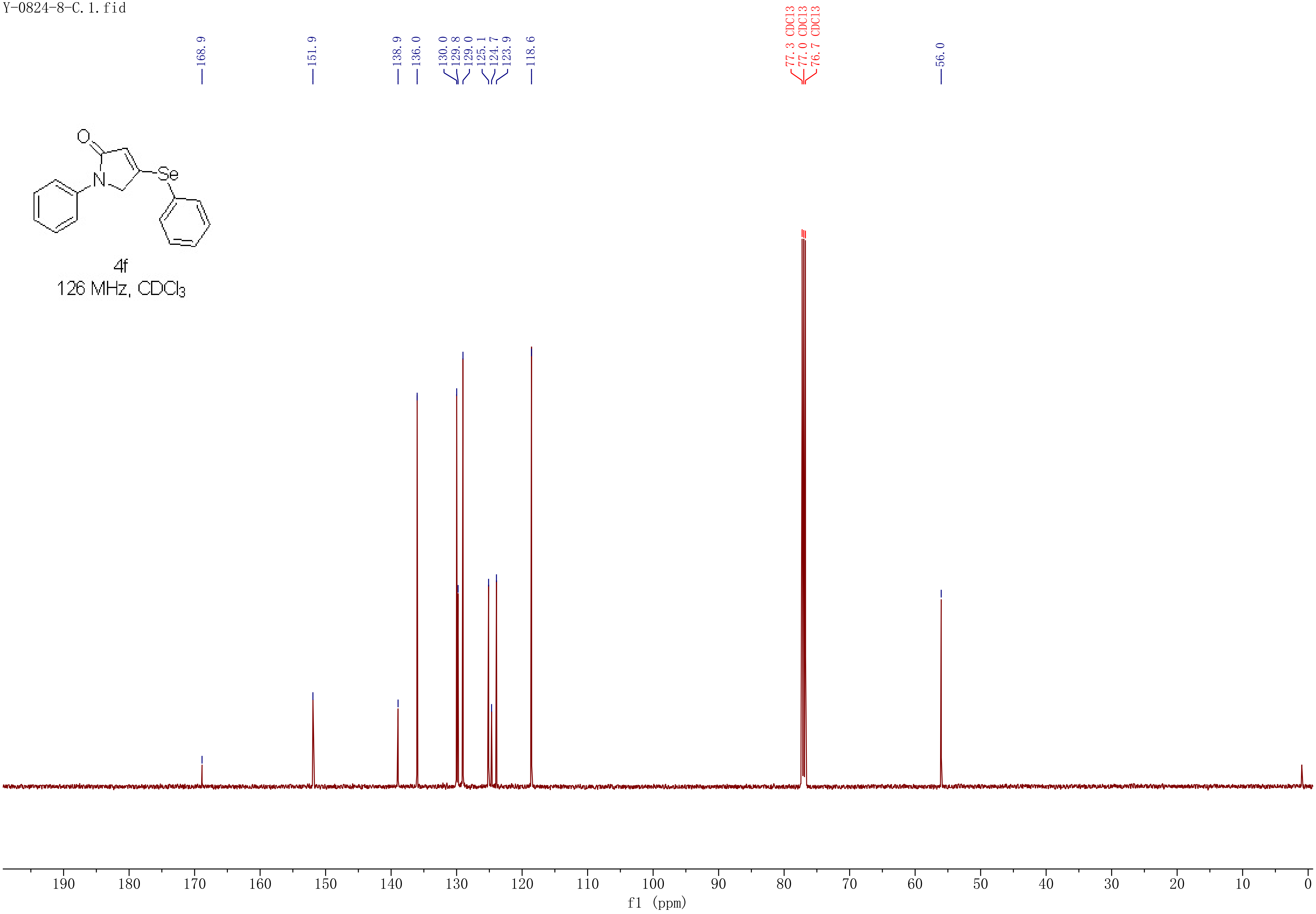
Y-0824-8-C.1.fid
168.9
151.9
138.9
136.0
130.0
129.8
129.0
125.1
124.7
123.9
118.6
77.3 CDCl3
77.0 CDCl3
76.7 CDCl3
56.0
O
N
Se
4f
126 MHz, $CDCl_3$
190 180 170 160 150 140 130 120 110 100 90 80 70 60 50 40 30 20 10 0
f1 (ppm)

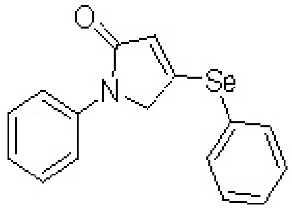

Qualitative Compound Identification Report

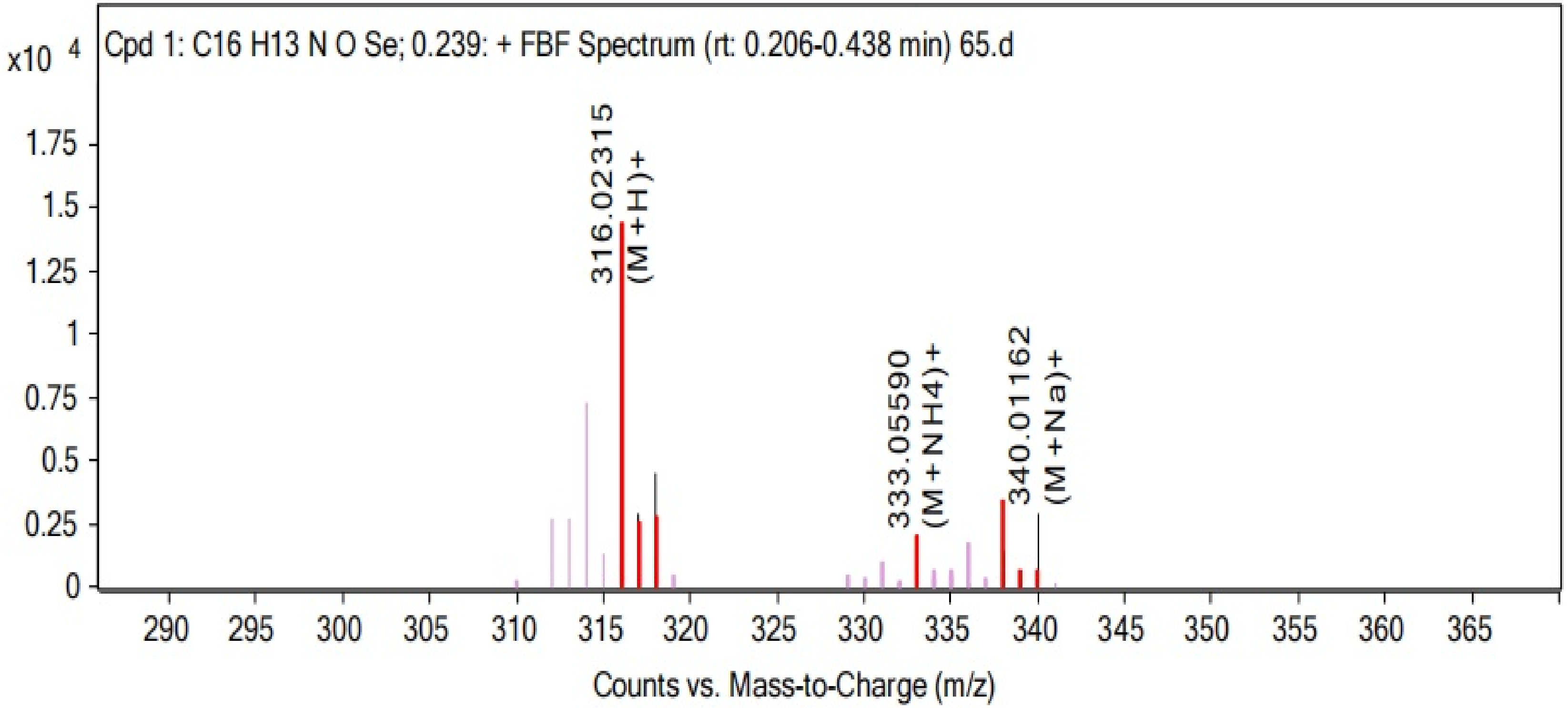

MS Spectrum Peak List

m/z	z	Abund	Ion
316.02315	1	12386.82	(M+H)+
317.02675	1	2853.21	(M+H)+
318.02371	1	4529.85	(M+H)+
333.0559	1	1996.03	(M+NH4)+
338.00824	1	1442.01	(M+Na)+
339.01245	1	274.68	(M+Na)+
340.01162	1	2923.9	(M+Na)+

MS Spectrum

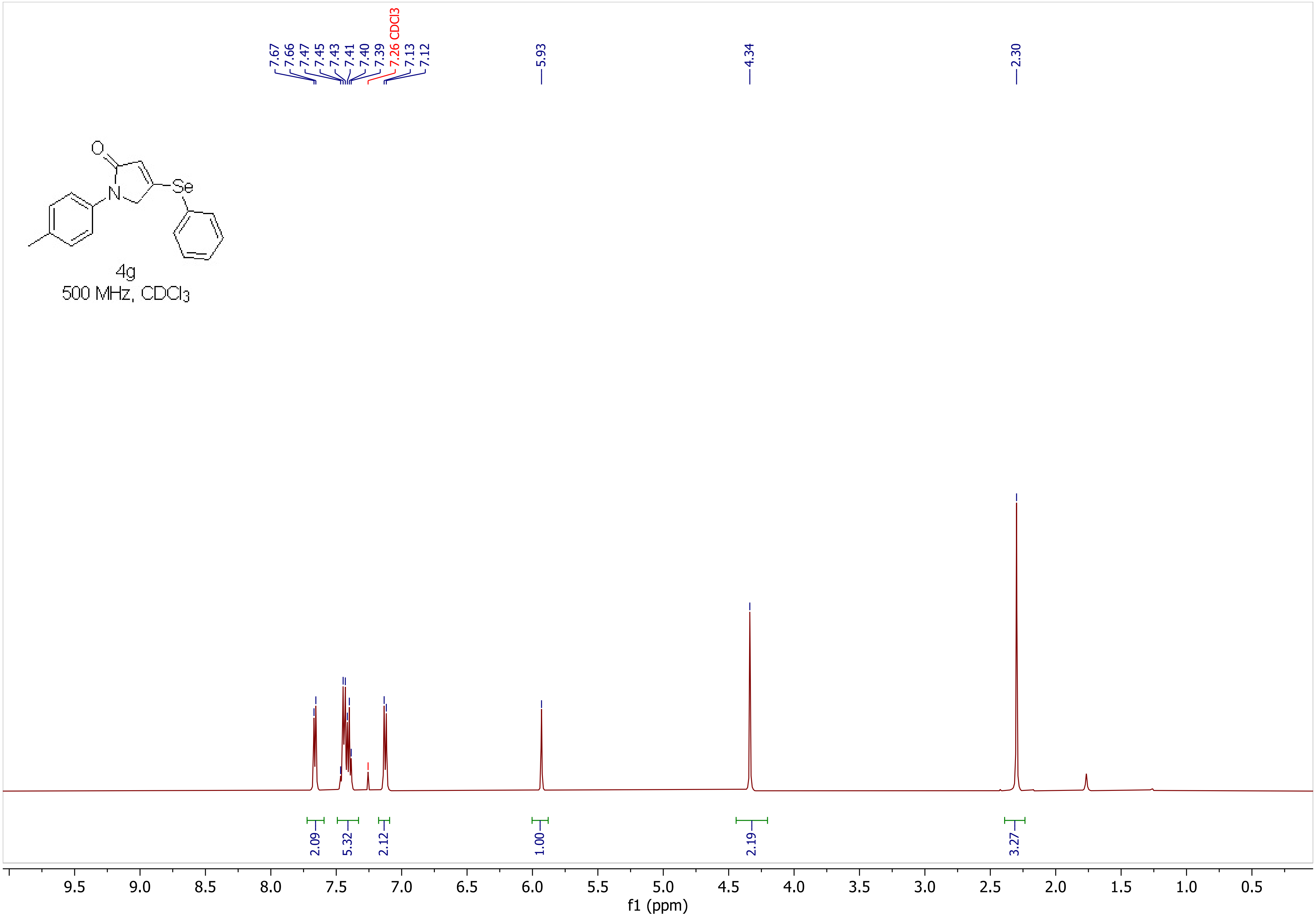

7.67
7.66
7.47
7.45
7.43
7.41
7.40
7.39
7.26 CDCl3
7.13
7.12
5.93
4.34
2.30
O
N
Se
4g
500 MHz, CDCl3
2.09
5.32
2.12
1.00
2.19
3.27
9.5
9.0
8.5
8.0
7.5
7.0
6.5
6.0
5.5
5.0
4.5
4.0
3.5
3.0
2.5
2.0
1.5
1.0
0.5
f1 (ppm)

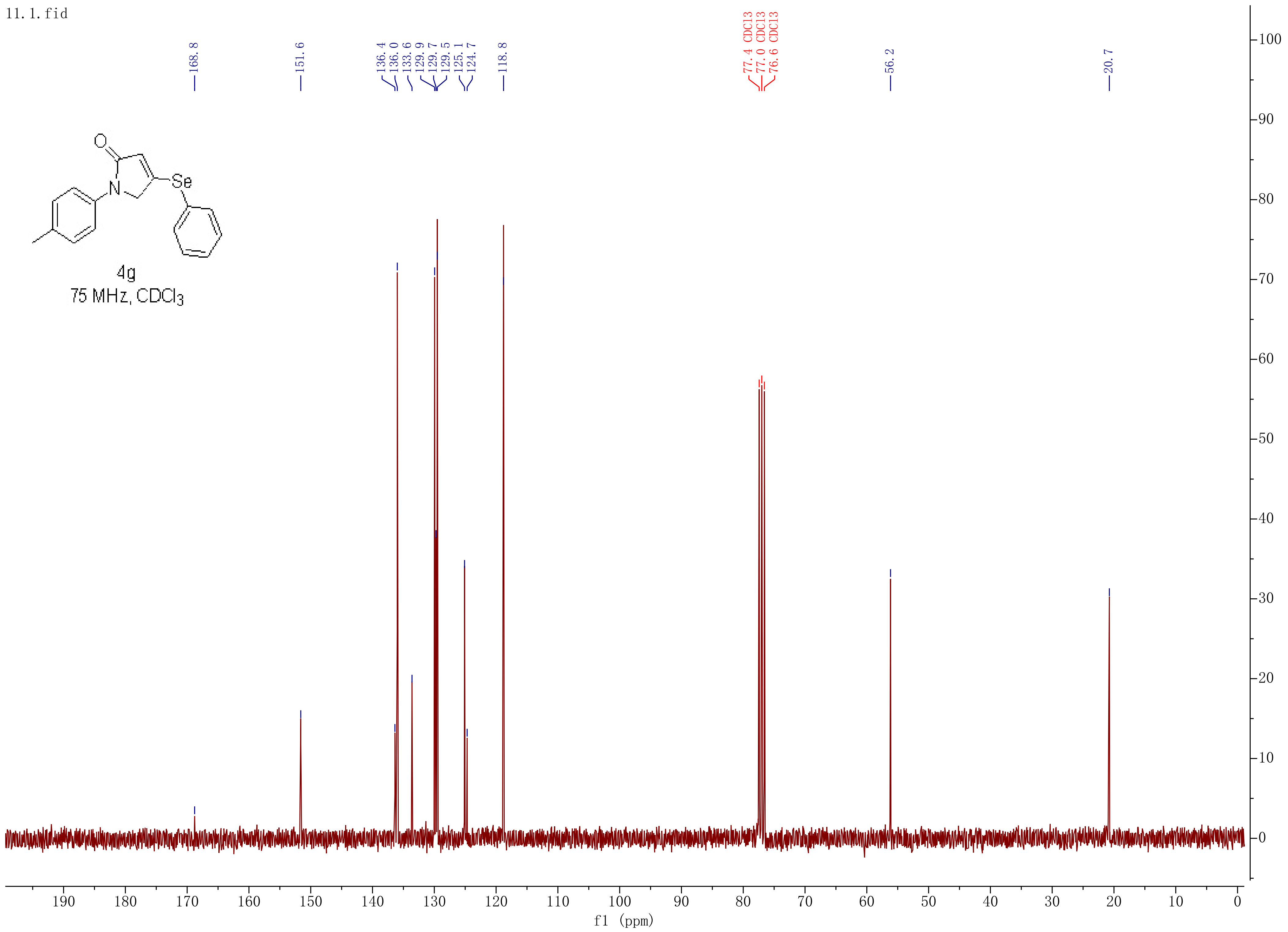

11.1.fid
168.8
151.6
136.4
136.0
133.6
129.9
129.7
129.5
125.1
124.7
118.8
77.4 CDCl3
77.0 CDCl3
76.6 CDCl3
56.2
20.7
O
N
Se
4g
75 MHz, CDCl3
f1 (ppm)

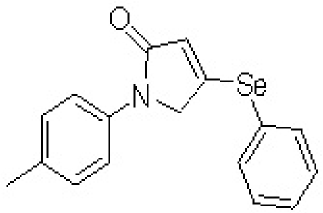

Qualitative Compound Identification Report

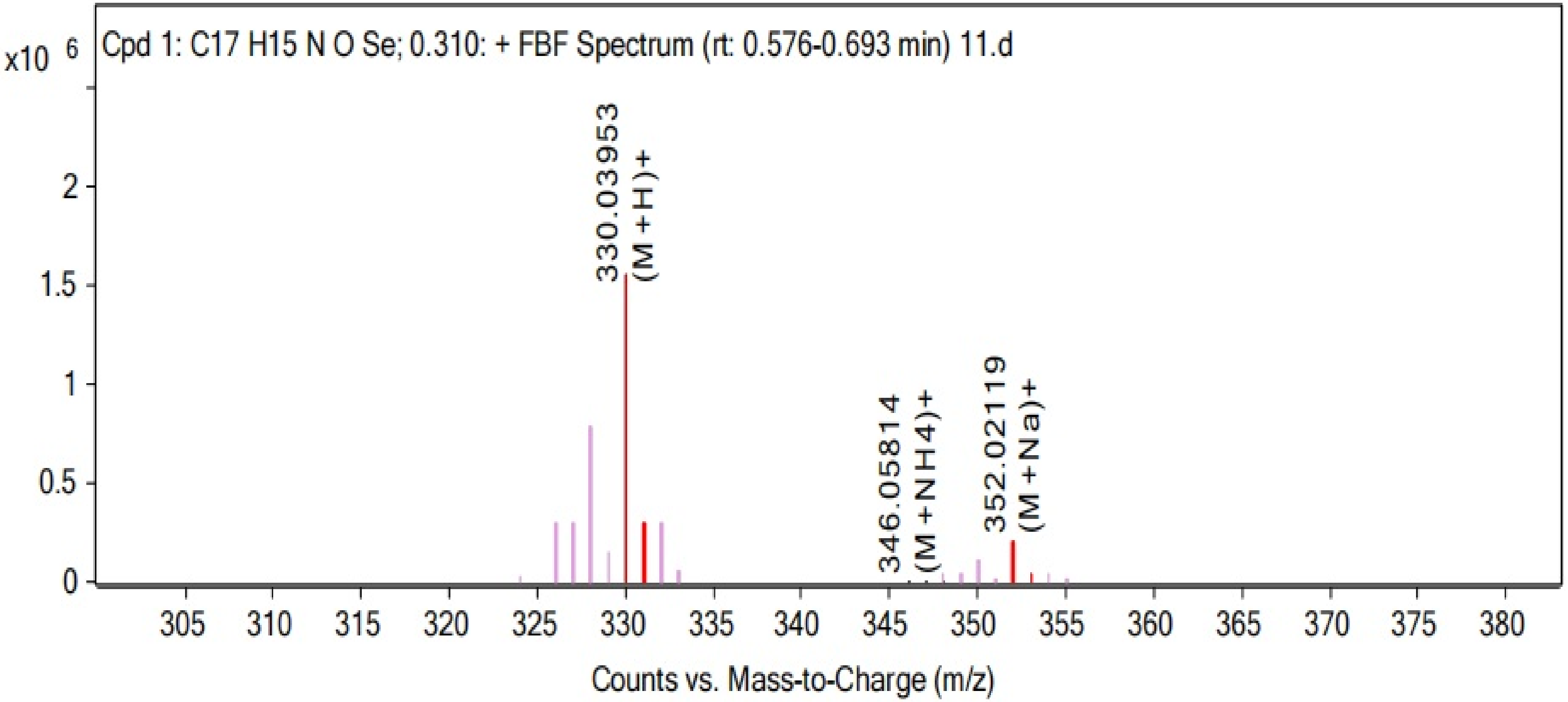

MS Spectrum Peak List

m/z	z	Abund	Ion
330.03953	1	1557910.88	(M+H)+
331.04256	1	293718.13	(M+H)+
346.05814	1	1184.69	(M+NH4)+
352.02119	1	203851	(M+Na)+
353.02452	1	43422.74	(M+Na)+

MS Spectrum

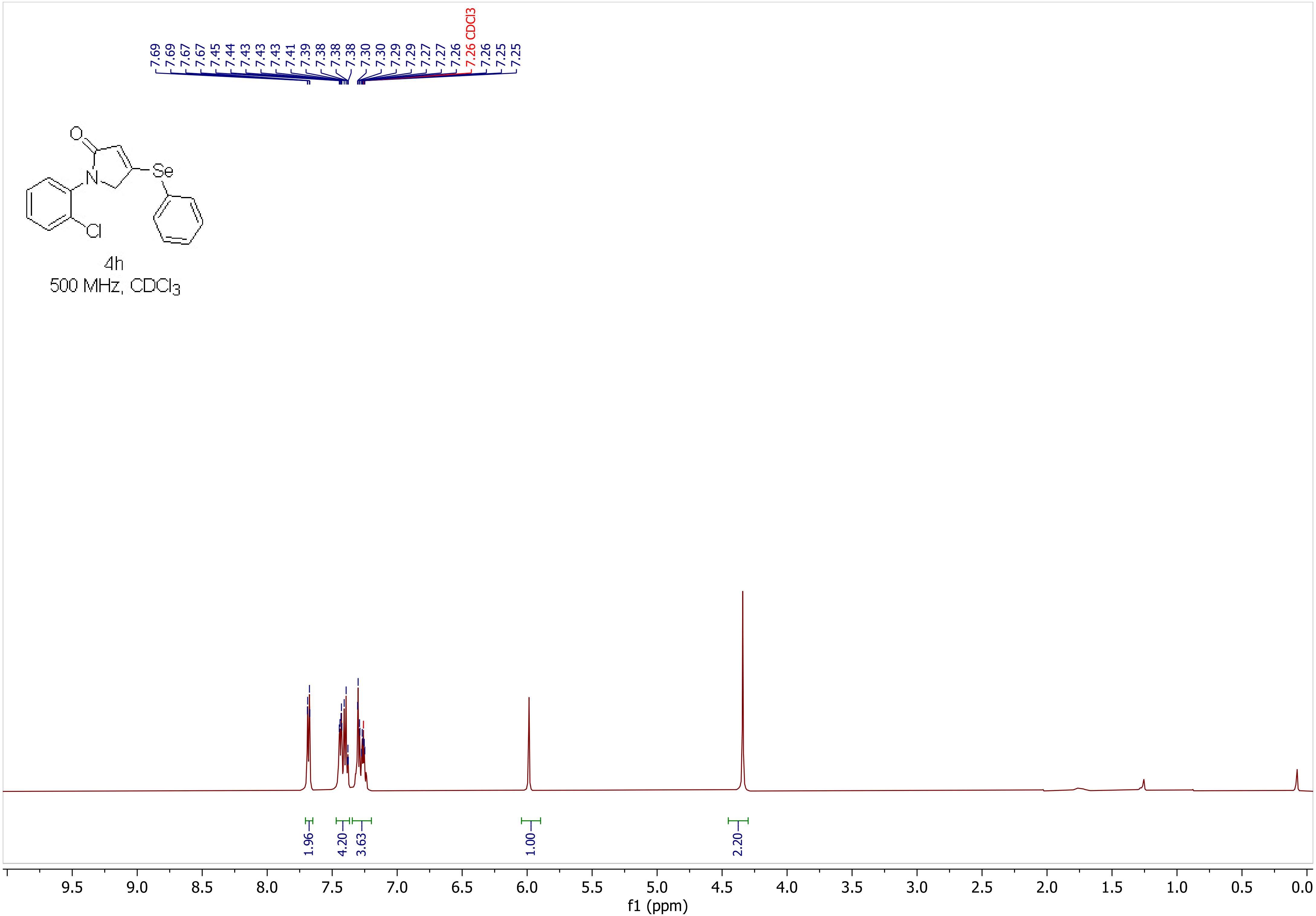

4h

500 MHz, $CDCl_3$

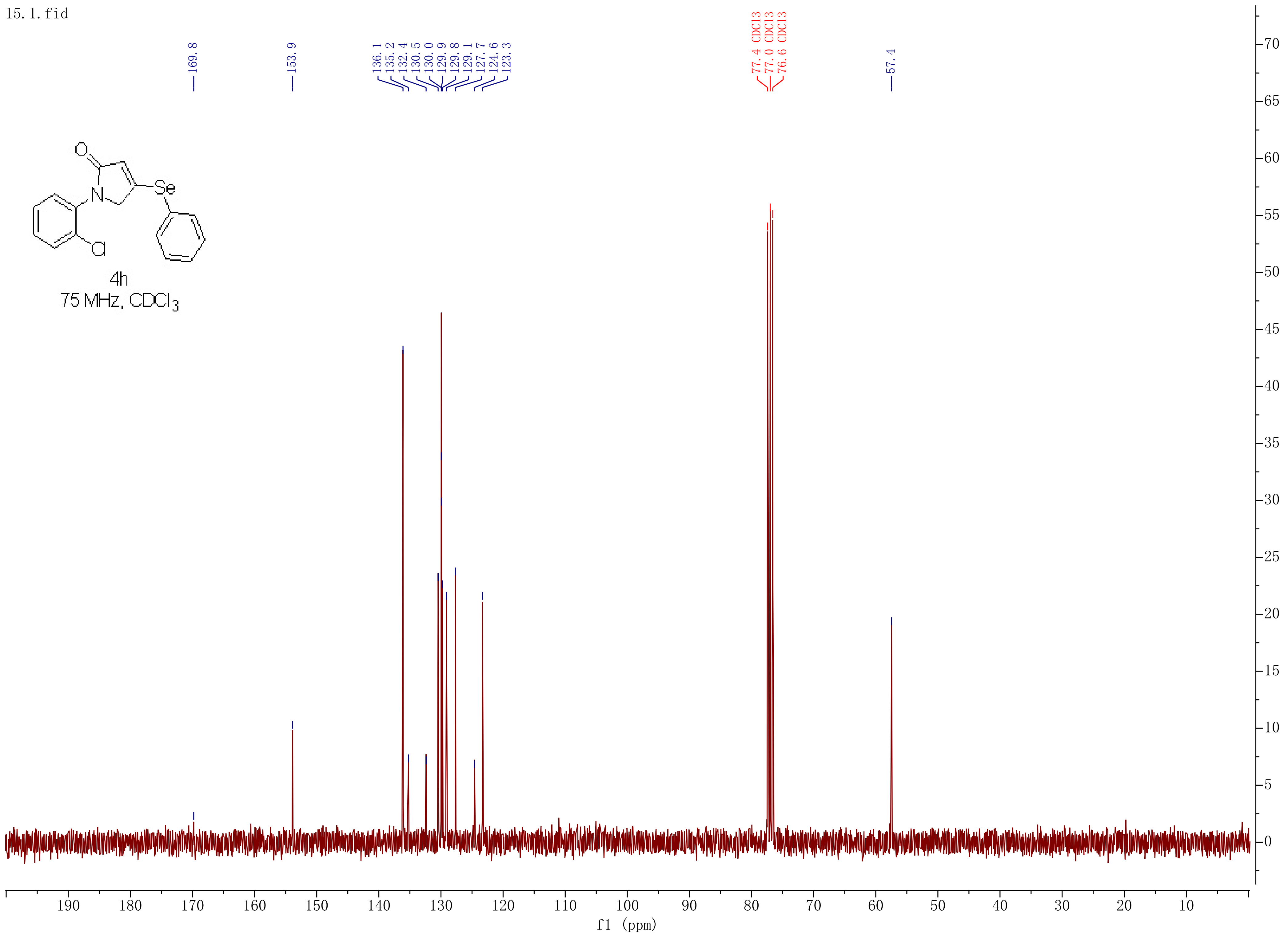

15.1.fid
169.8
153.9
136.1
135.2
132.4
130.5
130.0
129.9
129.8
129.1
127.7
124.6
123.3
77.4 CDCl3
77.0 CDCl3
76.6 CDCl3
57.4
O
N
Se
Cl
4h
75 MHz, CDCl3
f1 (ppm)

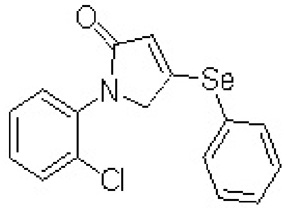

Qualitative Compound Identification Report

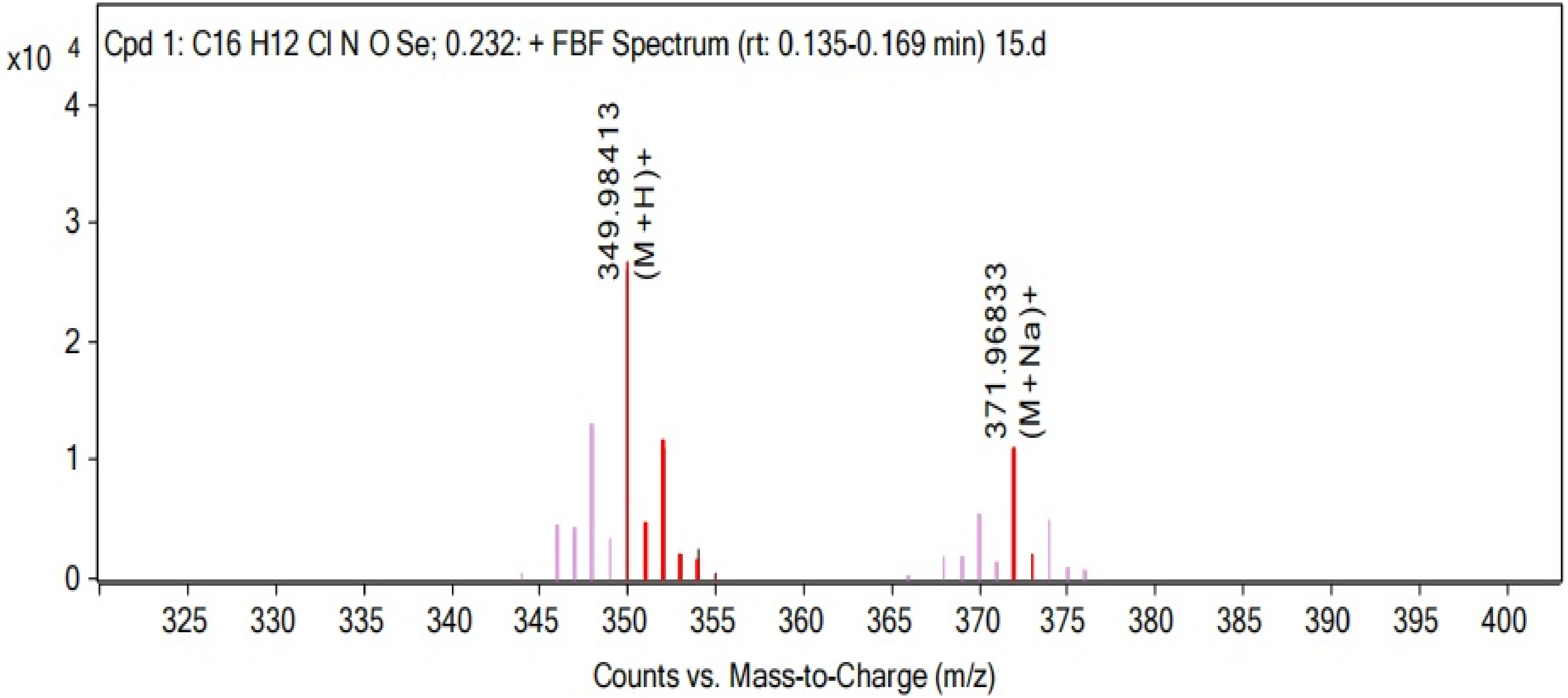

MS Spectrum Peak List

m/z	z	Abund	Ion
349.98413	1	26014.18	(M+H)+
350.98626	1	4673.59	(M+H)+
351.98297	1	11040.84	(M+H)+
353.98387	1	2510.04	(M+H)+
371.96833	1	11027.98	(M+Na)+

MS Spectrum

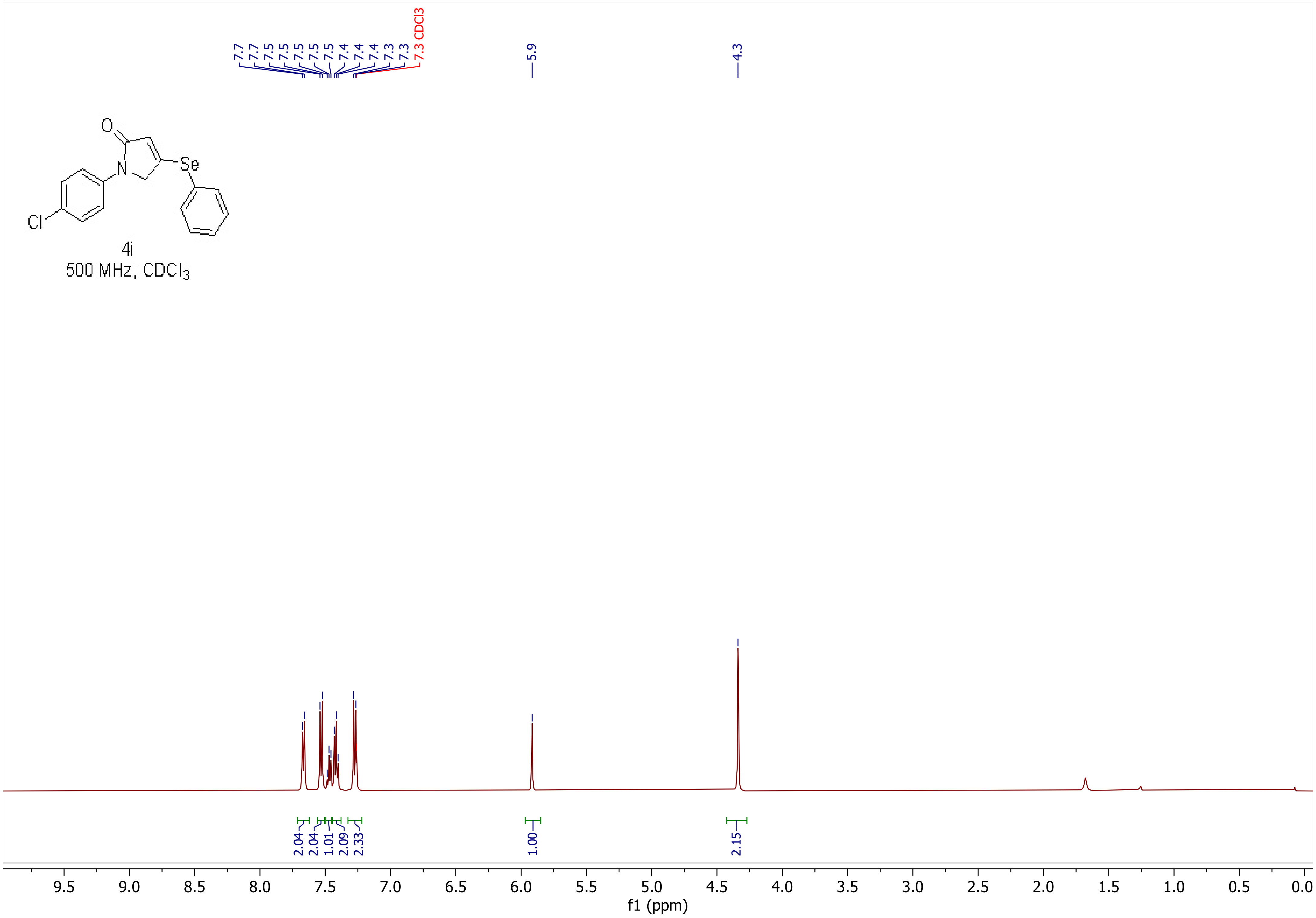

7.7
7.7
7.5
7.5
7.5
7.5
7.5
7.4
7.4
7.4
7.3
7.3
7.3 CDCl3
5.9
4.3
O
N
Se
Cl
4i
500 MHz, CDCl3
2.04
2.04
1.01
2.09
2.33
1.00
2.15
9.5
9.0
8.5
8.0
7.5
7.0
6.5
6.0
5.5
5.0
4.5
4.0
3.5
3.0
2.5
2.0
1.5
1.0
0.5
0.0
f1 (ppm)

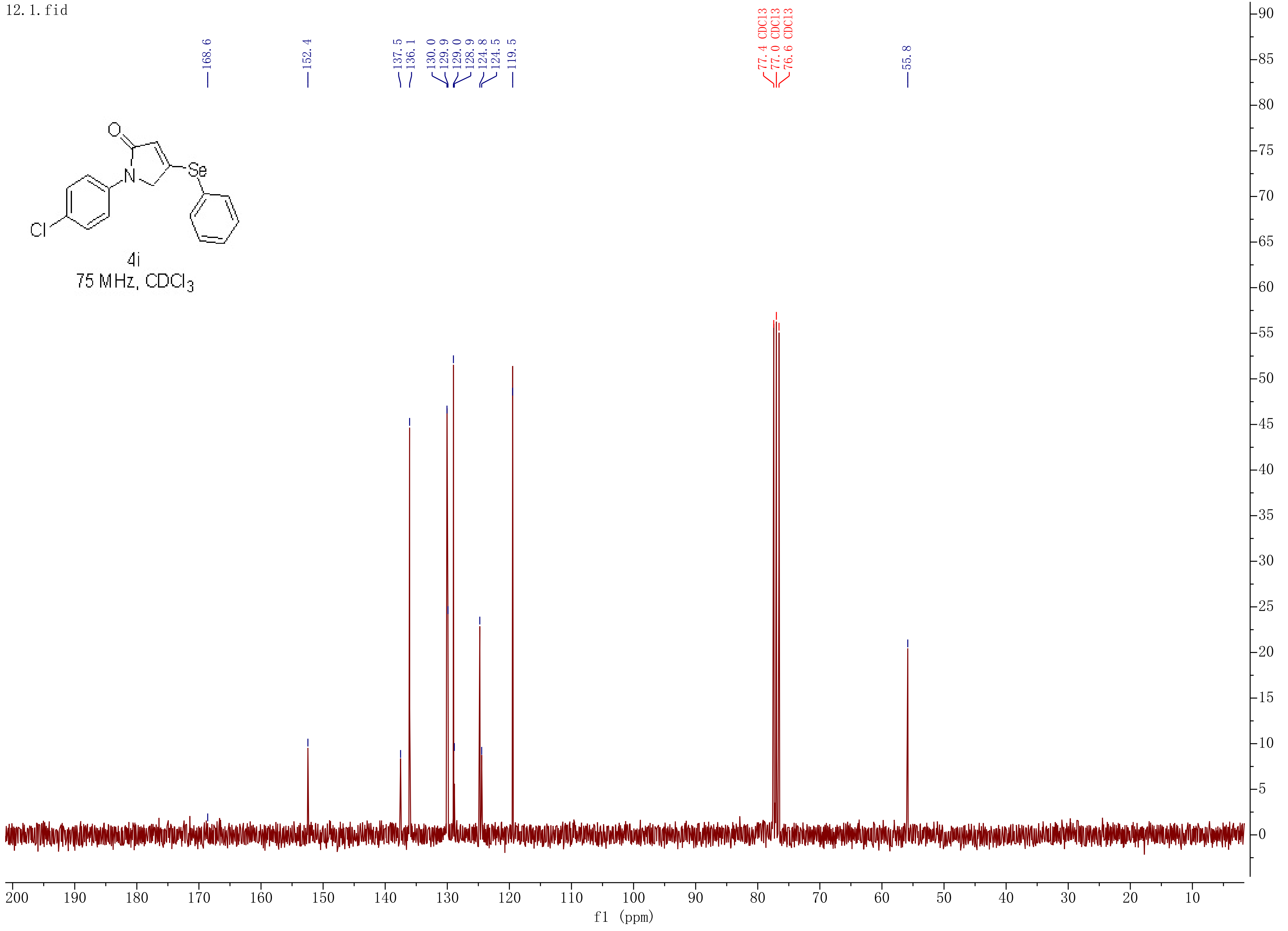

4i
75 MHz, $CDCl_3$

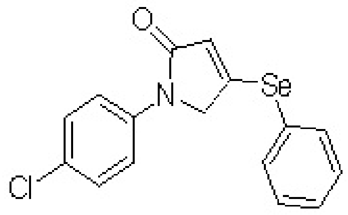

Qualitative Compound Identification Report

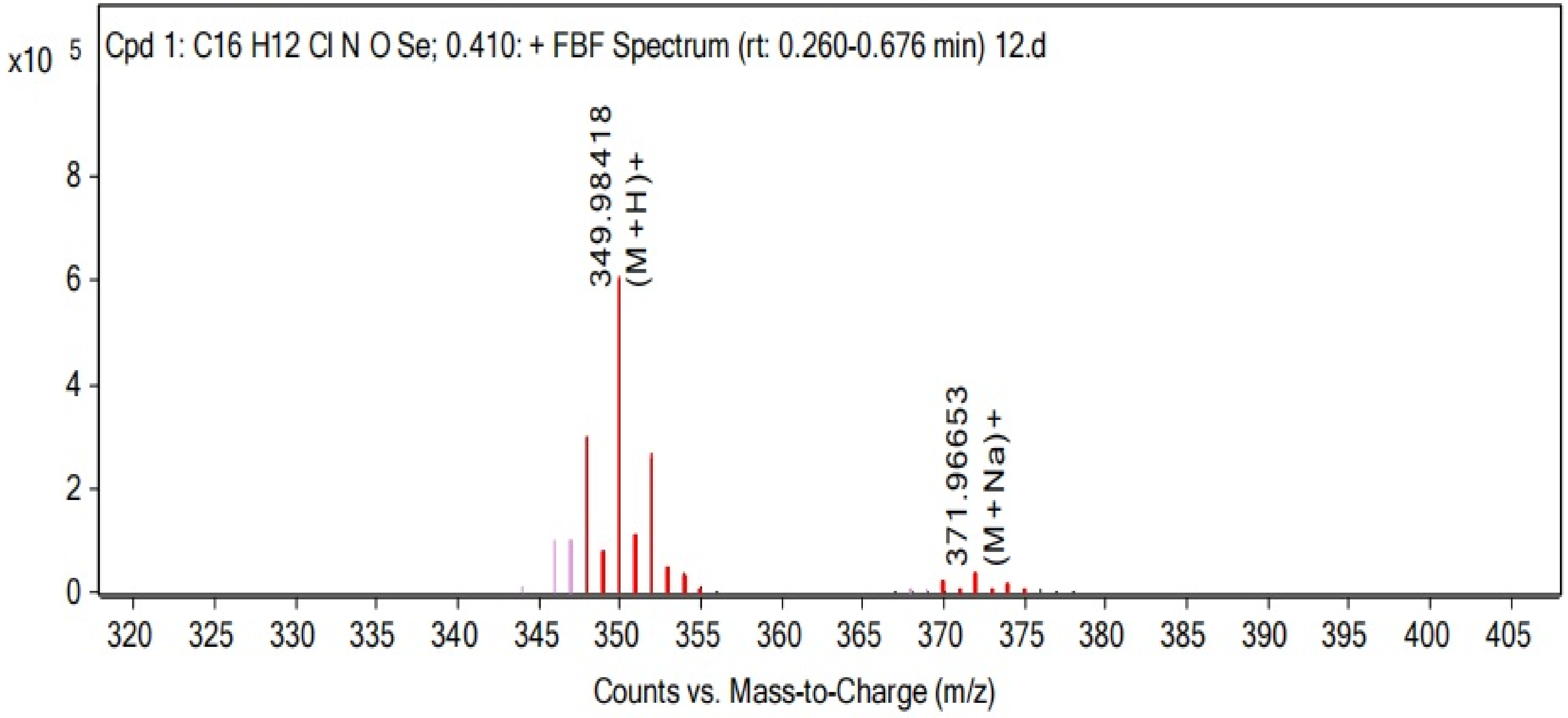

MS Spectrum Peak List

m/z	z	Abund	Ion
347.98518	1	299218.25	(M+H)+
348.98691	1	76823.2	(M+H)+
349.98418	1	602705.25	(M+H)+
350.9876	1	110470.68	(M+H)+
351.98281	1	255324.66	(M+H)+

MS Spectrum

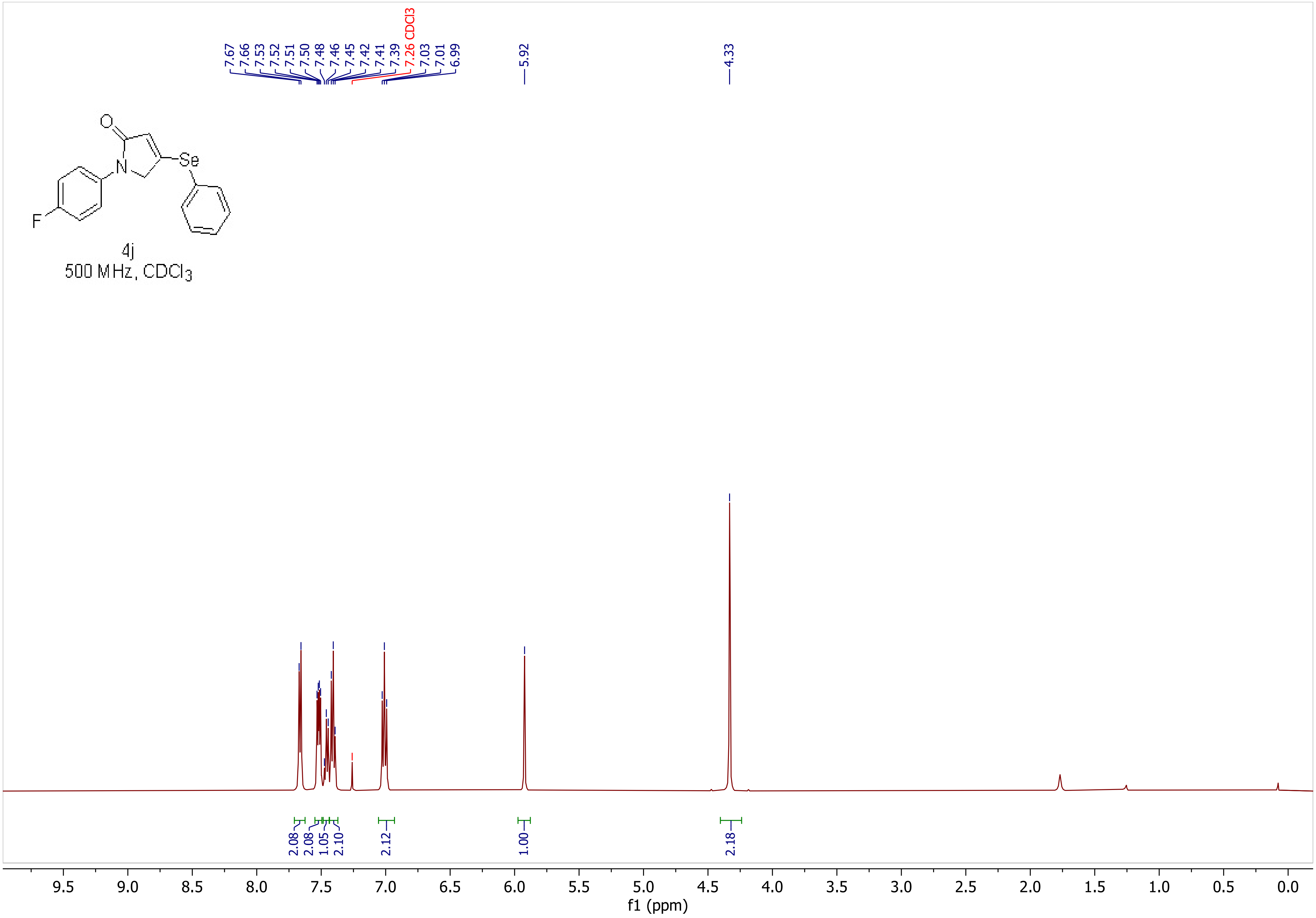
7.67
7.66
7.53
7.52
7.51
7.50
7.48
7.46
7.45
7.42
7.41
7.39
7.26 CDCl3
7.03
7.01
6.99
5.92
4.33
O
N
Se
F
4j
500 MHz, CDCl3
2.08
2.08
1.05
2.10
2.12
1.00
2.18
9.5
9.0
8.5
8.0
7.5
7.0
6.5
6.0
5.5
5.0
4.5
4.0
3.5
3.0
2.5
2.0
1.5
1.0
0.5
0.0
f1 (ppm)

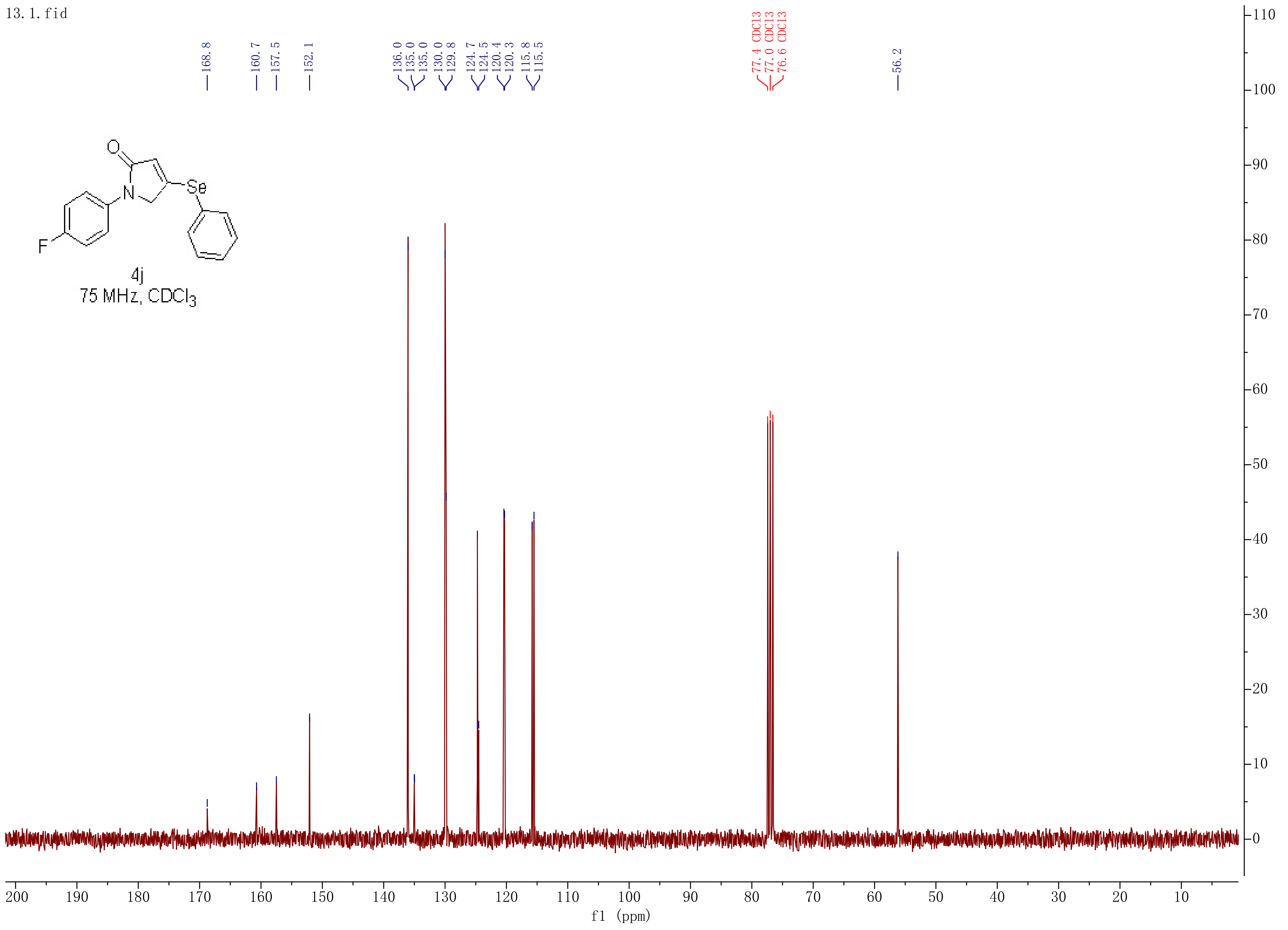
13.1.fid
168.8
160.7
157.5
152.1
136.0
135.0
135.0
130.0
129.8
124.7
124.5
120.4
120.3
115.8
115.5
77.4 CDCl3
77.0 CDCl3
76.6 CDCl3
56.2
O
N
Se
F
4j
75 MHz, CDCl3
f1 (ppm)

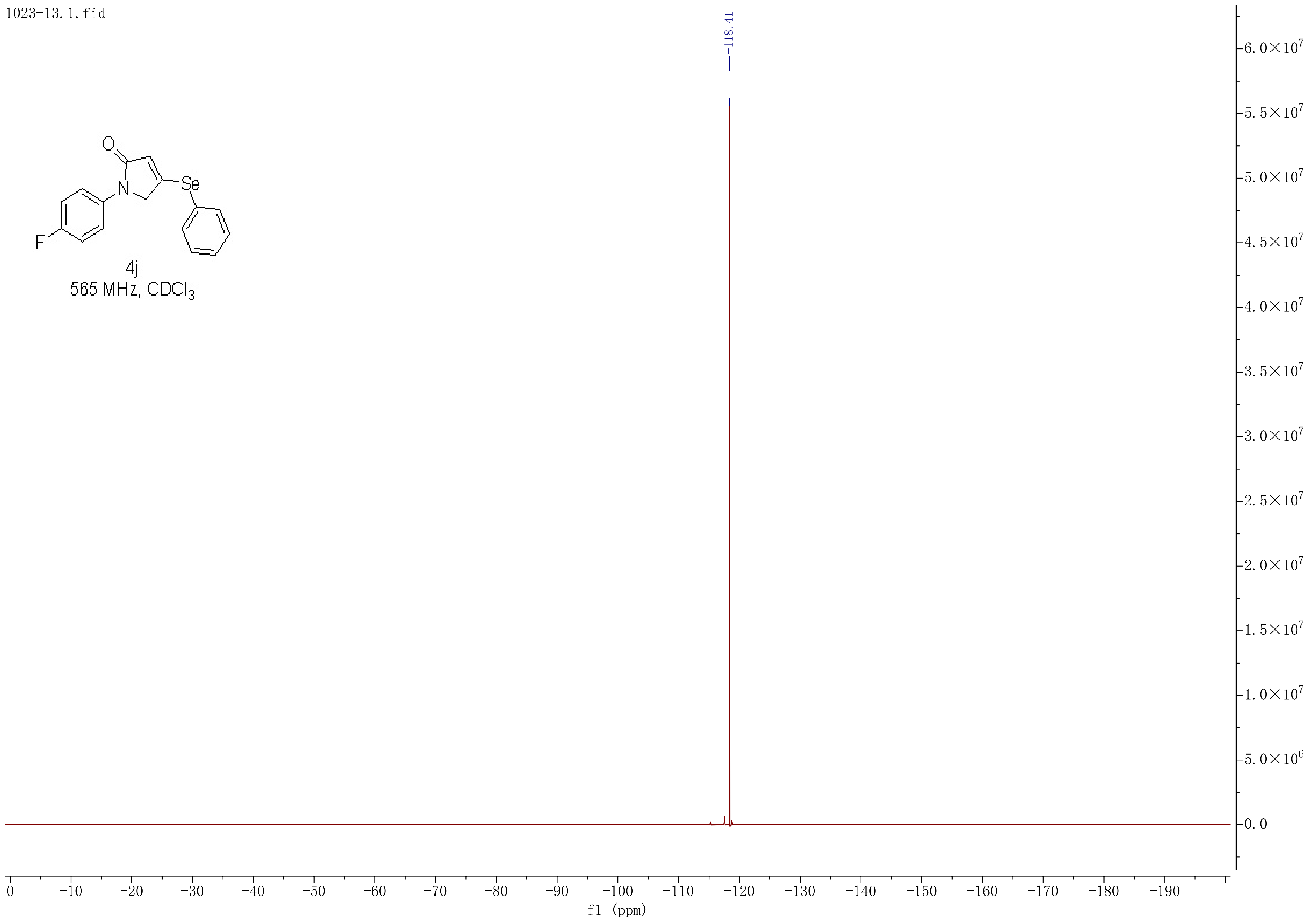
1023-13.1.fid
−118.41
4j
565 MHz, CDCl3
f1 (ppm)

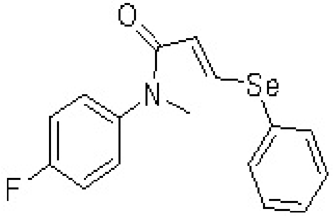

Qualitative Compound Identification Report

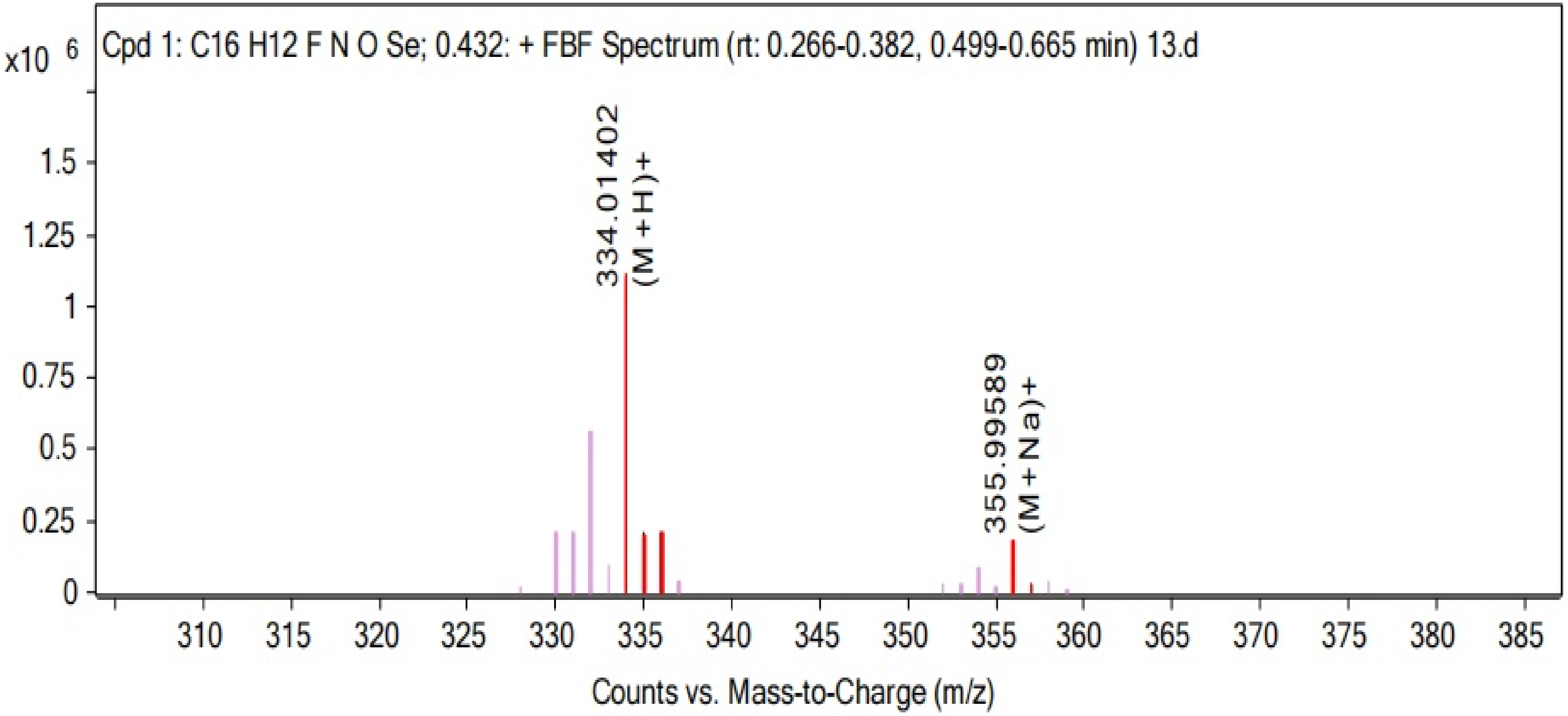

MS Spectrum Peak List

m/z	z	Abund	Ion
334.01402	1	1106909.63	(M+H)+
335.01748	1	207292.38	(M+H)+
336.0144	1	211799.13	(M+H)+
355.99589	1	174385.58	(M+Na)+
356.99953	1	33469.16	(M+Na)+

MS Spectrum

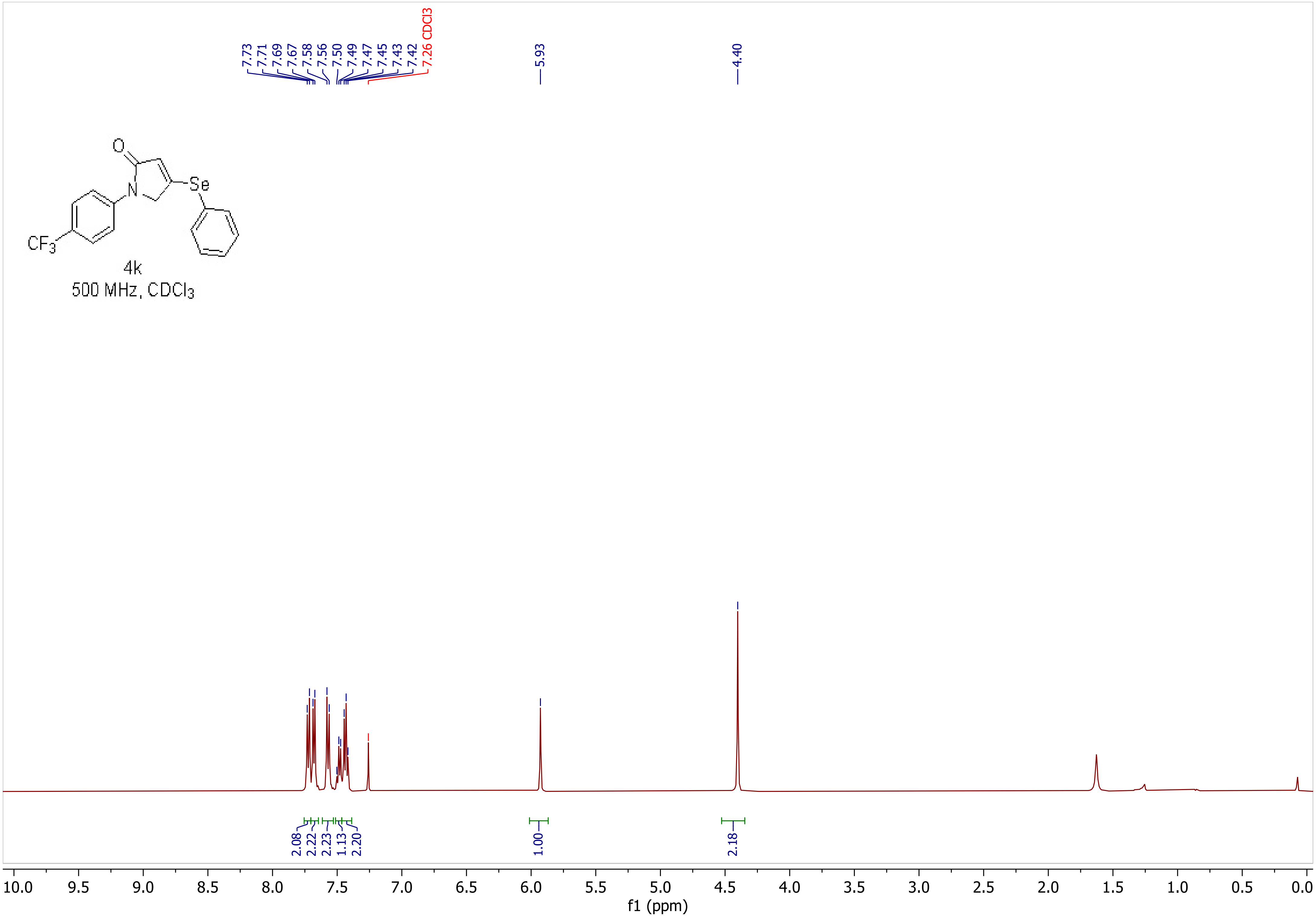
7.73
7.71
7.69
7.67
7.58
7.56
7.50
7.49
7.47
7.45
7.43
7.42
7.26 CDCl3
5.93
4.40
O
N
Se
CF3
4k
500 MHz, CDCl3
2.08
2.22
2.23
1.13
2.20
1.00
2.18
10.0
9.5
9.0
8.5
8.0
7.5
7.0
6.5
6.0
5.5
5.0
4.5
4.0
3.5
3.0
2.5
2.0
1.5
1.0
0.5
0.0
f1 (ppm)

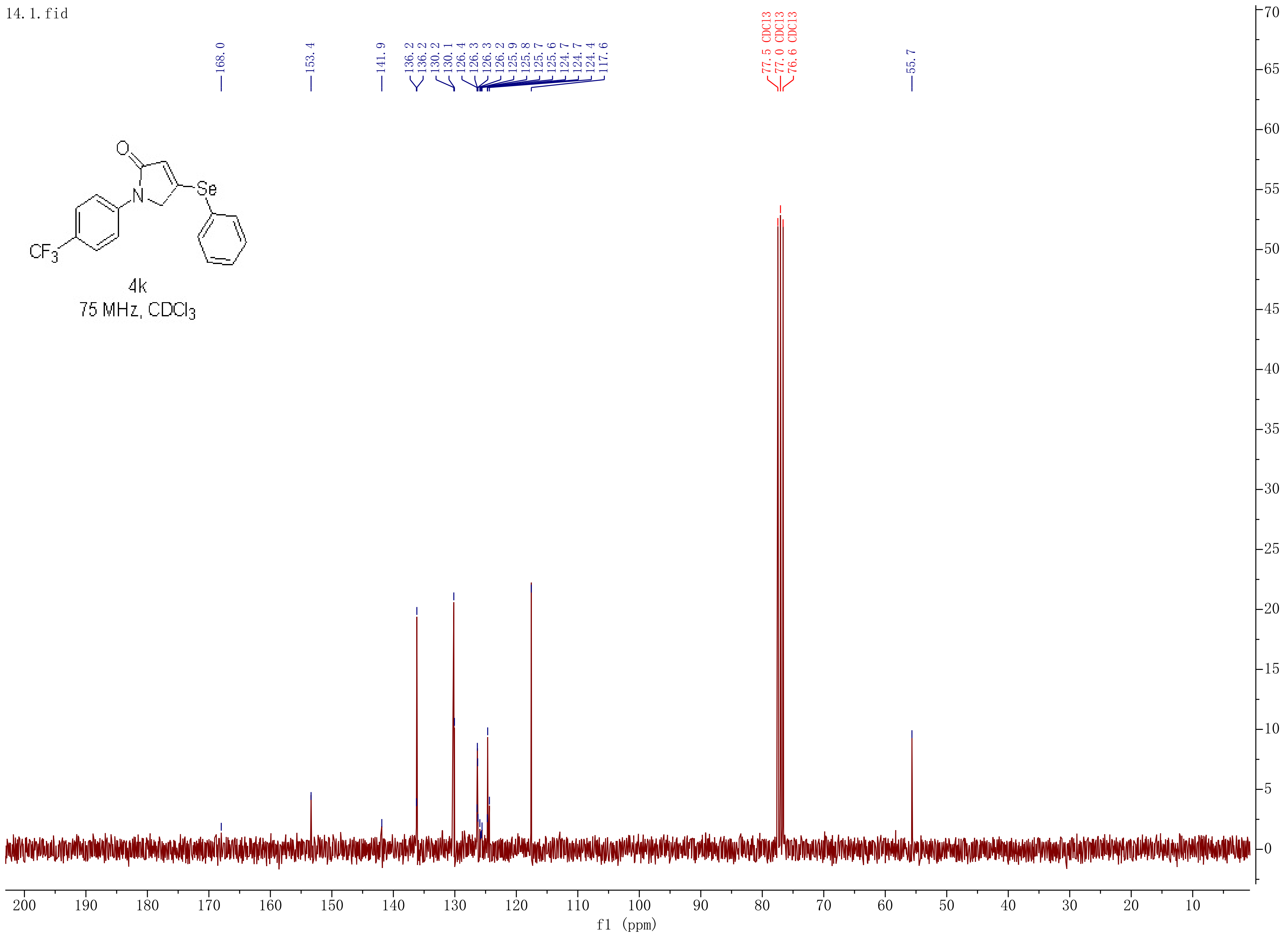
14.1.fid
168.0
153.4
141.9
136.2
136.2
130.2
130.1
126.4
126.3
126.3
126.2
125.9
125.8
125.7
125.6
124.7
124.7
124.4
117.6
77.5 CDCl3
77.0 CDCl3
76.6 CDCl3
55.7
O
N
Se
CF3
4k
75 MHz, CDCl3
200 190 180 170 160 150 140 130 120 110 100 90 80 70 60 50 40 30 20 10
f1 (ppm)
70 65 60 55 50 45 40 35 30 25 20 15 10 5 0

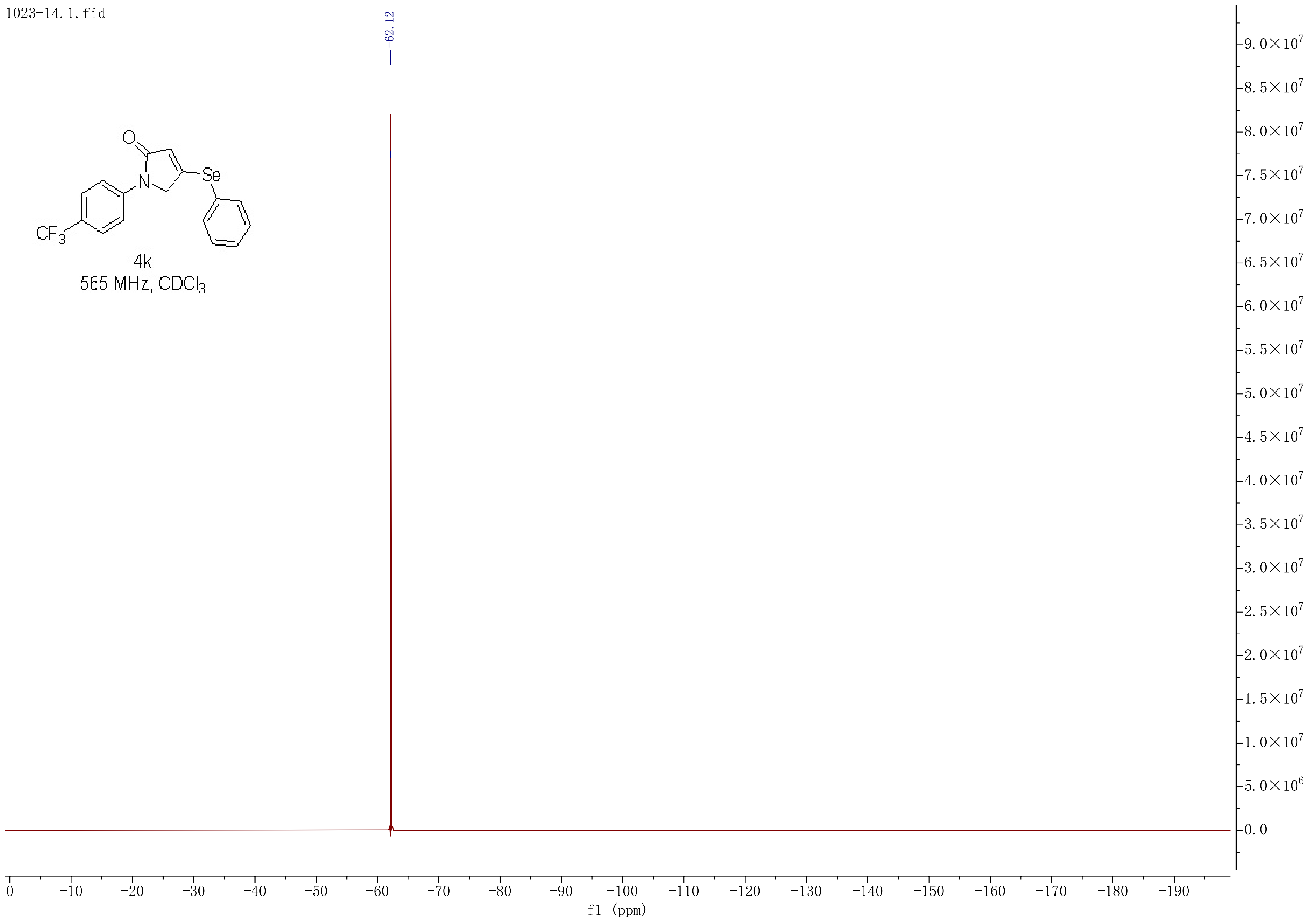
1023-14.1.fid
-62.12
4k
565 MHz, CDCl3
f1 (ppm)

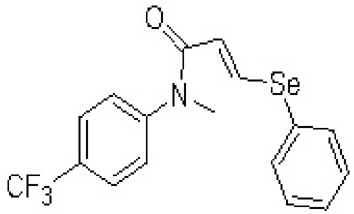

Qualitative Compound Identification Report

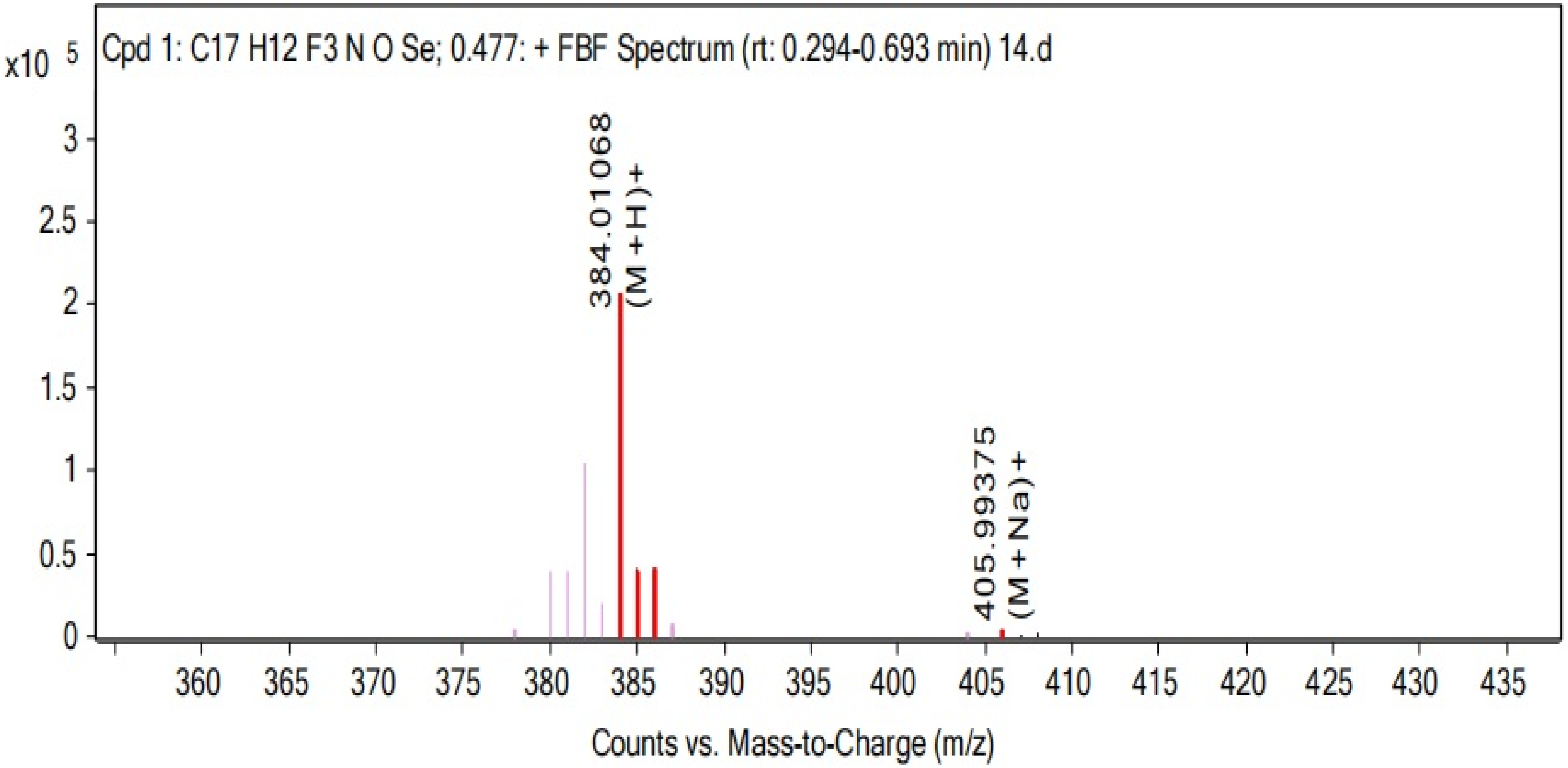

MS Spectrum Peak List

m/z	z	Abund	Ion
384.01068	1	203071.31	(M+H)+
385.01378	1	41295.7	(M+H)+
386.01103	1	40636.79	(M+H)+
405.99375	1	3670.61	(M+Na)+
407.99285	1	1353.14	(M+Na)+

MS Spectrum

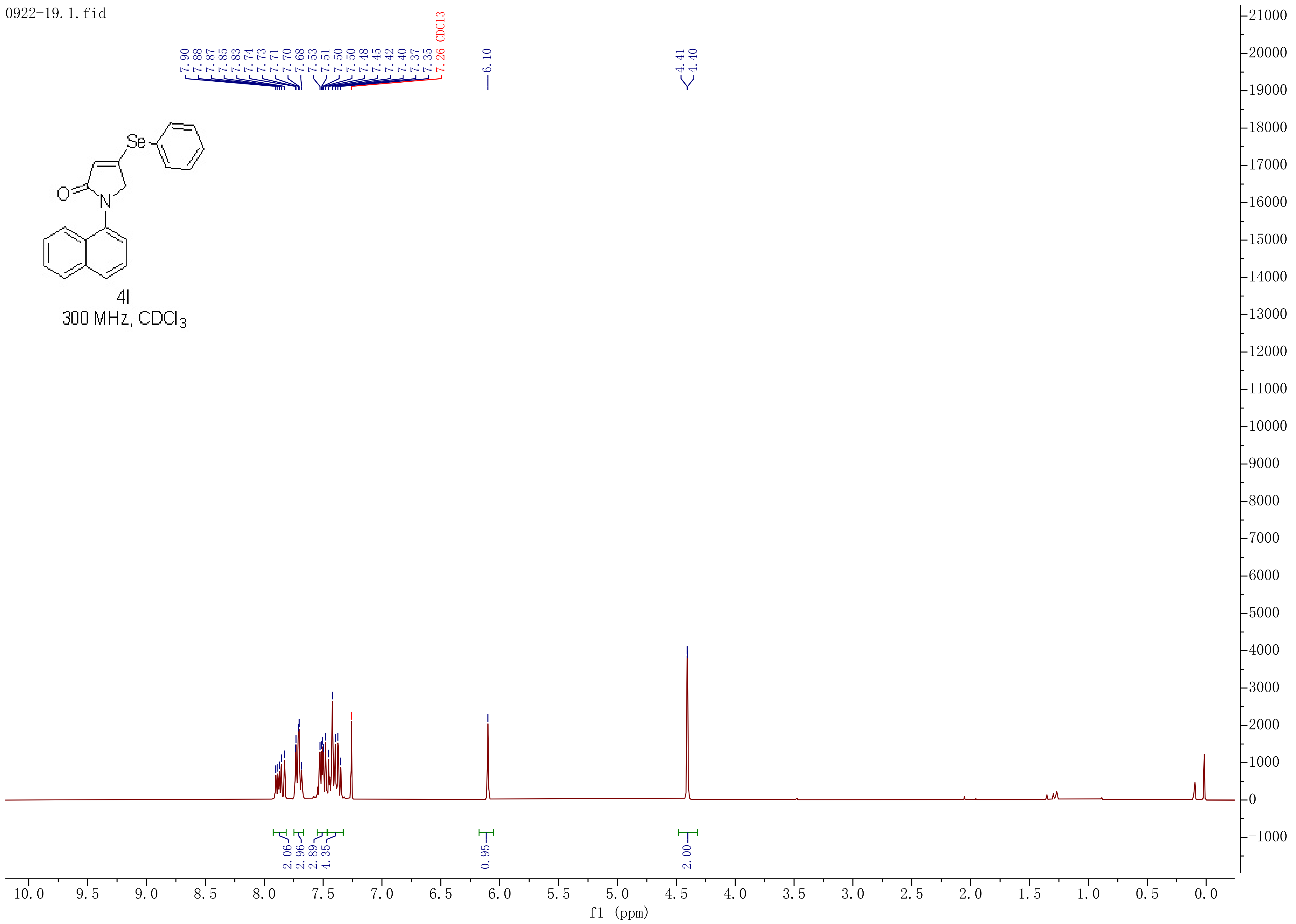
0922-19.1.fid
7.90
7.88
7.87
7.85
7.83
7.74
7.73
7.71
7.70
7.68
7.53
7.51
7.50
7.50
7.48
7.45
7.42
7.40
7.37
7.35
7.26 CDCl3
6.10
4.41
4.40
Se
O
N
4l
300 MHz, CDCl3
2.06
2.96
2.89
4.35
0.95
2.00
f1 (ppm)

0929-19.1.fid

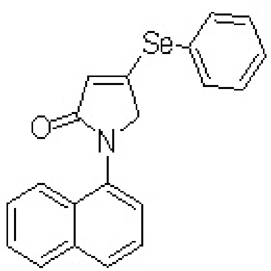

4l
75 MHz, $CDCl_3$

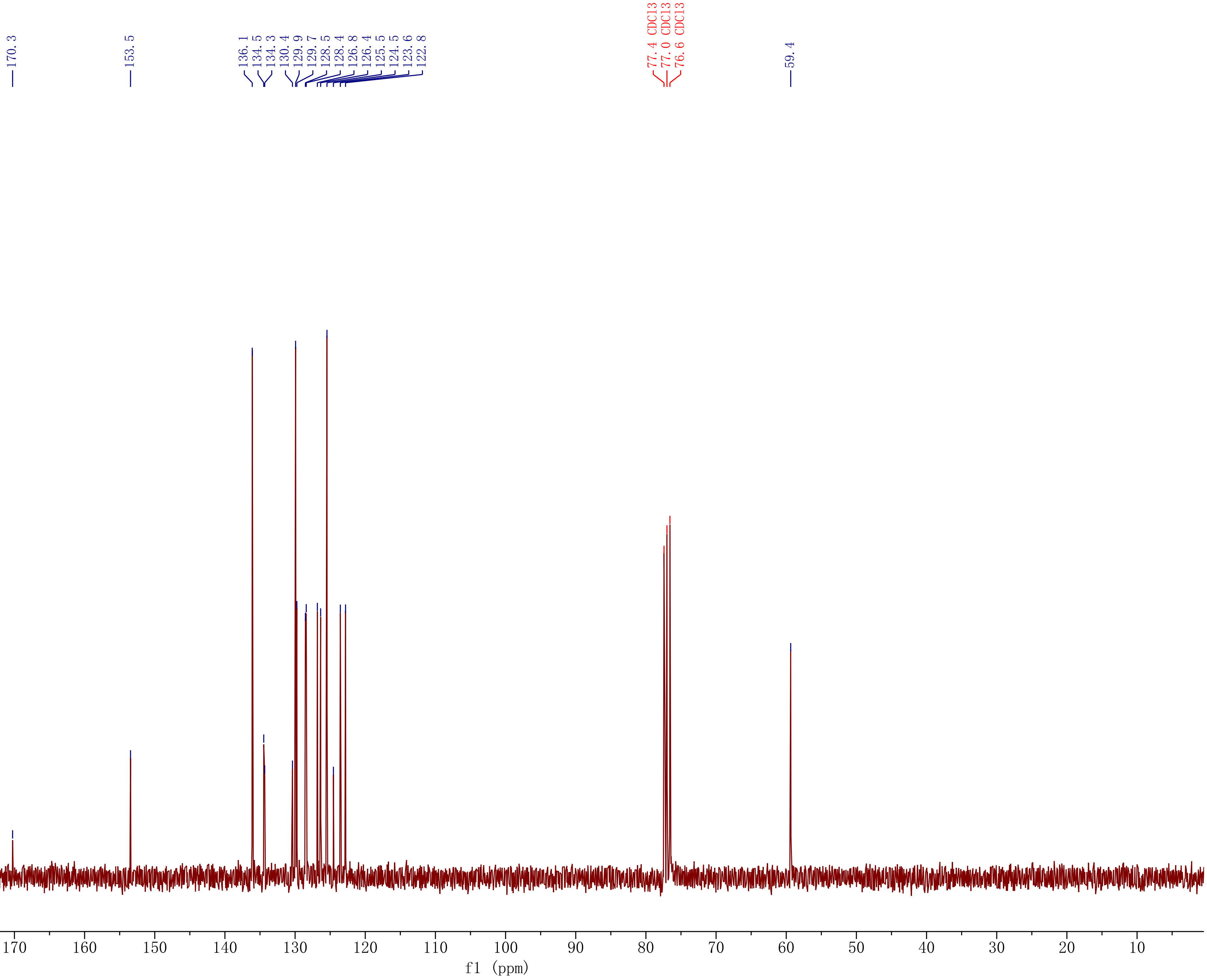

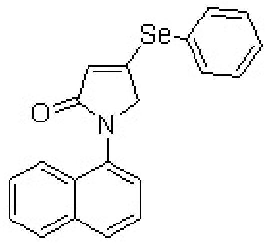

Qualitative Compound Identification Report

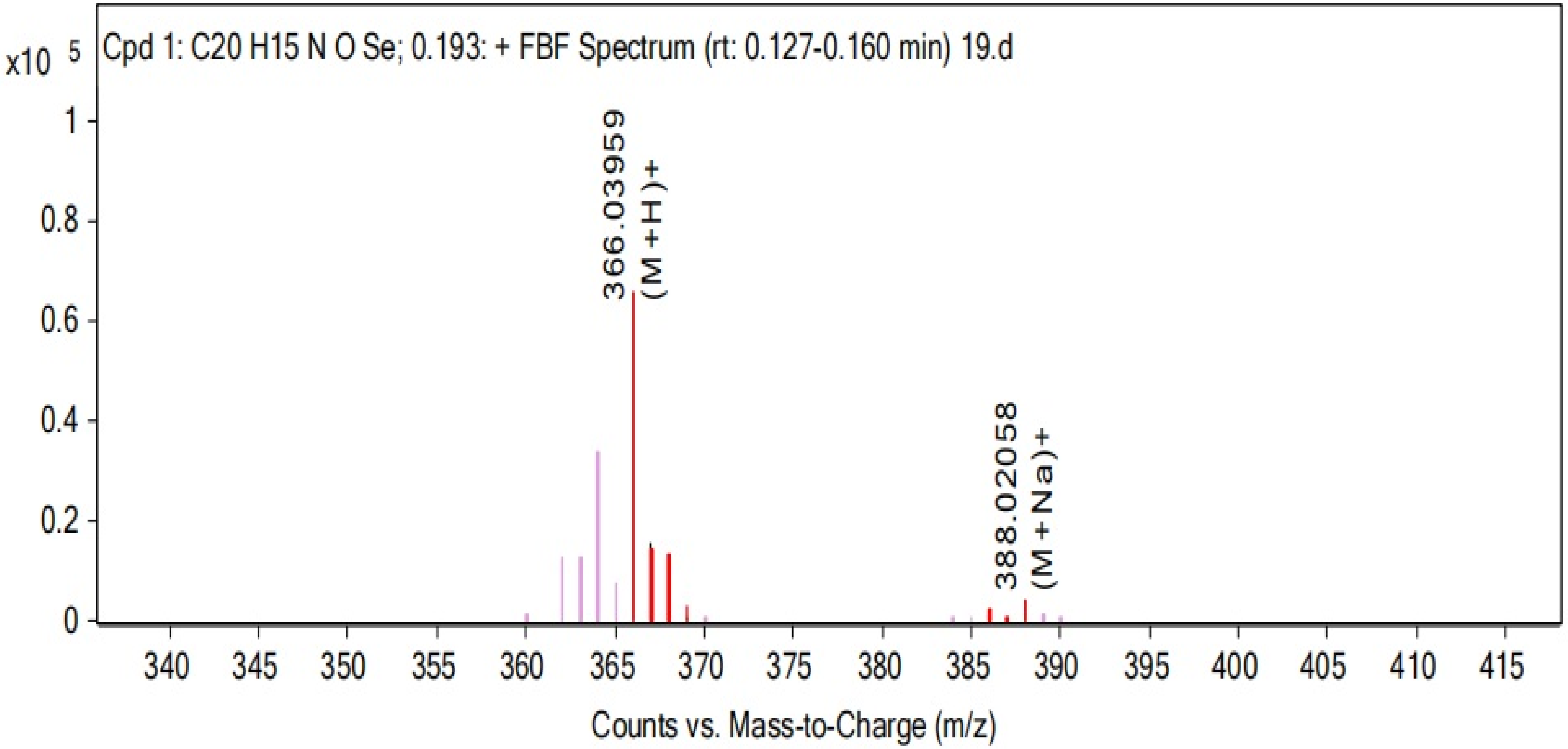

MS Spectrum Peak List

m/z	z	Abund	Ion
366.03959	1	65868.78	(M+H)+
367.04261	1	15216.35	(M+H)+
368.04006	1	12015.7	(M+H)+
386.02771	1	1144.04	(M+Na)+
388.02058	1	4029.54	(M+Na)+

MS Spectrum

Y-0824-9-H.1.fid

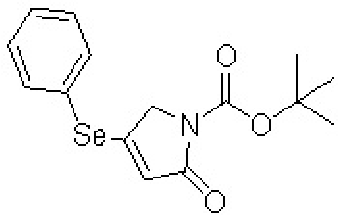

4m
500 MHz, CDCl3

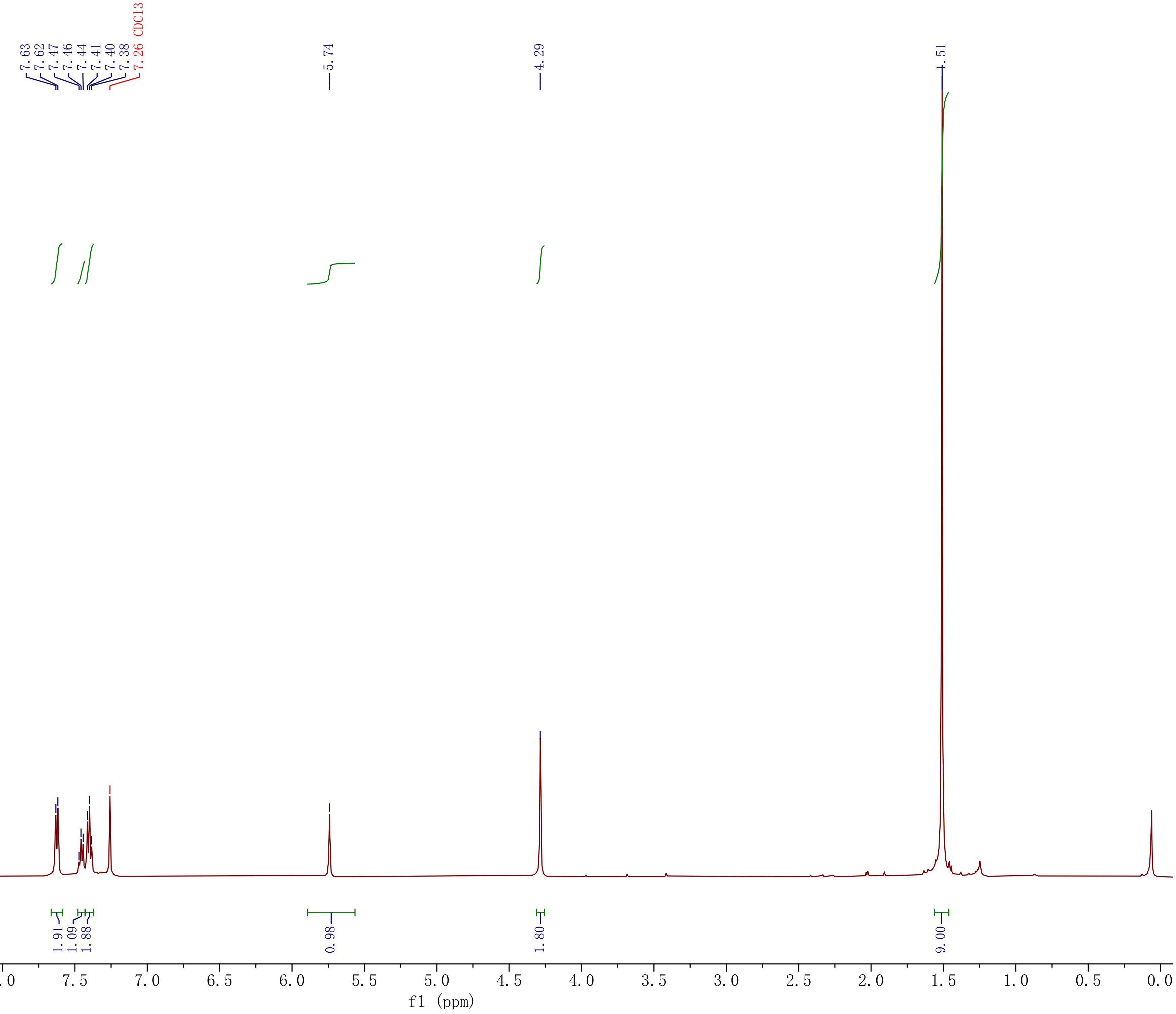

Y-0824-9-C.1.fid

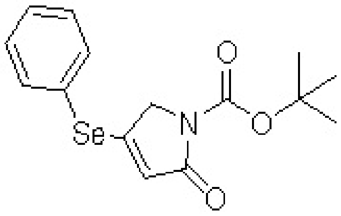

4m
126 MHz, $CDCl_3$

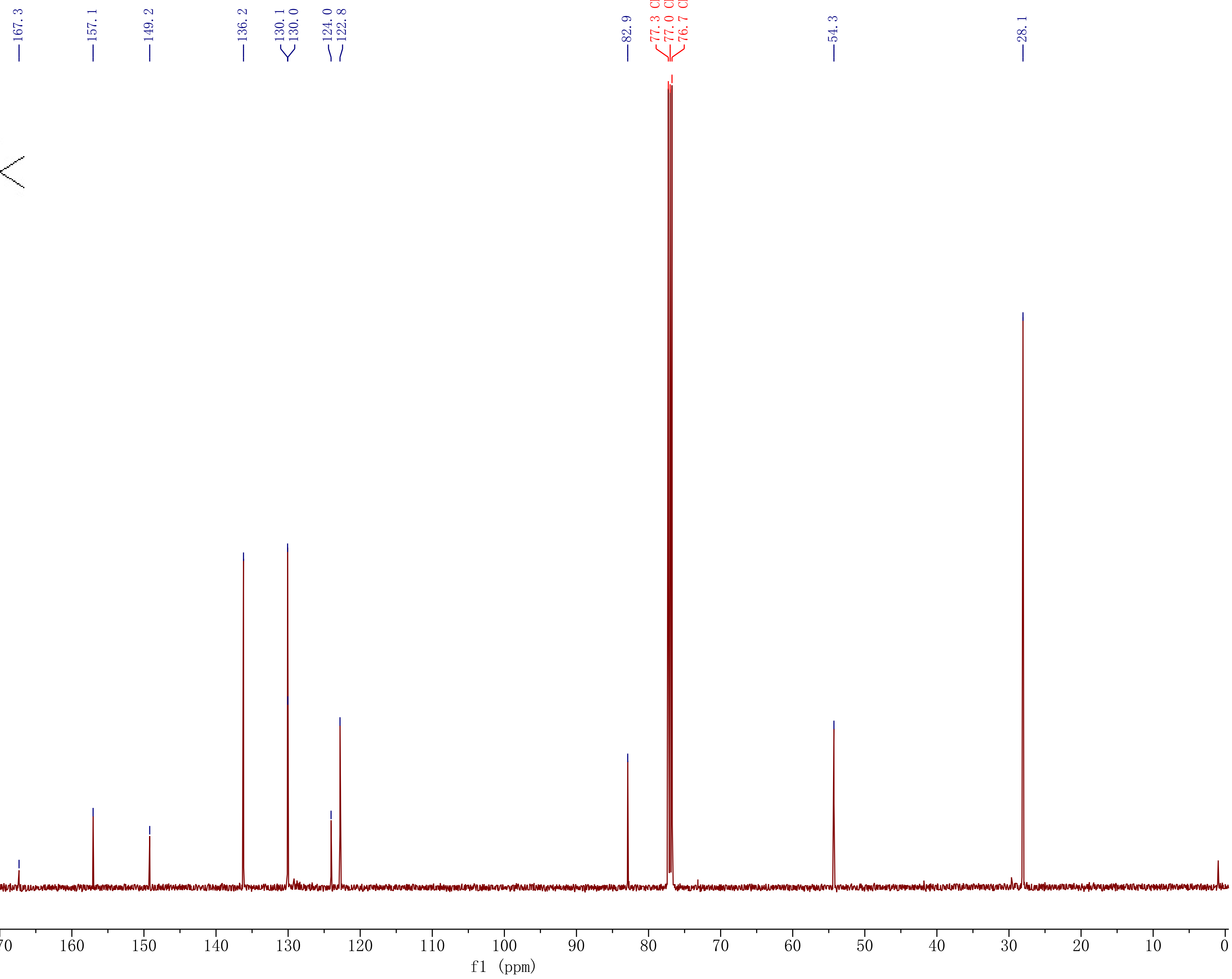

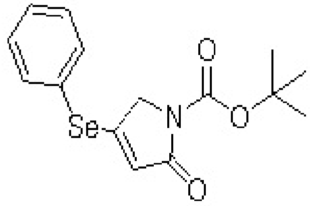

Qualitative Compound Identification Report

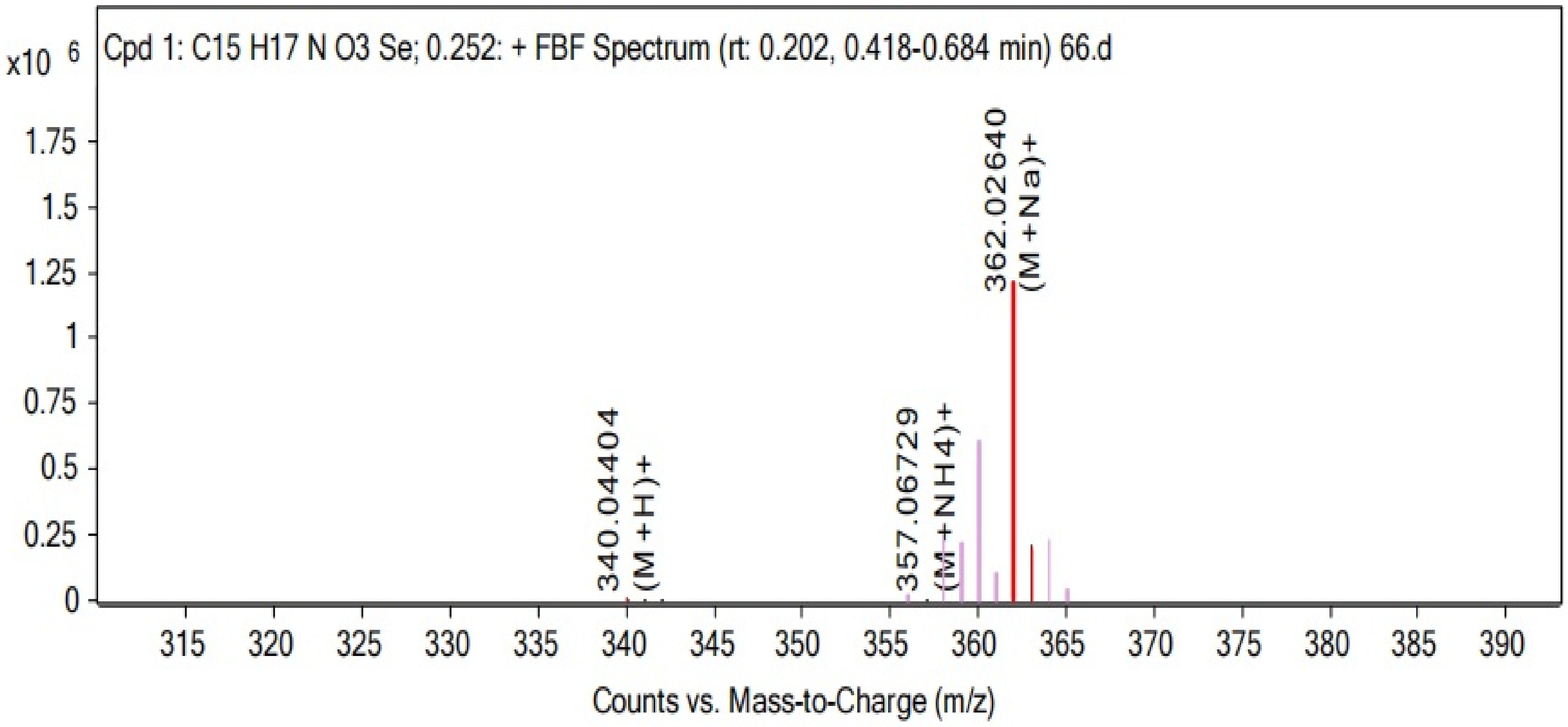

MS Spectrum Peak List

m/z	z	Abund	Ion
340.04404	1	5256.3	(M+H)+
341.04284	1	685.31	(M+H)+
342.03738	1	390.6	(M+H)+
357.06729	1	2663.63	(M+NH4)+
362.0264	1	1205944.88	(M+Na)+
363.02992	1	210941.25	(M+Na)+

MS Spectrum

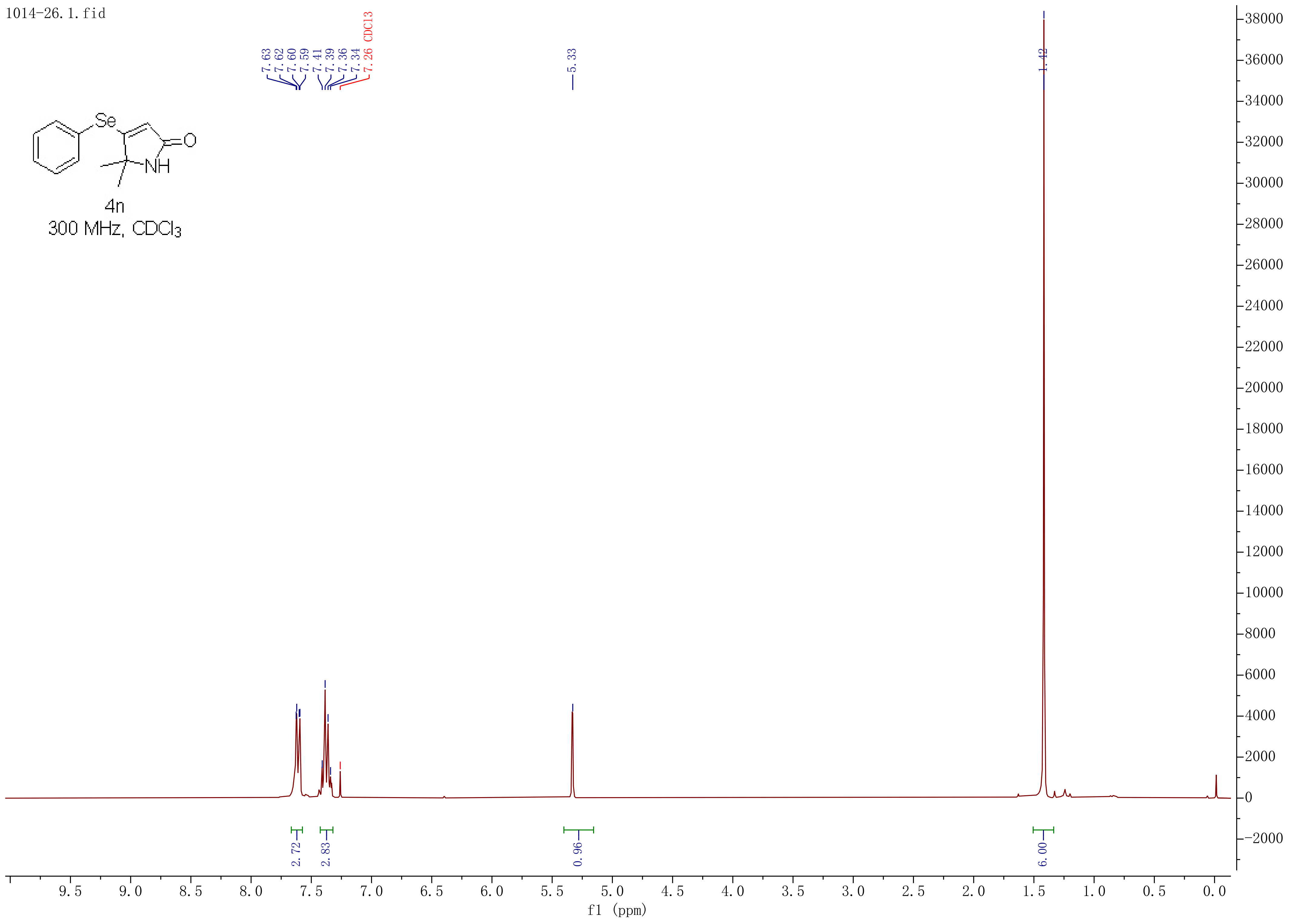

1014-26.1.fid
7.63
7.62
7.60
7.59
7.41
7.39
7.36
7.34
7.26 CDCl3
5.33
1.42
Se
O
NH
4n
300 MHz, CDCl3
2.72
2.83
0.96
6.00
f1 (ppm)

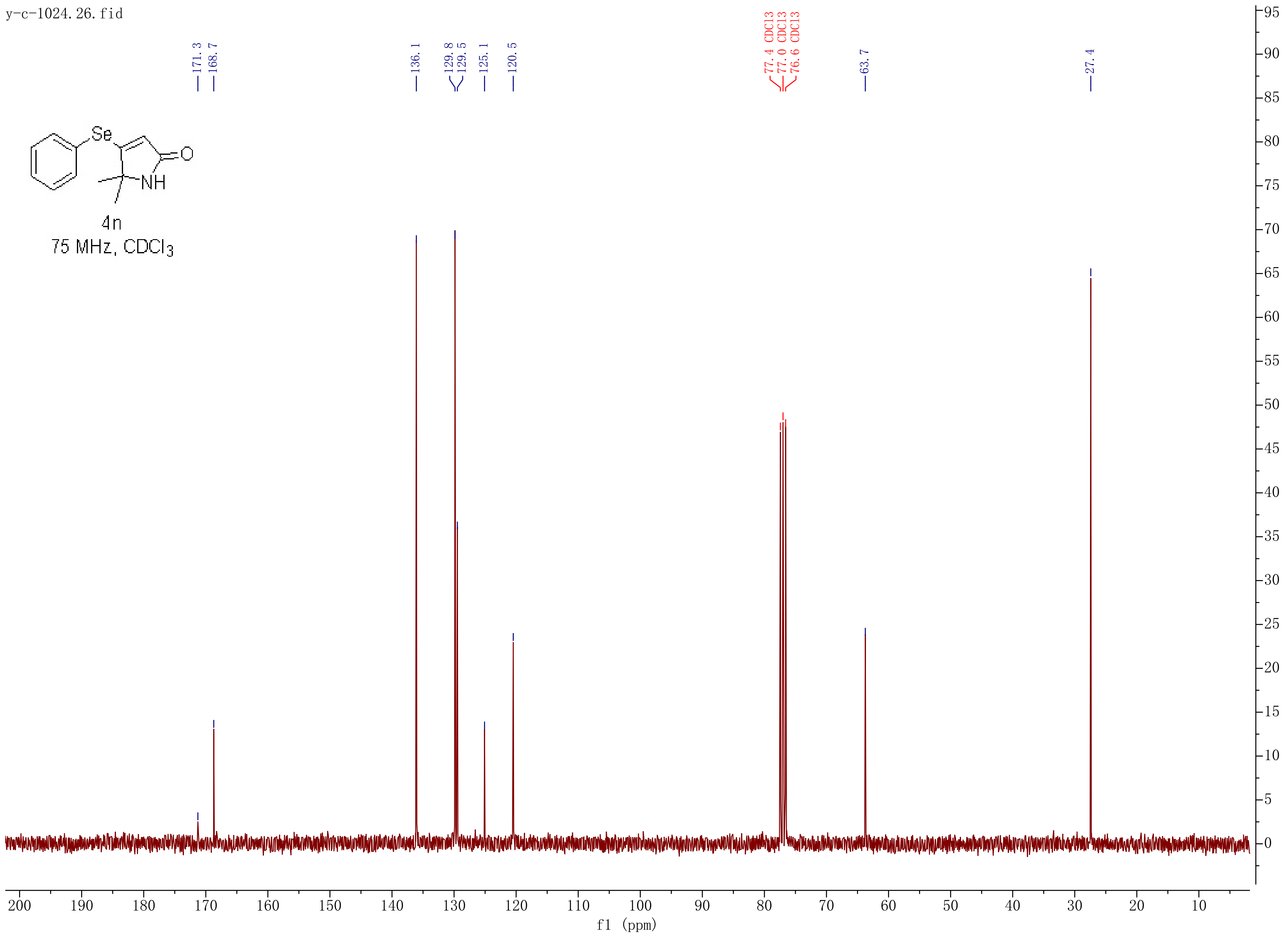

y-c-1024.26.fid
171.3
168.7
136.1
129.8
129.5
125.1
120.5
77.4 CDCl3
77.0 CDCl3
76.6 CDCl3
63.7
27.4
Se
O
NH
4n
75 MHz, CDCl3
f1 (ppm)

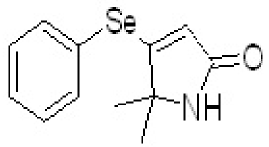

Qualitative Compound Identification Report

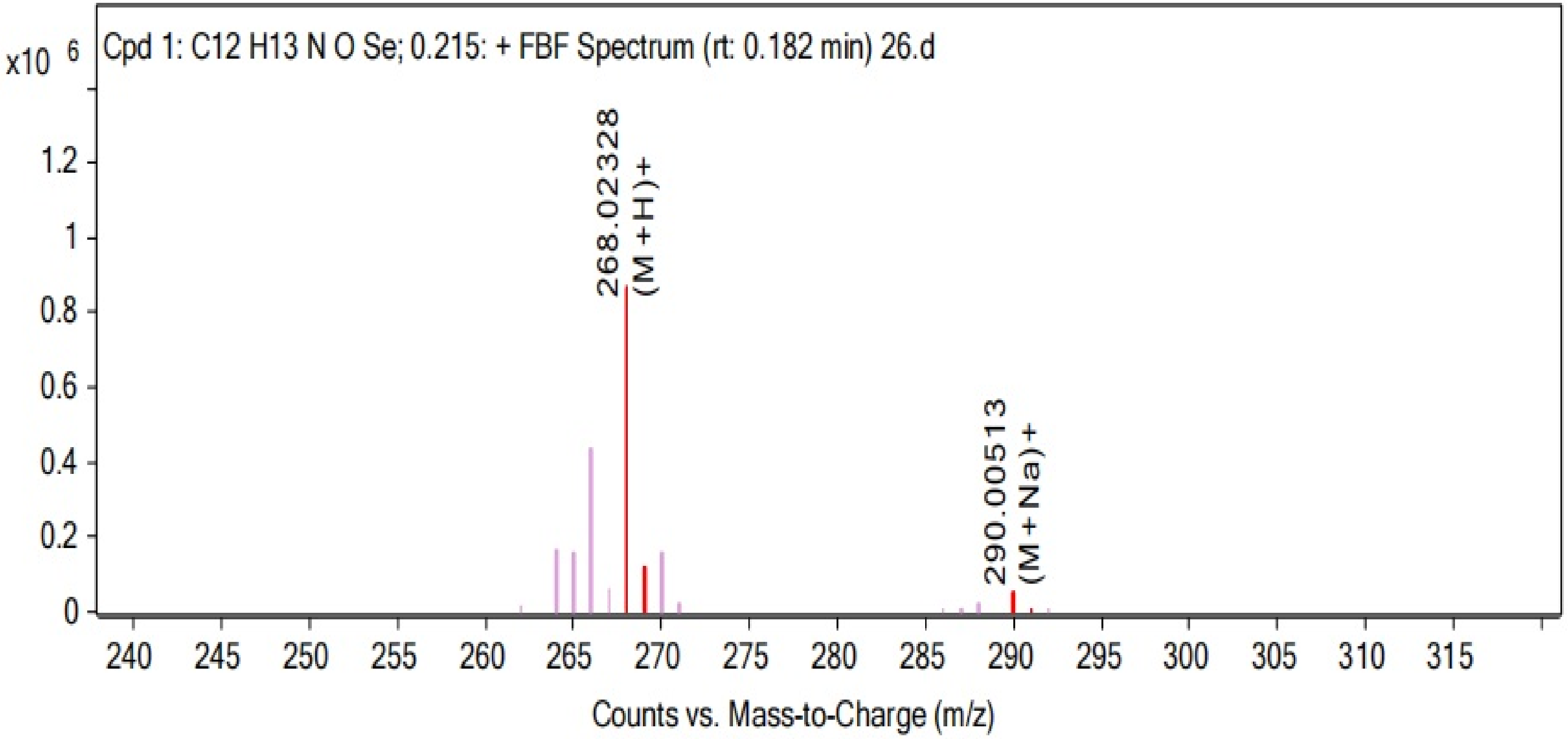

MS Spectrum Peak List

m/z	z	Abund	Ion
268.02328	1	867573.19	(M+H)+
269.02679	1	109681.84	(M+H)+
290.00513	1	51672.77	(M+Na)+
291.00874	1	7131.75	(M+Na)+

MS Spectrum

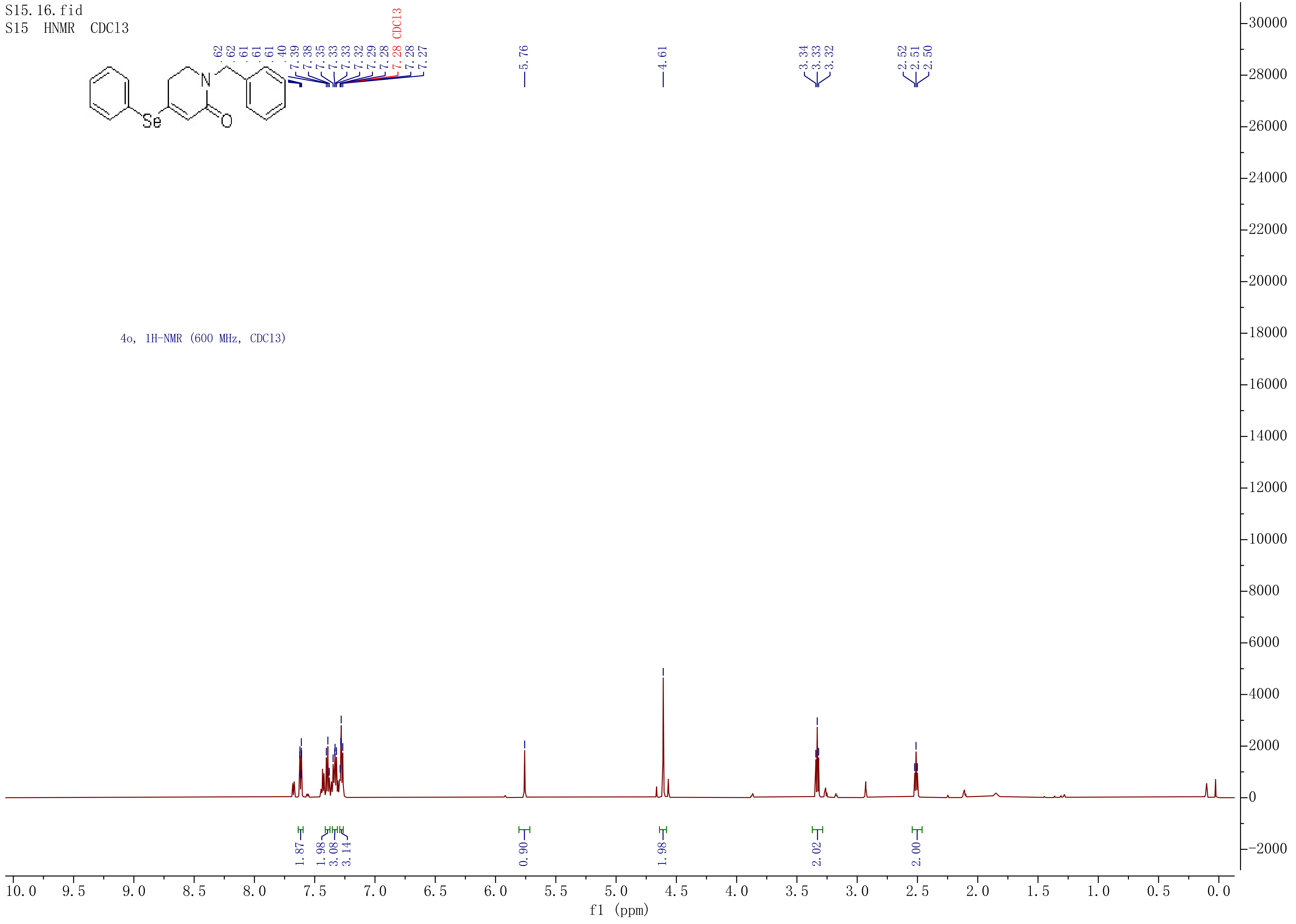

4o, 1H-NMR (600 MHz, CDCl3)

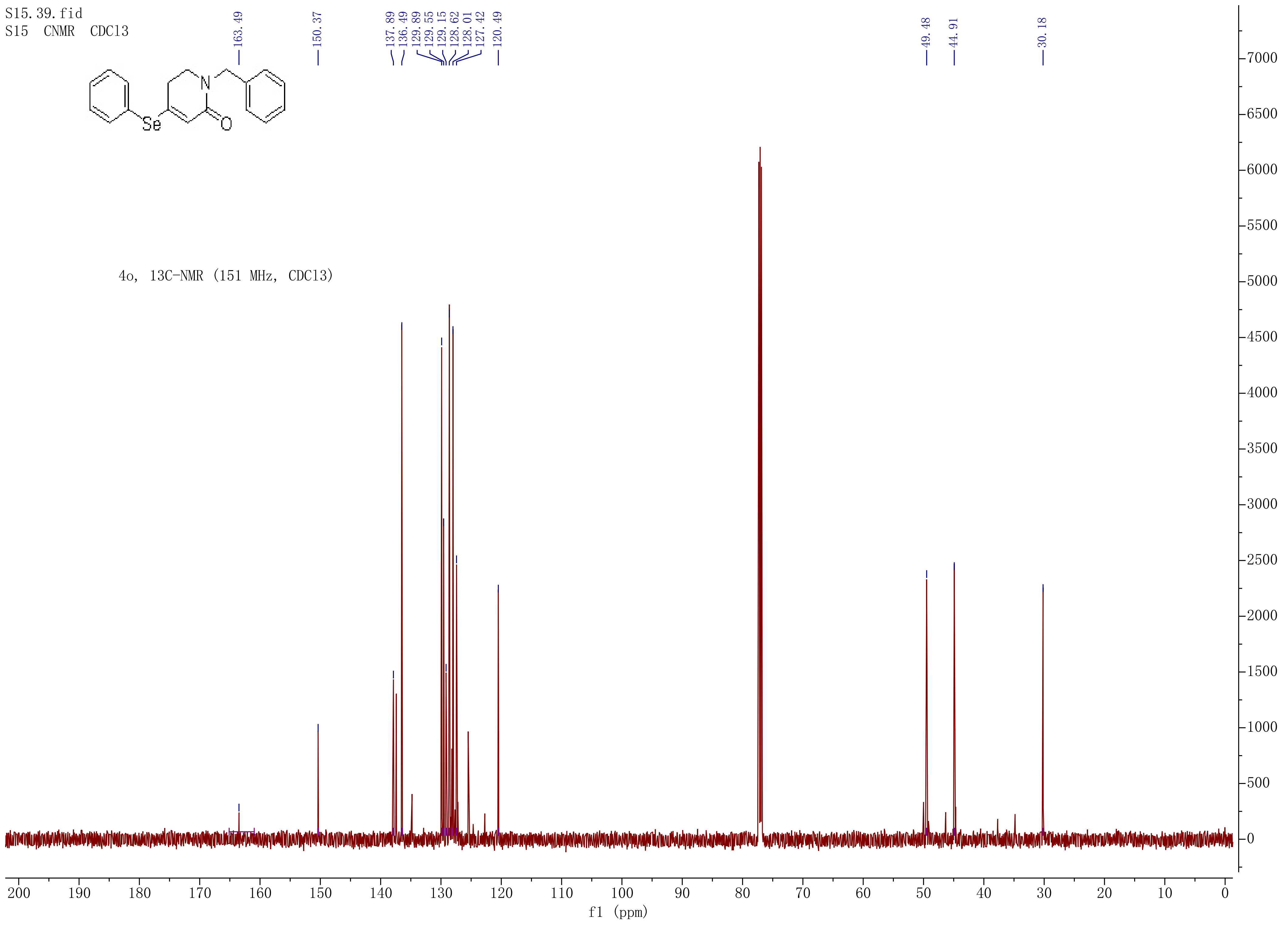

S15.39.fid
S15 CNMR CDCl3
Se
N
O
4o, 13C-NMR (151 MHz, CDCl3)
163.49
150.37
137.89
136.49
129.89
129.55
129.15
128.62
128.01
127.42
120.49
49.48
44.91
30.18
f1 (ppm)

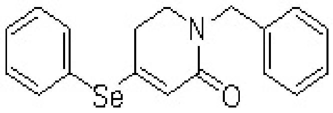

Qualitative Compound Identification Report

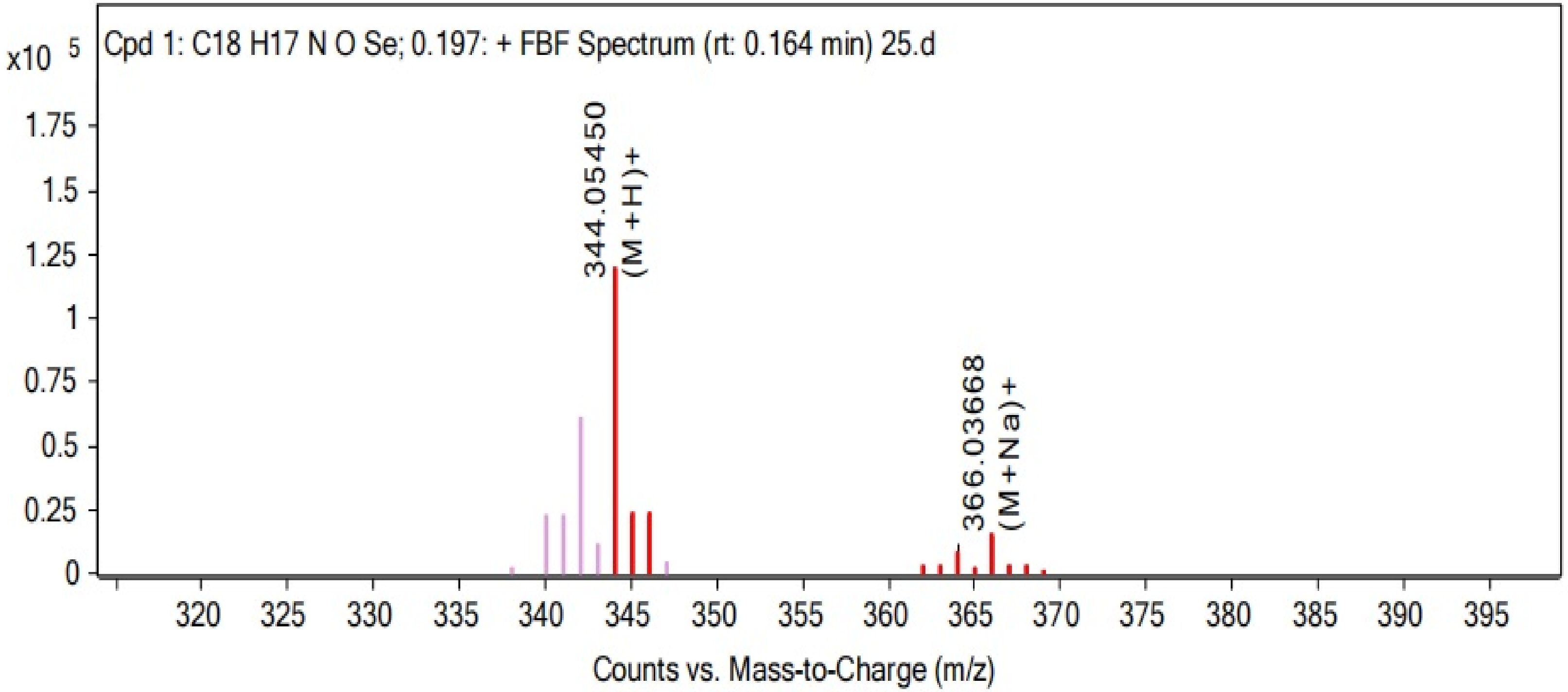

MS Spectrum Peak List

m/z	z	Abund	Ion
344.0545	1	119251.09	(M+H)+
345.058	1	23223.55	(M+H)+
346.05485	1	22478.28	(M+H)+
362.05397	1	2602.07	(M+Na)+
364.03744	1	10908.06	(M+Na)+
365.03882	1	1614.76	(M+Na)+
366.03668	1	14311.72	(M+Na)+
367.03786	1	3565.05	(M+Na)+
368.0377	1	2835.26	(M+Na)+
369.03935	1	1350.63	(M+Na)+

MS Spectrum

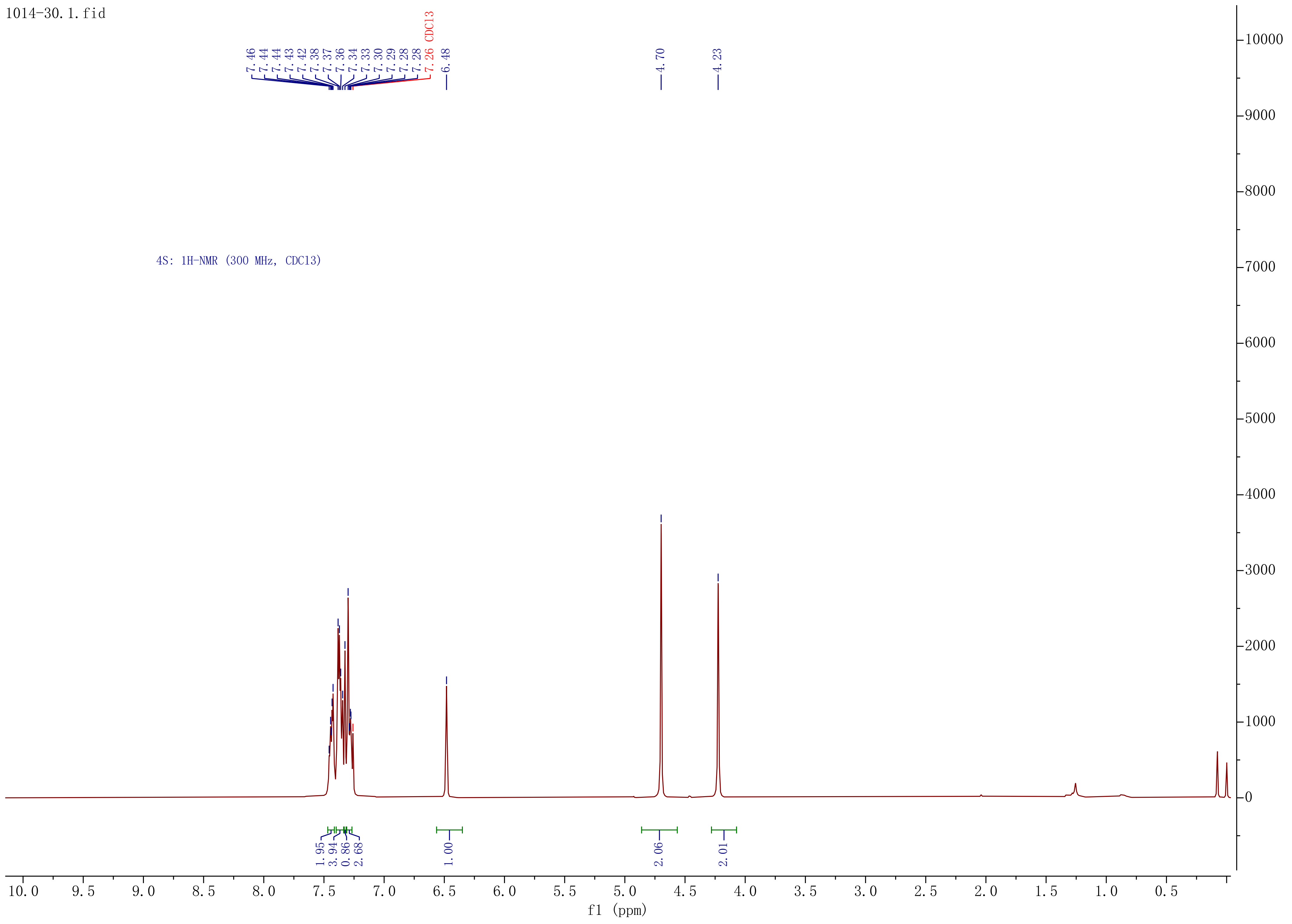
1014-30.1.fid
7.46
7.44
7.44
7.43
7.42
7.38
7.37
7.36
7.34
7.33
7.30
7.29
7.28
7.28
7.26 CDCl3
6.48
4.70
4.23
4S: 1H-NMR (300 MHz, CDCl3)
1.95
3.94
0.86
2.68
1.00
2.06
2.01
f1 (ppm)

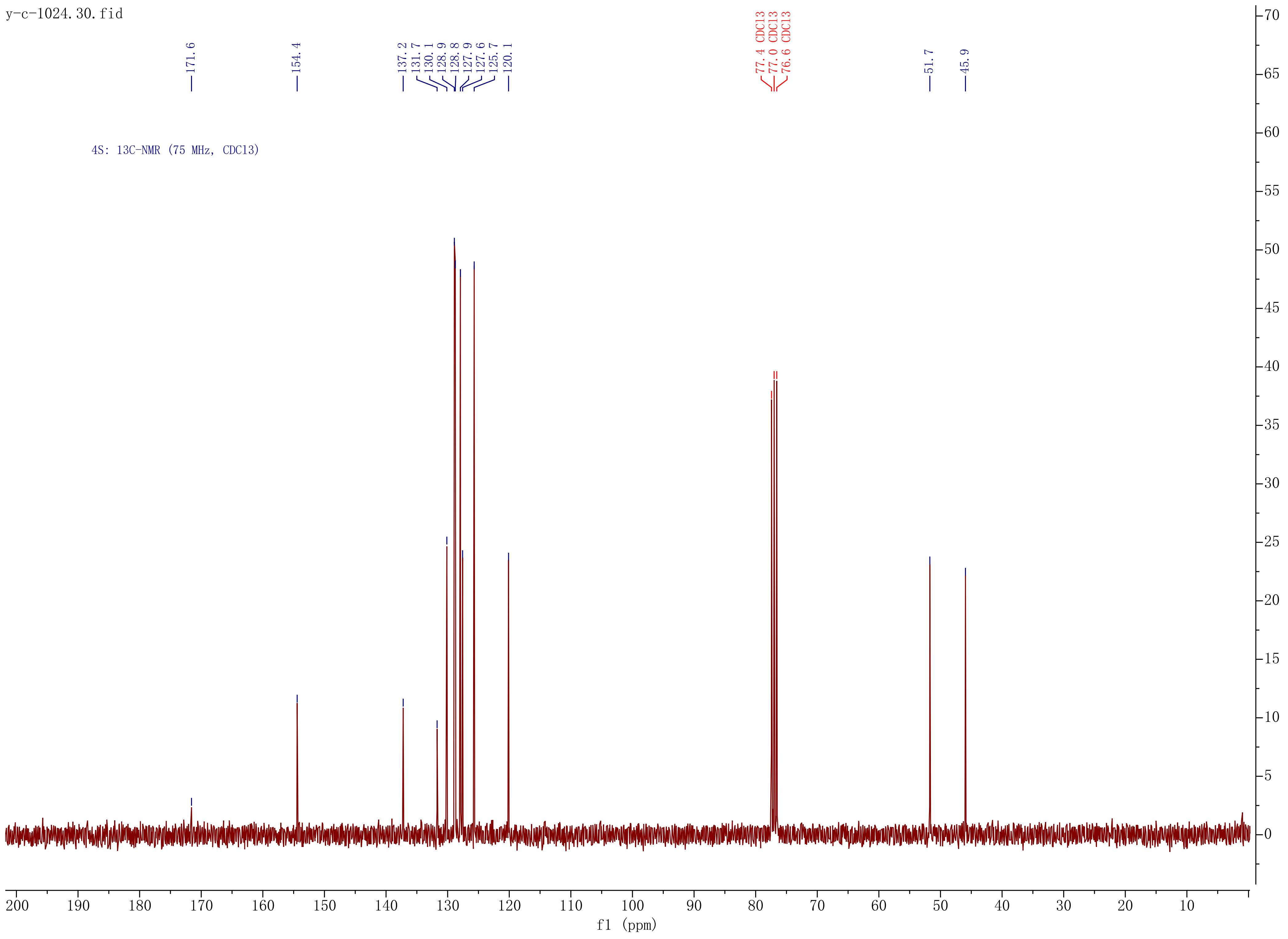

y-c-1024.30.fid
171.6
154.4
137.2
131.7
130.1
128.9
128.8
127.9
127.6
125.7
120.1
77.4 CDCl3
77.0 CDCl3
76.6 CDCl3
51.7
45.9
4S: 13C-NMR (75 MHz, CDCl3)
f1 (ppm)
